# Best genome sequencing strategies for annotation of complex immune gene families in wildlife

**DOI:** 10.1093/gigascience/giac100

**Published:** 2022-10-30

**Authors:** Emma Peel, Luke Silver, Parice Brandies, Ying Zhu, Yuanyuan Cheng, Carolyn J Hogg, Katherine Belov

**Affiliations:** School of Life and Environmental Sciences, The University of Sydney, Sydney, NSW 2006, Australia; Australian Research Council Centre of Excellence for Innovations in Peptide and Protein Science, University of Sydney, Sydney NSW 2006, Australia; School of Life and Environmental Sciences, The University of Sydney, Sydney, NSW 2006, Australia; School of Life and Environmental Sciences, The University of Sydney, Sydney, NSW 2006, Australia; Sichuan Provincial Academy of Natural Resource Sciences, Chengdu, Sichuan 610000, China; School of Life and Environmental Sciences, The University of Sydney, Sydney, NSW 2006, Australia; School of Life and Environmental Sciences, The University of Sydney, Sydney, NSW 2006, Australia; Australian Research Council Centre of Excellence for Innovations in Peptide and Protein Science, University of Sydney, Sydney NSW 2006, Australia; School of Life and Environmental Sciences, The University of Sydney, Sydney, NSW 2006, Australia; Australian Research Council Centre of Excellence for Innovations in Peptide and Protein Science, University of Sydney, Sydney NSW 2006, Australia

**Keywords:** immune gene, genome, quality, annotation, MHC, wildlife, disease

## Abstract

**Background:**

The biodiversity crisis and increasing impact of wildlife disease on animal and human health provides impetus for studying immune genes in wildlife. Despite the recent boom in genomes for wildlife species, immune genes are poorly annotated in nonmodel species owing to their high level of polymorphism and complex genomic organisation. Our research over the past decade and a half on Tasmanian devils and koalas highlights the importance of genomics and accurate immune annotations to investigate disease in wildlife. Given this, we have increasingly been asked the minimum levels of genome quality required to effectively annotate immune genes in order to study immunogenetic diversity. Here we set out to answer this question by manually annotating immune genes in 5 marsupial genomes and 1 monotreme genome to determine the impact of sequencing data type, assembly quality, and automated annotation on accurate immune annotation.

**Results:**

Genome quality is directly linked to our ability to annotate complex immune gene families, with long reads and scaffolding technologies required to reassemble immune gene clusters and elucidate evolution, organisation, and true gene content of the immune repertoire. Draft-quality genomes generated from short reads with HiC or 10× Chromium linked reads were unable to achieve this. Despite mammalian BUSCOv5 scores of up to 94.1% amongst the 6 genomes, automated annotation pipelines incorrectly annotated up to 59% of manually annotated immune genes regardless of assembly quality or method of automated annotation.

**Conclusions:**

Our results demonstrate that long reads and scaffolding technologies, alongside manual annotation, are required to accurately study the immune gene repertoire of wildlife species.

## Background

Globally, we are facing a biodiversity crisis, with 25% of known plant and animal species under threat and 1 million species facing extinction [1]. Disease is one of many drivers of global wildlife decline and extinction, with recent devastating examples such as chytridiomycosis in amphibians [2], white nose syndrome in bats [3], and devil facial tumour disease (DFTD) in Tasmanian devils (*Sarcophilus harrisii*) [4]. Habitat loss, fragmentation, and climate change lead to population decline and subsequent loss of genetic diversity, which increases susceptibility of populations to new and existing disease threats [5].

Genomics is increasingly applied in conservation [6] facilitated by a boom in genomes for wildlife species [7–10], with over 4,000 vertebrate genomes currently accessioned with the National Center for Biotechnology Information (NCBI) (March 2022). Genomics in conservation typically involves technologies, such as reduced representation sequencing, that capture single-nucleotide polymorphisms (SNPs) with a bias toward neutral regions of the genome [11, 12]. This can be used to investigate population genetic metrics such as heterozygosity, inbreeding, and relatedness to inform conservation management. This is a cost-effective approach for conservation and has been used in a range of taxa to inform conservation actions; for examples, see Tasmanian devils [13], gorillas (*Gorillia gorilla gorilla* and *Gorilla beringei graueri*) [14], helmeted honeyeaters (*Lichenostomus melanops cassidix*) [15], and bilbies (*Macrotis lagotis*) [16].

The COVID-19 pandemic is one of many examples that highlight the ever-increasing importance of understanding wildlife immunity and disease to better understand and manage disease spillover [17]. In the case of wildlife threatened by disease, conservation questions are more challenging to answer and typically involve immunogenetic diversity, which relies on accurate immune gene annotations. Immune genes in mammals can be classified into 6 major families based on their evolutionary history and function: T-cell receptors (TCRs), immunoglobulins (IGs), major histocompatibility complex (MHC), natural killer (NK) receptors, Toll-like receptors (TLRs), and cytokines. Mammals utilise 2 antigen recognition systems: TCR and IG expressed by T lymphocytes and B lymphocytes, respectively. TCR and IG are encoded in large clusters within the genome, each of which contain few constant sequences that define the receptor subtype, and multiple highly duplicated variable segments that recognise and bind antigens. The number and sequence polymorphism of IG and TCR V segments varies significantly between mammalian species [18–20]. Another major family of immune genes is the major histocompatibility complex, which contains 3 classes of genes (class I, II, and III). MHC class I and II genes encode cell-surface receptors that bind and present self- and pathogen-derived antigens to T lymphocytes, activating the adaptive immune response. Class I and II genes evolve via duplication and can be highly polymorphic; hence, gene number differs between species [21, 22]. NK cells directly kill virus-infected and cancerous cells and are an important component of innate immunity. Their activity is mediated via cell-surface receptors encoded by genes classified into 2 functionally similar but structurally dissimilar families: the leukocyte receptor complex (LRC) and natural killer complex (NKC). These families are encoded in separate clusters within the genome, and as they evolve via gene duplication, gene number varies significantly between species [23]. TLRs are membrane-spanning receptors expressed by immune and non-immune cells that bind pathogen-associated molecular patterns (PAMPs), activating the innate and adaptive immune response. Compared to other immune genes, TLR gene number and sequence are relatively conserved across mammals [24]. Lastly, cytokines are small proteins secreted by numerous cell types that direct the immune response. Cytokines can be classified into multiple families, including interferons (IFNs), tumour necrosis factors (TNFs), and interleukins (ILs), and gene content within each family varies between mammals [25].

Immune genes are some of the most polymorphic regions of the genome, owing to the need to generate diversity in response to ever-changing pathogenic pressures [26, 27]. Diversity within these gene families is generated through gene duplication, gene copy number variation, SNPs, and rapid evolution, resulting in a complex genomic organisation and high level of pseudogenization [26]. Generally, immune genes are encoded within repetitive clusters in the genome, especially highly duplicated families such as the MHC and NK receptors [28]. Given these factors, accurate assembly and annotation of genomic regions encoding immune genes can be challenging [29–31], especially in wildlife.

Automated annotation pipelines such as MAKER [32] and Fgenesh++ [33] are accurate at identifying the majority of protein-coding genes within a genome [34, 35]. However, they are less effective at characterising complex and highly variable gene families such as immune genes [36, 37], which are misassembled even in the high-quality human genome [29]. As such, manual annotation and curation of immune genes is required, which is conducted for model organism genomes accessioned with Ensembl [38]. Wildlife are not currently included in this scope, and hence immune genes are poorly annotated, or not annotated at all, in many species.

Advances in sequencing technology mean chromosome-length genomes are now achievable for a range of species [8]. Use of multiple sequencing, scaffolding, chromatin conformation, and optical mapping technologies leads to accurate assembly of complex and variable genomic regions, such as immune genes [8]. However, the high-input sample quantity and quality requirements are not always feasible for wildlife [39]. This leads to the use of lower-input short-read sequencing to generate a draft-quality genome assembled into scaffolds. However, short-read sequencing is well known to be incompetent at resolving highly repetitive and complex gene regions [40, 41]. While scaffolding technologies can improve contiguity of these assemblies, complex and variable regions often remain fragmented. The need to balance budget, sample, and genome assembly quality against accurate immune gene annotation is essential to answer questions around disease and immunity.

Over the past decade and a half, our research has focused on immunity and disease in 2 iconic marsupial species: the Tasmanian devil and koala (*Phascolarctos cinereus*). During this period, we have worked with bacterial artificial chromosome (BAC) and complementary DNA (cDNA) libraries and draft genomes of varying qualities. Our research, and that of others, has been crucial for understanding, managing, and preventing disease-induced decline [4, 42–44]. As the cost of sequencing has dropped and the appreciation of the power of genetics and genomics for population management has increased, we have increasingly been asked about the minimum levels of genome quality required to be able to effectively annotate immune genes in order to study levels of diversity in wild populations. Here we set out to answer that question.

Tasmanian devils are threatened by DFTD, a contagious cancer that has decimated over 80% of the population since it was first documented in 1996 [4]. The Tasmanian devil reference genome was sequenced using Illumina short reads in 2012 [45], generating a 3.17-Gbp genome with a scaffold N50 of 1.8 Mbp and contig N50 of 20 kbp. The MHC was not able to be annotated in the draft genome due to the high levels of fragmentation, scattered across at least 15 scaffolds. But manual annotation was possible alongside transcriptomes [46–48] and targeted sequencing of MHC-positive BAC clones [46, 49–53]. Development of MHC markers led to determination of gene copy number and nucleotide variation amongst the devil population, revealing devils have low MHC diversity, much of which is shared with DFTD [51, 54]. The low histocompatibility barriers, coupled with downregulation of tumour MHC expression, allow DFTD to transmit between individuals and evade the host immune response [52]. Recent MHC genotyping using long-read sequencing enabled the identification of full-phased MHC alleles and separation of highly similar alleles (1-bp difference), resulting in the identification of new functional MHC diversity within the devil population [55].

The koala is another iconic Australian marsupial in which disease is a major contributing factor to population decline [56]. Chlamydiosis is one of many threatening processes affecting koalas, a disease caused by infection with the intracellular bacterium *Chlamydia pecorum* [56]. A chromosome-length koala reference genome was sequenced in 2018 using Pacific Biosciences (PacBio) long reads, Illumina short reads, and BioNano optical maps [57]. This generated a 3.19-Gbp assembly with a scaffold N50 of 480 Mbp and contig N50 of 11.4 Mbp [57], a 400-fold increase in scaffold contiguity compared to the Tasmanian devil genome assembly [45]. This high-quality koala genome enabled accurate annotation of immune gene families, including the first complete reconstruction of MHC and TCR gene clusters from a genome sequence in marsupials [43, 58–60]. Preliminary genome resequencing identified that variants within IFNγ, TNFα, and MHC genes are essential for clearance of *Chlamydia* in koalas [42]. MHC genotype has also been linked to disease susceptibility and severity in different koala populations [61, 62].

In this study, our aim was to determine the impact of sequence data type, assembly quality, and automated annotation on accurate immune annotation. To achieve this, we manually annotated immune genes in the genomes of 5 marsupials and 1 monotreme. These include recent published genome assemblies of 5 marsupials—koala [57, 63, 64], woylie (*Bettongia penicillata*) [65], common wombat (*Vombatus ursinus*) [63, 64], brown antechinus (*Antechinus stuartii*) [66], and numbat (*Myrmecobius fasciatus*) [67]—and previous immune gene annotations from 1 monotreme, the platypus [41]. These 6 genomes differ in quality, including scaffold assemblies generated using only 10× Chromium linked reads (numbat, antechinus), short reads with high-throughput chromosome conformation capture (HiC) (wombat), long and short reads (woylie), and high-quality chromosome-length genomes generated using multiple data types (koala and platypus) (Table 1). We assess the accuracy of automated immune gene annotation by Fgenesh++, MAKER, and NCBI pipelines in these nonmodel species. To account for the impact of species-specific gene expansion/contraction on automated immune gene annotation, we also annotated 2 versions of the platypus genome from 2021 (GCA_004115215.4) and 2018 (GCA_002966995.1) with Fgenesh++. This study provides a guide of the impact of genome quality on immune gene annotation. Here we show that high-quality chromosome-length genomes are necessary for accurate immune annotation in the context of wildlife disease.

**Table 1:** Assembly: metrics and genome annotations for the 5 marsupial and 2 monotreme genome assemblies used in this study. The wombat and koala genome assemblies used in this study are not available on NCBI, and hence the accession ID is not provided.

	Platypus		Koala	Woylie	Wombat	Antechinus	Numbat
Genome assembly version	GCA_004115215.4 [41] 2021	GCA_002966995.1 2018	phaCin_unsw_v4.1 [57, 63, 64]	GCA_023548195.1 [65]	vu-2k [63, 64]	GCA_016696395.1 [66]	GCA_023553655.1 [67]
Data types	PacBio 10× Chromium BioNano HiC (Phase genomics & Dovetail) RNAseq (19 transcriptomes)	PacBio Illumina RNAseq (19 transcriptomes)	PacBio RS II Illumina BioNano HiC (DNAzoo) RNAseq (16 transcriptomes)	PacBio HiFi Illumina RNAseq (4 transcriptomes)	Illumina HiC (DNAzoo)	10× Chromium RNAseq (12 transcriptomes)	10× Chromium RNAseq (3 transcriptomes)
Genome size (Gbp)	2.13	1.99	3.19	3.39	3.34	3.31	3.42
GC (%)	46.23	46.64	39.05	38.64	38.89	36.20	36.3
No. scaffolds	322	4,568	1,318	1,116	633,737	30,876	112,299
No. contigs	834	5,044	1,935	3,016	685,859	106,199	219,447
Scaffold N50 (Mbp)	83.33	18.71	480.11	6.94	576.1	72.7	0.223
Contig N50 (Mbp)	15.1	7.5	11.4	1.995	0.07	0.08	0.038
Gaps (%)	0.81	0.0002	0.01	0.403	0.54	2.75	3.52
Complete mammalian BUSCOv5.3.2	83.0%	81.5%	94.1%	94.1%	89.3%	92.5%	78.7%
Genome annotations used in this study	NCBI Fgenesh++ (this study)	Fgenesh++ (this study)	Fgenesh++ (this study)	Fgenesh++ [65]	MAKER [63, 64] Fgenesh++ (this study)	Fgenesh++ [66]	Fgenesh++ [67]

## Analyses

Immune genes were annotated in the koala, woylie, wombat, antechinus, and numbat genomes and transcriptomes using similarity-based search methods such as BLAST [68] and HMMER [69] with known marsupial immune gene sequences as queries. This resulted in the manual characterisation of over 2,700 immune genes amongst the 5 species, from 6 immune gene families or groups: TLRs, TCRs, IGs, MHC, NK cell receptors, and cytokines (Table 2). Platypus immune gene families have previously been annotated [41, 70–81], some of which had already been mapped within the 2021 genome assembly (MHC and TCR) [41], and the remainder were mapped in both the 2018 and 2021 assemblies in this study. Genomic coordinates of all immune genes annotated in this study are available in Additional File 1. A comprehensive summary of results for each immune gene family is available in Additional File 2.

**Table 2:** Number of annotated immune genes in each of the 5 marsupials and 1 monotreme in this study. The percentage overlap of genomic coordinates between manual and automated annotations of immune genes is also provided for each family and species.

	Platypus	Koala	Woylie	Wombat	Antechinus	Numbat
Cytokines	49 (48%, 8%)	82 (20%, 22%)	77 (19%, 38%)	76 (33%, 44%)	68 (17%, 21%)	67 (21%, 30%)
TLR	10 (90%, 10%)	10 (0%, 20%)	10 (6%, 37%)	10 (100%, 0%)	10 (10%, 20%)	10 (10%, 20%)
MHC I	6 (14%, 0%)	19 (21%, 21%)	17 (5%, 5%)	5 (60%, 0%)	7 (22%, 10%)	3 (22%, 11%)
MHC II	5 (25%, 25%)	16 (6%, 25%)	23 (12%, 16%)	7 (42%, 0%)	14 (33%, 6%)	6 (33%, 6%)
MHC III	58 (88%, 4%)	39 (11%, 7%)	37 (23%, 2%)	38 (65%, 7%)	36 (11%, 32%)	35 (12%, 12%)
Ext. MHC & framework genes	20 (100%, 0%)	27 (13%, 10%)	31 (32%, 8%)	34 (41%, 11%)	31 (21, 10%)	33 (11%, 42%)
NKC	122 (31%, 63%)	17 (27%, 11%)	17 (27%, 11%)	11 (9%, 36%)	11 (18%, 27%)	17 (33%, 5%)
LRC	4 (0%, 0%)	25 (3%, 18%)	60 (3%, 63%)	33 (28%, 54%)	49 (5%, 38%)	41 (5%, 38%)
Extended LRC	11 (36%, 0%)	6 (0%, 12%)	22 (0%, 60%)	9 (0%, 100%)	15 (37%, 18%)	11 (56%, 31%)
IG constant	14 (5%, 50%)	15 (0%, 66%)	20 (4%, 22%)	10 (16%, 66%)	7 (28%, 14%)	6 (0%, 33%)
IG variable	118 (0.5%, 80%)	289 (0%, 58%)	226 (0%, 58%)	98 (0.9%, 81%)	145 (0.6%, 43%)	121 (0%, 34%)
TCR constant	19 (0%, 88%)	10 (0%, 45%)	12 (0%, 29%)	10 (0%, 81%)	11 (0%, 36%)	9 (0%, 22%)
TCR variable	252 (0%, 78%)	103 (0%, 58%)	122 (0%, 76%)	92 (2%, 86%)	126 (0%, 59%)	104 (0%, 71%)
Total	678 (21%, 57%)	658 (5%, 41%)	674 (6%, 48%)	440 (21%, 57%)	531 (8%, 37%)	463 (9%, 38%)

Includes complete and partial gene sequences. A more detailed comparison of immune genes annotated in this study, with those identified in other marsupials and humans, is available in Supplementary Table 2 within Additional File 2. The first percentage represents ≥90% overlap and the second represents ≤10% overlap between automated and manual annotations of the respective immune genes for each species. Values for the NCBI annotation of the 2021 platypus genome are presented here.

Overall, the immune gene repertoire of the koala, woylie, wombat, antechinus, and numbat was similar to other marsupials [58, 82], with marsupial-specific genes and eutherian orthologs identified. Relatively conserved immune genes such as TLRs and constant regions of TCR and IG, as well as polymorphic genes such as MHC and NK receptors, were identified in all 5 species. Numerous koala immune gene sequences have been characterised previously due to their involvement in chlamydiosis and koala retrovirus, which threaten populations [56]. These include MHC [57, 83–85], IG [58], TCR [57], NK receptors [59], and selected cytokines [58, 86–89] (Supplementary Table S2 in Additional File 2). We mapped the location of these genes within the current version of the genome and identified additional new sequences within the LRC, IG, and cytokine families (Table 2, Supplementary Table S2 in Additional File 2). Immune genes unique to the marsupial lineage were also characterised in the 5 species studied here. These included MHC class II genes DA, DB and DC, TLR1/6, and TCRμ. Large marsupial-specific gene expansions within the LRC NK receptors were characterised in all 5 species, as well as reduced gene content within the NKC cluster of NK receptors. Consistent with other marsupials investigated to date, Igδ was not found in any of the 5 assemblies [90]. A detailed outline of immune genes annotated in this study compared to those of other marsupials and humans is provided in Supplementary Table S2 within Additional File 2.

### Automated versus manual immune gene annotation

We assessed how well our manual immune gene annotation aligned with automated annotations by Fgenesh++ (2018 platypus, woylie, koala, antechinus, numbat and wombat), MAKER (wombat), and the NCBI pipeline (2021 platypus). Inclusion of the 2021 platypus NCBI and wombat MAKER annotations ensures that any differences in automated and manual immune gene annotation were not due to deficiencies within the Fgenesh++ annotation pipeline, as the woylie, antechinus, and numbat genomes were all annotated with Fgenesh++ using the same parameters.

**Figure 1:**
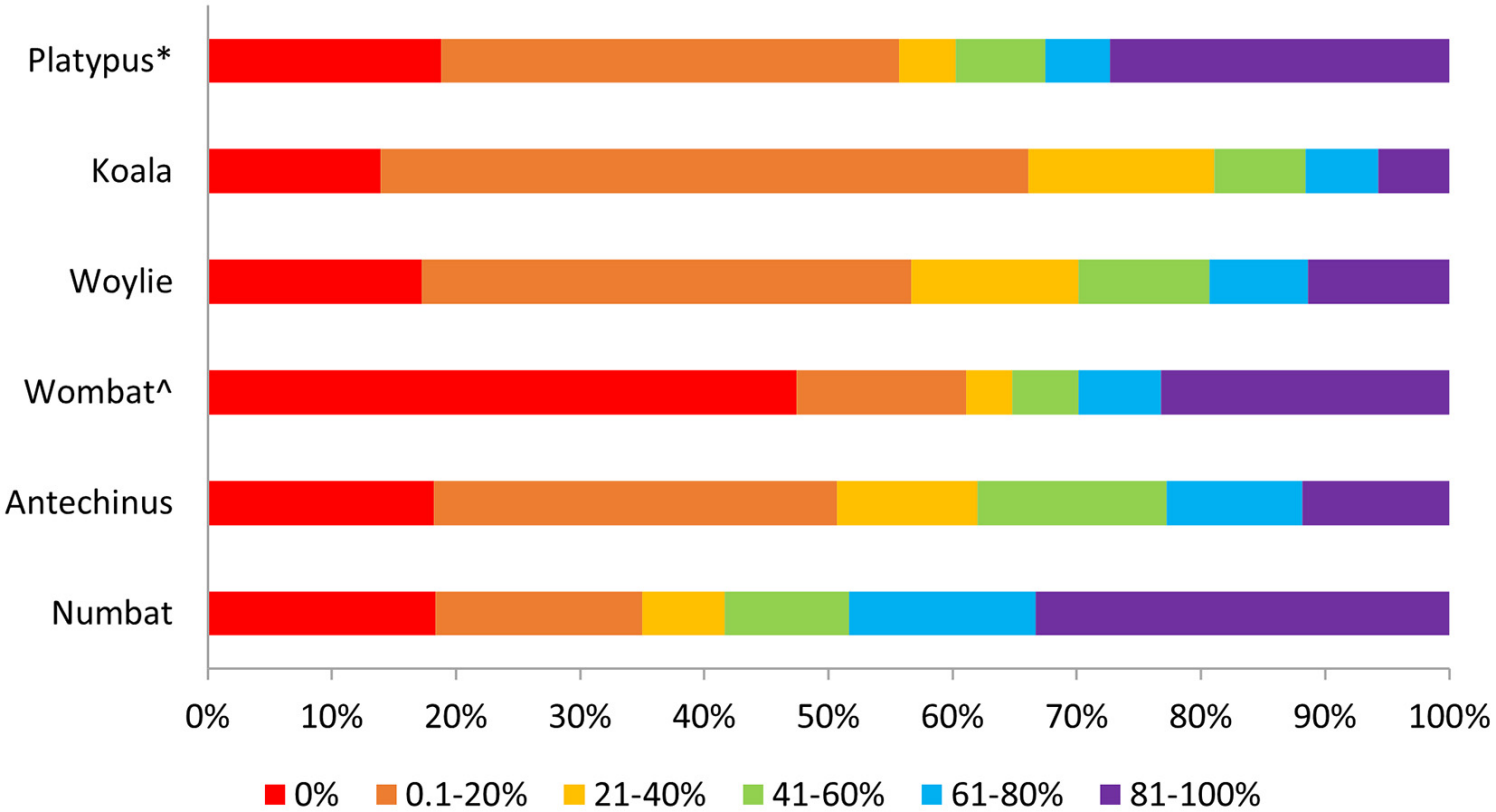
Percentage overlap of genomic coordinates between manual and automated annotations of immune genes in 6 genomes. *Denotes automated annotation by NCBI and ^denotes automated annotation by MAKER. The remaining genomes were annotated using Fgenesh++. Colours indicate proportion of immune genes with 0% to 100% overlap between manual and automated annotations, with 0 indicating manually annotated genes with no overlap of genomic coordinates with the automated annotation.

Automated annotation pipelines failed to characterise the complete immune repertoire of the platypus or any of the 5 marsupial species (Fig. 1). Only 21.27%, 5.66%, 6.89%, 21.82%, 8.68%, and 9.07% of immune genes were correctly annotated by the automated pipeline in the 2021 platypus, koala, woylie, wombat, antechinus, and numbat, respectively, defined as ≥90% overlap in genomic coordinates of immune genes between our manual annotations and the automated annotations (Fig. 1). Interestingly, more immune genes were correctly annotated by the automated software in the low-quality wombat, antechinus, and numbat genomes than the high-quality platypus, koala, and woylie genomes. This inverse relationship between genome quality and proportion of correctly annotated immune genes is likely related to the characterisation of additional divergent and polymorphic genes such as MHC class I and II in woylie, koala, and platypus, which could not be identified by automated or manual annotation in the wombat, antechinus, and numbat due to genome fragmentation (Table 1). All genomes analysed in this study displayed a high proportion of immune genes that were very poorly annotated by automated pipelines (≤10% overlap between immune gene coordinates from manual versus automated annotation): 57.01%, 41.78%, 48.96%, 57.01%, 37.05%, and 38.22% for 2021 platypus, koala, woylie, wombat, antechinus, and numbat, respectively (Fig. 1).

A breakdown of this analysis by immune family revealed that marsupial- and monotreme-specific immune genes that are not orthologous to those in eutherians were generally poorly annotated, regardless of automated pipeline or genome quality (Supplementary Fig. 1). This was particularly the case for TCR and IG gene families, with up to 88% of genes in these families incorrectly annotated by automated pipelines (≤10% overlap) amongst the 6 species (Table 2). This is likely due to highly duplicated variable gene segments that do not encode conventional exon–intron splice sites, which may hinder annotation with automated pipelines. Poor gene annotations of TCR and IG families were somewhat recovered at the exon level, as some TCR and IG variable gene segments were annotated as exons by automated pipelines. Correct annotation (≥90% overlap) of the TCR family increased from 0–2% at the gene level to 2–15% at the exon level amongst the 6 genomes (Supplementary Fig. 2). This improvement was even greater for the IG family, with an increase from 0–2% correct annotation at the gene level to 15–43% at the exon level amongst the 6 genomes (Supplementary Fig. 2). Despite this, up to 67% of TCR and IG variable segments were still not annotated at the exon level (0% overlap) amongst the 6 genomes, highlighting the difficulty in automated annotation of these regions. Similarly, marsupial-specific gene expansions within the LRC and monotreme-specific gene expansions within the NKC family of NK receptors were also poorly annotated by automated pipelines (Supplementary Fig. 1). As with TCR and IG families, correct annotation increased from the gene (0–28% marsupial LRC, 31% platypus NKC) to exon level (6–65% marsupial LRC, 79% platypus NKC) (Table 2, Supplementary Fig. 2), likely due to the presence of variable numbers of duplicated immunoglobulin superfamily (IGSF) domains and C-type lectin (CLEC) domains within each LRC and NKC gene, respectively.

This pattern of poor immune gene annotation was not an artefact of inherent differences between automated annotation pipelines amongst the 6 genomes (NCBI, MAKER, and Fgenesh++) or genome quality, as similar patterns were observed for Fgenesh++ annotations of the 2021 platypus and wombat genome generated as part of this study (Supplementary Fig. 3, Supplementary Fig. 4). Generally, the Fgenesh++ annotation resulted in fewer correctly annotated immune genes (≥90% overlap) compared to NCBI (2021 platypus) or MAKER (wombat) (Supplementary Fig. 3), although the proportion of missing immune genes (0% overlap) was higher in the NCBI (2021 platypus) and MAKER (wombat) annotation than the Fgenesh++ annotation of both species’ genomes. As with NCBI and MAKER, Fgenesh++ poorly annotated TCR and IG families at the gene level (Supplementary Fig. 4) in the high-quality platypus and low-quality wombat. Correct annotations were somewhat recovered at the exon level in both genomes (Supplementary Fig. 5), although the number of missing TCR and IG exons in the Fgenesh++ annotation was almost half that of NCBI and MAKER in platypus and wombat, respectively.

### Relationship between genome quality and manual immune gene annotation

**Figure 2:**
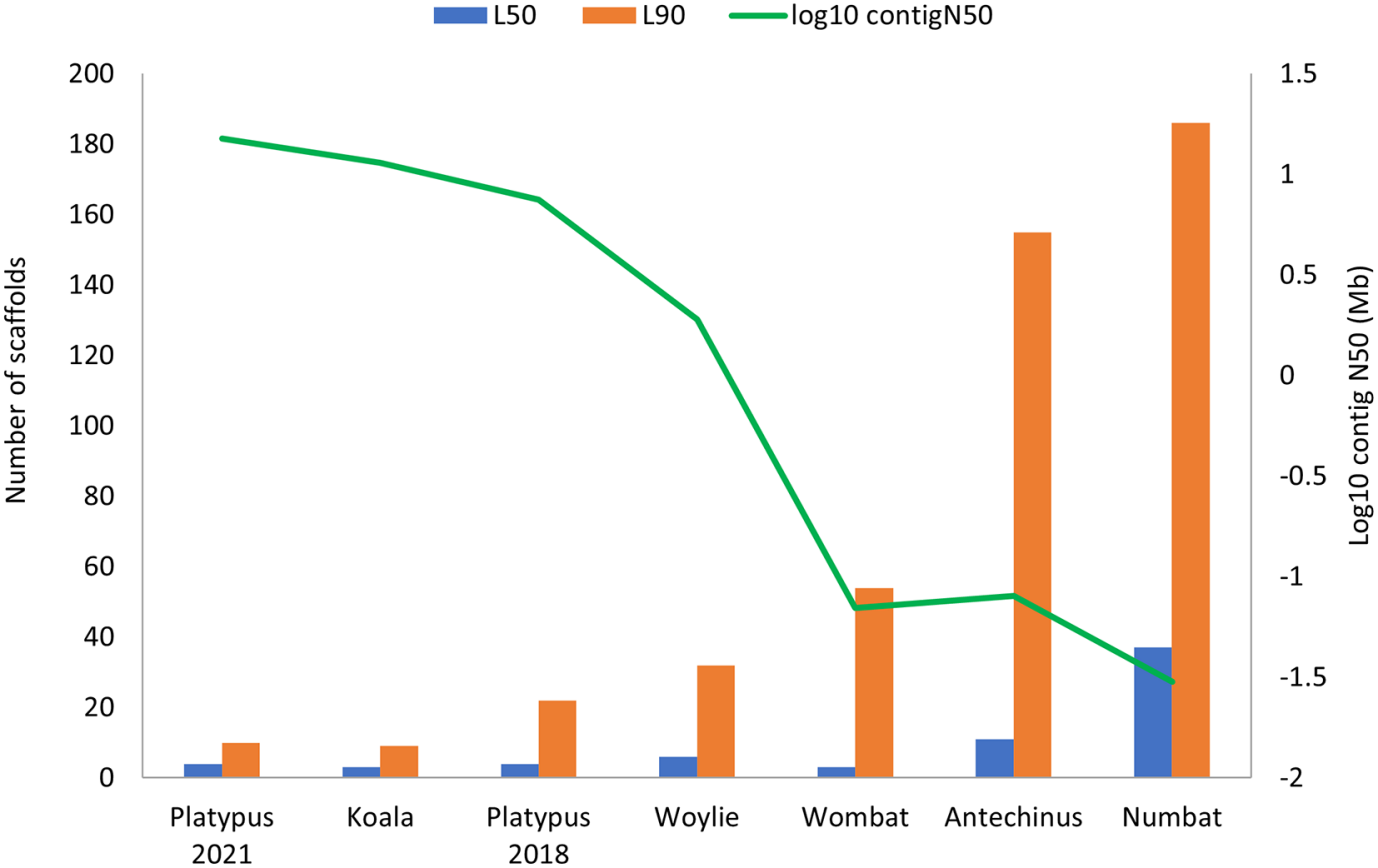
L50 and L90 immune gene metric for 7 genomes from 6 species, compared to log_10_ contig N50.

**Figure 3:**
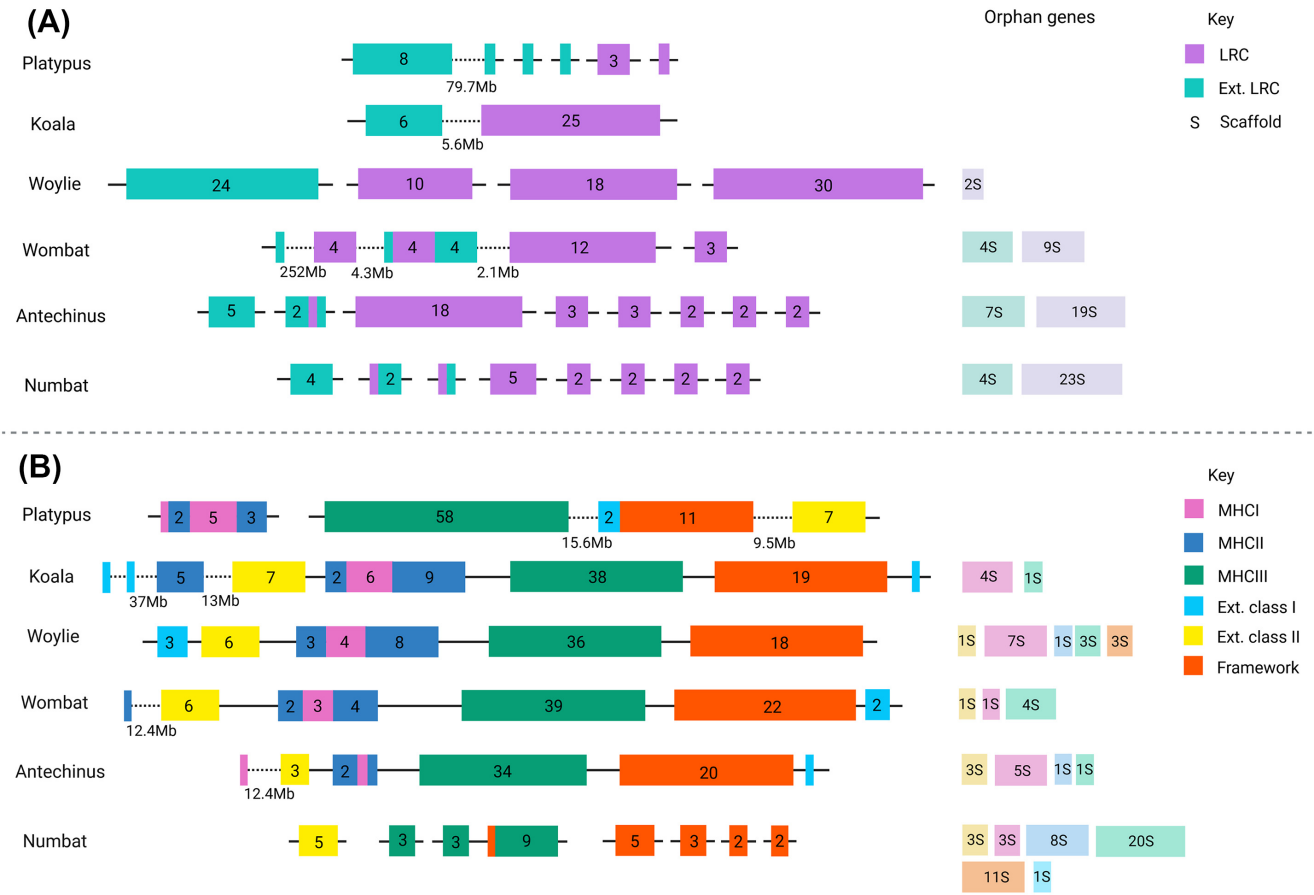
Genomic organisation and gene content of the LRC (A) and MHC region (B) in 6 genomes. The number of genes within each cluster is given, as well as scaffold counts of orphan genes (genes on single scaffolds). In A, LRC genes are purple, and extended LRC genes are teal. In B, MHC class I genes are red, class II blue, class III green, extended class I pink, extended class II yellow, and framework genes orange. Large distances between genes are given below the scaffold; otherwise, the distance between genes and/or clusters was within the expected range for each family. Figure created with BioRender.com.

Manual annotation of immune genes across the koala, woylie, wombat, antechinus, and numbat genomes, as well as mapping of previous annotations to both the 2018 and 2021 versions of the platypus genome, highlighted a clear relationship between immune gene fragmentation and genome quality (Fig. 2). Overall, the high-quality koala, 2021 platypus, and woylie genomes all contained complete immune gene family clusters, which were highly fragmented in the lower-quality wombat, antechinus, and numbat genomes. Fragmentation was particularly evident within families, which contain genes that do not share orthology to those in eutherians, such as LRC NK receptors and TCRμ, and highly duplicated families such as MHC (Fig. 3).

To rule out species-specific differences in our direct assessment of assembly quality on immune gene annotation, we annotated a previous version of the platypus genome from 2018 (GCA_002966995.1) with Fgenesh++ to enable comparison with our Fgenesh++ annotation of the 2021 platypus genome (GCA_004115215.4) also generated as part of this study. Compared to the 2021 assembly, the 2018 platypus assembly was more fragmented given the 6-fold increase in the number of contigs, 14-fold increase in the number of scaffolds, and associated 2-fold decrease in contig N50 and 4-fold decrease in scaffold N50 between the two assemblies. Despite these metrics, the 2018 platypus assembly is still highly contiguous as it was generated using long-read data.

To investigate the relationship between immune gene fragmentation and genome quality further, we calculated the number of scaffolds that encoded 50% (L50) and 90% (L90) of manually annotated immune genes in each of the 7 genomes from 6 species (Fig. 2).

The 2021 platypus, koala, and woylie had an L90 of 10, 9, and 36, respectively, which suggests immune gene families were highly contiguous within all 3 genomes (Fig. 2). Complete coding sequences were identified for 98% and 95% of immune genes in koala and woylie, respectively. In addition, 90% of annotated immune genes were located on scaffolds greater than 33.3 Mbp, 75 Mbp, and 1 Mbp in the 2021 platypus, koala, and woylie, respectively. Complex multigene immune families such as MHC, NK receptors, and TCR were highly intact in all 3 species. The koala and woylie MHC regions were both primarily located on a single scaffold (Fig. 3). Class I and II genes were interspersed and flanked by class III, framework, and extended class I and II gene clusters, which reflected the MHC organisation of other marsupials (Fig. 3) [18, 57]. Unlike marsupials, the platypus MHC is encoded within a pseudoautosomal region of 2 sex chromosomes. MHC class I and II genes were interspersed in a single cluster on chromosome X3 and class III, extended class I and II, and framework genes located in a single cluster on chromosome X5 (Fig. 3) in the 2021 assembly [41]. Large gene expansions within the LRC NK receptors were encoded on a single scaffold in koala and 6 scaffolds in woylie (Fig. 3). The number and type of monotreme NK receptor genes differ from marsupials, as they have a large expansion within the NKC gene cluster and reduction within the LRC gene cluster [72]. More than 80% of platypus NKC genes were located in a single cluster on chromosome 17, with LRC genes located on 5 different chromosomes in the 2021 assembly [72]. Fragmentation of the LRC cluster is not a factor of genome quality but reflects the evolutionary history of this immune family [72]. The 4 TCR loci (α/δ, β, γ, and μ) were encoded in single clusters on 3 chromosomes in the platypus 2021 assembly and single scaffolds in koala. The TCR loci were fragmented across up to 3 scaffolds in woylie. This includes genes known to flank these loci in other marsupials, which enabled resolution of TCR locus organisation in these species, and confirmed gene synteny across marsupials, human, and mouse as identified previously [18].

Fragmentation of immune genes in the wombat genome differed between immune families, with an L90 of 56 (Fig. 2). Twenty-two percent of scaffolds encoding immune genes were shorter than 100 kbp, and partial coding sequences were identified for 7% of annotated immune genes. The MHC region was relatively contiguous in the wombat, with 92% of genes encoded on a single scaffold (Fig. 3), although a number of MHC genes were encoded as orphan genes to the main MHC cluster, indicating this family is misassembled in the wombat genome. In addition, some MHC genes could not be identified in the wombat genome, while only single copies could be identified for others that are known to be duplicated in all other marsupials studied to date (Additional File 2). While this reduced MHC gene content in the wombat may reflect the true MHC gene repertoire of this species, it is likely MHC genes could not be annotated due to assembly error. The LRC cluster was highly fragmented across 16 scaffolds (Fig. 3), of which more than 80% encoded a single gene and were less than 10 kbp in length. Extended LRC and LRC genes were interspersed, likely due to misassembly of the region as these genes should be located in separate clusters as observed in koala and woylie (Fig. 3). TCRα, β, and γ loci were encoded on individual scaffolds, but TCRμ was fragmented across 10 scaffolds, with 34% of genes located on individual scaffolds of less than 15 kbp. While the TCRβ locus was encoded in a single cluster in the wombat, half of the locus was in the reverse orientation. This organisation is unusual amongst mammalian TCR and is likely a result of the HiC scaffolding error and not a true inversion.

Immune gene families were highly fragmented in the antechinus and numbat genomes, with an L90 of 156 and 218, respectively (Fig. 2). Twenty-nine percent and 43% of immune genes were located on scaffolds less than 100 kbp, and partial coding sequences were identified for 5.7% and 10.8% of immune genes, in antechinus and numbat, respectively. Complex multigene families such as MHC, NK, receptors and TCR were highly fragmented, with individual genes or exons located on short scaffolds. While 86% of MHC genes were located on a single scaffold in antechinus (Fig. 3), genome fragmentation prevented the identification of additional MHC genes, and hence the true MHC gene content could not be determined. The numbat MHC region was highly fragmented across 52 scaffolds, 63% of which were less than 100 kbp in length (Fig. 3). Large gene expansions of LRC NK receptors were fragmented across 34 scaffolds in antechinus and numbat, of which 67% (antechinus) and 35% (numbat) were less than 10 kb, and 76% of scaffolds encoded individual LRC genes in both species (Fig. 3). Similar to wombat, extended LRC and LRC genes were interspersed, likely a misassembly, as these genes should be encoded within separate clusters as observed in koala and woylie. All 4 TCR loci were fragmented in numbat, and all except TCRα in antechinus, with individual loci encoded across up to 6 scaffolds in numbat and 19 in antechinus. Low contiguity within genomic regions encoding immune gene families in the antechinus and numbat limited investigation of genomic organisation, synteny, and evolution in these species.

This relationship between genome quality and immune gene fragmentation is not an artefact of species-specific differences in immune gene repertoires. Comparison of manual immune gene annotations in the 2021 and 2018 platypus genome assemblies revealed similar patterns of immune gene fragmentation in the lower-quality 2018 assembly (Supplementary Figs. 6 and 7). The 2018 platypus assembly had an L90 metric of 22, indicating immune gene clusters were intact within this genome but not to the extent of the 2021 assembly (L90 of 10) (Fig. 2). In the 2018 assembly, only 28% of NKC genes were encoded on a single scaffold (compared to 80% in the 2021 assembly), the MHC was encoded across 6 scaffolds (compared to 2 in the 2021 assembly), and only 2 of the 4 TCR clusters were intact (all were intact in the 2021 assembly). Automated annotation of both assemblies with Fgenesh++ and comparison with our manual immune gene annotations yielded the same result as presented for the 5 marsupial genomes: immune genes are poorly characterised by automated pipelines regardless of genome quality. In the 2021 and 2018 assemblies, a similar proportion of immune genes were correctly annotated (10% and 9%, respectively) and not annotated (10% and 15%, respectively) by Fgenesh++ (Supplementary Fig. 6). As observed in the 5 marsupial genomes, TCR and IG were the most poorly annotated families by Fgenesh++ in both platypus assemblies (Supplementary Fig. 7).

## Discussion

By manually annotating immune genes in 5 marsupial genomes and 2 versions of the platypus genome, all varying qualities, we have confirmed that genome quality is directly linked to our ability to annotate complex immune gene families. Without long reads and scaffolding technologies, immune genes are scattered across many individual scaffolds, and gene family organisation and evolution cannot be elucidated. We conclude that long-read data, with or without HiC technology, to generate a high-quality genome assembly with a contig N50 of at least 1 Mbp are required to investigate immunity and disease in wildlife. However, a kitchen sink approach to genome sequencing and assembly will enable complete reconstruction of complex and duplicated families such as MHC, TCR, and LRC NK receptors as in the platypus 2021 and koala genomes.

The immune gene repertoire of the koala, woylie, wombat, antechinus, and numbat was similar to other marsupials such as Tasmanian devil [46, 49, 53], tammar wallaby (*Macropus eugenii*) [74, 91–94], and grey short-tailed opossum (*Monodelphis domestica*) [82]. The platypus immune gene repertoire has been characterised previously [41], and we identified their location within both the 2021 and 2018 genome assemblies. Fewer MHC genes were identified in the wombat, antechinus, and numbat, compared to the platypus, koala, and woylie (Table 2, Supplementary Table S2 in Additional File 2). This is likely due to poor read assembly within this highly variable and duplicated region of the genome, rather than a true reduction in MHC gene content within these 3 species, but further investigation into the MHC gene repertoire of additional marsupial species is required. The assembly of a complete MHC cluster in the platypus, koala, and woylie is due to the ability of long reads to span duplicated and variable sequences, which enables assembly algorithms to accurately reconstruct this complex region of the genome.

### Automated annotation poorly characterises immune genes in nonmodel species

Despite mammalian BUSCO scores of up to 94.1% amongst the 7 genomes in this study, indicating that the genomes were “functionally complete,” on average, 59% of immune genes were not accurately annotated (≤80% overlap) and 21% of genes were not annotated (0% overlap) by the automated software Fgenesh++ and MAKER, or the NCBI pipeline, compared to our manual annotations (Fig. 3). Aside from TCR and IG, the majority of immune genes incorrectly annotated or missing from the automated annotations were divergent genes not orthologous to those in eutherian mammals, such as MHC, marsupial-specific gene expansions within the LRC, and monotreme-specific gene expansions within the NKC. Given their divergence, these genes often have low or no BLAST homology to nucleotide or protein databases. Gene models generated by automated annotation software are hypotheses based on supporting evidence such as RNA sequencing (RNA-seq) data and homology to nucleotide and protein databases. While immune transcripts were identified in the transcriptomes from these species, RNA-seq data only supported gene models for a low proportion of MHC, LRC, and NKC genes. RNA-seq data only supported 8–16% of LRC gene predictions and 16–37% of MHC gene predictions amongst the 4 marsupial genome annotations, which used RNA-seq data as gene model evidence (koala, woylie, antechinus, and numbat). Similarly, around 60% of NKC genes within the platypus genomes were supported by RNA-seq data. Overall, RNA-seq data did not provide enough evidence to support gene models for ∼20% of immune genes within the genome. Some immune genes may not have been expressed in the tissue sequenced, were expressed at low levels, or were fragmented. For human and mouse, comprehensive and curated gene sets such as GENCODE and RefSeq are available to guide gene model predictions, comprising data from more than 10,000 RNA experiments and decades of dedicated work in this field [95, 96]. Given time, budget, and sample constraints for wildlife, these curated gene sets are not available, and hence RNA-seq evidence is incomplete, resulting in deficient gene models by automated annotation software.

It is not surprising that TCR and IG V segments were poorly or not annotated by all automated pipelines used to annotate the genomes in this study. These genes are notoriously difficult to characterise and are manually annotated in the human and mouse genome on Ensembl using the International Immunogenetics Information System (IMGT) database [38, 97]. Alignment of mature IG and TCR sequences from RNAseq data to the genome results in poor automated annotation, as V segments utilize different sequence signal splice sites to introns, which are not recognized by the open reading frame prediction algorithms. Indeed, RNAseq evidence only supported 7% to 18% of TCR V segment and 0% to 6.9%% of IG V segment gene predictions by automated pipelines amongst the four marsupial and platypus genomes. V sequences from three marsupials and two monotremes are available in IMGT, however as non-model species, they are not included in the scope for manual annotation by Ensembl or NCBI, so these important functional features are not annotated.

Our results highlight the importance of manual annotation and curation of complex and variable immune genes, and they caution reliance on BUSCO metrics to assess functional completeness of a genome. If this pattern is observed more widely across nonmodel species and other complex gene families, functionally important genes may not be accurately represented in genome annotations, which will flow on to downstream applications [36, 98]. While automated annotation is required to keep pace with the rapid sequencing of genome assemblies, manual gene characterisation is still the gold standard for genome annotation [95] and is conducted for the human, mouse, zebrafish, and rat genomes on Ensembl [99]. For nonmodel species, manual annotation is conducted by individual research groups following genome assembly accession with NCBI or Ensembl, who conduct in-house automated annotation for some but not all species [100, 101]. These highly valuable manual gene annotations are not incorporated into the Ensembl annotation release but are often listed in the supplementary materials of multiple individual publications. NCBI does have some capacity to incorporate manual changes to existing annotation records [102]. Changes to multiple annotations, such as adding new genes, as is the case in this study, require the genome to be reannotated, which is not feasible for all research groups. Given NCBI and Ensembl annotations are widely used by the scientific community, these institutions should consider incorporating manual gene annotations into the annotation record or provide scope for permanently storing these valuable data alongside the respective assembly.

### Genome quality correlates with immune gene fragmentation

**Figure 4:**
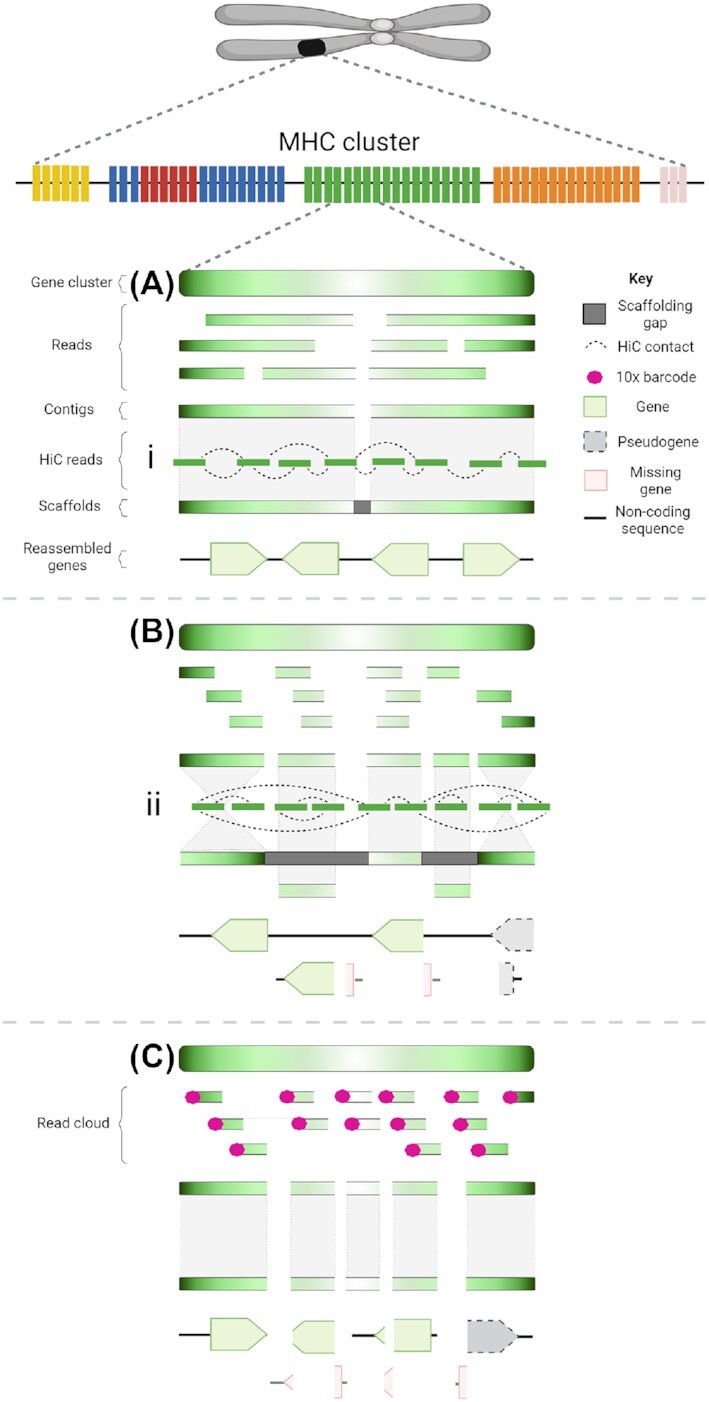
Impact of different sequencing technologies on the assembly of immune gene clusters such as the MHC. The impact of long-read (A—platypus, koala and woylie), short-read (B—wombat), and 10× Chromium linked read (C—antechinus and numbat) sequencing technologies, alone or in combination with HiC scaffolding (i—koala & platypus; ii—wombat), on the assembly of complex and repetitive immune gene clusters such as the MHC. Colour gradient represents gene orientation. (A) Long-read sequencing generates reads that span complex and repetitive sequences, resulting in long contigs and scaffolds that contain multiple immune genes with complete coding sequences. (B) Short-read sequencing generated reads that are unable to span immune genes; hence, reads are assembled into multiple short contigs that end when the algorithm is unable to assemble a repetitive and complex immune gene sequence. (C) In linked-read sequencing, individual DNA molecules are partitioned into gel beads and identical barcodes attached, then sequenced using short-read technology, resulting in read clouds [103]. As no individual read within the cloud spans the entire length of the DNA molecule, the algorithm is unable to assemble repetitive and complex sequences, resulting in multiple short contigs similar to a short-read assembly. Short contigs in B and C result in fragmentation of immune genes, leading to false pseudogenization and “missing” genes. (i) HiC sequencing provides contact information for DNA sequences located in close proximity within the nucleus, as frequency decreases with increasing linear distance within the genome assembly [104]. This contact information can be used to cluster, order, and orient contigs into chromosome-size scaffolds [105]. Long contigs scaffolded with HiC result in near-complete reconstruction of immune gene clusters. (ii) Short contigs scaffolded with HiC generate what appears to be long scaffolds, but complex immune gene clusters are incomplete. As multiple HiC contacts can span the length of the contig, the correct contig orientation is not apparent, leading to inversions and misplaced contigs during scaffolding. This leads to incorrect orientation of genes, which can cause pseudogenization and/or gene fragmentation. Manual immune gene annotation reveals that the true gene complement of the immune cluster is not contained within the scaffolded sequence. Figure created with BioRender.com.

As expected, we found that genome quality directly correlates with likelihood that an immune gene family was assembled and annotated correctly. Immune genes fragment as genome quality declines (Figs. 2 and 3). This highlights the importance of long reads and HiC scaffolding to reassemble complex gene families (platypus, koala, woylie), which are poorly assembled in short-read and linked-read assemblies (wombat, antechinus, numbat). Fig. 4 provides a graphical representation of the impact of different sequencing technologies on the assembly and fragmentation of immune gene clusters. When the average read or contig length is shorter than the gene length, the assembly algorithm is unable to reconstruct genes, which are fragmented across multiple short contigs [98]. The average immune gene in this study was ∼10 kbp in length. Long reads greater than 10 kbp in both platypus, koala, and woylie genomes were able to span these genes, whereas the ∼150-bp short reads in the wombat, antechinus, and numbat genomes were insufficient to reassemble the entire gene, resulting in gene fragments on short scaffolds. Gene families with copy number variation such as MHC and NK receptors are notoriously difficult to assemble and annotate [26, 29], so it is not surprising these gene families were highly fragmented in the antechinus and numbat genomes. Gene copies within these families can contain almost identical domains, may be pseudogenes, and are encoded in clusters within the genome [36]. For example, koala NK LRC genes share up to 96% amino acid sequence identity and are encoded within a single cluster. For these reasons, assembly and annotation of MHC and NK receptors have been used to illustrate improvements in assembly quality. For example, MHC class I genes were located on a single contig in a recent release of the human genome [29], but the highly repetitive MHC class II locus remains unresolved [29].

HiC scaffolding of contigs derived from platypus and koala long reads resulted in complete and accurate reassembly of immune gene clusters in both genomes (Fig. 4A). Conversely, HiC scaffolding of contigs from wombat short reads resulted in immune gene fragmentation (Fig. 4B), reflected in the high immune gene L90 for the wombat genome (Fig. 2). Both the koala and wombat genomes were scaffolded with DNAzoo HiC data using the same 3D-DNA pipeline [63, 64, 106]. This result underscores the importance of assessing annotations when determining genome quality, as the wombat genome is classified as chromosome length yet is highly fragmented within functionally important genomic regions. Input genome assembly contiguity is known to influence HiC scaffolding ordering and orientation errors [107], despite claims that HiC scaffolding with 3D-DNA generates chromosome-length scaffolds from US$1,000 short-read contigs [63]. Problems with HiC scaffolding within repetitive and duplicated regions are well documented [31, 107, 108], which is exacerbated by short contigs [107]. Modelling of human genome scaffolding performance using 3D-DNA revealed scaffold chimeras, ordering, and orientation errors increased as contig length decreased [107]. While the koala and platypus genomes used as input to HiC scaffolding benefited from polishing with short-read data and optical mapping [57], HiC scaffolding is insufficient to recover the majority of immune clusters from a fragmented genome.

The 3D-DNA pipeline orientates contigs within scaffolds by maximizing contact frequency between contig ends [64]. Short contigs, such as those from the wombat, would have multiple contacts that span the length of the contig. This means both true and false contig orientations would have a similar frequency, resulting in errors such as the partial inversion of the TCRβ locus, which is likely false (Additional File 2). At a gene level, these errors lead to the misplacement of genes on short scaffolds outside the main immune cluster and false pseudogenisation (Fig. 4B). Long contigs, such as those from the koala, would have fewer contacts that span the length of the contig, and hence the true orientation of the contig would be clear from the higher contact frequency at the correct joining end. The combination of long contigs, which span repetitive and highly heterozygous regions with HiC scaffolding, maximises contiguity within immune gene clusters (Fig. 4A).

The 10× Chromium linked-read sequencing was insufficient to accurately reassemble immune gene clusters in our study (Fig. 4C). While this technology is no longer available for genome sequencing, acknowledging the limitations of this technology for immune gene annotation remains valid in order to make use of existing 10× genomes. Complete marsupial immune gene clusters can span hundreds of kilobases to megabases, as shown by annotation of the complete MHC, NK receptor, and TCR regions in the koala (Additional File 2). DNA molecules input to 10× library preparation were on average 74 kbp and 23 kbp in antechinus and numbat, respectively. This molecule size spanned only smaller immune clusters in the antechinus, such as the 70-kbp TRG locus, but was insufficient to span any cluster in the numbat. Interestingly, the antechinus MHC cluster appears to be intact (Fig. 3), but manual annotation revealed multiple genes were “missing” within the scaffold and instead were located on individual short scaffolds. Regardless of input DNA molecule length, 10× libraries are still subject to the limitations of short-read sequencing regarding assembly of complex sequences. Antechinus and numbat 10× libraries were sequenced as short ∼150-bp reads; hence, while reads can be assigned back to the corresponding input DNA molecule, no single read spans the molecule length. Gaps between the reads make *de novo* assembly of repetitive and complex immune sequences difficult, often resulting in termination of contig extension and gene fragments scattered across short scaffolds [109–111]. These gene fragments can be misinterpreted as pseudogenes owing to loss of up/downstream coding regions (Fig. 4C). For example, antechinus and numbat NK LRC genes share up to 97% and 98% amino acid sequence identity amongst the genes identified in each species, respectively. The LRC should be encoded within a single cluster, as in the koala genome (Fig. 3). Instead, the antechinus and numbat LRC clusters are fragmented across 33 and 34 scaffolds, respectively.

As the global biodiversity crisis deepens, the need to sequence eukaryotic life while it remains is imperative [1, 7, 8]. High-quality genomes, using a combination of long-read and HiC, have recently been generated for a number of wildlife species [8], which have been used to answer questions involving chromosome evolution [112], comparative genomics [113], and runs of homozygosity [114], amongst others. Our results show that high-quality genomes are also necessary to study immune genes in wildlife.

Draft-quality *de novo* genomes—in this study, the antechinus and numbat (linked reads)—have limited capacity for usefully informing immunogenetics studies as only partial sequences will be identified for most immune genes. A scaffold-quality genome—in this study, the woylie and 2018 platypus assembly (long reads) or wombat (short reads with HiC)—would be suitable for immune marker development targeting most immune gene families and studying TCR and IG diversity. Long reads will provide contiguity within duplicated MHC and NK families, which should reassemble into complete clusters. HiC data may resolve some immune gene clusters from a short-read assembly but may introduce errors as discussed earlier. Finally, the kitchen sink approach—in this study, the 2021 platypus and koala genomes (multiple data types)—will accurately assemble immune gene clusters, which is essential for investigating genomic organisation, synteny, and evolution. In the context of wildlife disease, both sample availability and research dollars will dictate the type of data able to be generated for genome assembly; from this study, we recommend a minimum of long-read sequencing such as PacBio HiFi to allow for complete annotation of immune gene regions.

## Potential implications

The biodiversity crisis and increasing impact of wildlife disease on animal and human health provides impetus for studying immune genes in wildlife. Genomes are now available for many wildlife species, but utility of these assemblies for annotating complex immune gene families is unknown. We have provided an assessment of complex immune gene annotation across genomes of varying quality, using immune genes in 5 marsupials and 1 monotreme as an example. Genome quality directly influenced the reassembly of immune gene clusters and ability to investigate evolution, organisation, and true gene content of the immune repertoire. A high-quality genome generated from long reads, with or without HiC, accurately assembles immune gene clusters. However, draft-quality genomes generated from short reads with HiC, or the now obsolete 10× Chromium linked reads, were unable to achieve this. Aside from genome quality, manual annotation of immune genes is required to cover the shortfall in deficient gene models used by automated annotation software. Our results highlight the limitations of different sequencing technologies and established workflows for genome annotation and quality assessment, when applied to nonmodel species and the investigation of wildlife disease and immunity.

## Methods

Five published marsupial genomes—koala [57, 63, 64], woylie [65], wombat [63], antechinus [66], and numbat [67] (Table 1)—and 1 monotreme genome, platypus [41], were selected for this study based on use of different sequencing technologies (alone and in combination) and variation in assembly quality. These include assemblies generated using multiple data types (koala and platypus), long and short reads (woylie), short reads and HiC (wombat), or 10× Chromium linked reads (antechinus and numbat). BUSCO scores were generated by uploading the 6 genome assemblies to the Galaxy web platform [115], where the public server at galaxy.org was used to run BUSCOv5.3.2 [35] against the mammalian database.

Immune genes were annotated in the koala (phaCin_unsw_v4.1_HiC) [57, 63, 64], antechinus (anrechinusM_pseudohap2.1) [66], woylie (mBetpen1.pri.20210916) [65], wombat (vu-2k) [63, 64], and numbat genome (mMyrfas1.pri.20210917) [67] using multiple search strategies. BLAST was used to search genome assemblies, associated annotation files, and/or transcriptomes using published marsupial, monotreme, and eutherian immune gene sequences as queries, with default parameters and an e-value threshold of 10 so as not to exclude any potential gene candidates. HMMERv3.2 [116] was also used to identify putative genes within immune families that are known to contain duplications in other marsupials, such as NK receptors. Hidden Markov models (HMMs) were constructed using ClustalW alignments of published marsupial and eutherian immune gene sequences constructed in BioEditv7.2.5 [117], which were then used to search all genomes and transcriptomes using HMMER v3.2 with an e-value threshold of 10. For variable segments of T-cell receptor and immunoglobulin families, recombination signal sequences (RSS) downloaded from the IMGT database [97] and published koala sequences [57] were aligned using ClustalW in BioEditv7.2.5 [117] and used to construct HMMs. These RSS HMMs were then used to search each genome using HMMERv3.2 [116], to identify conserved RSS that flank each variable segment. For NK receptors, putative NKC and LRC sequences from BLAST+v2.7.1 [68] and HMMERv3.2 [116] searches were queried against the swissprot nonredundant database, and any sequences with top hits to swissprot NK genes, marsupial-specific NK genes, or the protein families database (Pfam) [118] immunoglobulin domain PF00047 or C-type lectin domain PF00059 HMM model were retained. IGSF domains within putative NK sequences from each species were identified using the Simple Modular Architecture Research Tool (SMART) database [119], and IGSF domains within 5 kbp were considered exons of a single LRC gene. Putative immune genes were named following the appropriate nomenclature for each family, with duplicated genes named according to their genomic location from the 5′ to 3′ end of the locus. For each immune gene family, amino acid sequences from all 5 species, in addition to other marsupial, monotreme, and eutherian sequences, were aligned using ClustalW in BioEditv7.2.5 [117]. This alignment was then used to construct neighbour-joining phylogenetic trees in MEGAXv10.2.4 [120] using the p-distance method, pairwise deletion, and 1,000 bootstrap replicates.

To investigate the impact of genome assembly quality on immune gene annotation, as well as discount species differences from our assessment, Fgenesh++ v7.2.2 [33] was used to annotate 2 different assemblies of the platypus genome: GCA_004115215.4 generated using multiple data types [41], and GCA_002966995.1 generated using only long- and short-read data. In addition, Fgenesh++ v7.2.2 [33] was used to annotate the koala and wombat genome assemblies to investigate the influence of the automated annotation method on immune gene annotation. To generate mRNA evidence for input to Fgenesh++, RNA-seq data from 19 platypus tissues and 16 koala tissues accessioned with the NCBI Sequence Read Archive (SRA) (Supplementary Table S3) were used to generate reference-guided global transcriptomes for each genome assembly (koala, platypus GCA_004115215.4 and GCA_002966995.1). No wombat RNA-seq data were available on the SRA; hence, a global transcriptome was not generated for this species. Briefly, raw RNA-seq reads were quality and length trimmed using Trimmomatic v0.39 [121] with the following parameters: ILLUMINACLIP:TruSeq3-SE.fa:2:30:10 SLIDINGWINDOW:4:5 LEADING:5 TRAILING:5 MINLEN:25. Over 90.53% of paired trimmed reads were retained for all 35 datasets (Supplementary Table S3). Trimmed reads were then aligned to the respective species genome and assembly version, using HISAT2 v2.1.0 [122] with default parameters. Resulting sam files were converted to sorted bam files using SAMTOOLS v1.9 [123], and then StringTie v2.1.6 [124] was used to generate gtf files for each tissue. Tama merge [125] was then used to merge aligned reads for each tissue into a single global transcriptome for each genome assembly (koala, platypus GCA_004115215.4 and GCA_002966995.1), with a 5′ threshold of 3 and a 3′ threshold of 500. CPC2 [126] was used to determine the coding potential of each transcript and Transdecoder v2.0.1 [127] to predict open reading frames within each transcript, for each global transcriptome.

The wombat, koala, and 2 platypus genome assemblies (GCA_004115215.4 and GCA_002966995.1) were annotated using Fgenesh++ v7.2.2 with general mammalian parameters using a custom machine at the Pawsey Supercomputing Centre with 64 CPUs, 256 GB RAM, and 1 TB of disk storage. An optimised gene-finding matrix from Tasmanian devils was used for koala and wombat genome annotations, while the platypus gene-finding matrix was used for both platypus genome assembly annotations. Transcripts with the longest open reading frame for each predicted gene were extracted from the global transcriptomes for platypus and koala as outlined in the previous section and used as mRNA-based gene predictions. The compute wall-time required to complete each annotation was as follows: wombat, 8 days, 1 hour, and 15 minutes; koala, 7 days, 8 hours, and 38 minutes; platypus GCA_002966995.1, 2 days, 2 hours, and 37 minutes; and platypus GCA_004115215.4, 1 day, 16 hours, and 13 minutes.

## Additional Files

Additional file 1_amended.xlsx

Additional file 2_amended.docx

Title of data: Supplementary Table S1

Description of data: Genomic coordinates of manually annotated immune genes in the koala, woylie, wombat, antechinus, and numbat genomes. The genomic coordinates of published platypus immune genes used in this study are also included.

File name: Additional File 2

File format: .doc

Title of data: Supplementary results

Description of data: A comprehensive comparison of manually annotated immune genes in this study to those in other marsupials and humans is provided in Supplementary Table 2. For each immune gene family characterised in this study, a summary of results and phylogenetic analysis is provided. This includes genes encoding Toll-like receptors, natural killer receptors, cytokines (interferons, interleukins, and tumour necrosis factors), T-cell receptor constant and variable regions (all 5 chains in marsupials and monotremes), immunoglobulin constant and variable regions (heavy and light chains), and major histocompatibility complex class I, II, and III genes. Additional File 2 contains 7 tables and 14 figures.

giac100_GIGA-D-22-00064_Original_Submission

giac100_GIGA-D-22-00064_Revision_1

giac100_GIGA-D-22-00064_Revision_2

giac100_Response_to_Reviewer_Comments_Original_Submission

giac100_Response_to_Reviewer_Comments_Revision_1

giac100_Reviewer_1_Report_Original_Submission Michael Hiller -- 4/30/2022 Reviewed

giac100_Reviewer_1_Report_Revision_1 Michael Hiller -- 8/19/2022 Reviewed

giac100_Reviewer_2_Report_Original_Submission Aurora Ruiz-Herrera -- 5/2/2022 Reviewed

giac100_Reviewer_3_Report_Original_Submission Tim Sackton -- 5/11/2022 Reviewed

giac100_Supplemental_Files

## Data Availability

The published woylie and numbat genome and global transcriptome assemblies are available through Amazon Web Services Open Datasets Program [128], NCBI under BioProject accession PRJNA763700 and GigaDB for woylie [129], and PRJNA786364 and GigaDB [130] for numbat. The published koala genome assembly and annotation (phaCin_unsw_v4.1_HiC.fasta) are available from the DNAzoo website [131]. The published wombat genome assembly and annotation (vu-2k.fasta) are also available from the DNAzoo website [131]. The published antechinus genome assembly and annotation (anrechinusM_pseudohap2.1.fasta) are available from NCBI under BioProject accession PRJNA664282 and GigaDB [129], as well as the published platypus genome assembly and annotation (mOrnAna1.pri.v4) under BioProject accession PRJNA489114. Genomic coordinates for all immune gene sequences annotated in this study are available in Additional File 1. Supporting information for this study is available in Additional File 2. Data contained in this article for all species and annotations are available in the GigaScience database GigaDB [132], including BUSCO analyses, .gff files of the annotations, and all data used to create the figures and phylogenetic trees.

## Abbreviations

BAC: bacterial artificial chromosome; BLAST: Basic Local Alignment Search Tool; bp: base pair; BUSCO: Benchmarking Single Copy Gene Orthologs; cDNA: complementary DNA; DFTD: devil facial tumour disease; Gpb: giga base pair; HiC: high-throughput chromosome conformation capture; HMM: hidden Markov model; IG: immunoglobulin; IGSF: immunoglobulin superfamily; IFN: interferon; IMGT: International Immunogenetic Information System; kbp: kilo base pair; LRC: leukocyte receptor complex; MHC: major histocompatibility complex; Mbp: mega base pair; NCBI: National Center for Biotechnology Information; NK: natural killer receptor; NKC: natural killer complex; PacBio: Pacific Biosciences; Pfam: protein families database; RSS: recombination signal sequence; SMART: Simple Modular Architecture Research Tool; SNP: single-nucleotide polymorphism; TCR: T-cell receptor; TLR: Toll-like receptor.

### Competing Interests

The authors declare that they have no competing interests.

### Funding

This work has been funded by the Australian Research Council Centre of Excellence for Innovations in Peptide and Protein Science (CE200100012) and Discovery Project (DP180102465). L.S. was supported by LP180100244 and P.B. was supported by an Australian Postgraduate award. Y.Z. is supported by the China Scholarship Council.

### Authors’ Contributions

L.S. assembled and annotated the woylie genome and transcriptomes, P.B. assembled and annotated the numbat genome and transcriptomes, and E.P. assisted with both. E.P., P.B., L.S., Y.C., and Y.Z. annotated immune genes. K.B., C.J.H., and E.P. designed the study. E.P. drafted the manuscript, and all authors read and commented on drafts of the manuscript and have approved the submission.
