# Supplementary material for: Best genome sequencing strategies for annotation of complex immune gene families in wildlife
Source: Gigascience. 2022 Oct 30;11:giac100. doi: 10.1093/gigascience/giac100 (PMC9618407; doi:10.1093/gigascience/giac100)
Supplement: giac100_GIGA-D-22-00064_Revision_1 [file giac100_giga-d-22-00064_revision_1.pdf]

GigaScience

Best genome sequencing strategies for annotation of complex immune gene families in wildlife

--Manuscript Draft--

**Manuscript Number:**	GIGA-D-22-00064R1
**Full Title:**	Best genome sequencing strategies for annotation of complex immune gene families in wildlife
**Article Type:**	Research
**Funding Information:**	Australian Research Council (CE200100012) — Prof Katherine Belov<br>Australian Research Council (DP180102465) — Prof Katherine Belov
**Abstract:**	Background<br><br>The biodiversity crisis and increasing impact of wildlife disease on animal and human health provides impetus for studying immune genes in wildlife. Despite the recent boom in genomes for wildlife species, immune genes are poorly annotated in non-model species owing to their high level of polymorphism and complex genomic organisation. Our research over the past decade and a half on Tasmanian devils and koalas highlights the importance of genomics and accurate immune annotations to investigate disease in wildlife. Given this, we have increasingly been asked the minimum levels of genome quality required to effectively annotate immune genes in order to study immunogenetic diversity. Here we set out to answer this question by manually annotating immune genes in five marsupial genomes and one monotreme genome to determine the impact of sequencing data type, assembly quality and automated annotation on accurate immune annotation.Results<br><br>Genome quality is directly linked to our ability to annotate complex immune gene families, with long reads and scaffolding technologies required to reassemble immune gene clusters and elucidate evolution, organisation and true gene content of the immune repertoire. Draft quality genomes generated from short-reads with HiC or 10x Chromium linked-reads were unable to achieve this. Despite mammalian BUSCOv5 scores of up to 94.1 % amongst the six genomes, automated annotation pipelines incorrectly annotated up to 59% of manually annotated immune genes regardless of assembly quality or method of automated annotation.Conclusions<br><br>Our results demonstrate that long-reads and scaffolding technologies, alongside manual annotation, are required to accurately study the immune gene repertoire of wildlife species.<br>Keywords: immune gene, genome, quality, annotation, MHC, wildlife, disease
**Corresponding Author:**	Carolyn Hogg<br><br>AUSTRALIA
**Corresponding Author Secondary Information:**	
**Corresponding Author's Institution:**	
**Corresponding Author's Secondary Institution:**	
**First Author:**	Emma Peel
**First Author Secondary Information:**	
**Order of Authors:**	Emma Peel<br>Luke Silver<br>Parice Brandies

Powered by Editorial Manager® and ProduXion Manager® from Aries Systems Corporation

	Ying Zhu
	Yuanyuan Cheng
	Carolyn Hogg
	Katherine Belov
**Order of Authors Secondary Information:**	
**Response to Reviewers:**	Also attached as a file to maintain formatting for ease of reading.<br><br>Response to reviewers<br>Reviewer #1:<br>REVIEWER COMMENT: Knowledge about immune genes is critical for species conservation programs. However, immune genes occur in large gene clusters that are difficult to assemble and annotate. This important and timely study uses a number of marsupial genomes and the platypus to assess which sequencing technologies enable complete reconstructions of immune gene clusters and which methods enable annotations of these immune genes.<br><br>I have the following comments.<br><br>Since Fgenesh++ and Maker produce automatic annotations, I wonder why not all 6 genomes were annotated with these two methods? This would allow a comparison between Fgenesh++ against Maker. Maybe it is possible to annotate at least a few genomes with both methods.<br><br>RESPONSE: All six genomes were not annotated using Fgenesh++ and Maker as the authors wanted to utilise existing annotations available for 5 of the 6 genomes in our study (all except koala). The authors agree that re-annotating all genomes with both Fgenesh++ and Maker would enable a direct comparison between the two methods. However, determining the best automated annotation software for immune gene annotation was not the focus of this study, but rather the impact of assembly quality on immune gene annotation. A secondary aim of the paper was to investigate whether automated annotation software was able to accurately identify immune genes, compared to our manual annotations. While it is widely known within the field of wildlife immunogenetics that automated genome annotations fail to correctly characterise immune genes, to date there are no publications which quantitatively assess this observation.<br>The computation required to annotate all six genomes using both Fgenesh++ and MAKER was not feasible within the given three-month timeframe provided for changes to the manuscript. As such, the koala, wombat and 2021 platypus genomes have been annotated with Fgenesh++ which will enable investigation of how this popular annotation software performs for immune gene annotation within all genome assemblies of varying quality included in this study (woylie, antechinus and numbat were already annotated with Fgenesh++). The methods, results and figure 1 have been modified to reflect this. See lines 213-218, 261-273 of the results and below.<br>Additional supplementary figures have been generated in response to the reviewer's comment. See supplementary figure 3, 4 and 5 in Additional file 2.<br>Table 1 has been modified to also include all genome annotations used in this study. This includes existing published annotations by NCBI, MAKER and Fgenesh++, as well as Fgenesh++ annotations conducted as part of this study.<br><br>Lines 213-218<br>"We assessed how well our manual immune gene annotation aligned with automated annotations by Fgenesh++ (2018 platypus, woylie, koala, antechinus, numbat and wombat), MAKER (wombat) and the NCBI pipeline (2021 platypus). Inclusion of the 2021 platypus NCBI and wombat MAKER annotations ensures that any differences in automated and manual immune gene annotation were not due to deficiencies within the Fgenesh++ annotation pipeline, as the woylie, antechinus and numbat genomes were all annotated with Fgenesh++ using the same parameters."<br>Lines 263-275<br>"This pattern of poor immune gene annotation was not an artefact of inherent differences between automated annotation pipelines amongst the six genomes (NCBI, MAKER and Fgenesh++) nor genome quality, as similar patterns were observed for

Fgenesh++ annotations of the 2021 platypus and wombat genome generated as part of this study (Supplementary Figure 3, Supplementary Figure 4). Generally, the Fgenesh++ annotation resulted in fewer correctly annotated immune genes (≥90% overlap) compared to NCBI (2021 platypus) or MAKER (wombat) (Supplementary Figure 3). Although, the proportion of missing immune genes (0% overlap) was higher in the NCBI (2021 platypus) and MAKER (wombat) annotation than the Fgenesh++ annotation of both species genomes. As with NCBI and MAKER, Fgenesh++ poorly annotated TCR and IG families at the gene-level (Supplementary Figure 4) in the high-quality platypus and low-quality wombat. Correct annotations were somewhat recovered at the exon-level in both genomes (Supplementary Figure 5), although, the number of missing TCR and IG exons in the Fgenesh++ annotation was almost half that of NCBI and MAKER in platypus and wombat respectively."

REVIEWER COMMENT: Direct assessments of assembly quality should ideally be done on different assemblies of the same species to rule out real differences between species. Would it be possible to include previous koala or platypus genome that was much more fragmented?

RESPONSE: The authors agree that multiple versions of the same genome assembly would enable direct assessment of assembly quality on immune gene annotation. As such, the authors have annotated the latest 2021 version of the platypus genome assembly published by Zhou et al 2021 (NCBI ID GCA_004115215.4) and the previous 2018 version (GCA_002966995.1) with Fgenesh++. Platypus was selected as the species for this comparison over koala (the only other species in our study with multiple genome assemblies available) as the improvement in assembly metrics between the 2021 and 2018 platypus genome assemblies is more significant than the 2018 and 2020 koala genome assemblies. This is due to the addition of numerous data types to the 2021 platypus assembly since the 2018 version. Genome assembly metrics for the 2018 platypus genome have been added to Table 1. The results section "Relationship between genome quality and manual immune gene annotation" has been modified to include a comparison between the 2018 and 2021 platypus assemblies. Specifically, see lines 283-290 and below. Figure 2 has also been updated to include the 2018 platypus genome assembly.
Fgenesh++ was selected for automated annotation of the two platypus assemblies over other methods such as MAKER as this would enable direct comparison of Fgenesh++ performance across all genomes in this study.
Lines 285-292
"To rule out species-specific differences in our direct assessment of assembly quality on immune gene annotation, we annotated a previous version of the platypus genome from 2018 (GCA_002966995.1) with Fgenesh++ to enable comparison with our Fgenesh++ annotation of the 2021 platypus genome (GCA_004115215.4) also generated as part of this study. Compared to the 2021 assembly, the 2018 platypus assembly was more fragmented given the 6-fold increase in the number of contigs, 14-fold increase in the number of scaffolds, and associated 2-fold decrease in contig N50 and 4-fold decrease in scaffold N50 between the two assemblies. Despite these metrics, the 2018 platypus assembly is still highly contiguous as it was generated using long-read data."

REVIEWER COMMENT: Figure 1 shows a useful of all immune genes. However, some genes like TLRs are actually easy to annotate as they are have a standard gene structure. Therefore, it would be informative to provide in this figure a breakdown of how well the different immune gene families are annotates, as the authors nicely did in table 2. This would inform on which immune genes are particularly difficult to annotate.

RESPONSE: A breakdown of Figure 1 by immune family is now presented in Supplementary Figure 1 of Additional File 2. A breakdown of annotation at the exon-level by immune family has been added as Supplementary Figure 2. See lines 235-255 and below. Similar breakdown of this analysis by immune family have also been added for Fgenesh++ versus MAKER (wombat) or NCBI (2021 platypus) annotations of the platypus and wombat genome assemblies at the gene level for all seven families (Supplementary Figure 4), in addition to exon-level for TCR and IG families (Supplementary figure 5).
Lines 235-255
"A breakdown of this analysis by immune family revealed that marsupial- and

monotreme-specific immune genes which are not orthologous to those in eutherians were generally poorly annotated, regardless of automated pipeline or genome quality (Supplementary Figure 1). This was particularly the case for TCR and IG gene families, with up to 88% of genes in these families incorrectly annotated by automated pipelines (≤10% overlap) amongst the six species (Table 2). This is likely due to highly duplicated variable gene segments that do not encode conventional exon-intron splice sites which may hinder annotation with automated pipelines. Poor gene annotations of TCR and IG families was somewhat recovered at the exon level, as some TCR and IG variable gene segments were annotated as exons by automated pipelines. Correct annotation (≥90% overlap) of the TCR family increased from 0-2% at the gene level to 2-15% at the exon level amongst the six genomes (Supplementary Figure 2). This improvement was even greater for the IG family, with an increase from 0-2% correct annotation at the gene level to 15-43% at the exon level amongst the six genomes (Supplementary Figure 2). Despite this, up to 67% of TCR and IG variable segments were still not annotated at the exon level (0% overlap) amongst the six genomes, highlighting the difficulty in automated annotation of these regions. Similarly, marsupial-specific gene expansions within the leukocyte receptor complex (LRC) and monotreme-specific gene expansions within the natural killer complex (NKC) family of NK receptors were also poorly annotated by automated pipelines (Supplementary Figure 1). As with TCR and IG families, correct annotation increased from the gene- (0-28% marsupial LRC, 31% platypus NKC) to exon-level (6-65% marsupial LRC, 79% platypus NKC) (Table 2, Supplementary Figure 2), likely due to the presence of variable numbers of duplicated immunoglobulin superfamily (IGSF) domains and C-type lectin (CLEC) domains within each LRC and NKC gene respectively."

REVIEWER COMMENT: Figure 3B is not colorblind friendly.

RESPONSE: Colours in Figure 3B have been amended according to the colourblind friendly palette outlined in Wong, B. Points of view: Color blindness. Nat Methods 8, 441 (2011). https://doi.org/10.1038/nmeth.1618

REVIEWER COMMENT: Line 275: The discussion makes it clear that this is a scaffolding error and not a real inversion. This should be clarified here as well.

RESPONSE: This has been clarified in the text, see lines 345-347 and below.
"This organisation is unusual amongst mammalian TCR and is likely a result of the HiC scaffolding error and not a true inversion."

REVIEWER COMMENT: I fully agree with the value of the manual annotations. Therefore, it would be helpful to provide the manual annotations also as a gff3 or gtf file that provide the full exon structure. Additional file 2 only lists the start and end coordinates of genes with multiple exons. The assembly accession should also be listed.

RESPONSE: Additional file 1 (previously Additional file 2) has been amended to include both the gene and exon coordinates for all immune genes across the 7 genome assemblies.

REVIEWER COMMENT: As a suggestion: A haplotype-resolved assembly of a marsupial is likely not yet available, but such an assembly would provide an opportunity to further investigate the
influence of assembly quality and haplotype variation in immune genes.

RESPONSE: The authors agree with the reviewer's comment. However, a haplotype-resolved assembly for marsupials will be challenging to generate given current recommendations include the use of trios to completely resolve paternal and maternal haplotypes. Samples from trios are incredibly difficult to obtain for wildlife such as marsupials given the opportunistic nature of most sample collection. This would be especially difficult for marsupials which are threatened or endangered, or are not currently housed in captivity.

Reviewer #2:
REVIEWER COMMENT: In this work, Peel and collaborators asses the accuracy of immune gene annotation in marsupial species by comparing the outcome of manually

Powered by Editorial Manager® and ProduXion Manager® from Aries Systems Corporation

and automated annotation approaches. This allowed them to conclude that sequence data type and assembly quality determine the accuracy of gene annotation. I find the study interesting, although I have some general comments. I find that both the introduction and discussion sections would benefit from some re-structuring. Both sections are a bit long, with some repetitions. Also, the discussion section contains material from results. I would also appreciate more detailed figure legends.

RESPONSE: The authors thank reviewer 2 for their comments. In light of no specific changes provided by reviewer 2, and changes already made to both the discussion and introduction for reviewer 1 and 3, we took no further action.

Reviewer #3:
REVIEWER COMMENT: In this manuscript, Peel et al examine the impact of assembly quality and sequencing/assembly method on the ability to annotate complex genes of the immune system, using a case study the five marsupial genomes and one monotreme genome of varying quality. While the conclusions the authors present are not particularly surprising given what we know about genome assembly, this manuscript does a nice job outlining the reasons why higher quality (in particular, long-read) assemblies are important to facilitate annotation of these critical genes, and exploring in depth the impact of various aspects of assembly quality. The authors present their results in a convincing and clear way, and this work provides a useful summary for the genomics community.

I do have some minor comments that I hope will help improve this work, listed below.

1. The conditional "in wildlife" is perhaps a little confusing in the title, as I believe the issues the authors raise should be widely relevant to vertebrate, or at least mammalian, genomes, and "wildlife" is a term with varying colloquial definitions among the readership of Gigascience. Relatedly the discussion in the background section of the abstract, as well as the intro of the manuscript and some parts of the discussion, could probably focus on mammals generally, or even vertebrates, not wildlife specifically. It would also make sense to make the implicit vertebrate focus explicit.

RESPONSE: The authors agree that the issues raised in our manuscript would be applicable to many mammalian or vertebrate genomes. However, genomics projects for non-model species such as wildlife generally work within constraints that are not always applicable to mammalian or vertebrate genomes more broadly. These include budget considerations, access to samples (remote locations, permits, CITES listing, threat status) and sample quantity (volume and tissue types available, sample quality (opportunistic sampling, non-invasive samples, sub-optimal preservation method, no access to liquid nitrogen or -80 freezer), amongst many others. All these factors influence the type of genome sequencing available to wildlife genomics projects, and hence resulting assembly quality. Mammals and many vertebrates more broadly, do not generally face these multitude of challenges when generating reference genomes. While the link between input sample, assembly quality and curation to generate a high-quality assembly has been established in wildlife (Rhie et al 2021), what has not been assessed is the impact of assembly quality on functionally important regions of the genome, such as immune genes. Our aim was to provide guidance for the wildlife genomics community, particularly those working on species impacted by disease, on how different genome sequencing strategies impact quality of immune gene annotations.

REVIEWER COMMENT: 2. The introduction goes into extensive detail about the case study systems presented here - perhaps more detail than is really needed (e.g., lines 130 - 136 on DFTD and chlamydial vaccines). However, there is little background information about the specific immune gene families that are the focus of this work. The authors present a compelling argument for why studying these gene families is important, but some additional information to help guide readers who may not be expert in the specific immune families under discussion would be valuable. In particular, reminding readers why these genes in particular are such a challenge to annotate, with perhaps a brief overview of the six immune gene families that are the focus of the work.

RESPONSE: Additional detail regarding the six immune gene families that are the

focus of the manuscript, and why immune genes are challenging to annotate has been provided in the introduction at lines 66-101 and below for easy reference.
“The COVID-19 pandemic is one of many examples which highlight the ever-increasing importance of understanding wildlife immunity and disease to better understand and manage disease spill over [17]. In the case of wildlife threatened by disease, conservation questions are more challenging to answer and typically involve immunogenetic diversity which relies on accurate immune gene annotations. Immune genes in mammals can be classified into six major families based on their evolutionary history and function: T cell receptors (TCR), immunoglobulins (IG), major histocompatibility complex (MHC), natural killer (NK) receptors, toll-like receptors (TLR) and cytokines. Mammals utilise two antigen recognition systems: TCR and IG expressed by T lymphocytes and B lymphocytes respectively. TCR and IG are encoded in large clusters within the genome, each of which contain few constant sequences that define the receptor sub-type, and multiple highly duplicated variable segments that recognise and bind antigens. The number and sequence polymorphism of IG and TCR V segments varies significantly between mammalian species [18-20]. Another major family of immune genes is the major histocompatibility complex which contains three classes of genes (class I, II and III). MHC class I and II genes encode cell-surface receptors which bind and present self- and pathogen-derived antigens to T lymphocytes, activating the adaptive immune response. Class I and II genes evolve via duplication and can be highly polymorphic, hence gene number differs between species [21, 22]. Natural killer (NK) cells directly kill virus-infected and cancerous cells and are an important component of innate immunity. Their activity is mediated via cell-surface receptors encoded by genes classified into two functionally similar but structurally dissimilar families; the leukocyte receptor complex (LRC) and natural killer complex (NKC). These families are encoded in separate clusters within the genome, and as they evolve via gene duplication, gene number varies significantly between species [23]. TLRs are membrane-spanning receptors expressed by immune and non-immune cells which bind pathogen-associated molecular patterns (PAMP), activating the innate and adaptive immune response. Compared to other immune genes, TLRs gene number and sequence is relatively conserved across mammals [24]. Lastly, cytokines are small proteins secreted by numerous cell types which direct the immune response. Cytokines can be classified into multiple families including interferons (IFN), tumour necrosis factors (TNF) and interleukins (IL), and gene content within each family varies between mammals [25].
Immune genes are some of the most polymorphic regions of the genome, owing to the need to generate diversity in response to ever-changing pathogenic pressures [26, 27]. Diversity within these gene families is generated through gene duplication, gene copy number variation, SNPs and rapid evolution, resulting in a complex genomic organisation and high level of pseudogenization [26]. Generally, immune genes are encoded within repetitive clusters in the genome, especially highly duplicated families such as the MHC and NK receptors [28]. Given these factors, accurate assembly and annotation of genomic regions encoding immune genes can be challenging [29-31], especially in wildlife.”

REVIEWER COMMENT: 3. I would recommend ordering the species in Table 1, Table 2, Figure 1, Figure 2, and Figure 3 in a consistent order, perhaps from highest to lowest contig N50. This will help readers keep track of the key patterns.

RESPONSE: Ordering of species and immune families in figures and tables (except for table 1) in the main manuscript and Additional file 2 is now consistent with the reviewer’s suggestion. Species are presented in the order of platypus, koala, woylie, wombat, antechinus then numbat, and immune families are presented in the order of cytokines, TLR, MHC, NKC, LRC, IG and TCR.

REVIEWER COMMENT: 4. The authors present a qualitative assessment of the kinds of genes where automated annotation fails in lines 202-212 and Fig 3. However a quantitative breakdown here would also I think be useful to the community, and should be easy to generate. One could simply list the fraction of manually annotated genes correctly recovered (and completely missed with <10% overlap) for each class in Table 2 for each species. This would also allow the authors to put some numbers alongside statements in this paragraph like "Most of these genes comprised... [line 210]"

RESPONSE: ≥90% and ≤10% overlap in genomic coordinates between manual and

Powered by Editorial Manager® and ProduXion Manager® from Aries Systems Corporation

automated annotation of immune genes has been added for each species and immune family in table 2. A quanitative breakdown has been added to this section of the results. See lines 235-255 and below. The authors have also added additional detail regarding automated versus manual immune annotation at the exon-level for the TCR, IG and LRC families which were poorly annotated by automated pipelines at the gene-level. Lines 235-255

“A breakdown of this analysis by immune family revealed that marsupial- and monotreme-specific immune genes which are not orthologous to those in eutherians were generally poorly annotated, regardless of automated pipeline or genome quality (Supplementary Figure 1). This was particularly the case for TCR and IG gene families, with up to 88% of genes in these families incorrectly annotated by automated pipelines (≤10% overlap) amongst the six species (Table 2). This is likely due to highly duplicated variable gene segments that don’t encode conventional exon-intron splice sites which may hinder annotation with automated pipelines. Poor gene annotations of TCR and IG families was somewhat recovered at the exon level, as some TCR and IG variable gene segments were annotated as exons by automated pipelines. Correct annotation (≥90% overlap) of the TCR family increased from 0-2% at the gene level to 2-15% at the exon level amongst the six genomes (Supplementary Figure 2). This improvement was even greater for the IG family, with an increase from 0-2% correct annotation at the gene level to 15-43% at the exon level amongst the six genomes (Supplementary Figure 2). Despite this, up to 67% of TCR and IG variable segments were still not annotated at the exon level (0% overlap) amongst the six genomes, highlighting the difficulty in automated annotation of these regions. Similarly, marsupial-specific gene expansions within the leukocyte receptor complex (LRC) and monotreme-specific gene expansions within the natural killer complex (NKC) family of NK receptors were also poorly annotated by automated pipelines (Supplementary Figure 1). As with TCR and IG families, correct annotation increased from the gene- (0-28% marsupial LRC, 31% platypus NKC) to exon-level (6-65% marsupial LRC, 79% platypus NKC) (Table 2, Supplementary Figure 2), likely due to the presence of variable numbers of duplicated immunoglobulin superfamily (IGSF) domains and C-type lectin (CLEC) domains within each LRC and NKC gene respectively.”

REVIEWER COMMENT: 5. I am not sure the statement (298-299): "that a kitchen sink approach, that uses long-read data combined with HiC technology, to generate a high-quality genome assembly is required to investigate immunity and disease in wildlife" is fully supported by the results the authors present. The annotation of the woylie genome, which as I understand it does not include any HiC scaffolding, seems to be as good or nearly as good as the two kitchen sink genomes. I would propose that the key conclusion is that long-read data specifically (with or without HiC) and high contig N50 (probably at least 1 Mb) is what is required for a successful manual annotation of these complex immune genes. This issue resurfaces in the discussion section, where again the point that HiC + Illumina is not sufficient is quite clear, but the converse does not seem well supported: long-read data in the absence of HiC does just fine.

RESPONSE: The authors agree that this statement could be improved. Our results do support the reviewer’s suggestion that assemblies based on long-read data, with or without scaffolding technology, are required for successful immune gene annotation. However, as outlined in the results section lines 298-320, immune gene families in the kitchen sink genomes represented by the 2021 platypus and koala assemblies were more intact than the woylie or 2018 platypus assembly (results presented in lines 368-380), both of which are based on long-read data. This was especially true for highly duplicated families such as the MHC, LRC NK receptors and TCR. The opening statement of the discussion has been modified to reflect the reviewer’s suggestion, see lines 382-390 and below.

“By manually annotating immune genes in five marsupial genomes and two versions of the platypus genome, all varying qualities, we have confirmed that genome quality is directly linked to our ability to annotate complex immune gene families. Without long reads and scaffolding technologies, immune genes are scattered across many individual scaffolds and gene family organisation and evolution cannot be elucidated. We conclude that long-read data, with or without HiC technology, to generate a high-quality genome assembly with a contig N50 of at least 1MB is required to investigate immunity and disease in wildlife. However, a kitchen sink approach to genome sequencing and assembly will enable complete reconstruction of complex and duplicated families such as MHC, TCR and LRC NK receptors as in the platypus 2021

Powered by Editorial Manager® and ProduXion Manager® from Aries Systems Corporation

and koala genomes.”

REVIEWER COMMENT: 6. The discussion of the limits of automated annotation is very important, but I found this section (starting on line 311) a little muddled. One key clarification is that it would probably be useful to separately discuss TCR and IG variable segments from all other immune genes. As the authors mention, automated analysis is not expected to successfully recover these variable regions, and it would probably be more useful to readers to get a sense of how automated analysis and RNA-seq alignment performs excluding these elements, in addition to the discussion on lines 329-337 of the specific challenges of variable regions.

RESPONSE: This section of the discussion has been revised in response to the reviewer’s comment and additional detail added. Automated annotation and RNAseq support for immune genes other than TCR and IG is now discussed in lines 408-437, while TCR and IG are solely discussed in lines 424-434. See amended text below.
“Aside from TCR and IG, the majority of immune genes incorrectly annotated or missing from the automated annotations were divergent genes not orthologous to those in eutherian mammals, such as MHC, marsupial-specific gene expansions within the LRC and monotreme-specific gene expansions within the NKC. Given their divergence, these genes often have low or no BLAST homology to nucleotide or protein databases. Gene models generated by automated annotation software are hypotheses based on supporting evidence such as RNAseq data and homology to nucleotide and protein databases. While immune transcripts were identified in the transcriptomes from these species, RNAseq data only supported gene models for a low proportion of MHC, LRC and NKC genes. RNAseq data only supported 8-16% of LRC gene predictions and 16-37% of MHC gene predictions amongst the four marsupial genome annotations which used RNAseq data as gene model evidence (koala, woylie, antechinus and numbat). Similarly, around 60% of NKC genes within the platypus genomes were supported by RNAseq data. Overall, RNAseq data did not provide enough evidence to support gene models for ~20% of immune genes within the genome. Some immune genes may not have been expressed in the tissue sequenced, were expressed at low levels, or were fragmented. For human and mouse, comprehensive and curated gene sets such as GENCODE and RefSeq are available to guide gene model predictions, comprising data from more than 10,000 RNA experiments and decades of dedicated work in this field [95, 96]. Given time, budget and sample constraints for wildlife, these curated gene sets are not available, hence RNAseq evidence is incomplete resulting in deficient gene models by automated annotation software.
It is not surprising that TCR and IG V segments were poorly or not annotated by all automated pipelines used to annotate the genomes in this study. These genes are notoriously difficult to characterise and are manually annotated in the human and mouse genome on Ensembl using the International Immunogenetics Information System (IMGT) database [38, 97]. Alignment of mature IG and TCR sequences from RNAseq data to the genome results in poor automated annotation, as V segments utilize different sequence signal splice sites to introns, which are not recognized by the open reading frame prediction algorithms. Indeed, RNAseq evidence only supported 7% to 18% of TCR V segment and 0% to 6.9%% of IG V segment gene predictions by automated pipelines amongst the four marsupial and platypus genomes. V sequences from three marsupials and two monotremes are available in IMGT, however as non-model species, they are not included in the scope for manual annotation by Ensembl or NCBI, so these important functional features are not annotated.”

REVIEWER COMMENT: 7. Regarding "it is not a requirement for manual changes to annotations to be tracked between genome versions" on line 353, I am not sure this is so simple. Even lifting over the old manual curation to new assembly coordinates probably needs itself to be manually verified before one can be confident that the new model is correct. But I do not think this would mean the information is lost, as I believe NCBI and Ensembl both maintain old annotations and assembly versions.

RESPONSE: The authors agree that this statement was vague and so has been removed from the manuscript. While NCBI and Ensembl maintain old annotations and assembly versions, our argument still stands as there is currently limited scope to include manual gene annotations of the scale presented in our manuscript alongside existing automated annotations from these databases.

	REVIEWER COMMENT: 8. Given that 10x linked reads are no longer available for genome assembly, the extensive discussion of their uses and limitations on lines 431-457 could probably be condensed considerably.<br><br>RESPONSE: This section of the discussion has been condensed, see lines 531—552 and text below. However, the authors feel discussing the limitations of 10x genomes for immune gene annotation is still warranted to make use of existing 10x assemblies, particularly for species where additional genome sequencing is unlikely due to sample or budget constraints.<br>“10x Chromium linked-read sequencing was insufficient to accurately re-assemble immune gene clusters in our study (Figure 4C). While this technology is no longer available for genome sequencing, acknowledging the limitations of this technology for immune gene annotation remains valid in order to make use of existing 10x genomes. Complete marsupial immune gene clusters can span hundreds of kilobases to megabases, as shown by annotation of the complete MHC, NK receptor and TCR regions in the koala (Additional file 2). DNA molecules input to 10x library preparation were on average 74 kbp and 23 kbp in antechinus and numbat respectively. This molecule size only spanned smaller immune clusters in the antechinus, such as the 70 kbp TRG locus, but was insufficient to span any cluster in the numbat. Interestingly, the antechinus MHC cluster appears to be intact (Figure 3), however manual annotation revealed multiple genes were “missing” within the scaffold and instead were located on individual short scaffolds. Regardless of input DNA molecule length, 10x libraries are still subject to the limitations of short-read sequencing regarding assembly of complex sequences. Antechinus and numbat 10x libraries were sequenced as short ~150 bp reads, hence while reads can be assigned back to the corresponding input DNA molecule, no single read spans the molecule length. Gaps between the reads make de novo assembly of repetitive and complex immune sequences difficult, often resulting in termination of contig extension and gene fragments scattered across short scaffolds [109-111]. These gene fragments can be misinterpreted as pseudogenes owing to loss of up/downstream coding regions (Figure 4C). For example, antechinus and numbat NK LRC genes share up to 97% and 98% amino acid sequence identity amongst the genes identified in each species respectively. The LRC should be encoded within a single cluster, as in the koala genome (Figure 3). Instead, the antechinus and numbat LRC clusters are fragmented across 33 and 34 scaffolds respectively.”
**Additional Information:**	
**Question**	**Response**
Are you submitting this manuscript to a special series or article collection?	No
**Experimental design and statistics**<br><br>Full details of the experimental design and statistical methods used should be given in the Methods section, as detailed in our Minimum Standards Reporting Checklist. Information essential to interpreting the data presented should be made available in the figure legends.<br><br>Have you included all the information requested in your manuscript?	No
If not, please give reasons for any omissions below.	The data included in this manuscript uses published genomic data to show complexities of immune gene annotation with varying degrees of genome quality. The method design is around genome assembly and annotation and all the relevant metrics

as follow-up to "**Experimental design and statistics**<br><br>Full details of the experimental design and statistical methods used should be given in the Methods section, as detailed in our Minimum Standards Reporting Checklist. Information essential to interpreting the data presented should be made available in the figure legends.<br><br>Have you included all the information requested in your manuscript?<br><br>"	are included in the manuscript.
**Resources**<br><br>A description of all resources used, including antibodies, cell lines, animals and software tools, with enough information to allow them to be uniquely identified, should be included in the Methods section. Authors are strongly encouraged to cite Research Resource Identifiers (RRIDs) for antibodies, model organisms and tools, where possible.<br><br>Have you included the information requested as detailed in our Minimum Standards Reporting Checklist?	Yes
**Availability of data and materials**<br><br>All datasets and code on which the conclusions of the paper rely must be either included in your submission or deposited in publicly available repositories (where available and ethically appropriate), referencing such data using a unique identifier in the references and in the "Availability of Data and Materials" section of your manuscript.<br><br>Have you have met the above	Yes

Powered by Editorial Manager® and ProduXion Manager® from Aries Systems Corporation

requirement as detailed in our Minimum Standards Reporting Checklist?	

Manuscript

Click here to access/download;Manuscript;Manuscript_amended.docx

Best genome sequencing strategies for annotation of complex immune gene families in wildlife

Emma Peel[1,2], Luke Silver[1], Parice Brandies[1], Ying Zhu[3], Yuanyuan Cheng[1], Carolyn J Hogg[1,2] & Katherine Belov[1,2]*

[1]School of Life and Environmental Sciences, The University of Sydney, Sydney, New South Wales, Australia

[2] Australian Research Council Centre of Excellence for Innovations in Peptide and Protein Science, University of Sydney, NSW 2006, Australia

[3] Sichuan Provincial Academy of Natural Resource Sciences, Chengdu, Sichuan, China

emma.peel@sydney.edu.au ORCID: 0000-0002-2335-8983

luke.silver@sydney.edu.au ORCID: 0000-0002-1718-5756

parice.brandies@sydney.edu.au ORCID: 0000-0003-1702-2938

so_zy2003@126.com

yuanyuan.cheng@sydney.edu.au ORCID: 0000-0002-1747-9308

carolyn.hogg@sydney.edu.au ORCID: 0000-0002-6328-398X

*Corresponding author kathy.belov@sydney.edu.au ORCID: 0000-0002-9762-5554

Abstract

Background

The biodiversity crisis and increasing impact of wildlife disease on animal and human health provides impetus for studying immune genes in wildlife. Despite the recent boom in genomes for wildlife species, immune genes are poorly annotated in non-model species owing to their high level of polymorphism and complex genomic organisation. Our research over the past decade and a half on Tasmanian devils and koalas highlights the importance of genomics and accurate immune annotations to investigate disease in wildlife. Given this, we have increasingly been asked the minimum levels of genome quality required to effectively annotate immune genes in order to study immunogenetic diversity. Here we set out to answer this question by manually annotating immune genes in five marsupial genomes and one monotreme genome to determine the impact of sequencing data type, assembly quality and automated annotation on accurate immune annotation.

Results

Genome quality is directly linked to our ability to annotate complex immune gene families, with long reads and scaffolding technologies required to reassemble immune gene clusters and elucidate evolution, organisation and true gene content of the immune repertoire. Draft quality genomes generated from short-reads with HiC or 10x Chromium linked-reads were unable to achieve this. Despite mammalian BUSCOv5 scores of up to 94.1 % amongst the six genomes, automated annotation pipelines incorrectly annotated up to 59% of manually annotated immune genes regardless of assembly quality or method of automated annotation.

Conclusions

Our results demonstrate that long-reads and scaffolding technologies, alongside manual annotation, are required to accurately study the immune gene repertoire of wildlife species.

Keywords: immune gene, genome, quality, annotation, MHC, wildlife, disease

Background

Globally we are facing a biodiversity crisis, with 25% of known plant and animal species under threat and one million species facing extinction [1]. Disease is one of many drivers of global wildlife decline and extinction, with recent devastating examples such as chytridiomycosis in amphibians [2], white nose syndrome in bats [3] and devil facial tumour disease (DFTD) in Tasmanian devils (*Sarcophilus harrisii*) [4]. Habitat loss, fragmentation and climate change lead to population decline and subsequent loss of genetic diversity, which increases susceptibility of populations to new and existing disease threats [5].

Genomics is increasingly applied in conservation [6] facilitated by a boom in genomes for wildlife species [7-10], with over 4,000 vertebrate genomes currently accessioned with the National Center for Biotechnology Information (NCBI) (March 2022). Genomics in conservation typically involves technologies such as reduced representation sequencing which capture single nucleotide polymorphisms (SNPs) with a bias towards neutral regions of the genome [11, 12]. This can be used to investigate population genetic metrics such as heterozygosity, inbreeding and relatedness to inform conservation management. This is a cost-effective approach for conservation and has been used in a range of taxa to inform conservation actions, for examples see Tasmanian devils [13], gorillas (*Gorillia gorilla gorilla* and *Gorilla beringei graueri*) [14], helmeted honeyeaters (*Lichenostomus melanops cassidix*) [15] and bilbies (*Macrotis lagotis*) [16].

The COVID-19 pandemic is one of many examples which highlight the ever-increasing importance of understanding wildlife immunity and disease to better understand and manage disease spill over [17]. In the case of wildlife threatened by disease, conservation questions are more challenging to answer and typically involve immunogenetic diversity which relies on accurate immune gene annotations. Immune genes in mammals can be classified into six major families based on their evolutionary history and function: T cell receptors (TCR), immunoglobulins (IG), major histocompatibility complex (MHC), natural killer (NK) receptors, toll-like receptors (TLR) and cytokines. Mammals utilise two antigen

recognition systems: TCR and IG expressed by T lymphocytes and B lymphocytes respectively. TCR and IG are encoded in large clusters within the genome, each of which contain few constant sequences that define the receptor sub-type, and multiple highly duplicated variable segments that recognise and bind antigens. The number and sequence polymorphism of IG and TCR V segments varies significantly between mammalian species [18-20]. Another major family of immune genes is the major histocompatibility complex which contains three classes of genes (class I, II and III). MHC class I and II genes encode cell-surface receptors which bind and present self- and pathogen-derived antigens to T lymphocytes, activating the adaptive immune response. Class I and II genes evolve via duplication and can be highly polymorphic, hence gene number differs between species [21, 22]. Natural killer (NK) cells directly kill virus-infected and cancerous cells and are an important component of innate immunity. Their activity is mediated via cell-surface receptors encoded by genes classified into two functionally similar but structurally dissimilar families; the leukocyte receptor complex (LRC) and natural killer complex (NKC). These families are encoded in separate clusters within the genome, and as they evolve via gene duplication, gene number varies significantly between species [23]. TLRs are membrane-spanning receptors expressed by immune and non-immune cells which bind pathogen-associated molecular patterns (PAMP), activating the innate and adaptive immune response. Compared to other immune genes, TLRs gene number and sequence is relatively conserved across mammals [24]. Lastly, cytokines are small proteins secreted by numerous cell types which direct the immune response. Cytokines can be classified into multiple families including interferons (IFN), tumour necrosis factors (TNF) and interleukins (IL), and gene content within each family varies between mammals [25].

Immune genes are some of the most polymorphic regions of the genome, owing to the need to generate diversity in response to ever-changing pathogenic pressures [26, 27]. Diversity within these gene families is generated through gene duplication, gene copy number variation, SNPs and rapid evolution, resulting in a complex genomic organisation and high level of pseudogenization [26]. Generally, immune genes are encoded within repetitive clusters in the genome, especially highly

duplicated families such as the MHC and NK receptors [28]. Given these factors, accurate assembly and annotation of genomic regions encoding immune genes can be challenging [29-31], especially in wildlife.

Automated annotation pipelines such as MAKER [32] and Fgenesh++ [33] are accurate at identifying the majority of protein-coding genes within a genome [34, 35]. However, they are less effective at characterising complex and highly variable gene families such as immune genes [36, 37] which are misassembled even in the high-quality human genome [29]. As such, manual annotation and curation of immune genes is required, which is conducted for model organism genomes accessioned with Ensembl [38]. Wildlife are not currently included in this scope, and hence immune genes are poorly annotated, or not annotated at all, in many species.

Advances in sequencing technology means chromosome-length genomes are now achievable for a range of species [8]. Use of multiple sequencing, scaffolding, chromatin conformation and optical mapping technologies leads to accurate assembly of complex and variable genomic regions, such as immune genes [8]. However, the high input sample quantity and quality requirements are not always feasible for wildlife [39]. This leads to the use of lower-input short-read sequencing to generate a draft-quality genome assembled into scaffolds. However, short-read sequencing is well known to be incompetent at resolving highly repetitive and complex gene regions [40, 41]. While scaffolding technologies can improve contiguity of these assemblies, complex and variable regions often remain fragmented. The need to balance budget, sample and genome assembly quality against accurate immune gene annotation is essential to answer questions around disease and immunity.

Over the past decade and a half our research has focused on immunity and disease in two iconic marsupial species; the Tasmanian devil and koala (*Phascolarctos cinereus*). During this period, we have worked with bacterial artificial chromosome (BAC) and complementary DNA (cDNA) libraries and draft genomes of varying qualities. Our research, and that of others, has been crucial for understanding, managing and preventing disease-induced decline [4, 42-44]. As the cost of sequencing has dropped,

and the appreciation of the power of genetics and genomics for population management has increased, we have increasingly been asked about the minimum levels of genome quality required to be able to effectively annotate immune genes in order to study levels of diversity in wild populations. Here we set out to answer that question.

Tasmanian devils are threatened by DFTD, a contagious cancer which has decimated over 80% of the population since it was first documented in 1996 [4]. The Tasmanian devil reference genome was sequenced using Illumina short-reads in 2012 [45], generating a 3.17 Gbp genome with a scaffold N50 of 1.8 Mbp and contig N50 of 20kbp. The Major Histocompatibility Complex was not able to be annotated in the draft genome due to the high levels of fragmentation, scattered across at least 15 scaffolds. But manual annotation was possible alongside transcriptomes [46-48] and targeted sequencing of MHC-positive BAC clones [46, 49-53]. Development of MHC markers led to determination of gene copy number and nucleotide variation amongst the devil population, revealing devils have low MHC diversity, much of which is shared with DFTD [51, 54]. The low histocompatibility barriers, coupled with downregulation of tumour MHC expression, allows DFTD to transmit between individuals and evade the host immune response [52]. Recent MHC genotyping using long-read sequencing enabled the identification of full-phased MHC alleles and separation of highly similar alleles (1bp difference), resulting in the identification of new functional MHC diversity within the devil population [55].

The koala is another iconic Australian marsupial where disease is a major contributing factor to population decline [56]. Chlamydiosis is one of many threatening processes affecting koalas, a disease caused by infection with the intracellular bacterium *Chlamydia pecorum* [56]. A chromosome-length koala reference genome was sequenced in 2018 using Pacific Biosciences (PacBio) long-reads, Illumina short-reads and BioNano optical maps [57]. This generated a 3.19 Gbp assembly with a scaffold N50 of 480 Mbp and contig N50 of 11.4 Mbp [57], a 400-fold increase in scaffold contiguity compared to the Tasmanian devil genome assembly [45]. This high-quality koala genome enabled accurate

annotation of immune gene families, including the first complete reconstruction of MHC and TCR gene clusters from a genome sequence in marsupials [43, 58-60]. Preliminary genome resequencing identified that variants within IFNγ, TNFα and MHC genes are essential for clearance of *Chlamydia* in koalas [42]. MHC genotype has also been linked to disease susceptibility and severity in different koala populations [61, 62].

In this study, our aim was to determine the impact of sequence data type, assembly quality and automated annotation on accurate immune annotation. To achieve this, we manually annotated immune genes in the genomes of five marsupials and one monotreme. These include recent published genome assemblies of five marsupials; koala [57, 63, 64], woylie (*Bettongia penicillata*) [65], common wombat (*Vombatus ursinus*) [63, 64], brown antechinus (*Antechinus stuartii*) [66] and numbat (*Myrmecobius fasciatus*) [67], and previous immune gene annotations from one monotreme, the platypus [41]. These six genomes differ in quality, from scaffold assemblies generated using only 10x Chromium linked-reads (numbat, antechinus), short-read with high-throughput chromosome conformation capture (HiC) (wombat), long and short-read (woylie), to high-quality chromosome-length genomes generated using multiple data types (koala and platypus) (Table 1). We assess the accuracy of automated immune gene annotation by Fgenesh++, MAKER and NCBI pipelines in these non-model species. To account for the impact of species-specific gene expansion/contraction on automated immune gene annotation, we also annotated two versions of the platypus genome from 2021 (GCA_004115215.4) and 2018 (GCA_002966995.1) with Fgenesh++. This study provides a guide of the impact of genome quality on immune gene annotation. Here we show that high quality chromosome-length genomes are necessary for accurate immune annotation in the context of wildlife disease.

Analyses

Immune genes were annotated in the koala, woylie, wombat, antechinus, and numbat genomes and transcriptomes using similarity-based search methods such as BLAST [68] and HMMER [69] with

known marsupial immune gene sequences as queries. This resulted in the manual characterisation of over 2,700 immune genes amongst the five species, from six immune gene families or groups: toll-like receptors (TLR), T cell receptors (TCR), immunoglobulins (IG), major histocompatibility complex (MHC), natural killer (NK) cell receptors and cytokines (Table 2). Platypus immune gene families have previously been annotated [41, 70-81], some of which had already been mapped within the 2021 genome assembly (MHC and TCR) [41] and the remainder were mapped in both the 2018 and 2021 assemblies in this study. Genomic coordinates of all immune genes annotated in this study are available in Additional file 1. A comprehensive summary of results for each immune gene family are available in Additional file 2.

Table 1. Assembly metrics and genome annotations for the five marsupial and two monotreme genome assemblies used in this study. The wombat and koala genome assemblies used in this study are not available on NCBI, hence the accession ID is not provided.

	Platypus		Koala	Woylie	Wombat	Antechinus	Numbat
Genome assembly version	GCA_004115215.4 [41]<br>2021	GCA_002966995.1<br>2018	phaCin_unsw_v4.1<br>[57, 63, 64]	GCA_023548195.1<br>[65]	vu-2k<br>[63, 64]	GCA_016696395.1<br>[66]	GCA_023553655.1<br>[67]
Data types	PacBio<br>10x Chromium<br>BioNano<br>HiC (Phase genomics & Dovetail)<br>RNAseq (19 transcriptomes)	PacBio<br>Illumina<br>RNAseq (19 transcriptomes)	PacBio RS II<br>Illumina<br>BioNano<br>HiC (DNAzoo)<br>RNAseq (16 transcriptomes)	PacBio<br>HiFi<br>Illumina<br>RNAseq (4 transcriptomes)	Illumina<br>HiC (DNAzoo)	10x Chromium<br>RNAseq (12 transcriptomes)	10x Chromium<br>RNAseq (3 transcriptomes)
Genome size (Gbp)	2.13	1.99	3.19	3.39	3.34	3.31	3.42
GC (%)	46.23	46.64	39.05	38.64	38.89	36.20	36.3
No. scaffolds	322	4,568	1,318	1,116	633,737	30,876	112,299
No. contigs	834	5,044	1,935	3,016	685,859	106,199	219,447
Scaffold N50 (Mbp)	83.33	18.71	480.11	6.94	576.1	72.7	0.223
Contig N50 (Mbp)	15.1	7.5	11.4	1.995	0.07	0.08	0.038
Gaps (%)	0.81	0.0002	0.01	0.403	0.54	2.75	3.52

Complete mammalian BUSCOv5.3.2	83.0%	81.5%	94.1%	94.1%	89.3%	92.5%	78.7%
Genome annotations used in this study	NCBI Fgenesh++ (this study)	Fgenesh++ (this study)	Fgenesh++ (this study)	Fgenesh+ + [65]	MAKER [63, 64] Fgenesh+ + (this study)	Fgenesh++ [66]	Fgenesh+ + [67]

Table 2. Number of annotated immune genes in each of the five marsupials and one monotreme in this study. The percentage overlap of genomic coordinates between manual and automated annotations of immune genes is also provided for each family and species.

	Platypus	Koala	Woylie	Wombat	Antechinus	Numbat
Cytokines	49 (48%, 8%)	82 (20%, 22%)	77 (19%, 38%)	76 (33%, 44%)	68 (17%, 21%)	67 (21%, 30%)
TLR	10 (90%, 10%)	10 (0%, 20%)	10 (6%, 37%)	10 (100%, 0%)	10 (10%, 20%)	10 (10%, 20%)
MHC I	6 (14%, 0%)	19 (21%, 21%)	17 (5%, 5%)	5 (60%, 0%)	7 (22%, 10%)	3 (22%, 11%)
MHC II	5 (25%, 25%)	16 (6%, 25%)	23 (12%, 16%)	7 (42%, 0%)	14 (33%, 6%)	6 (33%, 6%)
MHC III	58 (88%, 4%)	39 (11%, 7%)	37 (23%, 2%)	38 (65%, 7%)	36 (11%, 32%)	35 (12%, 12%)
Ext. MHC & framework genes	20 (100%, 0%)	27 (13%, 10%)	31 (32%, 8%)	34 (41%, 11%)	31 (21, 10%)	33 (11%, 42%)
NKC	122 (31%, 63%)	17 (27%, 11%)	17 (27%, 11%)	11 (9%, 36%)	11 (18%, 27%)	17 (33%, 5%)
LRC	4 (0%, 0%)	25 (3%, 18%)	60 (3%, 63%)	33 (28%, 54%)	49 (5%, 38%)	41 (5%, 38%)
Extended LRC	11 (36%, 0%)	6 (0%, 12%)	22 (0%, 60%)	9 (0%, 100%)	15 (37%, 18%)	11 (56%, 31%)
IG constant	14 (5%, 50%)	15 (0%, 66%)	20 (4%, 22%)	10 (16%, 66%)	7 (28%, 14%)	6 (0%, 33%)
IG variable	118 (0.5%, 80%)	289 (0%, 58%)	226 (0%, 58%)	98 (0.9%, 81%)	145 (0.6%, 43%)	121 (0%, 34%)
TCR constant	19 (0%, 88%)	10 (0%, 45%)	12 (0%, 29%)	10 (0%, 81%)	11 (0%, 36%)	9 (0%, 22%)
TCR variable	252 (0%, 78%)	103 (0%, 58%)	122 (0%, 76%)	92 (2%, 86%)	126 (0%, 59%)	104 (0%, 71%)
Total	678 (21%, 57%)	658 (5%, 41%)	674 (6%, 48%)	440 (21%, 57%)	531 (8%, 37%)	463 (9%, 38%)

Table 2 legend. Includes complete and partial gene sequences. A more detailed comparison of immune genes annotated in this study, with those identified in other marsupials and humans is available in Supplementary Table 2 within Additional file 2. The first percentage represents ≥90% overlap and the second represents ≤10% overlap between automated and manual annotations of

the respective immune genes for each species. Values for the NCBI annotation of the 2021 platypus genome are presented here.

Overall, the immune gene repertoire of the koala, woylie, wombat, antechinus, and numbat was similar to other marsupials [58, 82], with marsupial-specific genes and eutherian orthologs identified. Relatively conserved immune genes such as TLRs and constant regions of TCR and IG, as well as polymorphic genes such as MHC and NK receptors, were identified in all five species. Numerous koala immune gene sequences have been characterised previously due to their involvement in chlamydiosis and koala retrovirus which threaten populations [56]. These include MHC [57, 83-85], IG [58], TCR [57], NK receptors [59] and selected cytokines [58, 86-89] (Supplementary Table S2 in Additional file 2). We mapped the location of these genes within the current version of the genome, and identified additional new sequences within the LRC, IG and cytokine families (Table 2, Supplementary Table S2 in Additional file 2). Immune genes unique to the marsupial lineage were also characterised in the five species studied here. These included MHC class II genes DA, DB and DC, TLR1/6 and TCRμ. Large marsupial-specific gene expansions within the LRC NK receptors were characterised in all five species, as well as reduced gene content within the NKC cluster of NK receptors. Consistent with other marsupials investigated to date Igδ was not found in any of the five assemblies [90]. A detailed outline of immune genes annotated in this study compared to those of other marsupials and humans is provided in Supplementary Table S2 within Additional file 2.

Automated versus manual immune gene annotation

We assessed how well our manual immune gene annotation aligned with automated annotations by Fgenesh++ (2018 platypus, woylie, koala, antechinus, numbat and wombat), MAKER (wombat) and the NCBI pipeline (2021 platypus). Inclusion of the 2021 platypus NCBI and wombat MAKER annotations ensures that any differences in automated and manual immune gene annotation were not due to deficiencies within the Fgenesh++ annotation pipeline, as the woylie, antechinus and numbat genomes were all annotated with Fgenesh++ using the same parameters.

Automated annotation pipelines failed to characterise the complete immune repertoire of the platypus or any of the five marsupial species (Figure 1). Only 21.27%, 5.66%, 6.89%, 21.82%, 8.68%, 9.07% of immune genes were correctly annotated by the automated pipeline in the 2021 platypus, koala, woylie, wombat, antechinus, and numbat respectively, defined as ≥90% overlap in genomic coordinates of immune genes between our manual annotations and the automated annotations (Figure 1). Interestingly, more immune genes were correctly annotated by the automated software in the low-quality wombat, antechinus, and numbat genomes than the high-quality platypus, koala and woylie genomes. This inverse relationship between genome quality and proportion of correctly annotated immune genes is likely related to the characterisation of additional divergent and polymorphic genes such as MHC class I and II in woylie, koala and platypus, which could not be identified by automated or manual annotation in the wombat, antechinus, and numbat due to genome fragmentation (Table 3). All genomes analysed in this study displayed a high proportion of immune genes which were very poorly annotated by automated pipelines (≤10% overlap between immune gene coordinates from manual versus automated annotation); 57.01%, 41.78%, 48.96%, 57.01%, 37.05% and 38.22% for 2021 platypus, koala, woylie, wombat, antechinus, and numbat respectively (Figure 1).

A breakdown of this analysis by immune family revealed that marsupial- and monotreme-specific immune genes which are not orthologous to those in eutherians were generally poorly annotated, regardless of automated pipeline or genome quality (Supplementary Figure 1). This was particularly the case for TCR and IG gene families, with up to 88% of genes in these families incorrectly annotated by automated pipelines (≤10% overlap) amongst the six species (Table 2). This is likely due to highly duplicated variable gene segments that do not encode conventional exon-intron splice sites which may hinder annotation with automated pipelines. Poor gene annotations of TCR and IG families was somewhat recovered at the exon level, as some TCR and IG variable gene segments were annotated as exons by automated pipelines. Correct annotation (≥90% overlap) of the TCR family increased from 0-2% at the gene level to 2-15% at the exon level amongst the six genomes (Supplementary Figure 2).

This improvement was even greater for the IG family, with an increase from 0-2% correct annotation at the gene level to 15-43% at the exon level amongst the six genomes (Supplementary Figure 2). Despite this, up to 67% of TCR and IG variable segments were still not annotated at the exon level (0% overlap) amongst the six genomes, highlighting the difficulty in automated annotation of these regions. Similarly, marsupial-specific gene expansions within the leukocyte receptor complex (LRC) and monotreme-specific gene expansions within the natural killer complex (NKC) family of NK receptors were also poorly annotated by automated pipelines (Supplementary Figure 1). As with TCR and IG families, correct annotation increased from the gene- (0-28% marsupial LRC, 31% platypus NKC) to exon-level (6-65% marsupial LRC, 79% platypus NKC) (Table 2, Supplementary Figure 2), likely due to the presence of variable numbers of duplicated immunoglobulin superfamily (IGSF) domains and C-type lectin (CLEC) domains within each LRC and NKC gene respectively.

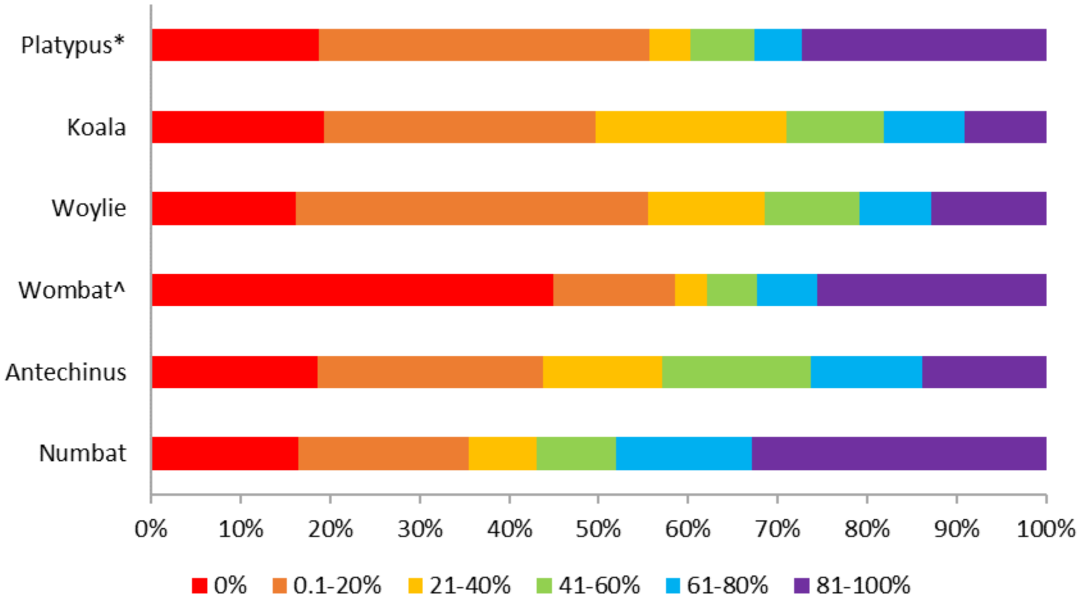

Figure 1. Percentage overlap of genomic coordinates between manual and automated annotations of immune genes in six genomes. *Denotes automated annotation by NCBI and ^denotes automated annotation by MAKER. The remaining genomes were annotated using Fgenesh++.

Figure 1 legend. Colours indicate proportion of immune genes with 0 to 100% overlap between manual and automated annotations, with 0 indicating manually annotated genes with no overlap of genomic coordinates with the automated annotation.

This pattern of poor immune gene annotation was not an artefact of inherent differences between automated annotation pipelines amongst the six genomes (NCBI, MAKER and Fgenesh++) nor genome quality, as similar patterns were observed for Fgenesh++ annotations of the 2021 platypus and wombat genome generated as part of this study (Supplementary Figure 3, Supplementary Figure 4). Generally, the Fgenesh++ annotation resulted in fewer correctly annotated immune genes (≥90% overlap) compared to NCBI (2021 platypus) or MAKER (wombat) (Supplementary Figure 3). Although, the proportion of missing immune genes (0% overlap) was higher in the NCBI (2021 platypus) and MAKER (wombat) annotation than the Fgenesh++ annotation of both species genomes. As with NCBI and MAKER, Fgenesh++ poorly annotated TCR and IG families at the gene-level (Supplementary Figure 4) in the high-quality platypus and low-quality wombat. Correct annotations were somewhat recovered at the exon-level in both genomes (Supplementary Figure 5), although, the number of missing TCR and IG exons in the Fgenesh++ annotation was almost half that of NCBI and MAKER in platypus and wombat respectively.

Relationship between genome quality and manual immune gene annotation

Manual annotation of immune genes across the koala, woylie, wombat, antechinus and numbat genomes, and mapping of previous annotations to both the 2018 and 2021 versions of the platypus genome, highlighted a clear relationship between immune gene fragmentation and genome quality (Figure 2). Overall, the high-quality koala, 2021 platypus and woylie genomes all contained complete immune gene family clusters, which were highly fragmented in the lower quality wombat, antechinus, and numbat genomes. Fragmentation was particularly evident within families which contain genes that do not share orthology to those in eutherians, such as LRC NK receptors and TCRμ, and highly duplicated families such as MHC (Figure 3).

To rule out species-specific differences in our direct assessment of assembly quality on immune gene annotation, we annotated a previous version of the platypus genome from 2018 (GCA_002966995.1) with Fgenesh++ to enable comparison with our Fgenesh++ annotation of the 2021 platypus genome (GCA_004115215.4) also generated as part of this study. Compared to the 2021 assembly, the 2018 platypus assembly was more fragmented given the 6-fold increase in the number of contigs, 14-fold increase in the number of scaffolds, and associated 2-fold decrease in contig N50 and 4-fold decrease in scaffold N50 between the two assemblies. Despite these metrics, the 2018 platypus assembly is still highly contiguous as it was generated using long-read data.

To investigate the relationship between immune gene fragmentation and genome quality further, we calculated the number of scaffolds which encoded 50% (L50) and 90% (L90) of manually annotated immune genes in each of the seven genomes from six species (Figure 2).

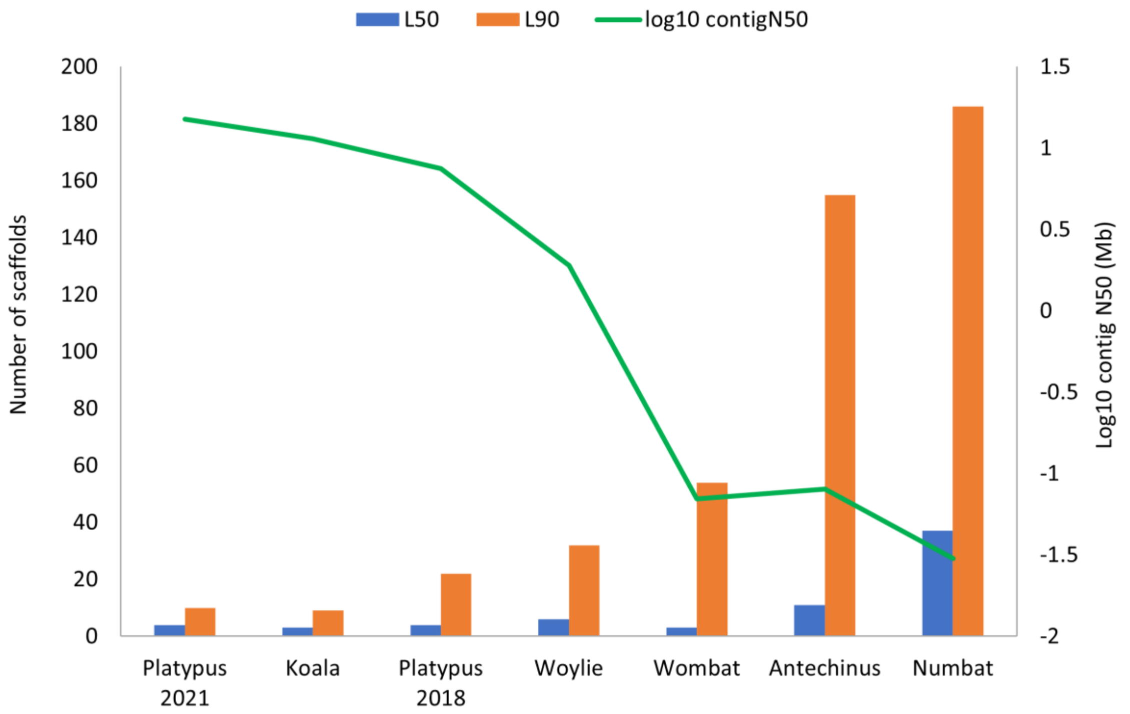

Figure 2. L50 and L90 immune gene metric for seven genomes from six species, compared to log_{10} contig N50.

The 2021 platypus, koala and woylie had an L90 of 10, 9 and 36 respectively, which suggests immune gene families were highly contiguous within all three genomes (Figure 2). Complete coding sequences were identified for 98% and 95% of immune genes in koala and woylie respectively. In addition, 90% of annotated immune genes were located on scaffolds greater than 33.3 Mbp, 75 Mbp and 1 Mbp in the 2021 platypus, koala, and woylie respectively. Complex multi-gene immune families such as MHC, NK receptors and TCR were highly intact in all three species. The koala and woylie MHC regions were both primarily located on a single scaffold (Figure 3). Class I and II genes were interspersed, and flanked by class III, framework and extended class I and II gene clusters, which reflected the MHC organisation of other marsupials (Figure 3) [18, 57]. Unlike marsupials, the platypus MHC is encoded within a pseudoautosomal region of two sex chromosomes. MHC class I and II genes were interspersed in a single cluster on chromosome X3, and class III, extended class I and II, and framework genes located in a single cluster on chromosome X5 (Figure 3) in the 2021 assembly [41]. Large gene expansions within the LRC NK receptors were encoded on a single scaffold in koala and six scaffolds in woylie (Figure 3). The number and type of monotreme NK receptor genes differs to marsupials, as they have a large expansion within the NKC gene cluster and reduction within the LRC gene cluster [72]. More than 80% of platypus NKC genes were located in a single cluster on chromosome 17, with LRC genes located on 5 different chromosomes in the 2021 assembly [72]. Fragmentation of the LRC cluster is not a factor of genome quality but reflects the evolutionary history of this immune family [72]. The four TCR loci (α/δ, β, γ and μ) were encoded in single clusters on three chromosomes in platypus 2021 assembly and single scaffolds in koala. The TCR loci were fragmented across up to three scaffolds in woylie. This includes genes known to flank these loci in other marsupials, which enabled resolution of TCR locus organisation in these species, and confirmed gene synteny across marsupials, human and mouse as identified previously [18].

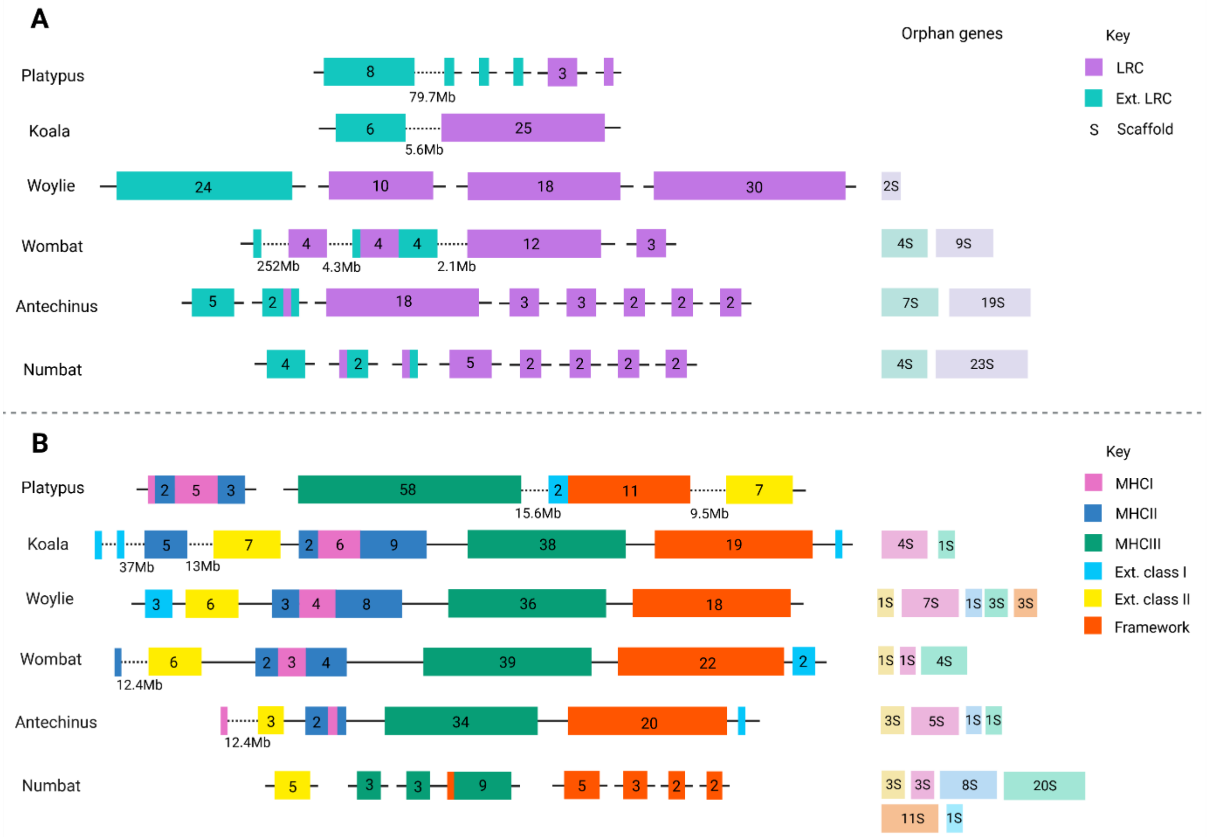

Figure 3. Genomic organisation and gene content of the LRC (A) and MHC region (B) in six genomes.

Figure 3 legend. The number of genes within each cluster are given, as well as scaffold counts of orphan genes (genes on single scaffolds). In A, LRC genes are purple, extended LRC genes are teal. In B, MHC class I genes are red, class II blue, class III green, extended class I pink, extended class II yellow and framework genes orange. Large distances between genes are given below the scaffold, otherwise the distance between genes and/or clusters was within the expected range for each family. Figure created with BioRender.com.

Fragmentation of immune genes in the wombat genome differed between immune families, with an L90 of 56 (Figure 2). 22% of scaffolds encoding immune genes were shorter than 100Kb and partial coding sequences were identified for 7% of annotated immune genes. The MHC region was relatively contiguous in the wombat, with 92% of genes encoded on a single scaffold (Figure 3). Although, a number of MHC genes were encoded as orphan genes to the main MHC cluster, indicating this family is misassembled in the wombat genome. In addition, some MHC genes could not be identified in the

wombat genome, while only single copies could be identified for others which are known to be duplicated in all other marsupials studied to date (Additional file 2). While this reduced MHC gene content in the wombat may reflect the true MHC gene repertoire of this species, it is likely MHC genes could not be annotated due to assembly error. The LRC cluster was highly fragmented across 16 scaffolds (Figure 3), of which more than 80% encoded a single gene and were less than 10kb in length. Extended LRC and LRC genes were interspersed, likely due to mis-assembly of the region as these genes should be located in separate clusters as observed in koala and woylie (Figure 3). TCRα, β and γ loci were encoded on individual scaffolds, however TCRμ was fragmented across 10 scaffolds, with 34% of genes located on individual scaffolds of less than 15Kb. While the TCRβ locus was encoded in a single cluster in the wombat, half of the locus was in the reverse orientation. This organisation is unusual amongst mammalian TCR and is likely a result of the HiC scaffolding error and not a true inversion.

Immune gene families were highly fragmented in the antechinus and numbat genomes, with an L90 of 156 and 218 respectively (Figure 2). 29% and 43% of immune genes were located on scaffolds less than 100Kb, and partial coding sequences were identified for 5.7% and 10.8% of immune genes, in antechinus and numbat respectively. Complex multi-gene families such as MHC, NK receptors and TCR were highly fragmented, with individual genes or exons located on short scaffolds. While 86% of MHC genes were located on a single scaffold in antechinus (Figure 3), genome fragmentation prevented the identification of additional MHC genes, hence the true MHC gene content could not be determined. The numbat MHC region was highly fragmented across 52 scaffolds, 63% of which were less than 100Kb in length (Figure 3). Large gene expansions of LRC NK receptors were fragmented across 34 scaffolds in antechinus and numbat, of which 67% (antechinus) and 35% (numbat) were less than 10Kb, and 76% of scaffolds encoded individual LRC genes in both species (Figure 3). Similar to wombat, extended LRC and LRC genes were interspersed, likely a mis-assembly as these genes should be encoded within separate clusters as observed in koala and woylie. All four TCR loci were fragmented in numbat, and all except TCRα in antechinus, with individual loci encoded across up to 6 scaffolds in

numbat and 19 in antechinus. Low contiguity within genomic regions encoding immune gene families in the antechinus and numbat limited investigation of genomic organisation, synteny and evolution in these species.

This relationship between genome quality and immune gene fragmentation is not an artefact of species-specific differences in immune gene repertoires. Comparison of manual immune gene annotations in the 2021 and 2018 platypus genome assemblies revealed similar patterns of immune gene fragmentation in the lower-quality 2018 assembly (Supplementary Figure 6 and 7). The 2018 platypus assembly had an L90 metric of 22, indicating immune gene clusters were intact within this genome but not to the extent of the 2021 assembly (L90 of 10) (Figure 2). In the 2018 assembly only 28% of NKC genes were encoded on a single scaffold (compared to 80% in the 2021 assembly), the MHC was encoded cross six scaffolds (compared to two in the 2021 assembly), and only 2 of the 4 TCR clusters were intact (all were intact in the 2021 assembly). Automated annotation of both assemblies with Fgenesh++, and comparison with our manual immune gene annotations, yielded the same result as presented for the five marsupial genomes: immune genes are poorly characterised by automated pipelines regardless of genome quality. In the 2021 and 2018 assemblies, a similar proportion of immune genes were correctly annotated (10% and 9% respectively) and not annotated (10% and 15% respectively) by Fgenesh++ (Supplementary Figure 6). As observed in the five marsupial genomes, TCR and IG were the most poorly annotated families by Fgenesh++ in both platypus assemblies (Supplementary Figure 7).

Discussion

By manually annotating immune genes in five marsupial genomes and two versions of the platypus genome, all varying qualities, we have confirmed that genome quality is directly linked to our ability to annotate complex immune gene families. Without long reads and scaffolding technologies, immune genes are scattered across many individual scaffolds and gene family organisation and evolution cannot be elucidated. We conclude that long-read data, with or without HiC technology, to generate

a high-quality genome assembly with a contig N50 of at least 1MB is required to investigate immunity and disease in wildlife. However, a kitchen sink approach to genome sequencing and assembly will enable complete reconstruction of complex and duplicated families such as MHC, TCR and LRC NK receptors as in the platypus 2021 and koala genomes.

The immune gene repertoire of the koala, woylie, wombat, antechinus and numbat was similar to other marsupials such as Tasmanian devil [46, 49, 53], tammar wallaby (*Macropus eugenii*) [74, 91-94] and grey short-tailed opossum (*Monodelphis domestica*) [82]. The platypus immune gene repertoire has been characterised previously [41], and we identified their location within both the 2021 and 2018 genome assemblies. Fewer MHC genes were identified in the wombat, antechinus, and numbat, compared to the platypus, koala, and woylie (Table 2, Supplementary Table S2 in Additional file 2). This is likely due to poor read assembly within this highly variable and duplicated region of the genome, rather than a true reduction in MHC gene content within these three species however, further investigation into the MHC gene repertoire of additional marsupial species is required. The assembly of a complete MHC cluster in the platypus, koala and woylie is due to the ability of long reads to span duplicated and variable sequences, which enables assembly algorithms to accurately reconstruct this complex region of the genome.

Automated annotation poorly characterises immune genes in non-model species

Despite mammalian BUSCO scores of up to 94.1% amongst the seven genomes in this study, indicating that the genomes were "functionally complete", on average 59% of immune genes were not accurately annotated (≤80% overlap) and 21% of genes were not annotated (0% overlap) by the automated software Fgenesh++ and MAKER, nor the NCBI pipeline, compared to our manual annotations (Figure 3). Aside from TCR and IG, the majority of immune genes incorrectly annotated or missing from the automated annotations were divergent genes not orthologous to those in eutherian mammals, such as MHC, marsupial-specific gene expansions within the LRC and monotreme-specific gene expansions within the NKC. Given their divergence, these genes often have low or no BLAST

homology to nucleotide or protein databases. Gene models generated by automated annotation software are hypotheses based on supporting evidence such as RNAseq data and homology to nucleotide and protein databases. While immune transcripts were identified in the transcriptomes from these species, RNAseq data only supported gene models for a low proportion of MHC, LRC and NKC genes. RNAseq data only supported 8-16% of LRC gene predictions and 16-37% of MHC gene predictions amongst the four marsupial genome annotations which used RNAseq data as gene model evidence (koala, woylie, antechinus and numbat). Similarly, around 60% of NKC genes within the platypus genomes were supported by RNAseq data. Overall, RNAseq data did not provide enough evidence to support gene models for ~20% of immune genes within the genome. Some immune genes may not have been expressed in the tissue sequenced, were expressed at low levels, or were fragmented. For human and mouse, comprehensive and curated gene sets such as GENCODE and RefSeq are available to guide gene model predictions, comprising data from more than 10,000 RNA experiments and decades of dedicated work in this field [95, 96]. Given time, budget and sample constraints for wildlife, these curated gene sets are not available, hence RNAseq evidence is incomplete resulting in deficient gene models by automated annotation software.

It is not surprising that TCR and IG V segments were poorly or not annotated by all automated pipelines used to annotate the genomes in this study. These genes are notoriously difficult to characterise and are manually annotated in the human and mouse genome on Ensembl using the International Immunogenetics Information System (IMGT) database [38, 97]. Alignment of mature IG and TCR sequences from RNAseq data to the genome results in poor automated annotation, as V segments utilize different sequence signal splice sites to introns, which are not recognized by the open reading frame prediction algorithms. Indeed, RNAseq evidence only supported 7% to 18% of TCR V segment and 0% to 6.9%% of IG V segment gene predictions by automated pipelines amongst the four marsupial and platypus genomes. V sequences from three marsupials and two monotremes are available in IMGT, however as non-model species, they are not included in the scope for manual annotation by Ensembl or NCBI, so these important functional features are not annotated.

Our results highlight the importance of manual annotation and curation of complex and variable immune genes, and caution reliance on BUSCO metrics to assess functional completeness of a genome. If this pattern is observed more widely across non-model species and other complex gene families, functionally important genes may not be accurately represented in genome annotations, which will flow on to downstream applications [36, 98]. While automated annotation is required to keep pace with the rapid sequencing of genome assemblies, manual gene characterisation is still the gold standard for genome annotation [95] and is conducted for the human, mouse, zebrafish and rat genomes on Ensembl [99]. For non-model species, manual annotation is conducted by individual research groups following genome assembly accession with NCBI or Ensembl, who conduct in-house automated annotation for some but not all species [100, 101]. These highly valuable manual gene annotations are not incorporated into the Ensembl annotation release but are often listed in the supplementary materials of multiple individual publications. NCBI does have some capacity to incorporate manual changes to existing annotation records [102]. Changes to multiple annotations, such as adding new genes as is the case in this study, require the genome to be re-annotated, which is not feasible for all research groups. Given NCBI and Ensembl annotations are widely used by the scientific community, these institutions should consider incorporating manual gene annotations into the annotation record or provide scope for permanently storing this valuable data alongside the respective assembly.

Genome quality correlates with immune gene fragmentation

As expected, we found that genome quality directly correlates with likelihood that an immune gene family was assembled and annotated correctly. Immune genes fragment as genome quality declines (Figure 2 and 3). This highlights the importance of long reads and HiC scaffolding to re-assemble complex gene families (platypus, koala, woylie), which are poorly assembled in short read and linked-read assemblies (wombat, antechinus, numbat). Figure 4 provides a graphical representation of the impact of different sequencing technologies on the assembly and fragmentation of immune gene clusters. When the average read or contig length is shorter than the gene length, the assembly

algorithm is unable to reconstruct genes, which are fragmented across multiple short contigs [98]. The average immune gene in this study was ~10 kbp in length. Long reads greater than 10 kbp in both platypus, koala and woylie genomes were able to span these genes, whereas the ~150 bp short reads in the wombat, antechinus and numbat genomes were insufficient to re-assemble the entire gene, resulting in gene fragments on short scaffolds. Gene families with copy number variation such as MHC and NK receptors are notoriously difficult to assemble and annotate [26, 29], so it is not surprising these gene families were highly fragmented in the antechinus and numbat genomes. Gene copies within these families can contain almost identical domains, may be pseudogenes and are encoded in clusters within the genome [36]. For example, koala NK LRC genes share up to 96% amino acid sequence identity and are encoded within a single cluster. For these reasons, assembly and annotation of MHC and NK receptors have been used to illustrate improvements in assembly quality. For example, MHC class I genes were located on a single contig in a recent release of the human genome [29], however the highly repetitive MHC class II locus remains unresolved [29].

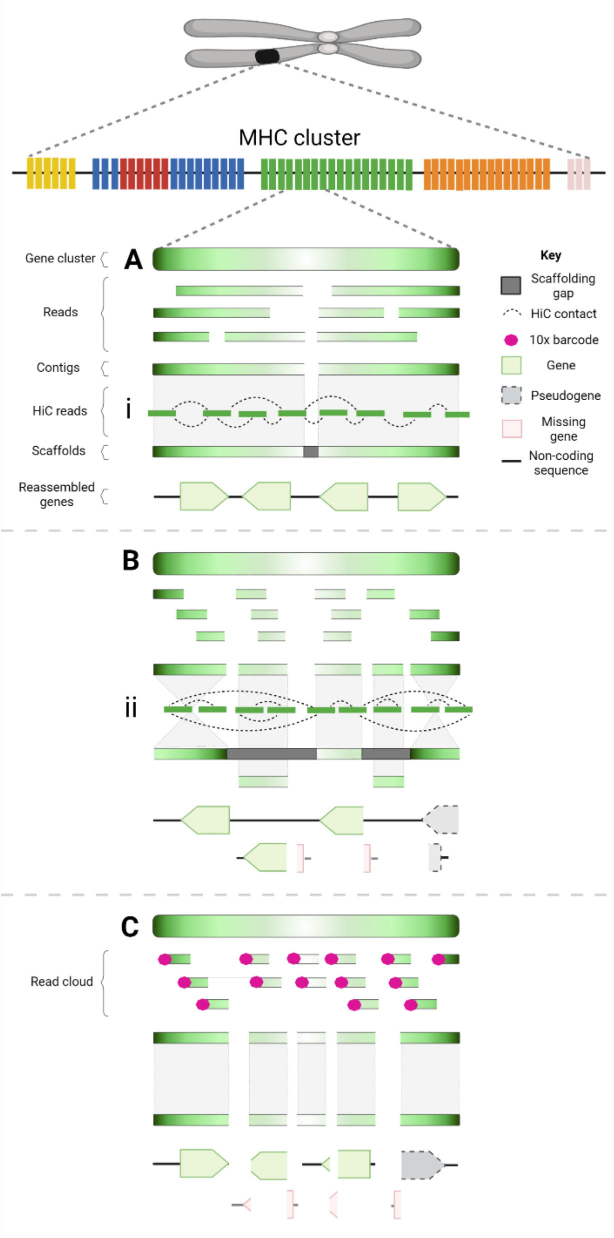

Figure 4. Impact of different sequencing technologies on the assembly of immune gene clusters such as the MHC.

Figure 4 legend. The impact of long-read (A – platypus, koala and woylie), short-read (B – wombat) and 10x Chromium linked read (C – antechinus and numbat) sequencing technologies, alone or in combination with HiC scaffolding (i – koala & platypus, and ii – wombat), on the assembly of complex and repetitive immune gene clusters such as the MHC. Colour gradient represents gene orientation (A) Long read sequencing generates reads which span complex and repetitive sequences, resulting in long contigs and scaffolds which contain multiple immune genes with complete coding sequences. (B)

Short-read sequencing generated reads which are unable to span immune genes, hence reads are assembled into multiple short contigs which end when the algorithm is unable to assemble a repetitive and complex immune gene sequence. (C) In linked-read sequencing, individual DNA molecules are partitioned into gel beads and identical barcodes attached, then sequenced using short-read technology resulting in read clouds [103]. As no individual read within the cloud spans the entire length of the DNA molecule, the algorithm is unable to assemble repetitive and complex sequences, resulting in multiple short contigs similar to a short-read assembly. Short contigs in B and C result in fragmentation of immune genes, leading to false pseudogenization and "missing" genes. (i) HiC sequencing provides contact information for DNA sequences located in close proximity within the nucleus, as frequency decreases with increasing linear distance within the genome assembly [104]. This contact information can be used to cluster, order and orient contigs into chromosome-size scaffolds [105]. Long contigs scaffolded with HiC result in near-complete reconstruction of immune gene clusters. (ii) Short contigs scaffolded with HiC generates what appears to be long scaffolds, however complex immune gene clusters are incomplete. As multiple HiC contacts can span the length of the contig, the correct contig orientation is not apparent leading to inversions and mis-placed contigs during scaffolding. This leads to incorrect orientation of genes, which can cause pseudogenization and/or gene fragmentation. Manual immune gene annotation reveals that the true gene complement of the immune cluster is not contained within the scaffolded sequence. Figure created with BioRender.com.

HiC scaffolding of contigs derived from platypus and koala long reads resulted in complete and accurate reassembly of immune gene clusters in both genomes (Figure 4A). Conversely, HiC scaffolding of contigs from wombat short reads resulted in immune gene fragmentation (Figure 4B), reflected in the high immune gene L90 for the wombat genome (Figure 2). Both the koala and wombat genomes were scaffolded with DNAzoo HiC data using the same 3D-DNA pipeline [63, 64, 106]. This result underscores the importance of assessing annotations when determining genome quality, as the wombat genome is classified as chromosome-length yet is highly fragmented within functionally

important genomic regions. Input genome assembly contiguity is known to influence HiC scaffolding ordering and orientation errors [107], despite claims that HiC scaffolding with 3D-DNA generates chromosome-length scaffolds from US$1,000 short read contigs [63]. Problems with HiC scaffolding within repetitive and duplicated regions are well documented [31, 107, 108], which is exacerbated by short contigs [107]. Modelling of human genome scaffolding performance using 3D-DNA revealed scaffold chimeras, ordering and orientation errors increased as contig length decreased [107]. While the koala and platypus genomes used as input to HiC scaffolding benefited from polishing with short read data and optical mapping [57], HiC scaffolding is insufficient to recover the majority of immune clusters from a fragmented genome.

The 3D-DNA pipeline orientates contigs within scaffolds by maximizing contact frequency between contig ends [64]. Short contigs, such as those from the wombat, would have multiple contacts that span the length of the contig. This means both true and false contig orientations would have a similar frequency, resulting in errors such as the partial inversion of the TCRB locus which is likely false (Additional file 2). At a gene level, these errors lead to the misplacement of genes on short scaffolds outside the main immune cluster and false pseudogenisation (Figure 4B). Long contigs, such as those from the koala, would have fewer contacts that span the length of the contig, hence the true orientation of the contig would be clear from the higher contact frequency at the correct joining end. The combination of long contigs which span repetitive and highly heterozygous regions with HiC scaffolding maximizes contiguity within immune gene clusters (Figure 4A).

10x Chromium linked-read sequencing was insufficient to accurately re-assemble immune gene clusters in our study (Figure 4C). While this technology is no longer available for genome sequencing, acknowledging the limitations of this technology for immune gene annotation remains valid in order to make use of existing 10x genomes. Complete marsupial immune gene clusters can span hundreds of kilobases to megabases, as shown by annotation of the complete MHC, NK receptor and TCR regions in the koala (Additional file 2). DNA molecules input to 10x library preparation were on average 74

kbp and 23 kbp in antechinus and numbat respectively. This molecule size only spanned smaller immune clusters in the antechinus, such as the 70 kbp TRG locus, but was insufficient to span any cluster in the numbat. Interestingly, the antechinus MHC cluster appears to be intact (Figure 3), however manual annotation revealed multiple genes were "missing" within the scaffold and instead were located on individual short scaffolds. Regardless of input DNA molecule length, 10x libraries are still subject to the limitations of short-read sequencing regarding assembly of complex sequences. Antechinus and numbat 10x libraries were sequenced as short ~150 bp reads, hence while reads can be assigned back to the corresponding input DNA molecule, no single read spans the molecule length. Gaps between the reads make *de novo* assembly of repetitive and complex immune sequences difficult, often resulting in termination of contig extension and gene fragments scattered across short scaffolds [109-111]. These gene fragments can be misinterpreted as pseudogenes owing to loss of up/downstream coding regions (Figure 4C). For example, antechinus and numbat NK LRC genes share up to 97% and 98% amino acid sequence identity amongst the genes identified in each species respectively. The LRC should be encoded within a single cluster, as in the koala genome (Figure 3). Instead, the antechinus and numbat LRC clusters are fragmented across 33 and 34 scaffolds respectively.

As the global biodiversity crisis deepens, the need to sequence eukaryotic life while it remains is imperative [1, 7, 8]. High quality genomes, using a combination of long-read and HiC, have recently been generated for a number of wildlife species [8], which have been used to answer questions involving chromosome evolution [112], comparative genomics [113] and runs of homozygosity [114] amongst others. Our results show that high-quality genomes are also necessary to study immune genes in wildlife.

Draft quality *de novo* genomes, in this study the antechinus and numbat (linked reads), have limited capacity for usefully informing immunogenetics studies as only partial sequences will be identified for most immune genes. A scaffold-quality genome, in this study the woylie and 2018 platypus assembly

(long-reads) or wombat (short-reads with HiC), would be suitable for immune marker development targeting most immune gene families, and studying TCR and IG diversity. Long-reads will provide contiguity within duplicated MHC and NK families, which should reassemble into complete clusters. HiC data may resolve some immune gene clusters from a short-read assembly, however, may introduce errors as discussed earlier. Finally, the kitchen sink approach, in this study the 2021 platypus and koala genomes (multiple data types), will accurately assemble immune gene clusters, which is essential for investigating genomic organisation, synteny and evolution. In the context of wildlife disease both sample availability and research dollars will dictate the type of data able to be generated for genome assembly, from this study we recommend a minimum of long-read sequencing such as PacBio HiFi to allow for complete annotation of immune gene regions

Potential implications

The biodiversity crisis and increasing impact of wildlife disease on animal and human health provides impetus for studying immune genes in wildlife. Genomes are now available for many wildlife species, however utility of these assemblies for annotating complex immune gene families is unknown. We have provided an assessment of complex immune gene annotation across genomes of varying quality, using immune genes in five marsupials and one monotreme as an example. Genome quality directly influenced the reassembly of immune gene clusters, and ability to investigate evolution, organisation, and true gene content of the immune repertoire. A high-quality genome generated from long-reads, with or without HiC, accurately assembles immune gene clusters. However, draft-quality genomes generated from short-reads with HiC, or the now obsolete 10x Chromium linked-reads, were unable to achieve this. Aside from genome quality, manual annotation of immune genes is required to cover the shortfall in deficient gene models used by automated annotation software. Our results highlight the limitations of different sequencing technologies and established workflows for genome annotation and quality assessment, when applied to non-model species and the investigation of wildlife disease and immunity.

Methods

Five published marsupial genomes, koala [57, 63, 64], woylie [65], wombat [63], antechinus [66] and numbat [67] (Table 1), and one monotreme genome, platypus [41], were selected for this study based on use of different sequencing technologies (alone and in combination) and variation in assembly quality. These include assemblies generated using multiple data types (koala and platypus), long and short-reads (woylie), short-reads and HiC (wombat) or 10x Chromium linked-reads (antechinus and numbat). BUSCO scores were generated by uploading the six genome assemblies to the Galaxy web platform [118], where the public server at galaxy.org was used to run BUSCOv5.3.2 [35] against the mammalian database.

Immune genes were annotated in the koala (phaCin_unsw_v4.1_HiC) [57, 63, 64], antechinus (anrechinusM_pseudohap2.1) [66], woylie (mBetpen1.pri.20210916) [65], wombat (vu-2k) [63, 64] and numbat genome (mMyrfas1.pri.20210917) [67] using multiple search strategies. BLAST was used to search genome assemblies, associated annotation files and/or transcriptomes using published marsupial, monotreme and eutherian immune gene sequences as queries, with default parameters and an e-value threshold of 10 so as not to exclude any potential gene candidates. HMMERv3.2 [119] was also used to identify putative genes within immune families that are known to contain duplications in other marsupials, such as NK receptors. Hidden markov models (HMM) were constructed using ClustalW alignments of published marsupial and eutherian immune gene sequences constructed in BioEditv7.2.5 [120], which were then used to search all genomes and transcriptomes using HMMER v3.2 with an e-value threshold of 10. For variable segments of T cell receptor and Immunoglobulin families, recombination signal sequences (RSS) downloaded from the IMGT database [97] and published koala sequences [57], were aligned using ClustalW in BioEditv7.2.5 [120] and used to construct HMM. These RSS HMM were then used to search each genome using HMMERv3.2 [119], to identify conserved RSS which flank each variable segment. For NK receptors, putative NKC and LRC sequences from BLAST+v2.7.1 [68] and HMMERv3.2 [119] searches were queried against the swissprot

nonredundant database, and any sequences with top hits to swissprot NK genes, marsupial-specific NK genes or the protein families database (Pfam) [121] immunoglobulin domain PF00047 or C-type lectin domain PF00059 HMM model were retained. IGSF domains within putative NK sequences from each species were identified using the simple modular architecture research tool (SMART) database [122], and IGSF domains within 5 kbp were considered exons of a single LRC gene. Putative immune genes were named following the appropriate nomenclature for each family, with duplicated genes named according to their genomic location from the 5' to 3' end of the locus. For each immune gene family, amino acid sequences from all five species, in addition to other marsupial, monotreme and eutherian sequences, were aligned using ClustalW in BioEditv7.2.5 [120]. This alignment was then used to construct neighbour-joining phylogenetic trees in MEGAXv10.2.4 [123] using the p-distance method, pairwise deletion and 1000 bootstrap replicates.

To investigate the impact of genome assembly quality on immune gene annotation, and discount species differences from our assessment, Fgenesh++ v7.2.2 [33] was used to annotate two different assemblies of the platypus genome; GCA_004115215.4 generated using multiple data types [41], and GCA_002966995.1 generated using only long and short-read data. In addition, Fgenesh++ v7.2.2 [33] was used to annotate the koala and wombat genome assemblies to investigate the influence of automated annotation method on immune gene annotation. To generate mRNA evidence for input to Fgenesh++, RNAseq data from 19 platypus tissues and 16 koala tissues accessioned with the NCBI sequence read archive (SRA) (Supplementary Table S3) was used to generate reference-guided global transcriptomes for each genome assembly (koala, platypus GCA_004115215.4 and GCA_002966995.1). No wombat RNAseq data was available on the SRA, hence a global transcriptome was not generated for this species. Briefly, raw RNAseq reads were quality and length trimmed using Trimmomatic v0.39 [124] with the following parameters: ILLUMINACLIP:TruSeq3-SE.fa:2:30:10 SLIDINGWINDOW:4:5 LEADING:5 TRAILING:5 MINLEN:25. Over 90.53% of paired trimmed reads were retained for all 35 datasets (Supplementary Table S3). Trimmed reads were then aligned to the respective species genome, and assembly version, using HISAT2 v2.1.0 [125] with default parameters.

Resulting sam files were converted to sorted bam files using SAMTOOLS v1.9 [126], then StringTie v2.1.6 [127] used to generate gtf files for each tissue. Tama merge [128] was then used to merge aligned reads for each tissue into a single global transcriptome for each genome assembly (koala, platypus GCA_004115215.4 and GCA_002966995.1), with a 5' threshold of 3 and a 3' threshold of 500. CPC2 [129] was used to determine the coding potential of each transcript, and Transdecoder v2.0.1 [130] to predict open reading frames within each transcript, for each global transcriptome.

The wombat, koala and two platypus genome assemblies (GCA_004115215.4 and GCA_002966995.1) were annotated using Fgenesh++ v7.2.2 with general mammalian parameters using a custom machine at the Pawsey Supercomputing Centre with 64 CPUs, 256GB RAM and 1TB of disk storage. An optimised gene-finding matrix from Tasmanian devils was used for koala and wombat genome annotations, while the platypus gene finding matrix was used for both platypus genome assembly annotations. Transcripts with the longest open reading frame for each predicted gene were extracted from the global transcriptomes for platypus and koala as outlined in the previous section and used as mRNA-based gene predictions. The compute wall-time required to complete each annotation was as follows: wombat 8 days, 1 hour and 15 minutes, koala 7 days, 8 hours and 38 minutes, platypus GCA_002966995.1 2 days 2 hours and 37 minutes and platypus GCA_004115215.4 1 day, 16 hours and 13 minutes.

Additional files

File name: Additional file 1

File format: .xls

Title of data: Supplementary Table S1

Description of data: Genomic coordinates of manually annotated immune genes in the koala, woylie, wombat, antechinus and numbat genomes. The genomic coordinates of published platypus immune genes used in this study are also included.

File name: Additional file 2

File format: .doc

Title of data: Supplementary results

Description of data: A comprehensive comparison of manually annotated immune genes in this study to those in other marsupials and humans is provided in Supplementary Table 2. For each immune gene family characterised in this study, a summary of results and phylogenetic analysis is provided. This includes genes encoding toll-like receptors, natural killer receptors, cytokines (interferons, interleukins and tumour necrosis factors), T cell receptor constant and variable regions (all five chains in marsupials and monotremes), immunoglobulin constant and variable regions (heavy and light chains) and major histocompatibility complex class I, II and III genes. Additional file 2 contains 7 tables and 14 figures.

Data availability

The published woylie and numbat genome and global transcriptome assemblies are available through Amazon Web Services Open Datasets Program https://registry.opendata.aws/australasian-genomics/, NCBI under BioProject accession PRJNA763700 and GigaDB for woylie and PRJNA786364 and GigaDB [131] for numbat. The published koala genome assembly and annotation (phaCin_unsw_v4.1_HiC.fasta) are available from the DNAzoo website https://www.dnazoo.org/assemblies/Phascolarctos_cinereus. The published wombat genome assembly and annotation (vu-2k.fasta) are also available from the DNAzoo website https://www.dnazoo.org/assemblies/Vombatus_ursinus. The published antechinus genome assembly and annotation (anrechinusM_pseudohap2.1.fasta) are available from NCBI under

BioProject accession PRJNA664282 and GigaDB [132], and published platypus genome assembly and annotation (mOrnAna1.pri.v4) under BioProject accession PRJNA489114. Genomic coordinates for all immune gene sequences annotated in this study are available in Additional file 1. Supporting information for this study is available in Additional file 2.

Declarations

List of abbreviations

Bacterial artificial chromosome (BAC), basic local alignment search tool (BLAST), benchmarking single copy gene orthologs (BUSCO), complementary DNA (cDNA), devil facial tumour disease (DFTD), giga-base-pair (Gpb), high-throughput chromosome conformation capture (HiC), hidden markov model (HMM), immunoglobulin (IG), immunoglobulin superfamily (IGSF), interferon (IFN), international immunogenetic information system (IMGT), kilo-base-pair (kbp), leukocyte receptor complex (LRC), major histocompatibility complex (MHC), mega-base-pair (Mbp), National Center for Biotechnology Information (NCBI), natural killer complex (NKC), natural killer receptor (NK), Pacific Biosciences (PacBio), protein families database (Pfam), recombination signal sequence (RSS), simple modular architecture research tool (SMART), single nucleotide polymorphisms (SNPs), T cell receptor (TCR) and toll-like receptor (TLR).

Consent for publication

Not applicable

Competing interests

The authors declare that they have no competing interests

Funding

This work has been funded by the Australian Research Council Centre of Excellence for Innovations in Peptide and Protein Science (CE200100012) and Discovery Project (DP180102465). LS was supported by LP180100244 and PB was supported by an Australian Postgraduate award. YZ is supported by the China Scholarship Council.

Authors' contributions

LS assembled and annotated the woylie genome and transcriptomes, PB assembled and annotated the numbat genome and transcriptomes, EP assisted with both. EP, PB, LS, YC and YZ annotated immune genes. KB, CJH and EP designed the study. EP drafted the manuscript, all authors read and commented on drafts of the manuscript and have approved the submission.

Acknowledgements

The authors would also like to acknowledge the generous contribution of the Presbyterian Ladies' College Sydney and Bioplatforms Australia. This work was supported by the Australian Fgenesh++ Service (biocommons.org.au/fgenesh-plus-plus) provided by the Australian BioCommons and the Pawsey Supercomputing Research Centre.

References

1. Diaz S, Settle J, Brondizio ES, Ngo HT, Gueze M, Agard J, et al. *IPBES (2019): Summary for policymakers of the global assessment report on biodiversity and ecosystem servies of the Intergovernmental Science-Policy Platform on Biodiversity and Ecosystem Services*. 2019. Bonn, Germany.
2. Scheele BC, Pasmans F, Skerratt LF, Berger L, Martel A, Beukema W, et al. Amphibian fungal panzootic causes catastrophic and ongoing loss of biodiversity. Science. 2019;363 6434:1459. doi:10.1126/science.aav0379.
3. Hoyt JR, Kilpatrick AM and Langwig KE. Ecology and impacts of white-nose syndrome on bats. Nature Reviews Microbiology. 2021;19 3:196-210. doi:10.1038/s41579-020-00493-5.
4. Woods GM, Lyons AB and Bettiol SS. A Devil of a Transmissible Cancer. Tropical Medicine and Infectious Disease. 2020;5 2:50.
5. Rohr JR, Civitello DJ, Halliday FW, Hudson PJ, Lafferty KD, Wood CL, et al. Towards common ground in the biodiversity–disease debate. Nature Ecology & Evolution. 2020;4 1:24-33. doi:10.1038/s41559-019-1060-6.
6. Hohenlohe PA, Funk WC and Rajora OP. Population genomics for wildlife conservation and management. Molecular Ecology. 2021;30 1:62-82. doi:https://doi.org/10.1111/mec.15720.
7. Lewin H, Robinson G, Kress WJ, Baker W, Coddington J, Crandall K, et al. Earth BioGenome Project: Sequencing life for the future of life. Proceedings of the National Academy of Sciences (PNAS). 2018;115 17:4325-33. doi:10.1073/pnas.1720115115.
8. Rhie A, McCarthy SA, Fedrigo O, Damas J, Formenti G, Koren S, et al. Towards complete and error-free genome assemblies of all vertebrate species. Nature. 2021;592 7856:737-46. doi:10.1038/s41586-021-03451-0.
9. Teeling EC, Vernes SC, Dávalos LM, Ray DA, Gilbert MTP and Myers E. Bat Biology, Genomes, and the Bat1K Project: To Generate Chromosome-Level Genomes for All Living Bat Species. Annual Review of Animal Biosciences. 2018;6 1:23-46. doi:10.1146/annurev-animal-022516-022811.
10. Zhang G. Bird sequencing project takes off. Nature. 2015;522 7554:34-. doi:10.1038/522034d.

11. Peterson BK, Weber JN, Kay EH, Fisher HS and Hoekstra HE. Double Digest RADseq: An Inexpensive Method for De Novo SNP Discovery and Genotyping in Model and Non-Model Species. PLOS ONE. 2012;7 5:e37135. doi:10.1371/journal.pone.0037135.
12. Sansaloni CP, Petroli CD, Carling J, Hudson CJ, Steane DA, Myburg AA, et al. A high-density Diversity Arrays Technology (DArT) microarray for genome-wide genotyping in Eucalyptus. Plant Methods. 2010;6 1:16. doi:10.1186/1746-4811-6-16.
13. McLennan EA, Grueber CE, Wise P, Belov K and Hogg CJ. Mixing genetically differentiated populations successfully boosts diversity of an endangered carnivore. Animal Conservation. 2020;23 6:700-12. doi:https://doi.org/10.1111/acv.12589.
14. Scally A, Yngvadottir B, Xue Y, Ayub Q, Durbin R and Tyler-Smith C. A Genome-Wide Survey of Genetic Variation in Gorillas Using Reduced Representation Sequencing. PLOS ONE. 2013;8 6:e65066. doi:10.1371/journal.pone.0065066.
15. Robledo-Ruiz DA, Pavlova A, Clarke RH, Magrath MJL, Quin B, Harrisson KA, et al. A novel framework for evaluating in situ breeding management strategies in endangered populations. Molecular Ecology Resources. 2021;n/a n/a doi:https://doi.org/10.1111/1755-0998.13476.
16. Lott MJ, Wright BR, Kemp LF, Johnson RN and Hogg CJ. Genetic Management of Captive and Reintroduced Bilby Populations. The Journal of Wildlife Management. 2020;84 1:20-32. doi:https://doi.org/10.1002/jwmg.21777.
17. Irving AT, Ahn M, Goh G, Anderson DE and Wang L-F. Lessons from the host defences of bats, a unique viral reservoir. Nature. 2021;589 7842:363-70. doi:10.1038/s41586-020-03128-0.
18. Parra ZE, Baker ML, Hathaway J, Lopez AM, Trujillo J, Sharp A, et al. Comparative genomic analysis and evolution of the T cell receptor loci in the opossum *Monodelphis domestica*. BMC Genomics. 2008;9 111:1-19.
19. Glusman G, Rowen L, Lee I, Boysen C, Roach JC, Smit AFA, et al. Comparative Genomics of the Human and Mouse T Cell Receptor Loci. Immunity. 2001;15 3:337-49. doi:https://doi.org/10.1016/S1074-7613(01)00200-X.
20. Sun Y, Wei Z, Li N and Zhao Y. A comparative overview of immunoglobulin genes and the generation of their diversity in tetrapods. Developmenal and Comparative Immunology. 2013;39:103-9.
21. Kelley J and Trowsdale J. Features of MHC and NK gene clusters. Transplant Immunology. 2005;14 3:129-34. doi:https://doi.org/10.1016/j.trim.2005.03.001.
22. Krasnec K, Sharp AR, Williams TL and Miller RD. The opossum MHC genomic region revisited. Immunogenetics. 2015;67:259-64.
23. Kelley J, Walfer L and Trowsdale J. Comparative genomics of natural killer cell receptor gene clusters. PLOS Genetics. 2005;1 2:129-39.
24. Takeda K, Kaisho T and Akira S. Toll-like receptors. Annual Review of Immunology. 2003;21 1.
25. Wong ESW, Papenfuss AT and Belov K. Genomic identification of chemokines and cytokines in opossum. Journal of Interferon and Cytokine Research. 2011;31 3:317-30.
26. Horton R, Wilming L, Rand V, Lovering RC, Bruford EA, Khodiyar VK, et al. Gene map of the extended human MHC. Nature Reviews Genetics. 2004;5 12:889-99. doi:10.1038/nrg1489.
27. Robinson J, Waller MJ, Parham P, Groot Nd, Bontrop R, Kennedy LJ, et al. IMGT/HLA and IMGT/MHC: sequence databases for the study of the major histocompatibility complex. Nucleic Acids Research. 2003;31 1:311-4. doi:10.1093/nar/gkg070.
28. Trowsdale J and Parham P. Mini-review: Defense strategies and immunity-related genes. European Journal of Immunology. 2004;34 1:7-17. doi:https://doi.org/10.1002/eji.200324693.
29. Jain M, Koren S, Miga KH, Quick J, Rand AC, Sasani TA, et al. Nanopore sequencing and assembly of a human genome with ultra-long reads. Nature Biotechnology. 2018;36 4:338-45. doi:10.1038/nbt.4060.

30. Ming L, Wang Z, Yi L, Batmunkh M, Liu T, Siren D, et al. Chromosome-level assembly of wild Bactrian camel genome reveals organization of immune gene loci. Molecular Ecology Resources. 2020;20 3:770-80. doi:https://doi.org/10.1111/1755-0998.13141.

31. Bickhart DM, Rosen BD, Koren S, Sayre BL, Hastie AR, Chan S, et al. Single-molecule sequencing and chromatin conformation capture enable de novo reference assembly of the domestic goat genome. Nature Genetics. 2017;49 4:643-50. doi:10.1038/ng.3802.

32. Holt C and Yandell M. MAKER2: an annotation pipeline and genome-database management tool for second-generation genome projects. BMC Bioinformatics. 2011;12 1:491. doi:10.1186/1471-2105-12-491.

33. Solovyev V, Kosarev P, Seledsov I and Vorobyev D. Automatic annotation of eukaryotic genes, pseudogenes and promoters. Genome Biology. 2006;7 Suppl 1:S10. doi:10.1186/gb-2006-7-s1-s10.

34. Rhie A, McCarthy SA, Fedrigo O, Damas J, Formenti G, Koren S, et al. Towards complete and error-free genome assemblies of all vertebrate species. Cold Spring Harbor Laboratory, 2020.

35. Seppey M, Manni M and Zdobnov EM. BUSCO: Assessing Genome Assembly and Annotation Completeness. In: Kollmar M, editor. Gene Prediction: Methods and Protocols. New York, NY: Springer New York; 2019. p. 227-45.

36. Mudge JM and Harrow J. The state of play in higher eukaryote gene annotation. Nature Reviews Genetics. 2016;17 12:758-72. doi:10.1038/nrg.2016.119.

37. Guigó R, Flicek P, Abril JF, Reymond A, Lagarde J, Denoeud F, et al. EGASP: the human ENCODE genome annotation assessment project. Genome Biology. 2006;7 Suppl 1:S2. doi:10.1186/gb-2006-7-s1-s2.

38. Ensembl: Annotation of immunoglobulin and T cell receptor genes. https://m.ensembl.org/info/genome/genebuild/ig_tcr.html (2021). Accessed 19th July 2021.

39. Hogg CJ, Ottewell K, Latch P, Rossetto M, Biggs J, Gilbert A, et al. Threatened Species Initiative: Empowering conservation action using genomic resources. Proceedings of the National Academy of Sciences. 2022;119 4:e2115643118. doi:10.1073/pnas.2115643118.

40. Gordon D, Huddleston J, Chaisson Mark JP, Hill Christopher M, Kronenberg Zev N, Munson Katherine M, et al. Long-read sequence assembly of the gorilla genome. Science. 2016;352 6281:aae0344. doi:10.1126/science.aae0344.

41. Zhou Y, Shearwin-Whyatt L, Li J, Song Z, Hayakawa T, Stevens D, et al. Platypus and echidna genomes reveal mammalian biology and evolution. Nature. 2021; doi:10.1038/s41586-020-03039-0.

42. Quigley BL and Timms P. The Koala Immune Response to Chlamydial Infection and Vaccine Development—Advancing Our Immunological Understanding. Animals. 2021;11 2:380.

43. Madden D, Whaite A, Jones E, Belov K, Timms P and Polkinghorne A. Koala immunology and infectious diseases: How much can the koala bear? Developmental & Comparative Immunology. 2018;82:177-85. doi:https://doi.org/10.1016/j.dci.2018.01.017.

44. Peel E and Belov K. Lessons learnt from the Tasmanian devil facial tumour regarding immune function in cancer. Mammalian Genome. 2018; doi:10.1007/s00335-018-9782-3.

45. Murchison EP, Schulz-Trieglaff OB, Ning Z, Alexandrow LB, Bauer MJ, Fu B, et al. Genome sequencing and analysis of the Tasmanian devil and its transmissible cancer. Cell. 2012;148:780-91.

46. Morris B, Cheng Y, Warren W, Papenfuss AT and Belov K. Identification and analysis of divergent immune gene families within the Tasmanian devil genome. BMC Genomics. 2015;16 1.

47. Murchison EP, Tovar C, Hsu AL, Bender HS, Kheradpour P, Rebbeck CA, et al. The Tasmanian devil transcriptome reveals schwann cell origins in a clonally transmissible cancer. Science. 2010;327:84-7.

48. Hewavisenti RV, Morris KM, O'Meally D, Cheng Y, Papenfuss AT and Belov K. The identification of immune genes in the milk transcriptome of the Tasmanian devil (*Sarcophilus harrisii*). PeerJ. 2016;4:1569.
49. Cheng Y, Stuart A, Morris K, Taylor R, Siddle HV, Deakin JE, et al. Antigen-presenting genes and genomic copy number variations in the Tasmanian devil MHC. BMC Genomics. 2012;13:87.
50. Morris KM, Wright B, Grueber CE, Hogg C and Belov K. Lack of genetic diversity across diverse immune genes in an endangered mammal, the Tasmanian devil (*Sarcophilus harrisii*). Molecular Ecology. 2015;24:3860-72.
51. Siddle HV, Kreiss A, Eldridge MDB, Noonan E, Clarke CJ, Pyecroft S, et al. Transmission of a fatal clonal tumor by biting occurs due to depleted MHC diversity in a threatened carnivorous marsupial. PNAS. 2007;104 41:16221-6.
52. Siddle HV, Kreiss A, Tovar C, Yuen CK, Chen Y, Belov K, et al. Reversible epigenetic down-regulation of MHC molecules by devil facial tumour disease illustrates immune escape by a contagious cancer. PNAS. 2013;110 13:5103-8.
53. Siddle HV, Sanderson CE and Belov K. Characterization of major histocompatability complex class I and II genes from the Tasmanian devil (*Sarcophilus harrisii*). Immunogenetics. 2007;59:753-60.
54. Cheng Y, Sanderson CE, Jones M and Belov K. Low MHC class II diversity in the Tasmanian devil. Immunogenetics. 2012;64:525-33.
55. Cheng Y, Grueber C, Hogg CJ and Belov K. Improved high-throughput MHC typing for non-model species using long-read sequencing. Molecular Ecology Resources. 2021;n/a n/a doi:https://doi.org/10.1111/1755-0998.13511.
56. Quigley BL and Timms P. Helping koalas battle disease – Recent advances in Chlamydia and koala retrovirus (KoRV) disease understanding and treatment in koalas. FEMS Microbiology Reviews. 2020; doi:10.1093/femsre/fuaa024.
57. Johnson RN, O'Meally D, Chen Z, Etherington GJ, Ho SYW, Nash WJ, et al. Adaptation and conservation insights from the koala genome. Nature Genetics. 2018;50 8:1102-11. doi:10.1038/s41588-018-0153-5.
58. Morris K, Prentis PJ, O'Meally D, Pavasovic A, Brown AT, Timms P, et al. The koala immunological toolkit: sequence identification and comparison of key markers of the koala (*Phascolarctos cinereus*) immune response. Australian Journal of Zoology. 2014;62:195-9.
59. Morris KM, Matthew M, Waugh C, Ujvari B, Timms P, Polkinghorne A, et al. Identification, characterisation and expression analysis of natural killer receptor genes in *Chlamydia pecorum* infected koalas (*Phascolarctos cinereus*). BMC Genomics. 2015;16 1.
60. Morris KM, O'Meally D, Zaw T, Song X, Gillett A, Molloy MP, et al. Characterisation of the immune compounds in koala milk using a combined transcriptomic and proteomic approach. Scientific Reports. 2016;6:e35011.
61. Lau Q, Griffith JE and Higgins DP. Identification of MHCII variants associated with chlamydial disease in the koala (*Phascolarctos cinereus*). PeerJ. 2014;2:443.
62. Robbins A, Hanger J, Jelocnik M, Quigley BL and Timms P. Koala immunogenetics and chlamydial strain type are more directly involved in chlamydial disease progression in koalas from two south east Queensland koala populations than koala retrovirus subtypes. Scientific Reports. 2020;10 1:15013. doi:10.1038/s41598-020-72050-2.
63. Dudchenko O, Shamim MS, Batra SS, Durand NC, Musial NT, Mostofa R, et al. The Juicebox Assembly Tools module facilitates *de novo* assembly of mammalian genomes with chromosome-length scaffolds for under $1000. bioRxiv. 2018:254797. doi:10.1101/254797.
64. Dudchenko O, Batra SS, Omer AD, Nyquist SK, Hoeger M, Durand NC, et al. De novo assembly of the *Aedes aegypti* genome using Hi-C yields chromosome-length scaffolds. Science advances. 2017;356:92-5.

65. Peel E, Silver L, Brandies P, Hogg CJ and Belov K. A reference genome for the critically endangered woylie, *Bettongia penicillata ogilbyi*. Gigabyte. 2021;1 doi:https://doi.org/10.46471/gigabyte.35.
66. Brandies PA, Tang S, Johnson RSP, Hogg CJ and Belov K. The first *Antechinus* reference genome provides a resource for investigating the genetic basis of semelparity and age-related neuropathologies. Gigabyte. 2020;2020:0. doi:10.46471/gigabyte.7.
67. Peel E, Silver L, Brandies PA, Hayakawa T, Belov K and Hogg CJ. Genome assembly of the numbat (*Myrmecobius fasciatus*), the only termitivorous marsupial. Gigabyte. 2022; doi:https://doi.org/10.46471/gigabyte.47.
68. Camacho C, Coulouris G, Avagyan V, Ma N, Papadopoulos J, Bealer K, et al. BLAST+: architecture and applications. BMC bioinformatics. 2009;10 421.
69. Eddy SR. A probabilistic model of local sequence alignment that simplifies statistical significance estimation. PLOS Computational Biology. 2008;4 5:e1000069.
70. Belov K, Lam MKP, Hellman L and Colgan DJ. Evolution of the major histocompataibility complex: isolation of the class II beta cDNAs from two monotremes, the platypus and the short-beaked echidna. Immunogenetics. 2003;55:402-11.
71. Belov K and Hellman L. Immunoglobulin genetics of *Ornithorhynchus anatinus* (platypus) and *Tachyglossus aculeatus* (short-beaked echidna). Comparative Biochemistry and Physiology. 2003;136:811-9.
72. Wong ESW, Sanderson CE, Deakin JE, Whittington CM, Papenfuss AT and Belov K. Identification of natural killer cell receptor clusters in the platypus genome reveals an expansion of C-type lectin genes. Immunogenetics. 2009;61:565-79.
73. Dohm JC, Tsend-Ayush E, Reinhardt R, Grutzner F and Himmelbauer H. Distribution and pseudoautosomal localization of the major histocompatability complex in monotremes. Genome Biology. 2007;8:R175-R.16.
74. Papenfuss AT, Feng Z, Krasnec K, Deakin JE, Baker ML and Miller RD. Marsupials and monotremes possess a novel family of MHC class I genes that is lost from the eutherian lineage. BMC Genomics. 2015;16:535.
75. Nowak MA, Parra ZE, Hellman L and Miller RD. The complexity of expressed kappa light chains in egg-laying mammals. Immunogenetics. 2004;56:555-63.
76. Johansson J, Aveskogh M, Munday BL and Hellman L. Heavy chain V region diversity in the duck-billed platypus (*Ornithorhynchus anatinus*): long and highly variable complementary-determining region 3 compensates for limited germline diversity. The Journal of Immunology. 2002;168:5155-62.
77. Johansson J, Salazar JN, Aveskogh M, Munday B, Miller RD and Hellman L. High variability in complementarity-determining regions compensates for a low number of V gene families in the λ light chain locus of the platypus. European Journal of Immunology. 2005;35 10:3008-19. doi:https://doi.org/10.1002/eji.200425574.
78. Parra ZE, Arnold T, Nowak MA, Hellman L and Miller RD. TCR gamma chain diversity in the splen of the duckbill platypus (*Ornithorhynchus anatinus*). Developmenal and Comparative Immunology. 2006;30:699-71.
79. Parra ZE, Lillie M and Miller RD. A model for the evolution of the mammalian T-cell receptor alpha/delta and mu loci based on evidence from the duckbill platypus. Molecular Biology and Evolution. 2012;29 10:3205-14.
80. Wang X, Parra ZE and Miller RD. Platypus TCRmu provides insight into the origins and evolution of a uniquely mammalian TCR locus. The Journal of Immunology. 2011;187:5246-54.
81. Wong ESW, Papenfuss AT, Miller RD and Belov K. Hatching time for monotreme immunology. Australian Journal of Zoology. 2006;57:185-98.

82. Belov K, Sanderson CE, Deakin JE, Wong ESW, Assange D, McColl KA, et al. Characterization of the opossum immune genome provides insight into the evolution of the mammalian immune system. Genome Research. 2007;17:982-91.
83. Lau Q, Jobbins SE, Belov K and Higgins DP. Characterisation of four major histocompatibility complex class II genes of the koala (*Phascolarctos cinereus*). Immunogenetics. 2013;65:37-46.
84. Jobbins SE, Sanderson CE, Griffith JE, Krockenberger MB, Belov K and Higgins DP. Diversity of MHC class II *DAB1* in the koala (*Phascolarctos cinereus*). Australian Journal of Zoology. 2012;60 1:1-9. doi:https://doi.org/10.1071/ZO12013.
85. Cheng Y, Polkinghorne A, Gillett A, Jones EA, O'Meally D, Timms P, et al. Characterisation of MHC class I genes in the koala. Immunogenetics. 2018;70:125-33.
86. Matthew M, Beagley KW, Timms P and Polkinghorne A. preliminary characterisation of tumour necrosis factor alpha and interleukin-10 responses to *Chlamydia pecorum* infection in the koala (*Phascolarctos cinereus*). PLOS one. 2013;8 3:e59958.
87. Matthew M, Pavasovic A, Prentis PJ, Beagley KW, Timms P and Polkinghorne A. Molecular characterisation and expression analysis of Interferon gamma in response to natural *Chlamydia* infection in the koala, *Phascolarctos cinereus*. Gene. 2013;527:570-7.
88. Mathew M, Waugh C, Beagley KW, Timms P and Polkinghorne A. Interleukin 17A is an immune marker for chlamydial disease severity and pathogenesis in the koala (Phascolarctos cinereus). Developmental & Comparative Immunology. 2014;46 2:423-9. doi:http://dx.doi.org/10.1016/j.dci.2014.05.015.
89. Maher IE, Griffith JE, Lau Q, Reeves T and Higgins DP. Expression profiles of the immune genes CD4, CD8 beta, IFN gamma, IL-4, IL-6 and IL-10 in mitogen-stimulated koala lymphocytes (*Phascolarctos cinereus*) by qRT-PCR. PeerJ. 2014;2.
90. Miller RD. Those other mammals: The immunoglobulins and T cell receptors of marsupials and monotremes. Seminars in Immunology. 2010;22:3-9.
91. Siddle HV, Deakin JE, Coggill P, Whilming LG, Harrow J, Kaufman J, et al. The tammar wallaby major histocompatability complex shows evidence of past genomic instability. BMC Genomics. 2011;12:421.
92. Cheng Y, Siddle HV, Beck S, Eldridge MDB and Belov K. High levels of genetic variation at MHC class II DBB loci in the tammar wallaby (*Macropus eugenii*). Immunogenetics. 2009;61:111-8.
93. Zuccolotto P, Harrison GA and Deane EM. Cloning of marsupial T cell receptor alpha and beta constant region cDNAs. Immunology and Cell Biology. 2000;78 2:103-9.
94. Daly KA, Digby M, Lefevre C, Mailer S, Thomson P, Nicholas KR, et al. Analysis of the expression of immunoglobulins throughout lactation suggests two periods of immune transfer in the tammar wallaby (*Macropus eugenii*). Veterinary Immunology and Immunopathology. 2007;120:187-200.
95. Frankish A, Diekhans M, Ferreira A-M, Johnson R, Jungreis I, Loveland J, et al. GENCODE reference annotation for the human and mouse genomes. Nucleic Acids Research. 2018;47 D1:D766-D73. doi:10.1093/nar/gky955.
96. Pertea M, Shumate A, Pertea G, Varabyou A, Breitwieser FP, Chang Y-C, et al. CHESS: a new human gene catalog curated from thousands of large-scale RNA sequencing experiments reveals extensive transcriptional noise. Genome Biology. 2018;19 1:208. doi:10.1186/s13059-018-1590-2.
97. Lefranc M-P, Giudicelli V, Duroux P, Jabado-Michaloud J, Folch G, Aouinti S, et al. IMGT®, the international ImMunoGeneTics information system® 25 years on. Nucleic Acids Research. 2015;43 D1:D413-D22. doi:10.1093/nar/gku1056.
98. Salzberg SL. Next-generation genome annotation: we still struggle to get it right. Genome Biology. 2019;20 1:92. doi:10.1186/s13059-019-1715-2.

99. Ensembl: Manual gene annotation by Havana. https://m.ensembl.org/info/genome/genebuild/manual_havana.html (2021). Accessed 19th July 2021.
100. National Center for Biotechnology Information: The NCBI Eukaryotic Genome Annotation Pipeline. https://www.ncbi.nlm.nih.gov/genome/annotation_euk/process/ (2021). Accessed 19th July 2021.
101. Ensembl: Gene annotation in Ensembl. https://m.ensembl.org/info/genome/genebuild/index.html (2021). Accessed 19th July 2021.
102. National Center for Biotechnology Information: Updating information on GenBank genome records. https://www.ncbi.nlm.nih.gov/genbank/wgs_update/ (2021). Accessed 4th August 2021.
103. Weisenfeld NI, Kumar V, Shah P, Church DM and Jaffe DB. Direct determination of diploid genome sequences. Genome Research. 2017;27 5:757-67. doi:10.1101/gr.214874.116.
104. Lieberman-Aiden E, van Berkum NL, Williams L, Imakaev M, Ragoczy T, Telling A, et al. Comprehensive Mapping of Long-Range Interactions Reveals Folding Principles of the Human Genome. Science. 2009;326 5950:289. doi:10.1126/science.1181369.
105. Luo J, Wei Y, Lyu M, Wu Z, Liu X, Luo H, et al. A comprehensive review of scaffolding methods in genome assembly. Briefings in Bioinformatics. 2021; doi:10.1093/bib/bbab033.
106. Durand NC, Robinson JT, Shamim MD, Machol I, Mesirov JP, Lander ES, et al. Juicebox provides a visualisation system for Hi-C contact maps with unlimited zoom. Cell Systems. 2016;3:99-101.
107. Ghurye J, Rhie A, Walenz BP, Schmitt A, Selvaraj S, Pop M, et al. Integrating Hi-C links with assembly graphs for chromosome-scale assembly. PLOS Computational Biology. 2019;15 8:e1007273. doi:10.1371/journal.pcbi.1007273.
108. Burton JN, Adey A, Patwardhan RP, Qiu R, Kitzman JO and Shendure J. Chromosome-scale scaffolding of de novo genome assemblies based on chromatin interactions. Nature Biotechnology. 2013;31 12:1119-25. doi:10.1038/nbt.2727.
109. Marks P, Garcia S, Barrio AM, Belhocine K, Bernate J, Bharadwaj R, et al. Resolving the full spectrum of human genome variation using Linked-Reads. Genome Research. 2019;29 4:635-45. doi:10.1101/gr.234443.118.
110. Ho SS, Urban AE and Mills RE. Structural variation in the sequencing era. Nature Reviews Genetics. 2020;21 3:171-89. doi:10.1038/s41576-019-0180-9.
111. Ott A, Schnable JC, Cheng-Ting Y, Wu L, Liu C, Heng-Cheng H, et al. Linked read technology for assembling large complex and polyploid genomes. BMC Genomics. 2018;19 doi:http://dx.doi.org/10.1186/s12864-018-5040-z.
112. Damas J, Corbo M and Lewin HA. Vertebrate Chromosome Evolution. Annual Review of Animal Biosciences. 2021;9 1:1-27. doi:10.1146/annurev-animal-020518-114924.
113. Zoonomia Consortium. A comparative genomics multitool for scientific discovery and conservation. Nature. 2020;587 7833:240-5. doi:10.1038/s41586-020-2876-6.
114. Ceballos FC, Joshi PK, Clark DW, Ramsay M and Wilson JF. Runs of homozygosity: windows into population history and trait architecture. Nature Reviews Genetics. 2018;19 4:220-34. doi:10.1038/nrg.2017.109.
115. Genome 10K Community of Scientists. Genome 10K: a proposal to obtain whole-genome sequence for 10 000 vertebrate species. Journal of Heredity. 2009;100 6:659-74.
116. Kingan SB, Heaton H, Cudini J, Lambert CC, Baybayan P, Galvin BD, et al. A High-Quality De novo Genome Assembly from a Single Mosquito Using PacBio Sequencing. Genes. 2019;10 1:62. doi:10.3390/genes10010062.
117. Lawniczak M, Blaxter M, Johnson WE, Pettersson OV, Barker K and The Sample Collection and Processing Subcommittee. *Report on sample collection and processing standards*. March 2021 2021. Earth Biogenomne Project,.

118. Afgan E, Baker D, Batut B, van den Beek M, Bouvier D, Čech M, et al. The Galaxy platform for accessible, reproducible and collaborative biomedical analyses: 2018 update. Nucleic Acids Research. 2018;46 W1:W537-W44. doi:10.1093/nar/gky379.
119. Mistry J, Finn RD, Eddy SR, Bateman A and Punta M. Challenges in homology search: HMMER3 and convergent evolution of coiled-coil regions. Nucleic Acids Research. 2013;41 12:e121-e. doi:10.1093/nar/gkt263.
120. Hall T. BioEdit v7.2.2 ed.: Ibis Biosciences, 2013.
121. Mistry J, Chuguransky S, Williams L, Qureshi M, Salazar Gustavo A, Sonnhammer ELL, et al. Pfam: The protein families database in 2021. Nucleic Acids Research. 2020;49 D1:D412-D9. doi:10.1093/nar/gkaa913.
122. Letunic I, Khedkar S and Bork P. SMART: recent updates, new developments and status in 2020. Nucleic Acids Research. 2020;49 D1:D458-D60. doi:10.1093/nar/gkaa937.
123. Kumar S, Stecher G, Li M, Knyaz C and Tamura K. MEGA X: Molecular Evolutionary Genetics Analysis across Computing Platforms. Molecular biology and evolution. 2018;35 6:1547-9. doi:10.1093/molbev/msy096.
124. Bolger AM, Lohse M and Usadel B. Trimmomatic: a flexible trimmer for Illumina sequence data. Bioinformatics. 2014;30 15:2114-20.
125. Kim D, Paggi JM, Park C, Bennett C and Salzberg SL. Graph-based genome alignment and genotyping with HISAT2 and HISAT-genotype. Nature Biotechnology. 2019;37 8:907-15. doi:10.1038/s41587-019-0201-4.
126. Danecek P, Bonfield JK, Liddle J, Marshall J, Ohan V, Pollard MO, et al. Twelve years of SAMtools and BCFtools. GigaScience. 2021;10 2:giab008. doi:10.1093/gigascience/giab008.
127. Kovaka S, Zimin AV, Pertea GM, Razaghi R, Salzberg SL and Pertea M. Transcriptome assembly from long-read RNA-seq alignments with StringTie2. Genome Biology. 2019;20 1:278. doi:10.1186/s13059-019-1910-1.
128. Kuo RI, Cheng Y, Zhang R, Brown JWS, Smith J, Archibald AL, et al. Illuminating the dark side of the human transcriptome with long read transcript sequencing. BMC Genomics. 2020;21 1:751. doi:10.1186/s12864-020-07123-7.
129. Kang Y-J, Yang D-C, Kong L, Hou M, Meng Y-Q, Wei L, et al. CPC2: a fast and accurate coding potential calculator based on sequence intrinsic features. Nucleic Acids Research. 2017;45 W1:W12-W6. doi:10.1093/nar/gkx428.
130. Bryant DM, Johnson KM, DiTommaso T, Tickle T, Couger MB, Payzin-Dogru D, et al. A tissue-mapped axolotl de novo transcriptome enables identification of limb regeneration factors. Cell Reports. 2017;18:762-76.
131. Peel E, Silver L, Brandies PA, Hayakawa T, Belov K and Hogg CJ. Supporting data for "Genome assembly of the numbat (*Myrmecobius fasciatus*), the only termitivorous marsupial. GigaDB. 2022; doi:http://dx.doi.org/10.5524/100999.
132. Peel E, Silver L, Brandies PA, Hogg CJ and Belov K. Supporting data for "A reference genome for the critically endanfered woylie, *Bettongia penicillata ogilbyi*". GigaDB. 2021; doi:http://dx.doi.org/10.5524/100951.

Click here to access/download;Figure;Peel_Fig1.tiff

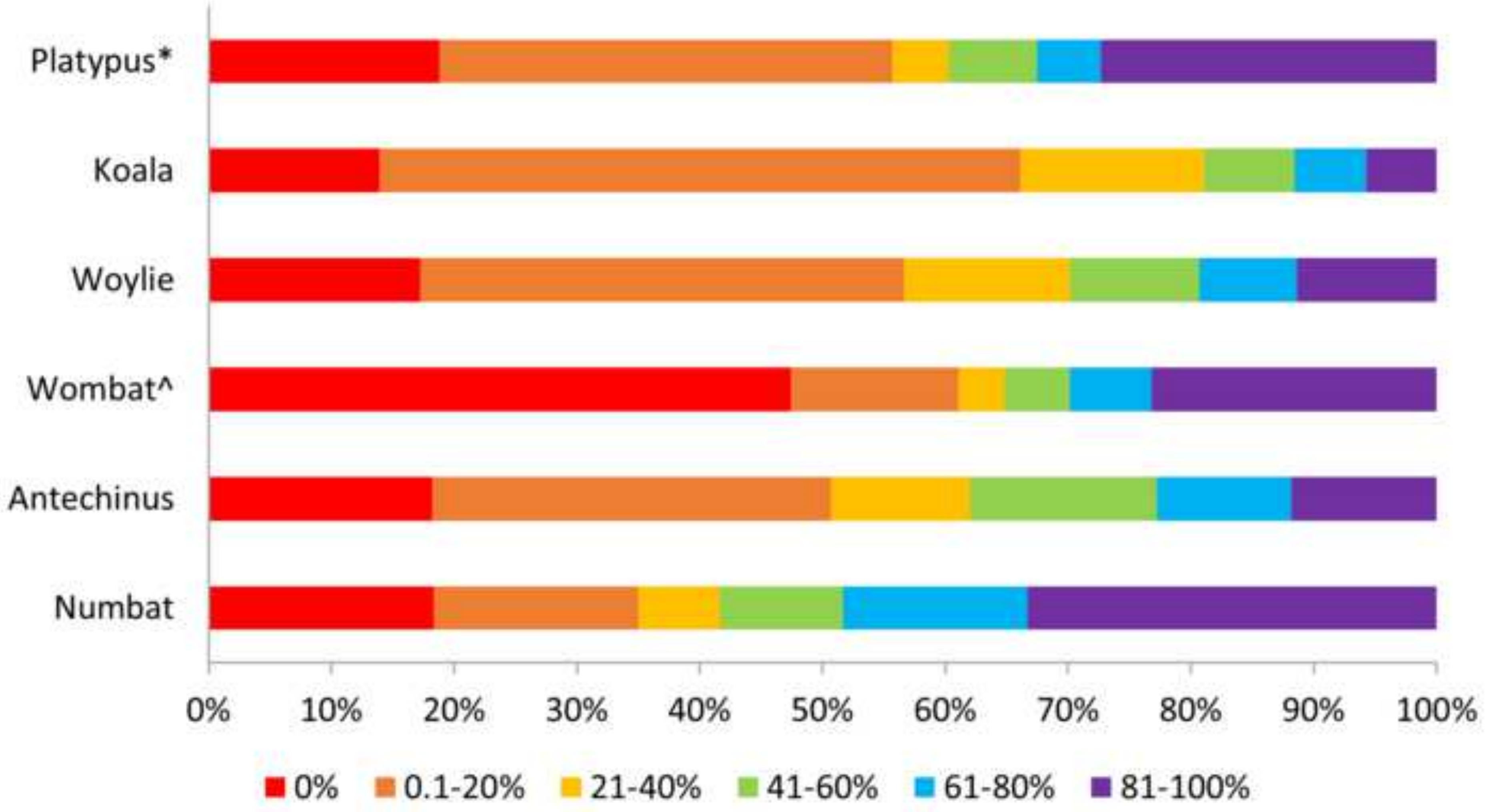

 Click here to access/download;Figure;Peel_Fig2.tiff

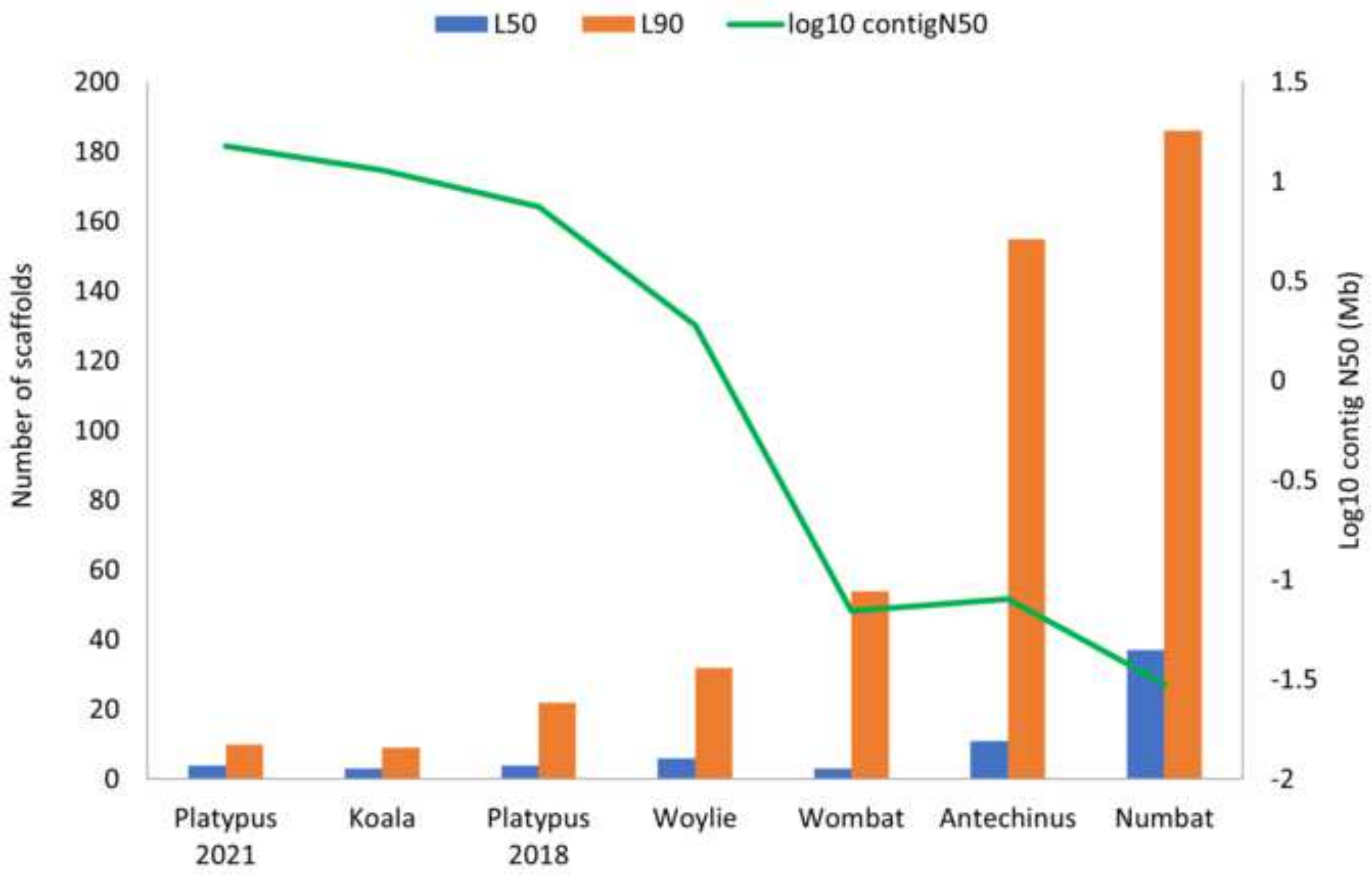

Figure3 Click here to access/download;Figure;Peel_Fig3.tiff

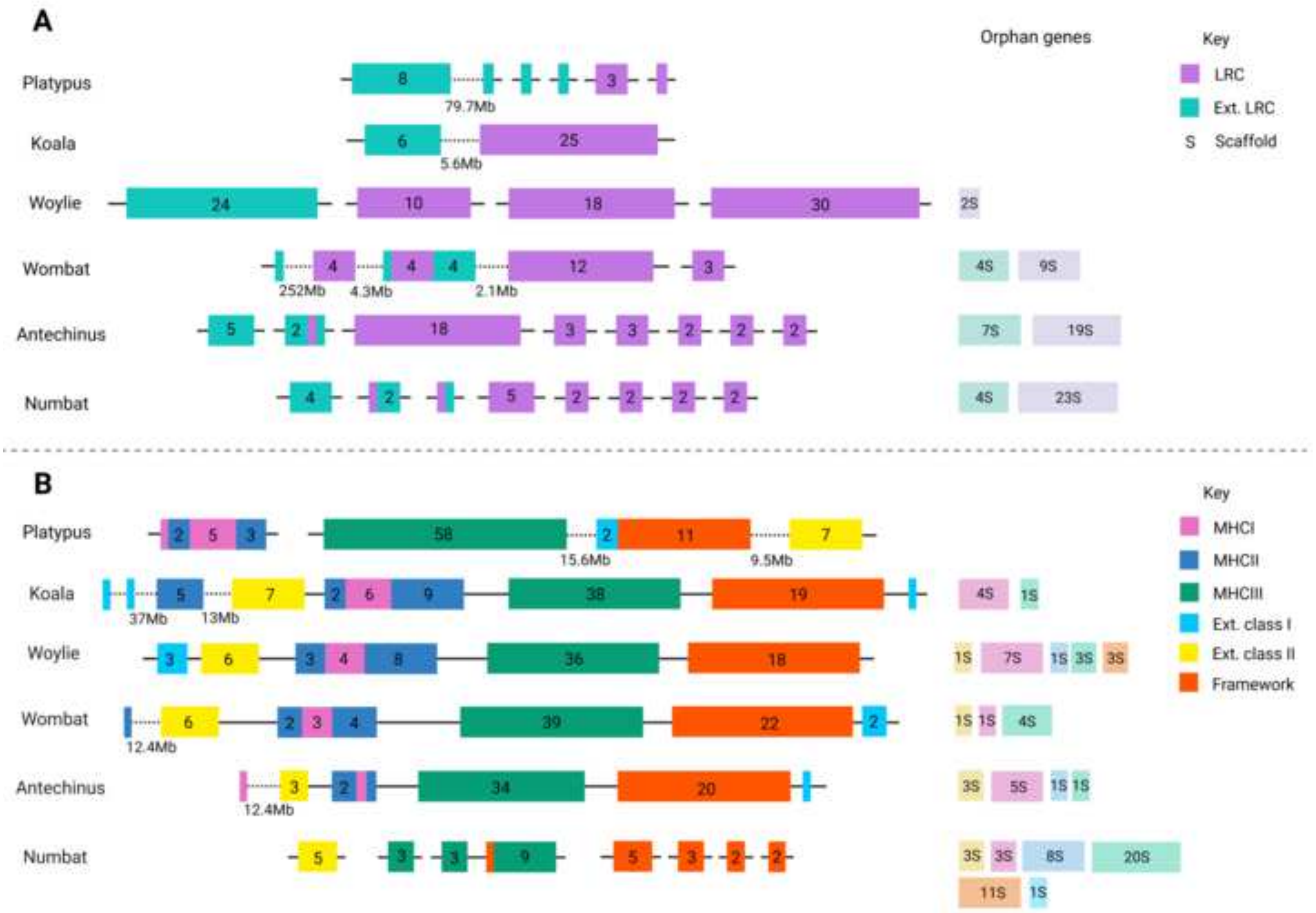

49 Spadina Ave. Suite 200
Toronto ON M5V 2J1 Canada
www.biorender.com

Confirmation of Publication and Licensing Rights

May 12th, 2022
Science Suite Inc.

Subscription:	*Postdoc Plan*
Agreement number:	*FG23WNFBN3*
Journal name:	*Gigascience*

To whom this may concern,

This document is to confirm that Emma Peel has been granted a license to use the BioRender content, including icons, templates and other original artwork, appearing in the attached completed graphic pursuant to BioRender's Academic License Terms. This license permits BioRender content to be sublicensed for use in journal publications.

All rights and ownership of BioRender content are reserved by BioRender. All completed graphics must be accompanied by the following citation: "Created with BioRender.com".

BioRender content included in the completed graphic is not licensed for any commercial uses beyond publication in a journal. For any commercial use of this figure, users may, if allowed, recreate it in BioRender under an Industry BioRender Plan.

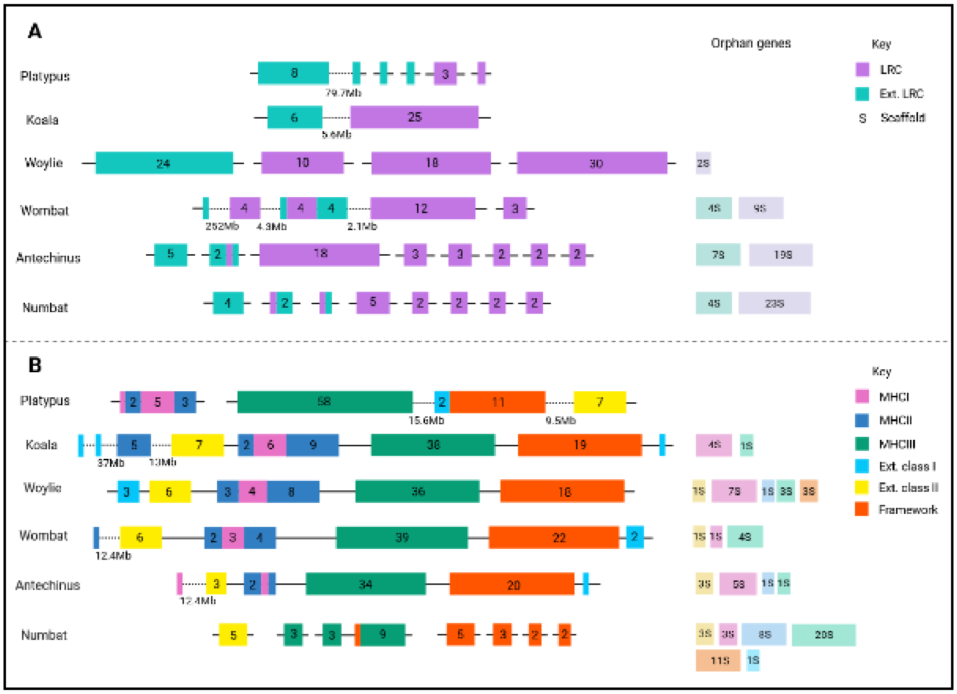

For any questions regarding this document, or other questions about publishing with BioRender refer to our BioRender Publication Guide, or contact BioRender Support at support@biorender.com.

Figure4

Click here to access/download;Figure;Peel_Fig4.tiff

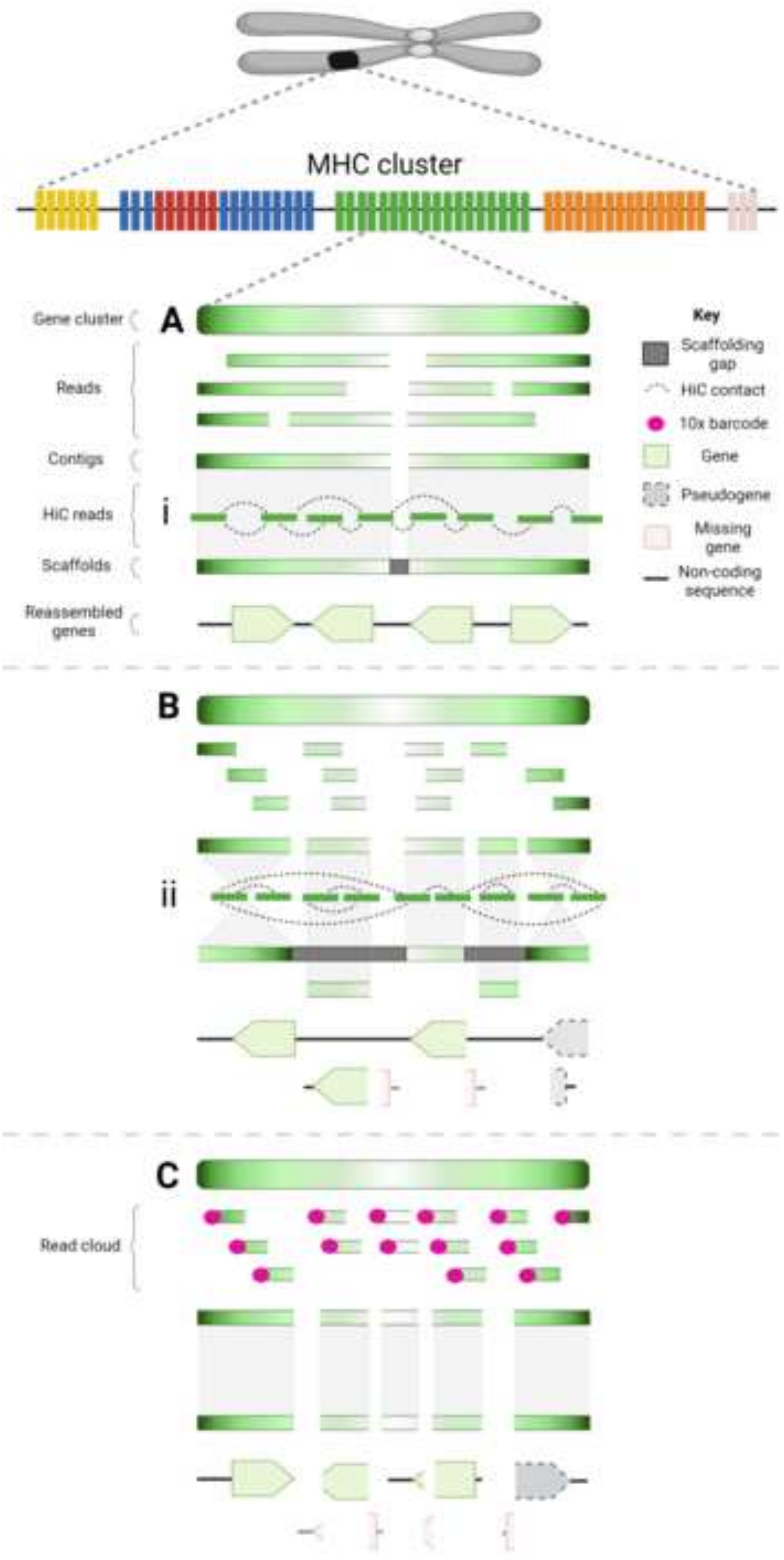

Click here to access/download
Supplementary Material
Additional file 1_amended.xlsx

Click here to access/download
Supplementary Material
Additional file 2_amended.docx

Click here to access/download;Personal Cover;Response to reviewers.docx

Response to reviewers

Reviewer #1:

Knowledge about immune genes is critical for species conservation programs. However, immune genes occur in large gene clusters that are difficult to assemble and annotate. This important and timely study uses a number of marsupial genomes and the platypus to assess which sequencing technologies enable complete reconstructions of immune gene clusters and which methods enable annotations of these immune genes.

I have the following comments.

Since Fgenesh++ and Maker produce automatic annotations, I wonder why not all 6 genomes were annotated with these two methods? This would allow a comparison between Fgenesh++ against Maker. Maybe it is possible to annotate at least a few genomes with both methods.

All six genomes were not annotated using Fgenesh++ and Maker as the authors wanted to utilise existing annotations available for 5 of the 6 genomes in our study (all except koala). The authors agree that re-annotating all genomes with both Fgenesh++ and Maker would enable a direct comparison between the two methods. However, determining the best automated annotation software for immune gene annotation was not the focus of this study, but rather the impact of assembly quality on immune gene annotation. A secondary aim of the paper was to investigate whether automated annotation software was able to accurately identify immune genes, compared to our manual annotations. While it is widely known within the field of wildlife immunogenetics that automated genome annotations fail to correctly characterise immune genes, to date there are no publications which quantitatively assess this observation.

The computation required to annotate all six genomes using both Fgenesh++ and MAKER was not feasible within the given three-month timeframe provided for changes to the manuscript. As such, the koala, wombat and 2021 platypus genomes have been annotated with Fgenesh++ which will enable investigation of how this popular annotation software performs for immune gene annotation within all genome assemblies of varying quality included in this study (woylie, antechinus and numbat were already annotated with Fgenesh++). The methods, results and figure 1 have been modified to reflect this. See lines 213-218, 261-273 of the results and below. Additional supplementary figures have been generated in response to the reviewer's comment. See supplementary figure 3, 4 and 5 in Additional file 2.

Table 1 has been modified to also include all genome annotations used in this study. This includes existing published annotations by NCBI, MAKER and Fgenesh++, as well as Fgenesh++ annotations conducted as part of this study.

Lines 213-218

"We assessed how well our manual immune gene annotation aligned with automated annotations by Fgenesh++ (2018 platypus, woylie, koala, antechinus, numbat and wombat), MAKER (wombat) and the NCBI pipeline (2021 platypus). Inclusion of the 2021 platypus NCBI and wombat MAKER annotations ensures that any differences in automated and manual immune gene annotation were not due to deficiencies within the Fgenesh++ annotation pipeline, as the woylie, antechinus and numbat genomes were all annotated with Fgenesh++ using the same parameters."

Lines 263-275

"This pattern of poor immune gene annotation was not an artefact of inherent differences between automated annotation pipelines amongst the six genomes (NCBI, MAKER and Fgenesh++) nor genome quality, as similar patterns were observed for Fgenesh++ annotations of the 2021 platypus and wombat genome generated as part of this study (Supplementary Figure 3, Supplementary Figure 4). Generally, the Fgenesh++ annotation resulted in fewer correctly annotated immune genes (≥90% overlap) compared to NCBI (2021 platypus) or MAKER (wombat) (Supplementary Figure 3). Although, the proportion of missing immune genes (0% overlap) was higher in the NCBI (2021 platypus) and MAKER (wombat) annotation than the Fgenesh++ annotation of both species genomes. As with NCBI and MAKER, Fgenesh++ poorly annotated TCR and IG families at the gene-level (Supplementary Figure 4) in the high-quality platypus and low-quality wombat. Correct annotations were somewhat recovered at the exon-level in both genomes (Supplementary Figure 5), although, the number of missing TCR and IG exons in the Fgenesh++ annotation was almost half that of NCBI and MAKER in platypus and wombat respectively."

Direct assessments of assembly quality should ideally be done on different assemblies of the same species to rule out real differences between species. Would it be possible to include previous koala or platypus genome that was much more fragmented?

The authors agree that multiple versions of the same genome assembly would enable direct assessment of assembly quality on immune gene annotation. As such, the authors have annotated the latest 2021 version of the platypus genome assembly published by Zhou et al 2021 (NCBI ID GCA_004115215.4) and the previous 2018 version (GCA_002966995.1) with Fgenesh++. Platypus was selected as the species for this comparison over koala (the only other species in our study with multiple genome assemblies available) as the improvement in assembly metrics between the 2021 and 2018 platypus genome assemblies is more significant than the 2018 and 2020 koala genome assemblies. This is due to the addition of numerous data types to the 2021 platypus assembly since the 2018 version. Genome assembly metrics for the 2018 platypus genome have been added to Table 1. The results section "Relationship between genome quality and manual immune gene annotation" has been modified to include a comparison between the 2018 and 2021 platypus assemblies. Specifically, see lines 283-290 and below. Figure 2 has also been updated to include the 2018 platypus genome assembly.

Fgenesh++ was selected for automated annotation of the two platypus assemblies over other methods such as MAKER as this would enable direct comparison of Fgenesh++ performance across all genomes in this study.

Lines 285-292

"To rule out species-specific differences in our direct assessment of assembly quality on immune gene annotation, we annotated a previous version of the platypus genome from 2018 (GCA_002966995.1) with Fgenesh++ to enable comparison with our Fgenesh++ annotation of the 2021 platypus genome (GCA_004115215.4) also generated as part of this study. Compared to the 2021 assembly, the 2018 platypus assembly was more fragmented given the 6-fold increase in the number of contigs, 14-fold increase in the number of scaffolds, and associated 2-fold decrease in contig N50 and 4-fold decrease in scaffold N50 between the two assemblies. Despite these metrics, the 2018 platypus assembly is still highly contiguous as it was generated using long-read data."

Figure 1 shows a useful of all immune genes. However, some genes like TLRs are actually easy to annotate as they are have a standard gene structure. Therefore, it would be informative to provide in this figure a breakdown of how well the different immune gene families are annotates, as the authors nicely did in table 2. This would inform on which immune genes are particularly difficult to annotate.

A breakdown of Figure 1 by immune family is now presented in Supplementary Figure 1 of Additional File 2. A breakdown of annotation at the exon-level by immune family has been added as Supplementary Figure 2. See lines 235-255 and below. Similar breakdown of this analysis by immune family have also been added for Fgenesh++ versus MAKER (wombat) or NCBI (2021 platypus) annotations of the platypus and wombat genome assemblies at the gene level for all seven families (Supplementary Figure 4), in addition to exon-level for TCR and IG families (Supplementary figure 5).

Lines 235-255

"A breakdown of this analysis by immune family revealed that marsupial- and monotreme-specific immune genes which are not orthologous to those in eutherians were generally poorly annotated, regardless of automated pipeline or genome quality (Supplementary Figure 1). This was particularly the case for TCR and IG gene families, with up to 88% of genes in these families incorrectly annotated by automated pipelines (≤10% overlap) amongst the six species (Table 2). This is likely due to highly duplicated variable gene segments that do not encode conventional exon-intron splice sites which may hinder annotation with automated pipelines. Poor gene annotations of TCR and IG families was somewhat recovered at the exon level, as some TCR and IG variable gene segments were annotated as exons by automated pipelines. Correct annotation (≥90% overlap) of the TCR family increased from 0-2% at the gene level to 2-15% at the exon level amongst the six genomes (Supplementary Figure 2). This improvement was even greater for the IG family, with an increase from 0-2% correct annotation at the gene level to 15-43% at the exon level amongst the six genomes (Supplementary Figure 2). Despite this, up to 67% of TCR and IG variable segments were still not annotated at the exon level (0% overlap) amongst the six genomes, highlighting the difficulty in automated annotation of these regions. Similarly, marsupial-specific gene expansions within the leukocyte receptor complex (LRC) and monotreme-specific gene expansions within the natural killer complex (NKC) family of NK receptors were also poorly annotated by automated pipelines (Supplementary Figure 1). As with TCR and IG families, correct annotation increased from the gene- (0-28% marsupial LRC, 31% platypus NKC) to exon-level (6-65% marsupial LRC, 79% platypus NKC) (Table 2, Supplementary Figure 2), likely due to the presence of variable numbers of duplicated immunoglobulin superfamily (IGSF) domains and C-type lectin (CLEC) domains within each LRC and NKC gene respectively."

Figure 3B is not colorblind friendly.

Colours in Figure 3B have been amended according to the colourblind friendly palette outlined in Wong, B. Points of view: Color blindness. *Nat Methods* **8**, 441 (2011). https://doi.org/10.1038/nmeth.1618

Line 275: The discussion makes it clear that this is a scaffolding error and not a real inversion. This should be clarified here as well.

This has been clarified in the text, see lines 345-347 and below.

"This organisation is unusual amongst mammalian TCR and is likely a result of the HiC scaffolding error and not a true inversion."

I fully agree with the value of the manual annotations. Therefore, it would be helpful to provide the manual annotations also as a gff3 or gtf file that provide the full exon structure. Additional file 2 only lists the start and end coordinates of genes with multiple exons. The assembly accession should also be listed.

Additional file 1 (previously Additional file 2) has been amended to include both the gene and exon coordinates for all immune genes across the 7 genome assemblies.

As a suggestion: A haplotype-resolved assembly of a marsupial is likely not yet available, but such an assembly would provide an opportunity to further investigate the influence of assembly quality and haplotype variation in immune genes.

The authors agree with the reviewer's comment. However, a haplotype-resolved assembly for marsupials will be challenging to generate given current recommendations include the use of trios to completely resolve paternal and maternal haplotypes. Samples from trios are incredibly difficult to obtain for wildlife such as marsupials given the opportunistic nature of most sample collection. This would be especially difficult for marsupials which are threatened or endangered, or are not currently housed in captivity.

Reviewer #2:

In this work, Peel and collaborators asses the accuracy of immune gene annotation in marsupial species by comparing the outcome of manually and automated annotation approaches. This allowed them to conclude that sequence data type and assembly quality determine the accuracy of gene annotation. I find the study interesting, although I have some general comments. I find that both the introduction and discussion sections would benefit from some re-structuring. Both sections are a bit long, with some repetitions. Also, the discussion section contains material from results. I would also appreciate more detailed figure legends.

The authors thank reviewer 2 for their comments. In light of no specific changes provided by reviewer 2, and changes already made to both the discussion and introduction for reviewer 1 and 3, we took no further action.

Reviewer #3:

In this manuscript, Peel et al examine the impact of assembly quality and sequencing/assembly method on the ability to annotate complex genes of the immune system, using a case study the five marsupial genomes and one monotreme genome of varying quality. While the conclusions the authors present are not particularly surprising given what we know about genome assembly, this manuscript does a nice job outlining the reasons why higher quality (in particular, long-read) assemblies are important to facilitate annotation of these critical genes, and exploring in depth the impact of various aspects of assembly quality. The authors present their results in a convincing and clear way, and this work provides a useful summary for the genomics community.

I do have some minor comments that I hope will help improve this work, listed below.

1. The conditional "in wildlife" is perhaps a little confusing in the title, as I believe the issues the authors raise should be widely relevant to vertebrate, or at least mammalian, genomes, and

"wildlife" is a term with varying colloquial definitions among the readership of Gigascience. Relatedly the discussion in the background section of the abstract, as well as the intro of the manuscript and some parts of the discussion, could probably focus on mammals generally, or even vertebrates, not wildlife specifically. It would also make sense to make the implicit vertebrate focus explicit.

The authors agree that the issues raised in our manuscript would be applicable to many mammalian or vertebrate genomes. However, genomics projects for non-model species such as wildlife generally work within constraints that are not always applicable to mammalian or vertebrate genomes more broadly. These include budget considerations, access to samples (remote locations, permits, CITES listing, threat status) and sample quantity (volume and tissue types available, sample quality (opportunistic sampling, non-invasive samples, sub-optimal preservation method, no access to liquid nitrogen or -80 freezer), amongst many others. All these factors influence the type of genome sequencing available to wildlife genomics projects, and hence resulting assembly quality. Mammals and many vertebrates more broadly, do not generally face these multitude of challenges when generating reference genomes. While the link between input sample, assembly quality and curation to generate a high-quality assembly has been established in wildlife (Rhie et al 2021), what has not been assessed is the impact of assembly quality on functionally important regions of the genome, such as immune genes. Our aim was to provide guidance for the wildlife genomics community, particularly those working on species impacted by disease, on how different genome sequencing strategies impact quality of immune gene annotations.

2. The introduction goes into extensive detail about the case study systems presented here - perhaps more detail than is really needed (e.g., lines 130 - 136 on DFTD and chlamydial vaccines). However, there is little background information about the specific immune gene families that are the focus of this work. The authors present a compelling argument for why studying these gene families is important, but some additional information to help guide readers who may not be expert in the specific immune families under discussion would be valuable. In particular, reminding readers why these genes in particular are such a challenge to annotate, with perhaps a brief overview of the six immune gene families that are the focus of the work.

Additional detail regarding the six immune gene families that are the focus of the manuscript, and why immune genes are challenging to annotate has been provided in the introduction at lines 66-101 and below for easy reference.

"The COVID-19 pandemic is one of many examples which highlight the ever-increasing importance of understanding wildlife immunity and disease to better understand and manage disease spill over [17]. In the case of wildlife threatened by disease, conservation questions are more challenging to answer and typically involve immunogenetic diversity which relies on accurate immune gene annotations. Immune genes in mammals can be classified into six major families based on their evolutionary history and function: T cell receptors (TCR), immunoglobulins (IG), major histocompatibility complex (MHC), natural killer (NK) receptors, toll-like receptors (TLR) and cytokines. Mammals utilise two antigen recognition systems: TCR and IG expressed by T lymphocytes and B lymphocytes respectively. TCR and IG are encoded in large clusters within the genome, each of which contain few constant sequences that define the receptor sub-type, and multiple highly duplicated variable segments that recognise and bind antigens. The number and sequence polymorphism of IG and TCR V segments varies significantly between mammalian species [18-20]. Another major family of immune genes is the major histocompatibility complex which contains three classes of genes (class I, II and III). MHC class I and II genes encode cell-surface receptors which bind and present self- and pathogen-derived antigens to T

lymphocytes, activating the adaptive immune response. Class I and II genes evolve via duplication and can be highly polymorphic, hence gene number differs between species [21, 22]. Natural killer (NK) cells directly kill virus-infected and cancerous cells and are an important component of innate immunity. Their activity is mediated via cell-surface receptors encoded by genes classified into two functionally similar but structurally dissimilar families; the leukocyte receptor complex (LRC) and natural killer complex (NKC). These families are encoded in separate clusters within the genome, and as they evolve via gene duplication, gene number varies significantly between species [23]. TLRs are membrane-spanning receptors expressed by immune and non-immune cells which bind pathogen-associated molecular patterns (PAMP), activating the innate and adaptive immune response. Compared to other immune genes, TLRs gene number and sequence is relatively conserved across mammals [24]. Lastly, cytokines are small proteins secreted by numerous cell types which direct the immune response. Cytokines can be classified into multiple families including interferons (IFN), tumour necrosis factors (TNF) and interleukins (IL), and gene content within each family varies between mammals [25].

Immune genes are some of the most polymorphic regions of the genome, owing to the need to generate diversity in response to ever-changing pathogenic pressures [26, 27]. Diversity within these gene families is generated through gene duplication, gene copy number variation, SNPs and rapid evolution, resulting in a complex genomic organisation and high level of pseudogenization [26]. Generally, immune genes are encoded within repetitive clusters in the genome, especially highly duplicated families such as the MHC and NK receptors [28]. Given these factors, accurate assembly and annotation of genomic regions encoding immune genes can be challenging [29-31], especially in wildlife."

3. I would recommend ordering the species in Table 1, Table 2, Figure 1, Figure 2, and Figure 3 in a consistent order, perhaps from highest to lowest contig N50. This will help readers keep track of the key patterns.

Ordering of species and immune families in figures and tables (except for table 1) in the main manuscript and Additional file 2 is now consistent with the reviewer's suggestion. Species are presented in the order of platypus, koala, woylie, wombat, antechinus then numbat, and immune families are presented in the order of cytokines, TLR, MHC, NKC, LRC, IG and TCR.

4. The authors present a qualitative assessment of the kinds of genes where automated annotation fails in lines 202-212 and Fig 3. However a quantitative breakdown here would also I think be useful to the community, and should be easy to generate. One could simply list the fraction of manually annotated genes correctly recovered (and completely missed with <10% overlap) for each class in Table 2 for each species. This would also allow the authors to put some numbers alongside statements in this paragraph like "Most of these genes comprised... [line 210]"

≥90% and ≤10% overlap in genomic coordinates between manual and automated annotation of immune genes has been added for each species and immune family in table 2. A quanitative breakdown has been added to this section of the results. See lines 235-255 and below. The authors have also added additional detail regarding automated versus manual immune annotation at the exon-level for the TCR, IG and LRC families which were poorly annotated by automated pipelines at the gene-level.

Lines 235-255

"A breakdown of this analysis by immune family revealed that marsupial- and monotreme-specific immune genes which are not orthologous to those in eutherians were generally poorly annotated, regardless of automated pipeline or genome quality (Supplementary Figure 1). This was particularly the case for TCR and IG gene families, with up to 88% of genes in these families incorrectly annotated by automated pipelines (≤10% overlap) amongst the six species (Table 2). This is likely due to highly duplicated variable gene segments that don't encode conventional exon-intron splice sites which may hinder annotation with automated pipelines. Poor gene annotations of TCR and IG families was somewhat recovered at the exon level, as some TCR and IG variable gene segments were annotated as exons by automated pipelines. Correct annotation (≥90% overlap) of the TCR family increased from 0-2% at the gene level to 2-15% at the exon level amongst the six genomes (Supplementary Figure 2). This improvement was even greater for the IG family, with an increase from 0-2% correct annotation at the gene level to 15-43% at the exon level amongst the six genomes (Supplementary Figure 2). Despite this, up to 67% of TCR and IG variable segments were still not annotated at the exon level (0% overlap) amongst the six genomes, highlighting the difficulty in automated annotation of these regions. Similarly, marsupial-specific gene expansions within the leukocyte receptor complex (LRC) and monotreme-specific gene expansions within the natural killer complex (NKC) family of NK receptors were also poorly annotated by automated pipelines (Supplementary Figure 1). As with TCR and IG families, correct annotation increased from the gene- (0-28% marsupial LRC, 31% platypus NKC) to exon-level (6-65% marsupial LRC, 79% platypus NKC) (Table 2, Supplementary Figure 2), likely due to the presence of variable numbers of duplicated immunoglobulin superfamily (IGSF) domains and C-type lectin (CLEC) domains within each LRC and NKC gene respectively."

5. I am not sure the statement (298-299): "that a kitchen sink approach, that uses long-read data combined with HiC technology, to generate a high-quality genome assembly is required to investigate immunity and disease in wildlife" is fully supported by the results the authors present. The annotation of the woylie genome, which as I understand it does not include any HiC scaffolding, seems to be as good or nearly as good as the two kitchen sink genomes. I would propose that the key conclusion is that long-read data specifically (with or without HiC) and high contig N50 (probably at least 1 Mb) is what is required for a successful manual annotation of these complex immune genes. This issue resurfaces in the discussion section, where again the point that HiC + Illumina is not sufficient is quite clear, but the converse does not seem well supported: long-read data in the absence of HiC does just fine.

The authors agree that this statement could be improved. Our results do support the reviewer's suggestion that assemblies based on long-read data, with or without scaffolding technology, are required for successful immune gene annotation. However, as outlined in the results section lines 298-320, immune gene families in the kitchen sink genomes represented by the 2021 platypus and koala assemblies were more intact than the woylie or 2018 platypus assembly (results presented in lines 368-380), both of which are based on long-read data. This was especially true for highly duplicated families such as the MHC, LRC NK receptors and TCR. The opening statement of the discussion has been modified to reflect the reviewer's suggestion, see lines 382-390 and below.

"By manually annotating immune genes in five marsupial genomes and two versions of the platypus genome, all varying qualities, we have confirmed that genome quality is directly linked to our ability to annotate complex immune gene families. Without long reads and scaffolding technologies, immune genes are scattered across many individual scaffolds and gene family organisation and evolution cannot be elucidated. We conclude that long-read data, with or without HiC technology, to generate a high-quality genome assembly with a contig N50 of at least 1MB is required to investigate immunity

and disease in wildlife. However, a kitchen sink approach to genome sequencing and assembly will enable complete reconstruction of complex and duplicated families such as MHC, TCR and LRC NK receptors as in the platypus 2021 and koala genomes."

6. The discussion of the limits of automated annotation is very important, but I found this section (starting on line 311) a little muddled. One key clarification is that it would probably be useful to separately discuss TCR and IG variable segments from all other immune genes. As the authors mention, automated analysis is not expected to successfully recover these variable regions, and it would probably be more useful to readers to get a sense of how automated analysis and RNA-seq alignment performs excluding these elements, in addition to the discussion on lines 329-337 of the specific challenges of variable regions.

This section of the discussion has been revised in response to the reviewer's comment and additional detail added. Automated annotation and RNAseq support for immune genes other than TCR and IG is now discussed in lines 408-437, while TCR and IG are solely discussed in lines 424-434. See amended text below.

"Aside from TCR and IG, the majority of immune genes incorrectly annotated or missing from the automated annotations were divergent genes not orthologous to those in eutherian mammals, such as MHC, marsupial-specific gene expansions within the LRC and monotreme-specific gene expansions within the NKC. Given their divergence, these genes often have low or no BLAST homology to nucleotide or protein databases. Gene models generated by automated annotation software are hypotheses based on supporting evidence such as RNAseq data and homology to nucleotide and protein databases. While immune transcripts were identified in the transcriptomes from these species, RNAseq data only supported gene models for a low proportion of MHC, LRC and NKC genes. RNAseq data only supported 8-16% of LRC gene predictions and 16-37% of MHC gene predictions amongst the four marsupial genome annotations which used RNAseq data as gene model evidence (koala, woylie, antechinus and numbat). Similarly, around 60% of NKC genes within the platypus genomes were supported by RNAseq data. Overall, RNAseq data did not provide enough evidence to support gene models for ~20% of immune genes within the genome. Some immune genes may not have been expressed in the tissue sequenced, were expressed at low levels, or were fragmented. For human and mouse, comprehensive and curated gene sets such as GENCODE and RefSeq are available to guide gene model predictions, comprising data from more than 10,000 RNA experiments and decades of dedicated work in this field [95, 96]. Given time, budget and sample constraints for wildlife, these curated gene sets are not available, hence RNAseq evidence is incomplete resulting in deficient gene models by automated annotation software.

It is not surprising that TCR and IG V segments were poorly or not annotated by all automated pipelines used to annotate the genomes in this study. These genes are notoriously difficult to characterise and are manually annotated in the human and mouse genome on Ensembl using the International Immunogenetics Information System (IMGT) database [38, 97]. Alignment of mature IG and TCR sequences from RNAseq data to the genome results in poor automated annotation, as V segments utilize different sequence signal splice sites to introns, which are not recognized by the open reading frame prediction algorithms. Indeed, RNAseq evidence only supported 7% to 18% of TCR V segment and 0% to 6.9%% of IG V segment gene predictions by automated pipelines amongst the four marsupial and platypus genomes. V sequences from three marsupials and two monotremes are available in IMGT, however as non-model species, they are not included in the scope for manual annotation by Ensembl or NCBI, so these important functional features are not annotated."

7. Regarding "it is not a requirement for manual changes to annotations to be tracked between genome versions" on line 353, I am not sure this is so simple. Even lifting over the old manual curation to new assembly coordinates probably needs itself to be manually verified before one can be confident that the new model is correct. But I do not think this would mean the information is lost, as I believe NCBI and Ensembl both maintain old annotations and assembly versions.

The authors agree that this statement was vague and so has been removed from the manuscript. While NCBI and Ensembl maintain old annotations and assembly versions, our argument still stands as there is currently limited scope to include manual gene annotations of the scale presented in our manuscript alongside existing automated annotations from these databases.

8. Given that 10x linked reads are no longer available for genome assembly, the extensive discussion of their uses and limitations on lines 431-457 could probably be condensed considerably.
This section of the discussion has been condensed, see lines 531—552 and text below. However, the authors feel discussing the limitations of 10x genomes for immune gene annotation is still warranted to make use of existing 10x assemblies, particularly for species where additional genome sequencing is unlikely due to sample or budget constraints.

"10x Chromium linked-read sequencing was insufficient to accurately re-assemble immune gene clusters in our study (Figure 4C). While this technology is no longer available for genome sequencing, acknowledging the limitations of this technology for immune gene annotation remains valid in order to make use of existing 10x genomes. Complete marsupial immune gene clusters can span hundreds of kilobases to megabases, as shown by annotation of the complete MHC, NK receptor and TCR regions in the koala (Additional file 2). DNA molecules input to 10x library preparation were on average 74 kbp and 23 kbp in antechinus and numbat respectively. This molecule size only spanned smaller immune clusters in the antechinus, such as the 70 kbp TRG locus, but was insufficient to span any cluster in the numbat. Interestingly, the antechinus MHC cluster appears to be intact (Figure 3), however manual annotation revealed multiple genes were "missing" within the scaffold and instead were located on individual short scaffolds. Regardless of input DNA molecule length, 10x libraries are still subject to the limitations of short-read sequencing regarding assembly of complex sequences. Antechinus and numbat 10x libraries were sequenced as short ~150 bp reads, hence while reads can be assigned back to the corresponding input DNA molecule, no single read spans the molecule length. Gaps between the reads make de novo assembly of repetitive and complex immune sequences difficult, often resulting in termination of contig extension and gene fragments scattered across short scaffolds [109-111]. These gene fragments can be misinterpreted as pseudogenes owing to loss of up/downstream coding regions (Figure 4C). For example, antechinus and numbat NK LRC genes share up to 97% and 98% amino acid sequence identity amongst the genes identified in each species respectively. The LRC should be encoded within a single cluster, as in the koala genome (Figure 3). Instead, the antechinus and numbat LRC clusters are fragmented across 33 and 34 scaffolds respectively."

Personal Cover
Click here to access/download;Personal Cover;GS cover letter_amended MS.pdf

THE UNIVERSITY OF SYDNEY

Professor Katherine Belov AO *BSc (Hons) PhD*
Pro Vice-Chancellor Global Engagement

11th August 2022

Dr Scott Edmunds
Chief Editor
GigaScience

Dear Dr Edmunds,

Please find attached our revised manuscript "*Best genome sequencing strategies for annotation of complex immune gene families in wildlife*" which we are re-submitting as a research article for publication in GigaScience. The text of the manuscript totals 7882 words, with four figures, two tables and two additional files. We would like to thank the reviewers for their valuable contributions. We have undertaken a re-analysis of the genomes using the same automated software, Fgenesh++, as recommended by reviewer 1, in addition to our previous work.

Globally we are in the midst of a biodiversity crisis and infectious diseases are a major driver of wildlife decline. The COVID-19 pandemic highlights the impact of wildlife disease on animal and human health, and provides impetus for studying immune genes in wildlife. Despite the recent increase in genomes for wildlife species, our understanding of immune genes in these species is limited owing to their high level of polymorphism and complex genomic organisation which makes assembly and annotation notoriously difficult. Due to our decade of research in wildlife immunogenetics we are increasingly asked the minimum genome quality required to effectively annotate immune genes which underpin wildlife disease investigations. In this manuscript we aimed to answer this question by manually annotating immune genes in five marsupial genomes and one monotreme genome of different qualities to determine the impact of sequencing strategy and automated annotation on accurate immune annotation.

We determined that high-quality chromosome-length genome assemblies generated using long-reads and scaffolding technologies are required to accurately annotate immune genes. Draft-quality genomes generated using short-reads and HiC technology, or now obsolete 10x Chromium linked-read technology, resulted in highly fragmented immune genes which led to incorrect annotation and prevented interpretation of genomic organisation and gene family evolution.

We feel the manuscript is now improved and will appeal to researchers involved in sequencing, assembly, annotation and translation of genomics data. We hope you will agree that this work represents an important contribution to GigaScience.

Yours sincerely,

KBelov.

Professor Kathy Belov
Corresponding Author
On Behalf of co-authors Emma Peel, Luke Silver, Parice Brandies, Ying Zhu, Yuanyuan Cheng and Carolyn Hogg

Office of the Pro Vice-Chancellor
Global Engagement
F23 Administration Building, Level 3
Corner of Eastern Avenue and City Road
The University of Sydney NSW 2006 Australia

T +61 2 9351 4461
E pvc.ge@sydney.edu.au

ABN 15 211 513 464
CRICOS 00026A
