# Supplementary material for: Best genome sequencing strategies for annotation of complex immune gene families in wildlife
Source: Gigascience. 2022 Oct 30;11:giac100. doi: 10.1093/gigascience/giac100 (PMC9618407; doi:10.1093/gigascience/giac100)
Supplement: giac100_Supplemental_Files [file giac100_supplemental_files.zip › Additional file 2_amended.docx]

Supplementary results

Best genome sequencing strategies for annotation of complex immune gene families in wildlife

Emma Peel^1^, Luke Silver^1^, Parice Brandies^1^, Ying Zhu^2^, Yuanyuan Cheng^1^, Carolyn J Hogg^1^ & Katherine Belov^1^

^1^School of Life and Environmental Sciences, The University of Sydney, Sydney, New South Wales, Australia ^2^ Sichuan Provincial Academy of Natural Resource Sciences, Chengdu, Sichuan, China

**Table of Contents**

[Supplementary Table 2 3](#_Toc109917644)

[Supplementary Figure 1 10](#_Toc109917645)

[Supplementary Figure 2 11](#_Toc109917646)

[Supplementary Figure 3 12](#_Toc109917647)

[Supplementary Figure 4 13](#_Toc109917648)

[Supplementary Figure 5 14](#_Toc109917649)

[Supplementary Figure 6 14](#_Toc109917650)

[Supplementary Figure 7 15](#_Toc109917651)

[Supplementary Table 3 16](#_Toc109917652)

[Toll-like receptors 17](#_Toc109917653)

[Supplementary Figure 8 18](#_Toc109917654)

[Natural killer receptors 19](#_Toc109917655)

[Natural killer complex (NKC) 19](#_Toc109917656)

[Supplementary Table 4 19](#_Toc109917657)

[Supplementary Figure 9 20](#_Toc109917658)

[Leukocyte receptor complex (LRC) 21](#_Toc109917659)

[Supplementary Table 5 22](#_Toc109917660)

[Supplementary Figure 10 23](#_Toc109917661)

[Supplementary Figure 11 24](#_Toc109917662)

[Cytokines 25](#_Toc109917663)

[Interferons 25](#_Toc109917664)

[Supplementary Figure 12 26](#_Toc109917665)

[Interleukins 27](#_Toc109917666)

[Supplementary Figure 13 28](#_Toc109917667)

[Tumour necrosis factors and transforming growth factors 29](#_Toc109917668)

[Supplementary Figure 14 30](#_Toc109917669)

[T cell receptors 31](#_Toc109917670)

[TRA/D 31](#_Toc109917671)

[Supplementary Table 6. 32](#_Toc109917672)

[Supplementary Figure 15. 33](#_Toc109917673)

[Supplementary Figure 16. 34](#_Toc109917674)

[TRB 35](#_Toc109917675)

[Supplementary Figure 17. 36](#_Toc109917676)

[TRG 37](#_Toc109917677)

[Supplementary Figure 18. 38](#_Toc109917678)

[TRM 39](#_Toc109917679)

[Supplementary Figure 19 40](#_Toc109917680)

[Immunoglobulins 41](#_Toc109917681)

[Supplementary Table 7 42](#_Toc109917682)

[Supplementary Figure 20 43](#_Toc109917683)

[Major Histocompatibility Complex 44](#_Toc109917684)

[Supplementary Table 8 44](#_Toc109917685)

[Supplementary Figure 21 45](#_Toc109917686)

[References 46](#_Toc109917687)

Supplementary Table 2. Marsupial-specific genes which are not orthologous to those in eutherians are italicised. * indicates the gene was not identified likely due to genome fragmentation and/or assembly error. ^includes partial sequences.

| **Immune gene family** | **Gene name** | **Koala** | **Woylie** | **Antechinus** | **Wombat** | **Numbat** | **Genes previously published in other marsupials** | **Number of human gene orthologs** |
| --- | --- | --- | --- | --- | --- | --- | --- | --- |
| **Immunoglobulin constant regions** | Cα | 1[1] | 1 | 1 | 1 | 1 | 1 devil, opossum, tammar wallaby [2] and long-nosed bandicoot [3] | 2 |
|  | Cε | 2 (1 identified in [1]) | 1 | 1 | 1 | 1 | 1 devil, opossum [4], tammar wallaby [2] and long-nosed bandicoot [3] | 1 |
|  | Cγ | 1 [1] | 3 | 1 | 1 | 1 | 1 devil, opossum [5], brushtail possum [6], tammar wallaby [2] and long-nosed bandicoot [3] | 4 |
|  | Cμ | 2 (1 identified in [7]) | 3 | 1 | 1 | 1 | 1 devil, opossum [8], brushtail possum [9] & long-nosed bandicoot [3] | 1 |
|  | Cδ | 0 [7] | 0 | 0 | 0 | 0 | 0 opossum [5] & long-nosed bandicoot [3] | 1 |
|  | Cλ | 9 (4 identified in [7]) | 10 | 2 | 4^ | 1 | 4 devil [10]  8 opossum [5]  4 brushtail possum [11]  2 long-nosed bandicoot [3] | 5 |
|  | Cκ | 1 [7] | 2 | 1 | 4^ | 1 | 1 devil, opossum [12], brushtail possum [13] & long-nosed bandicoot [3] | 1 |
| **T cell receptor constant (C) and variable (V) regions** | Cα | 1 | 1 | 1 | 1 | 1 | 1 devil, opossum [14], brushtail possum [15], northern brown bandicoot [16] & long-nosed bandicoot [3] | 1 |
|  | Cδ | 1 | 1 | 1 | 1 | 1 | 1 devil, opossum [17], long-nosed bandicoot [3] & tammar wallaby [18] | 1 |
|  | Vα/δ | 59 [7] | 76 | 57 | 57 | 51 | 60 opossum [17] | 44-47 |
|  | Cβ | 3 [7] | 3 | 3 | 3 | 3 | 4 opossum [14]  3 devil [10]  2 long-nosed bandicoot [3]  3 thylacine [19] | 2 |
|  | Vβ | 34 [7] | 26 | 46 | 17 | 34 | 27 opossum [17] | 40-48 |
|  | Cγ | 1 [7] | 1 | 1 | 1 | 1 | 1 devil, opossum [17], northern brown bandicoot [16] & long-nosed bandicoot [3] | 2 |
|  | Vγ | 5 [7] | 12 | 13 | 6 | 12 | 9 opossum [17] | 4-6 |
|  | *Cμ* | 3[7] | 6 | 5 | 4 | 3 | 7 devil [10]  8 opossum [17, 20]  6 northern brown bandicoot [16]  6 long-nosed bandicoot [3]  4 thylacine [19] | Not orthologous |
|  | *Vμ* | 3 [7] | 4 | 5 | 4 | 3 | 5 opossum [17] | Not orthologous |
|  | *Vμj* | 3 [7] | 4 | 5 | 3 | 4 | 8 opossum [17] | Not orthologous |
| **Toll-like receptors** | TLR1 | Not identified | | | | | Not identified in devil [21] or opossum [22] | 1 |
|  | TLR2 | 1 [23] | 1 | 1 | 1 | 1 | 1 devil [21], opossum [22] & long-nosed bandicoot [3] | 1 |
|  | TLR3 | 1 [23] | 1 | 1 | 1 | 1 |  | 1 |
|  | TLR4 | 1 [23] | 1 | 1 | 1 | 1 |  | 1 |
|  | TLR5 | 1 [23] | 1 | 1 | 1 | 1 |  | 1 |
|  | TLR6 | Not identified | | | | | Not identified in devil [21] or opossum [22] | 1 |
|  | TLR7 | 1 [23] | 1 | 1 | 1 | 1 | 1 devil [21], opossum [22], long-nosed bandicoot [3] | 1 |
|  | TLR8 | 1 [23] | 1 | 1 | 1 | 1 |  | 1 |
|  | TLR9 | 1 [23] | 1 | 1 | 1 | 1 |  | 1 |
|  | TLR10 | 1 [23] | 1 | 1 | 1 | 1 |  | 1 |
|  | TLR13 | 1 [23] | 1 | 1 | 1 | 1 |  | 1 |
|  | *TLR1/6* | 1 [23] | 1 | 1 | 1 | 1 |  | Not orthologous |
| **MHC class I** | 11 | 19 | 17 | 7 | 5 | 3 | 15 devil [24-26]( Papenfuss 2015)  18 opossum [26, 27]  11 tammar wallaby [26, 28]  1 long-nosed bandicoot [3] | Some marsupial genes are not orthologous to eutherians |
| **MHC class II** | *DA, DB, DC* and DM (α and β chain encoded by separate loci for each) | 16 (Johnson 2018) | 23 | 14 | 7 | 6 | 10 opossum [27, 29]  14-17 tammar wallaby [30]  7 long-nosed bandicoot [3] | All except DM are not orthologous |
| **Interleukins** | IL3 | Not identified [31, 32] | Not identified | Not identified | Not identified | Not identified | Not identified in devil [10] or opossum [31, 32] | 1 |
|  | IL32 |  |  |  |  |  |  | 1 |
|  | IL37 |  |  |  |  |  |  | 1 |
|  | IL11 |  | 1 | 1 | 1 | 1 | Not identified in devil [10], koala or opossum [31, 32] | 1 |
|  | IL17A, B, C, D, E (IL25) & F | 6 [33] | 6 | 6 | 6 | 6 | All six members identified in opossum [31] & devil [10] | All six members |
|  | *IL36L1* | Pseudogene | 1 | 1 | 1 | 1 | 1 devil [10]  Not identified in opossum [31] | Not orthologous |
|  | *IL36L2* | 1 | 1 | 1 | 1 | 1 | 1 devil [10]  Not identified in opossum [31] | Not orthologous |
|  | IL18A | 1 | 1 | 1 | 1 | 1 | 1 devil [10], opossum [31], long-nosed bandicoot [3] & tammar wallaby | 1 |
|  | *IL18B* | 1 | 1 | 1 | 1 | 1 | 1 devil [10] & opossum [10] | Not orthologous |
|  | *IL18C* | 1 | 1 | 1 | 1 | 1 | 1 devil [10] | Not orthologous |
|  | IL1A | 1 | 1 | 1 | 1 | 1 | 1 devil [10] | 1 |
|  | IL1B | 1 | 1 | 1 | 1 | 1 | 1 devil [10], koala [1], brushtail possum [34] & tammar wallaby [35] | 1 |
|  | IL1F10 | 1 | 1 | 1 | 1 | 1 | 1 devil [10] & opossum [31] | 1 |
|  | IL1RN | 1 | 1 | 1 | 1 | 1 | 1 devil [10] & opossum [31] | 1 |
|  | IL2 | 1 | 1 | 1 | 1 | 1 | 1 devil [36], tammar wallaby [36], brushtail possum [37] & opossum [32] | 1 |
|  | IL4 | 1 | 1 | 1 | 1 | 1 | 1 devil [10], koala [1], tammar wallaby [38], long-nosed bandicoot [3] & opossum [32] | 1 |
|  | IL5 | 1 | 1 | 1 | 1 | 1 | 1 devil [10], stripe-faced dunnart [39], long-nosed bandicoot [3], tammar wallaby [39]& opossum [31] | 1 |
|  | IL6 | 1 [40] | 1 | 1 | 1 | 1 | 1 devil [10] , opossum [32], red-tailed phascogale [41], kultarr [41], fat-tailed dunnart [41], stripe-faced dunnart [41], long-nosed bandicoot [3] & tammar wallaby [42] | 1 |
|  | IL7 | 1 | 1 | 1 | 1 | 1 | 1 devil [10] & opossum [31] | 1 |
|  | IL8 | 1 | 1 | 1 | 1 | 1 | 1 devil [10] & opossum [31] | 1 |
|  | IL9 | 1 | 1 | 1 | 1 | 1 | 1 devil [10] & opossum [31] | 1 |
|  | IL10 | 1 [40] | 1 | 1 | 1 | 1 | 1 devil [10] brushtail possum [43], tammar wallaby [44], rufous hare-wallaby [44], long-nosed bandicoot [3] & opossum [31] | 1 |
|  | IL12A | 1 | 1 | 1 | 1 | 1 | 1 devil [10], koala [1] & opossum [32] | 1 |
|  | IL12B | 1 | 1 | 1 | 1 | 1 | 1 devil [10], koala [1] & opossum [31] | 1 |
|  | IL13 | 1 | 1 | 1 | 1 | 1 | 1 devil [10], long-nosed bandicoot [3] & opossum [32] | 1 |
|  | IL15 | 1 | 1 | 1 | 1 | 1 | 1 devil [10] & opossum [31] | 1 |
|  | IL16 | 1 | 1 | 1 | 1 | 1 | 1 devil [10] | 1 |
|  | IL19 | 1 | 1 | 1 | 1 | 1 | 1 devil [10] & opossum [32] | 1 |
|  | IL20 | 1 | 1 | 1 | 1 | 1 | 1 devil [10] & opossum [32] | 1 |
|  | IL21 | 1 | 1 | 1 | 1 | 1 | 1 devil [10], tammar wallaby [45], bridled nailtail wallaby [45] & opossum [32] | 1 |
|  | IL22 | 1 | 1 | 1 | 1 | 1 | 5 homologs identified in the devil (IL22F1 through 5) [10]  1 opossum [32] | 1 |
|  | IL23 | 1 | 1 | 1 | 1 | 1 | 1 devil [10] & opossum [31] | 1 |
|  | IL24 | 1 | 1 | 1 | 1 | 1 | 1 devil [10] & opossum [32] | 1 |
|  | IL26 | 1 | 1 | 1 | 1 | 1 | 1 devil [10] & opossum [32] | 1 |
|  | IL27 | 1 | 1 | 1 | 1 | 1 | 1 devil [10] & opossum [31] | 1 |
|  | IL31 | 0 | 0 | 1 | 1 | 1 | 1 devil [10] & opossum [31] | 1 |
|  | IL33 | 1 | 1 | 1 | 1 | 1 | 1 devil [10] & opossum [31] | 1 |
|  | IL36RN | 1 | 1 | 1 | 1 | 1 | 1 devil [10] & opossum [31] | 1 |
| **Interferons** | IFNδ | 0 | 0 | 0 | 0 | 0 | Not identified in any marsupial studied to date [10, 31, 32] | 1 |
|  | IFNε |  |  |  |  |  |  | 0 |
|  | IFNω |  |  |  |  |  |  | 1 |
|  | IFNκ | 1 | 1 | 0* | 1 | 0* | 1 opossum [32] & thylacine [19]  Not identified in devil [10] | 1 |
|  | IFNβ | 5 | 1 | 1 | 1 | 1 | 1 devil [10], thylacine [19] & opossum [32] | 1 |
|  | IFNα | 13 | 13 | 4 | 11 | 2 | 4 devil [10]  7 opossum [32]  4 thylacine [19] | 13 |
|  | IFNγ | 1 | 1 | 1 | 1 | 1 | 1 devil [10], koala [40], tammar wallaby [46], rufous hare-wallaby [46], long-nosed bandicoot [3], thylacine [19] & opossum [32] | 1 |
|  | IFNλ | 1 | 1 | 1 | 1 | 1 | 1 devil [10]  0 opossum [31]  1 tammar wallaby | 4 |
| **Tumour necrosis factors** | TNFSF18 | 1 | 1 | 1 | 1 | 1 | 1 devil [10] | 1 |
|  | TNFSF10L | 1 | 1 | 1 | 1 | 1 | 1 devil [10] & opossum [31] | 1 |
|  | TNFSF10 (CD253) | 1 | 1 | 1 | 1 | 1 | 1 devil [10] & opossum [31] | 1 |
|  | TNFSF11 (CD254) | 1 | 1 | 1 | 1 | 1 | 1 devil [10] & opossum [31] | 1 |
|  | TNFSF12 | 1 | 0 | 0 | 0 | 0 |  |  |
|  | TNFSF13B (CD256) | 1 | 1 | 1 | 1 | 1 | 1 devil [10] | 1 |
|  | *TNFSF13L* | 1 | 1 | 1 | 1 | 1 | 1 devil [10] & opossum [31] | Not orthologous |
|  | TNFSF14 (CD258) | 1 | 1 | 1 | 1 | 1 | 1 devil [10] & opossum [31] | 1 |
|  | TNFSF15 (TL1) | 1 | 1 | 1 | 1 | 1 | 1 devil [10] & opossum [31] | 1 |
|  | TNFSF9 | 1 | 1 | 1 | 1 | 1 | 1 devil [10] | 1 |
|  | TNFSF8 (CD153) | 1 | 1 | 1 | 1 | 1 | 1 devil [10] | 1 |
|  | TNFSF4 | 1 | 1 | 1 | 1 | 1 | 1 devil [10] | 1 |
|  | EDA | 1 [7] | 1 | 1 | 1 | 1 | 1 devil [10], tammar wallaby [47], long-nosed bandicoot [3] & opossum [31] | 1 |
|  | LTA (TNFβ) | 1 [7] | 1 | 1 | 1 | 1 | 1 devil [10], tammar wallaby [48], long-nosed bandicoot [3] & opossum [31] | 1 |
|  | LTB (TNFγ) | 1 | 1 | 1 | 1 | 1 | 1 devil [10]  0 opossum [31] (pseudogene) | 1 |
|  | FASLG | 1 [7] | 1 | 1 | 1 | 1 | 1 devil [10], brushtail possum [49], long-nosed bandicoot [3] & opossum [31] | 1 |
|  | TNF (TNFα) | 1 | 1 | 1 | 1 | 1 | 1 devil [10] & opossum [31] | 1 |
|  | CD70 | 1 | 1 | 1 | 1 | 1 | 1 devil [10] & opossum [31] | 1 |
|  | CD40LG | 1 | 1 | 1 | 1 | 1 | 1 devil [10]  0 opossum [31]  1 tammar wallaby | 1 |
| **Transforming growth factors** | TGFβ1 | 1 | 1 | 1 | 1 | 1 | 1 devil (Morris 2015) | 1 |
|  | TGFβ2 | 1 | 1 | 1 | 1 | 1 | 1 devil (Morris 2015) | 1 |
|  | TGFβ3 | 1 | 1 | 1 | 1 | 1 | 1 devil (Morris 2015) | 1 |
| **Natural killer receptors:**  **NKC** | CLEC4 family | CLEC4E [50]  CLEC4G  CLEC4-like 1 | CLEC4  CLEC4E  CLEC4G  CLEC15A  CLEC4-like 1 | CLEC4  CLEC4E  CLEC15A | CLEC4  CLEC4E  CLEC15A | CLEC4  CLEC4E  CLEC4G  CLEC15A  CLEC4-like 1 | CLEC4E devil [51], opossum [22]  CLEC4 opossum [22]  CLEC15A opossum [22] | 8 members: CLEC4A, CLEC4C, CLEC4D, CLEC4E, CLEC4F, CLEC4G, CLEC4M & CLEC4O |
|  | CLEC1A | 1 [50] | 1 | 1 | 1 | 1 | 1 devil [51], opossum [22] & thylacine [19] | 1 |
|  | CLEC1B | 1 [50] | 1 | 1 | 1 | 1 | 1 devil [51] & opossum [22] & thylacine [19] | 1 |
|  | CLEC11A | 1 | 0 | 0 | 0 | 1 | Not identified in any other marsupial to date | 1 |
|  | CLEC17A | 1 | 1 | 0 | 0 | 1 | Not identified in any other marsupial to date | 1 |
|  | CD69 | 1 [50] | 1 | 1 | 1 | 1 | 1 devil & opossum | 1 |
|  | OLR1 | 0 | 0 | 0 | 0 | 0 | Not identified in any marsupial to date | 1 |
|  | *CLEC2-like* | 1 [50] | 1 | 1 | 0* | 1 | 1 devil [51], opossum [22] & thylacine [19] | 0 |
|  | *KLR family* | KLRK1 [50]  KLRF1  KLRG2  KLRF-like1  KLRF-like2  KLRF1-like | KLRK1  KLRF1  KLRG2  KLRF-like1  KLRF-like2 | KLRK1  KLRF1  KLRF-like1  KLRF-like2 | KLRK1  KLRF1  KLRF-like1  KLRF-like2  KLRF1-like | KLRK1  KLRF1  KLRG2  KLRF-like1  KLRF-like2  KLRF1-like | KLRK1 devil [51] & opossum [22]  KLRF1-like1 & KLRF1-like2 opossum [22] | 8 members: KLRC1, KLRC2, KLRC3, KLRK1 (KLRC4), KLRD, KLRF1 & KLRF2 |
| **Natural killer receptors:**  **LRC** | *IG domains not orthologous to eutherian LRC families* | 43 [50] | 60 | 51 | 33 | 38 | 66 long-nosed bandicoot [3]  35 devil DIGs [51]  154 opossum MAIRs[22]  95 thylacine TIGs [19] | Humans have ~30 NK receptor genes encoded within the LRC. Each gene contains between 2 and 7 Ig domains depending on the family (KIR, LILR, LAIR, GP6, NCR1 and FCAR). |
|  | GPVI | 1 [50] | 1 | 2 | 0* | 2 | 1 devil [10] and opossum [22] | 1 |

**
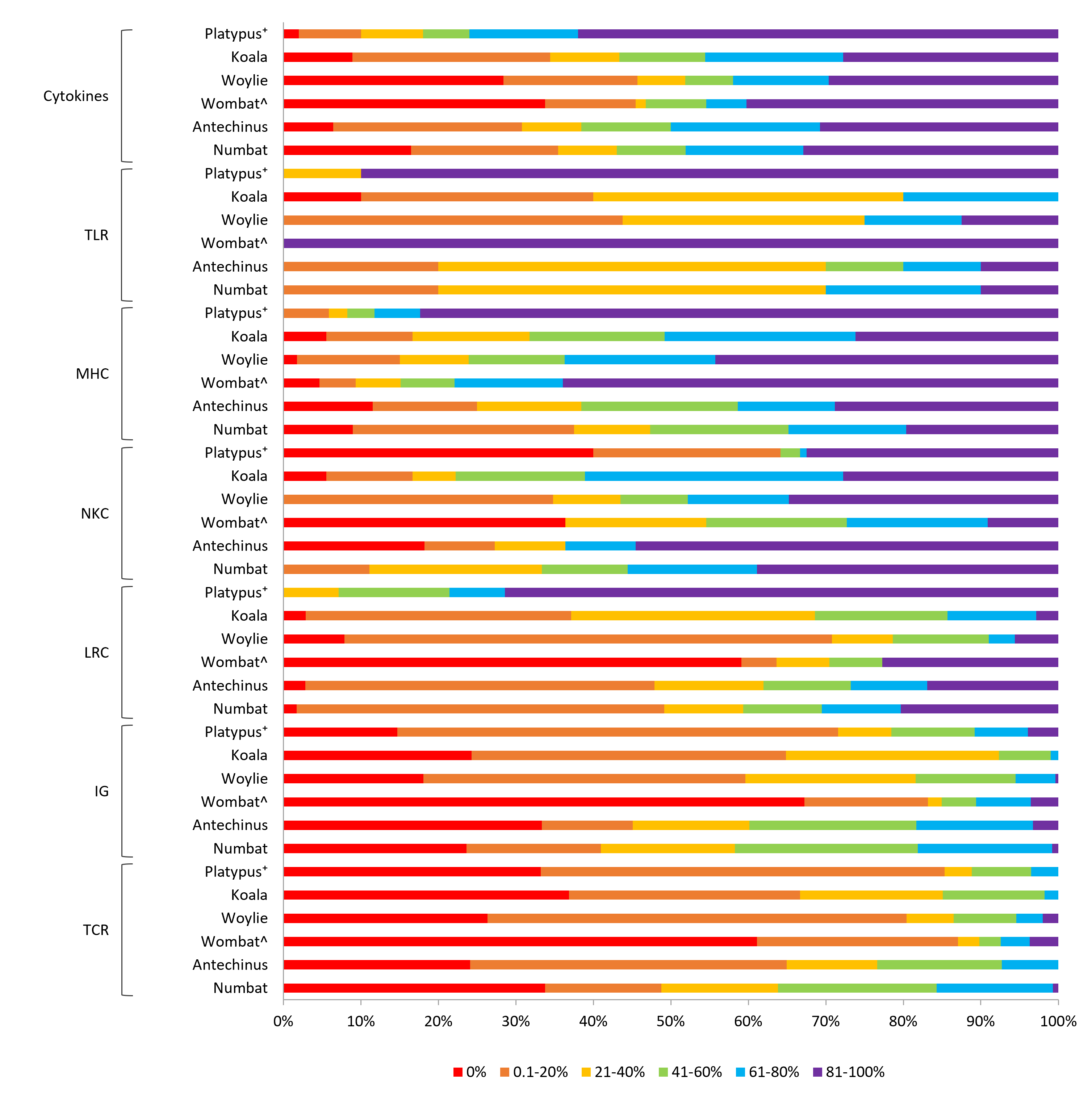
**Supplementary Figure 1. Percentage overlap of genomic coordinates between manual and automated annotations of immune genes from seven families (TCR, MHC, IG, LRC, NKC, TLR and cytokines) in six genomes. The platypus 2021 genome assembly GCA_004115215.4 was used in this analysis. *Denotes automated annotation by NCBI and ^denotes automated annotation by MAKER. The remaining genomes were annotated using Fgenesh++. Colours indicate proportion of immune genes with 0 to 100% overlap between manual and automated annotations, with 0 indicating manually annotated genes with no overlap of genomic coordinates with the automated annotation.

**
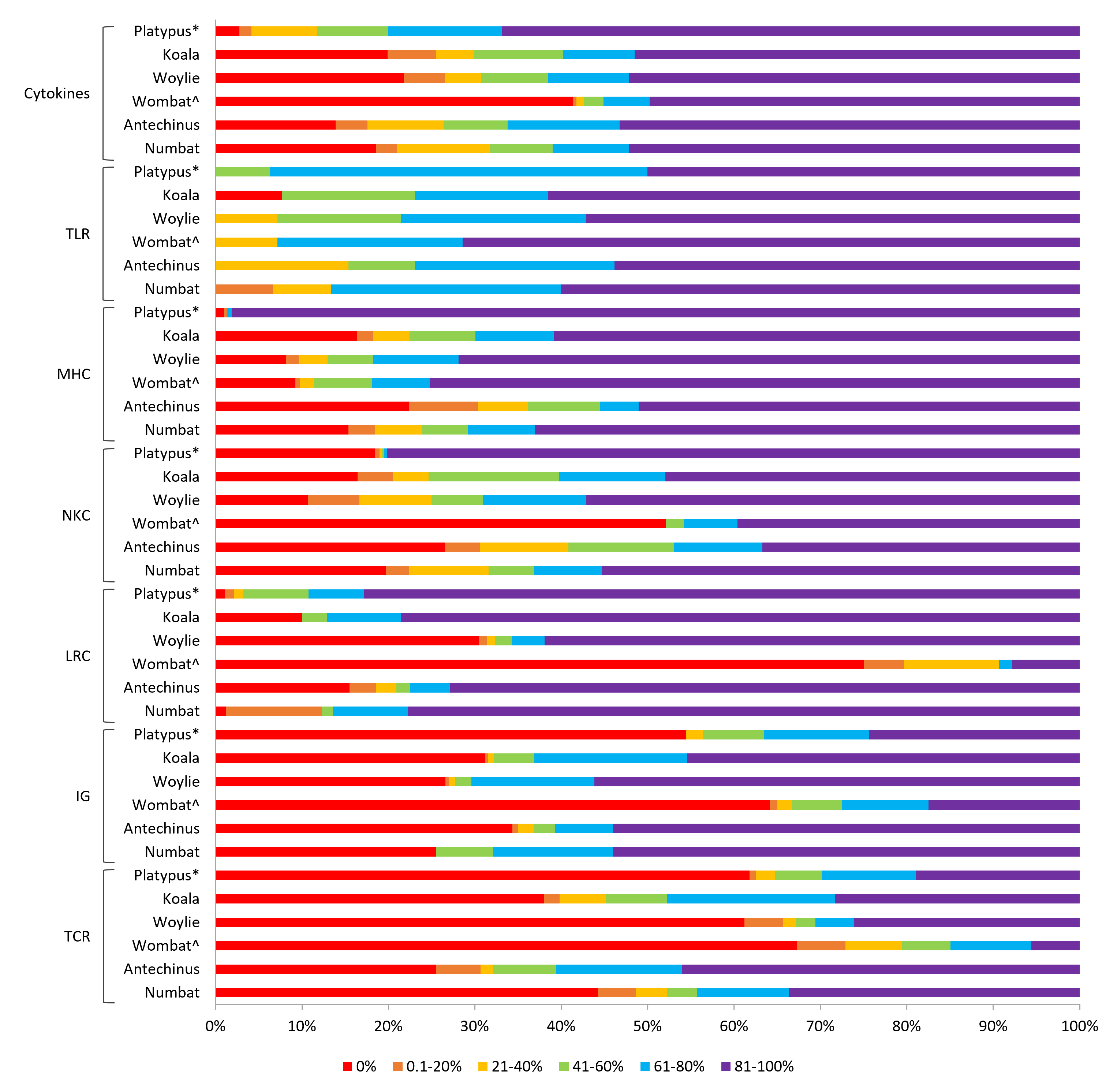
**Supplementary Figure 2. Percentage overlap of genomic coordinates between manual and automated annotations of exons encoding immune genes from seven families (TCR, MHC ,IG, LRC, NKC, TLR and cytokines) in six genomes. The platypus 2021 genome assembly GCA_004115215.4 was used in this analysis. *Denotes automated annotation by NCBI and ^denotes automated annotation by MAKER. The remaining genomes were annotated using Fgenesh++. Colours indicate proportion of immune genes with 0 to 100% overlap between manual and automated annotations, with 0 indicating manually annotated genes with no overlap of genomic coordinates with the automated annotation.

**
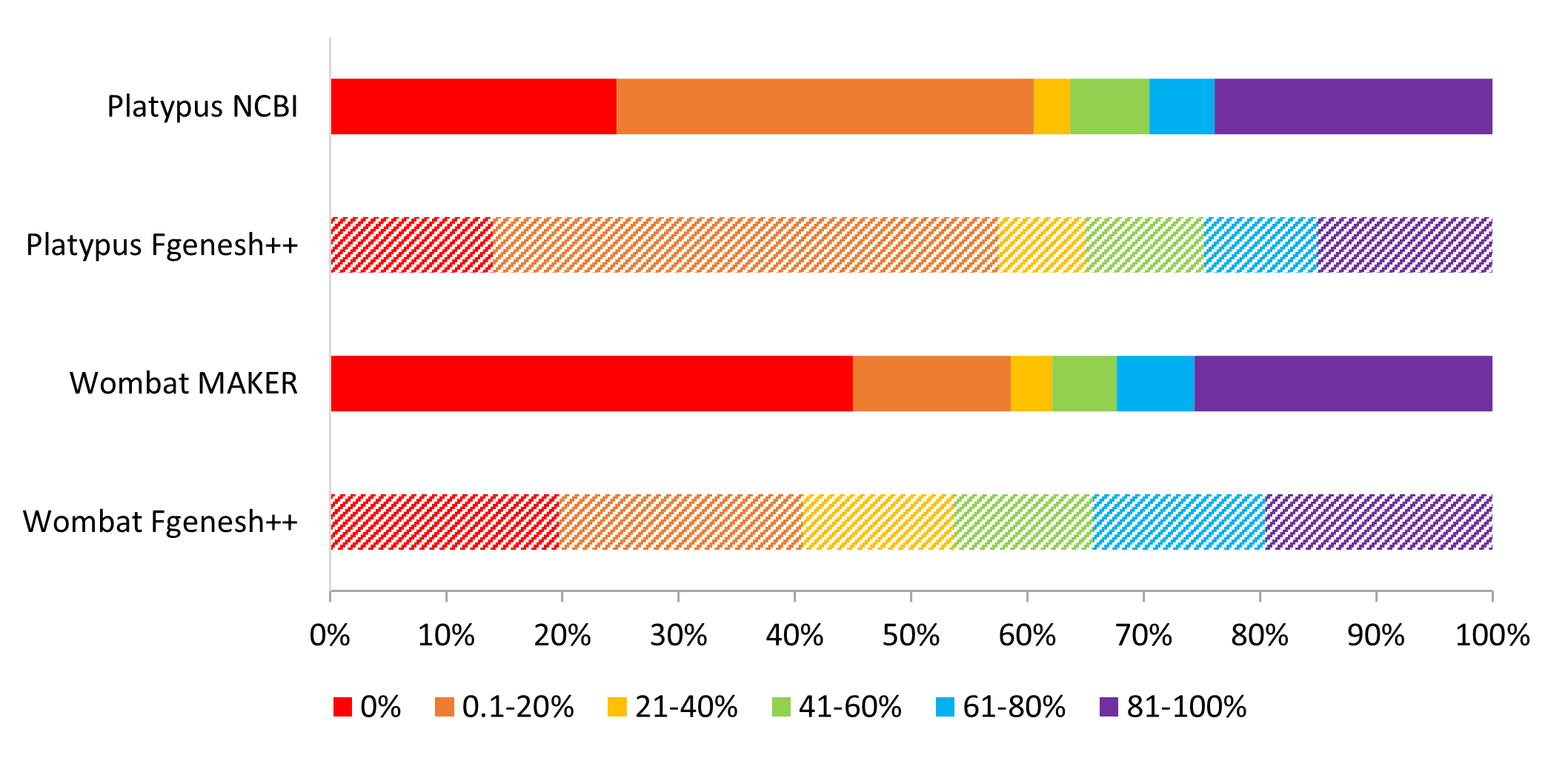
**Supplementary Figure 3. Percentage overlap of genomic coordinates between manual and automated immune gene annotations from NCBI (platypus 2021 genome assembly GCA_004115215.4) and MAKER (wombat) indicated by solid bars, compared to those from Fgenesh++ for the two species, indicated by hashed bars. Colours indicate proportion of immune genes with 0 to 100% overlap between manual and automated annotations, with 0 indicating manually annotated genes with no overlap of genomic coordinates with the automated annotation.

**
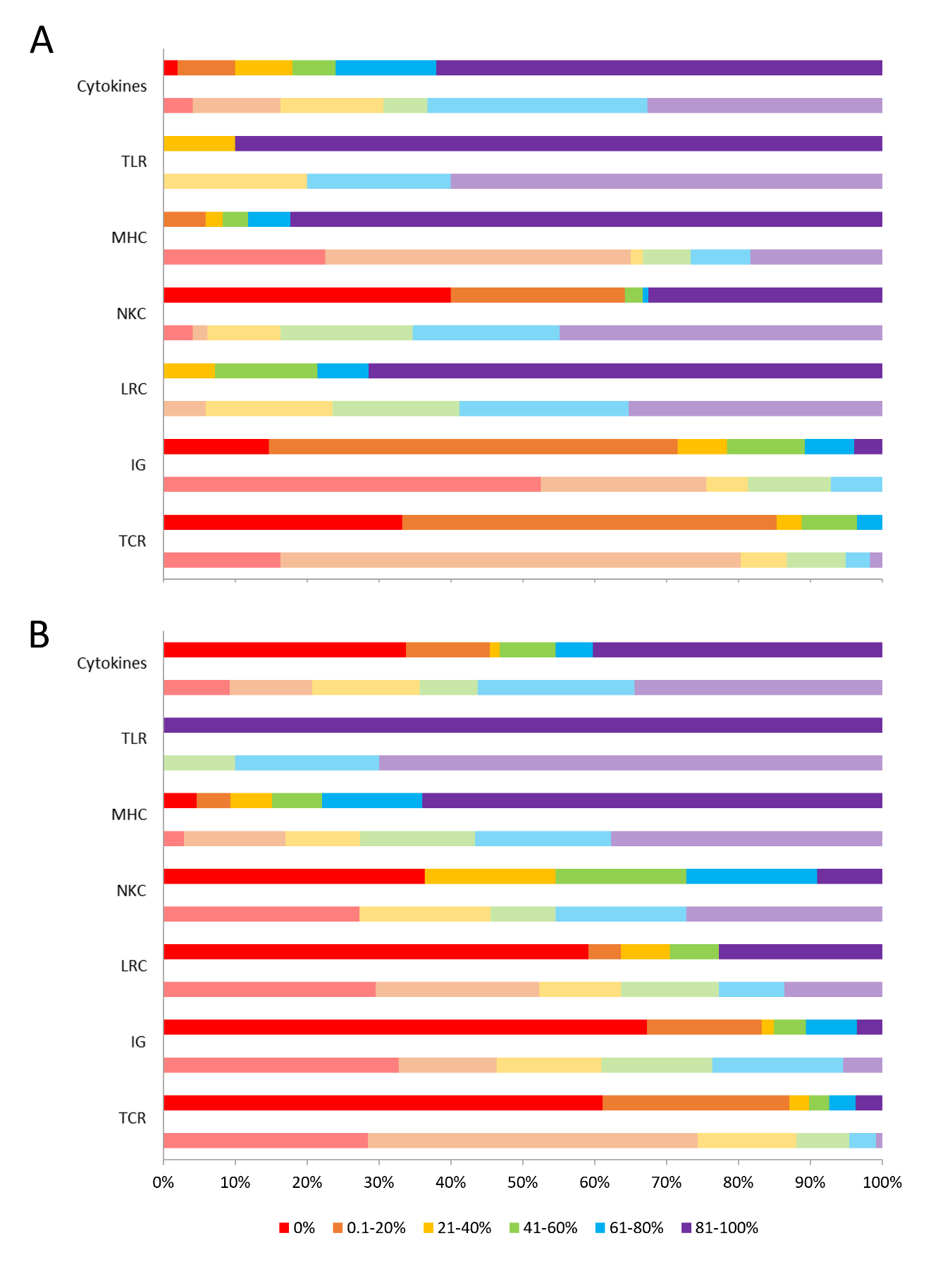
**Supplementary Figure 4. (A) percentage overlap of genomic coordinates between manual and NCBI (solid colour) or Fgenesh++ (faded colour) automated annotations of immune genes from seven families in the platypus 2021 genome GCA_004115215.4. (B) percentage overlap of genomic coordinates between manual and MAKER (solid colour) or Fgenesh++ (faded colour) automated annotations of immune genes from seven famlies in the wombat genome. Colours indicate proportion of immune genes with 0 to 100% overlap between manual and automated annotations, with 0 indicating manually annotated genes with no overlap of genomic coordinates with the automated annotation.


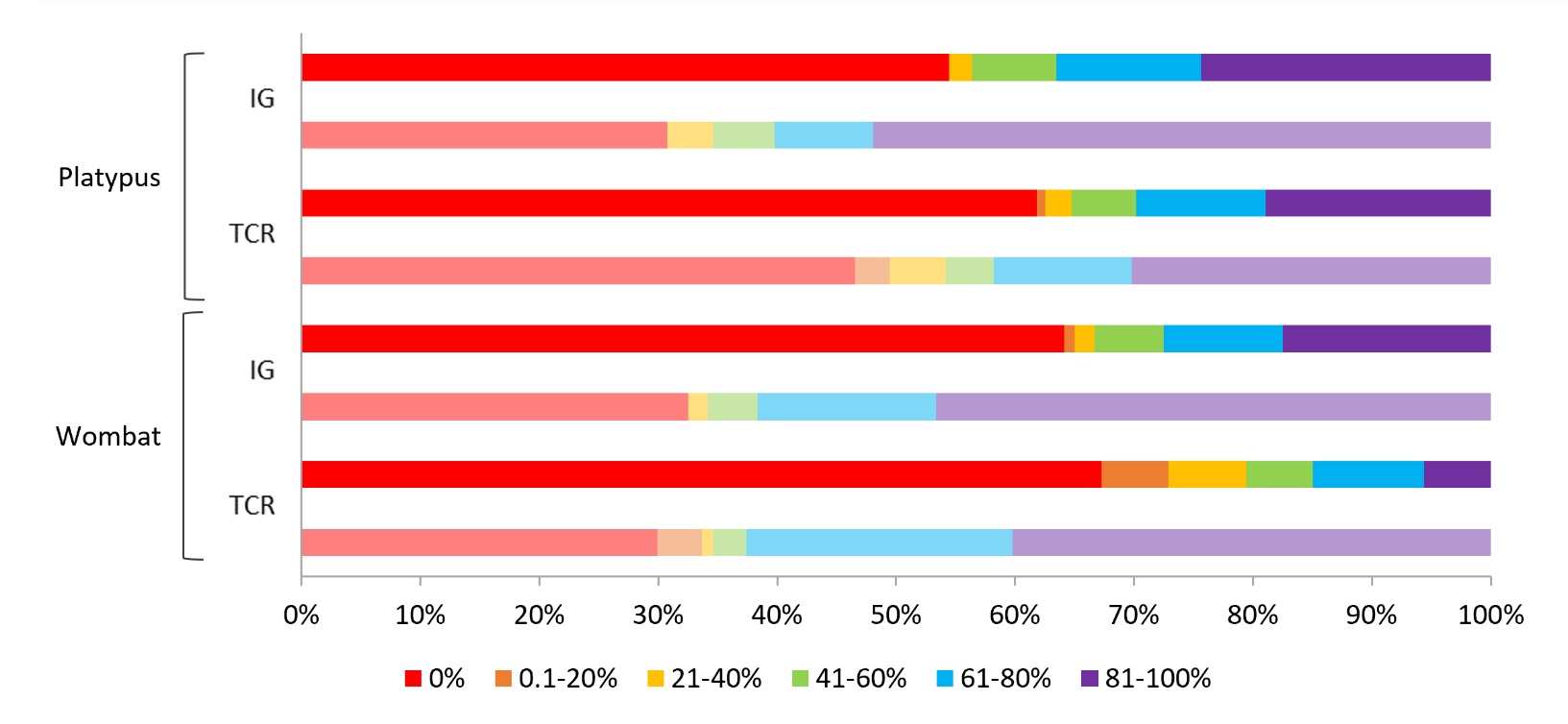
Supplementary Figure 5. Percentage overlap of genomic coordinates between manual and automated annotations of exons encoding immune genes from the TCR and IG families in the platypus 2021 and wombat genomes. Automated annotations used in this analysis are from NCBI (platypus) or MAKER (wombat) indicated by solid colour, compared to those from Fgenesh++ for the two species, indicated by faded colour. Colours indicate proportion of immune genes with 0 to 100% overlap between manual and automated annotations, with 0 indicating manually annotated genes with no overlap of genomic coordinates with the automated annotation.


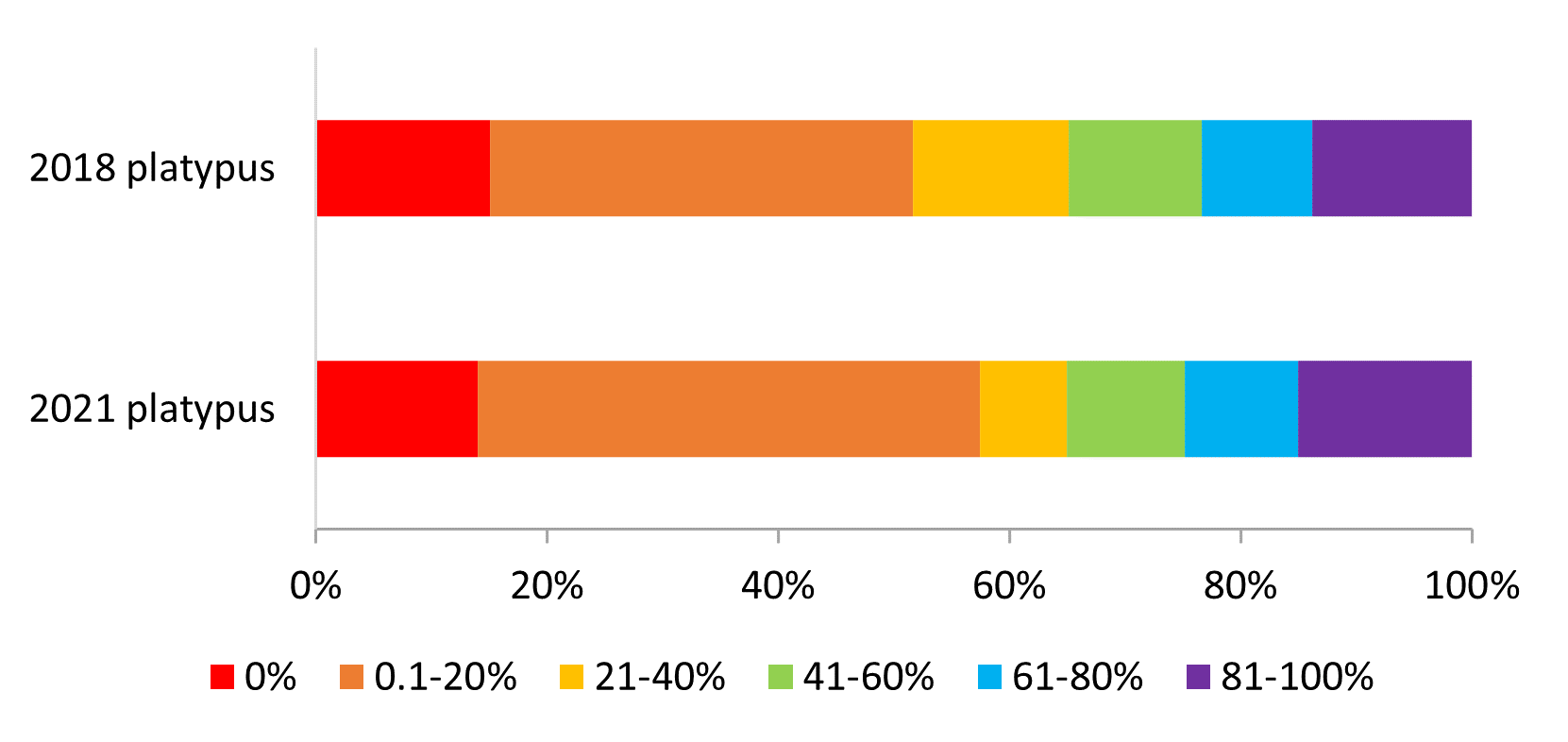


Supplementary Figure 6. Percentage overlap of genomic coordinates between manual and automated annotations of immune genes in the 2021 versus 2018 platypus genome assemblies. Both assemblies were annotated using Fgenesh++. Colours indicate proportion of immune genes with 0 to 100% overlap between manual and automated annotations, with 0 indicating manually annotated genes with no overlap of genomic coordinates with the automated annotation.

**
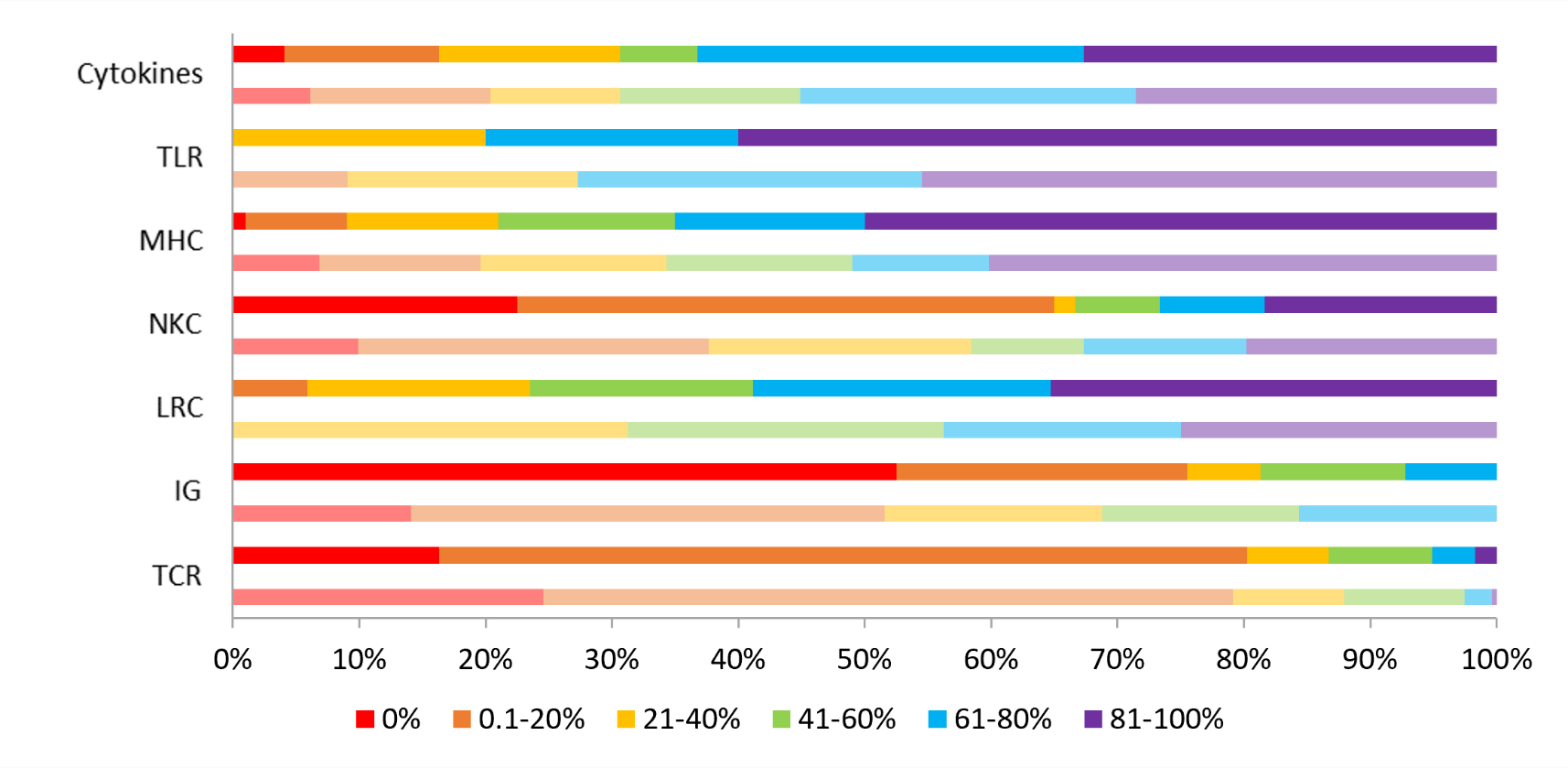
**Supplementary Figure 7. Percentage overlap of genomic coordinates between manual and Fgenesh++ automated annotations of immune genes in the 2021 (solid bars) and 2018 (faded bars) platypus genome assemblies. Colours indicate proportion of immune genes with 0 to 100% overlap between manual and automated annotations, with 0 indicating manually annotated genes with no overlap of genomic coordinates with the automated annotation.

Supplementary Table 3. Sequence read archive (SRA) accession numbers of RNAseq data from platypus and koala used to generate global transcriptomes. The percentage of paired RNAseq reads retained following quality and length trimming for each tissue is also listed.

| **Species** | **Tissue** | **SRA accession number** | **% Paired reads retained post-trimming** |
| --- | --- | --- | --- |
| Platypus  (*Ornithorhynchus anatinus*) | Ovary female 1 | SRX2704286 | 95.57 |
|  | Ovary female 2 | SRX2704287 | 96.81 |
|  | Testis male 1 | SRX2704288 | 95.63 |
|  | Testis male 2 | SRX2704289 | 89.98 |
|  | Liver male 1 | SRX2704284 | 90.87 |
|  | Liver male 2 | SRX2704285 | 89.47 |
|  | Liver female 1 | SRX2704282 | 97.29 |
|  | Liver female 2 | SRX2704283 | 96.42 |
|  | Kidney male 1 | SRX2704280 | 92.60 |
|  | Kidney male 2 | SRX2704281 | 90.53 |
|  | Kidney female 1 | SRX2704278 | 97.35 |
|  | Kidney female 2 | SRX2704279 | 96.42 |
|  | Heart male 1 | SRX2704276 | 92.53 |
|  | Heart male 2 | SRX2704277 | 91.42 |
|  | Brain male 1 | SRX2704272 | 96.18 |
|  | Brain male 2 | SRX2704271 | 91.48 |
|  | Brain female 1 | SRX2704270 | 98.54 |
|  | Brain female 2 | SRX2704271 | 97.83 |
|  | Male crural gland | SRX026473 | 99.95 |
| Koala  (*Phascolarctos cinereus*) | Female spleen | SRX500280 | 99.12 |
|  | Female liver | SRX501262 | 98.28 |
|  | Uterus | SRX501302 | 99.11 |
|  | Female kidney | SRX501314 | 98.94 |
|  | Female lung | SRX501320 | 99.05 |
|  | Female heart | SRX501342 | 98.34 |
|  | Female brain | SRX501343 |  |
|  | Female adrenal gland | SRX501344 | 99.10 |
|  | Mammary gland | SRX1881580 | 97.46 |
|  | Male salivary gland, testis, kidney, bone marrow, lymph node, spleen and liver | SRR1106690 | 99.67 |
|  |  | SRR1106707 | 99.87 |
|  |  | SRR1122141 | 99.92 |
|  |  | SRR1121764 | 99.09 |
|  |  | SRR1207973 | 98.84 |
|  |  | SRR1207974 | 99.51 |
|  |  | SRR1207975 | 98.91 |

# Toll-like receptors

Toll-like receptor (TLRs) are membrane-spanning receptors expressed on the surface of immune and non-immune cells, which form an important component of the innate immune system. TLRs recognise and bind conserved molecules on the surface of pathogens, known as pathogen-associated molecular patterns (PAMPs), leading to activation of the innate and adaptive immune response. Ten TLR genes were identified in the woylie, antechinus, wombat and numbat genomes, as characterised previously in the koala [23]. Of these, nine have direct orthologs in eutherians; TLR2- 5, TLR7-10 and TLR13 (supplementary figure 8). The marsupial-specific TLR1/6-like was also identified in woylie, antechinus, wombat, and numbat genomes, which cluster in a sister clade to eutherian TLR1 and TLR6 (supplementary figure 8). This reflects the ancestral nature of TLR1/6, which is thought to have evolved through gene duplication into TLR1 and TLR6 identified in eutherian mammals [52]. In the woylie, wombat, antechinus, and numbat genomes, ten TLR genes were located on eight different scaffolds, while in the koala genome they were located on six. All ten TLRs were highly conserved across the five species studied, sharing up to 96% amino acid identity amongst them. A defining feature of TLRs is the presence of multiple leucine rich repeats (LRR) motifs that are involved in PAMP recognition and binding. From 19 to 27 LRRs were identified in woylie, wombat, antechinus and numbat TLRs, similar to previously published for koala TLRs [23]. In addition, amino acid residues essential for PAMP binding in human and mouse, such as bacterial lipopolysaccharide and flagellin, were also identified in the marsupial species studied here [53-58].

**
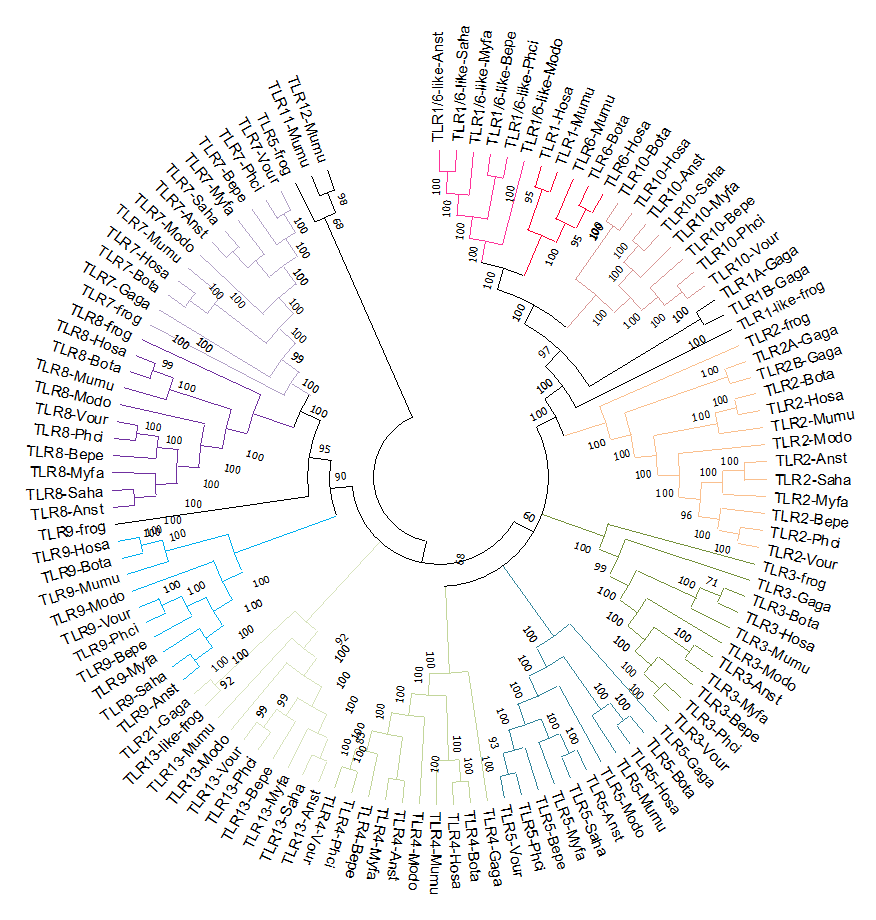
**

Supplementary Figure 8. Phylogenetic relationships amongst woylie, antechinus, wombat, numbat, other marsupial, monotreme and eutherian toll-like receptors (TLRs). Colours indicate clustering of TLR genes. The neighbour-joining phylogenetic tree was constructed in MEGAX using the p-distance method, pairwise deletion and 500 bootstrap replicates. Bootstrap values less than 50% are not shown.

# Natural killer receptors

Natural killer (NK) cells are an essential component of innate immunity as they identify and target cancerous and virus-infected cells. Membrane-bound receptors expressed on the surface of NK cells bind to MHC class I- and class II-bound ligands, which initiates cytokine secretion and degranulation that ultimately leads to target cell death. The genes which encode NK receptors all share the same function, however contain two different protein domains and are located in two separate regions of the genome. Genes encoded within the leukocyte receptor complex (LRC) contain immunoglobulin (Ig) domains belonging to the immunoglobulin superfamily (IgSF), while genes encoded within the natural killer complex (NKC) contain C-type lectin domains (CTLD) and hence belong to the C-type lectin superfamily (CLSF). As NK receptors generally evolve rapidly through gene duplication and loss [59], the number of genes within the LRC and NKC differs between species.

## Natural killer complex (NKC)

Marsupial NKC genes are generally orthologous to those in eutherians. However, the marsupial NKC is minimal [22, 50, 51] compared to monotremes and eutherians, both of which have large gene expansions within this NK receptor complex [59, 60]. 10-19 NKC genes were characterised in the woylie, wombat, antechinus and numbat genomes (Supplementary Table 4), a similar number to those previously identified in koala [50], devil [51] and opossum [22]. The NKC region was generally intact in the koala and wombat genome assemblies and was located across five scaffolds. This was not the case for woylie, antechinus or numbat, where the NKC region was highly fragmented across 11-12 scaffolds.

NKC genes containing C-type lectin domains were identified in all five marsupials and have an orthologous relationship to those in eutherians as evidenced by strong bootstrap support (Supplementary Figure 9). These include CLEC1A and 1B, varying numbers of the CLEC4 family and the marsupial-specific CLEC2-like which is not orthologous to eutherian NKC genes (supplementary figure 9). Two CTLD genes, CLEC4G and CLEC17A, not previously identified in marsupials were characterised in this study. In addition, two novel NKC genes were identified in the woylie, koala and numbat genome, named CLEC4-like1 and -like2. Both genes contained C-type lectin domains, and cluster with eutherian CLEC4K and 4F, yet have poor bootstrap support for an orthologous relationship (Supplementary Figure 9). Members of the killer cell lectin-like (KLR) family were also identified in woylie, wombat, antechinus and numbat (Supplementary Figure 9), and as previously published for koala [50]. Similar to CLEC genes, woylie, wombat, antechinus and numbat KLR genes were orthologous to those in eutherians, as observed in other marsupials [22, 50, 51]. Two KLR genes not previously identified in marsupials were identified, both of which have strong bootstrap support as orthologs of eutherian KLR genes; KLRF1 was characterised in all five species and KLRG2 in woylie and numbat (Supplementary Figure 9).

Supplementary Table 4. Number of NKC genes identified from the KLR and CLEC families in the koala, woylie, wombat, antechinus and numbat genomes, including partial sequences.

| **Number of genes** | **Koala** | **Woylie** | **Wombat** | **Antechinus** | **Numbat** |
| --- | --- | --- | --- | --- | --- |
| **KLR family** | 6 | 5 | 5 | 4 | 6 |
| **CLEC family** | 10 | 11 | 5 | 6 | 10 |


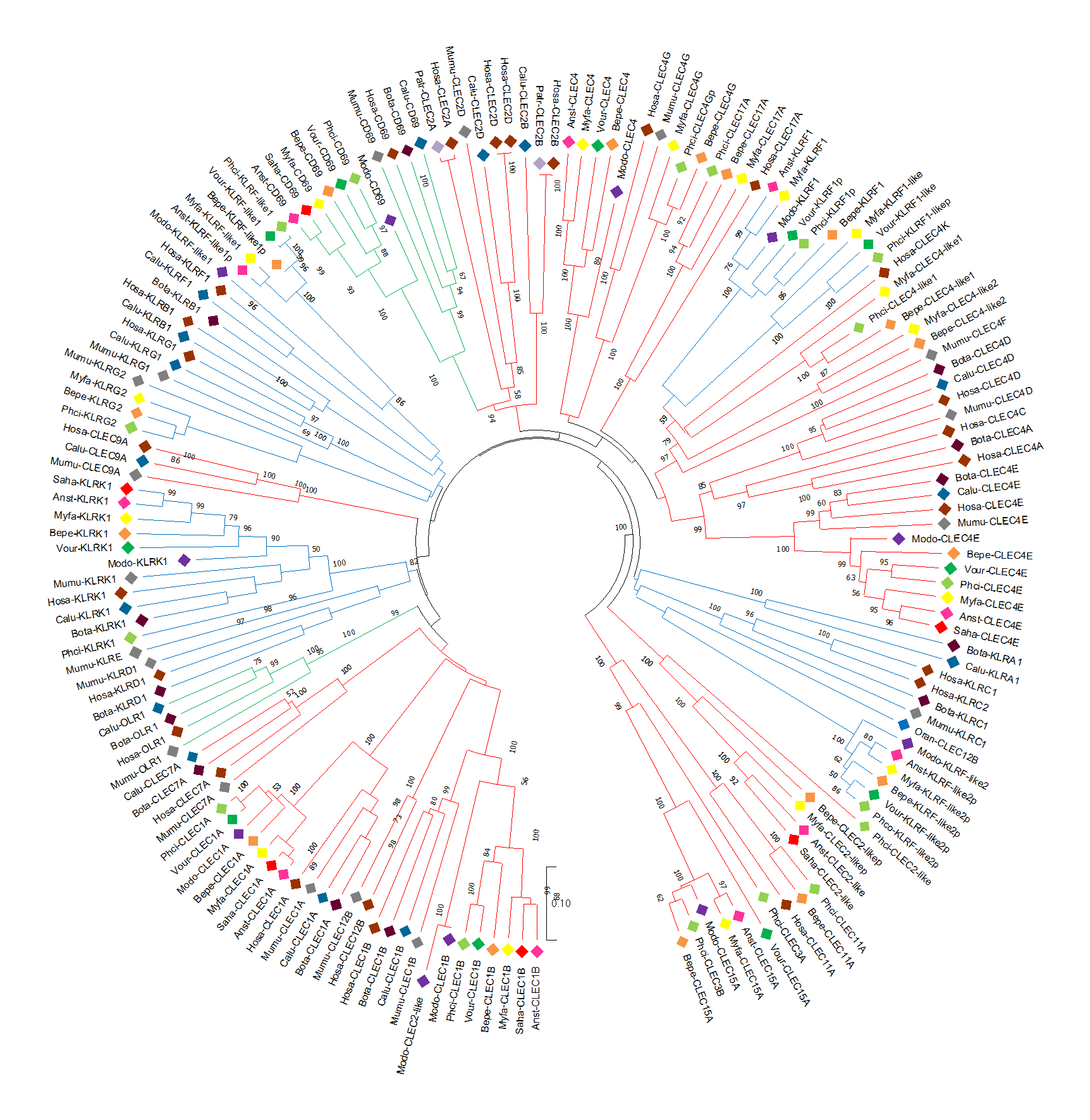
Supplementary Figure 9. Phylogenetic relationships amongst woylie (orange), antechinus (pink), wombat (dark green), koala (light green), numbat (yellow), opossum (purple), chimpanzee (lilac), cow (dark red), human (brown) and mouse (grey) NKC gene amino acid sequences. Branch colour indicates NKC receptor gene groups; killer cell lectin-like receptors (KLRs) are blue, C-type lectin receptors (CLEC) are red and other genes encoded within the NKC are green. The neighbour-joining phylogenetic tree was constructed in MEGAX using the p-distance method, pairwise deletion and 1000 bootstrap replicates. Bootstrap values less than 50% are not shown.

## Leukocyte receptor complex (LRC)

Unlike the NKC, marsupials have a large expansion of Ig-domain containing genes within the LRC [3, 22, 50, 51] compared to monotremes and eutherian mammals [59]. Similarly, an expansion of Ig LRC domains was identified in the woylie, wombat, antechinus and numbat genomes. This indicates these marsupial species also likely relied on NK receptors from the LRC family for activation and inhibition of NK cells. The number of unique Ig LRC domains was highest in the woylie with 111 identified, followed by antechinus 91, numbat 70 and wombat 55 (Supplementary Table 4). This is lower than opossum (154) [22] but higher than devil (24) [51], although genome quality is likely a factor in the annotation of devil Ig LRC domains. The Ig LRC domains identified in this study have been named according to the convention for devil Ig LRC domains [51]; woylie (BetIG), wombat (VourIG), antechinus (AnstIG) and numbat (MyfaIG). In addition to previously characterised LRC Ig domains in koala (KIG) [50, 61], we identified three new unique Ig LRC domains, bringing the total number in this species to 51. The Ig domains identified likely comprise from 18-40 LRC genes as outlined in Supplementary Table 5, as up to six Ig domains were encoded within a single LRC gene amongst the five species studied. The genomic organisation of the koala and woylie LRC was highly intact, as all genes were located on a single scaffold in koala and across five scaffolds in woylie (Supplementary Table 5). This is likely due to long-read PacBio sequencing used in the generation of these genomes, as long reads span the highly repetitive LRC region containing duplicate Ig domains, enabling resolution of this complex gene family. In comparison, the LRC in the wombat, antechinus and numbat genomes was highly fragmented, located across 16 to 34 scaffolds (Supplementary Table 5) with some Ig domains located on individual short scaffolds. As such, Ig domains may be encoded by non-functional pseudogenes, hence the true number of LRC NK receptors could not be resolved in these three species.

Within the genome, the LRC is flanked by a region known as the extended LRC that contains genes such as carcinoembryonic antigen-related cell adhesion molecules (CEACAMs) and sialic acid-binding Ig-like lectins (SIGLECs). The extended LRC region in the koala genome was highly intact, as CEACAM and SIGLEC genes were identified immediately adjacent to the LRC region on scaffold 2. A number of CEACAM and SIGLEC genes were identified in the wombat genome, interspersed amongst Ig domains on the longest scaffold encoding the LRC (scaffold 3). However, additional extended LRC genes were also identified on a number of short scaffolds. For woylie, antechinus and numbat, the extended LRC was fragmented, with genes identified on numerous scaffolds some of which also contained Ig LRC domains.

Marsupial LRC genes show little orthology to eutherian LRC genes, however a number of interesting relationships are evident from phylogenetic analyses. Species-specific expansions of Ig LRC domains were observed in koala, woylie, wombat, antechinus and numbat, in addition to marsupial orthologs, as noted previously in other marsupials [3, 22, 50, 51] (Supplementary Figure 10). These Ig LRC domains are not orthologous to eutherian MII type LRC domains such as KIR and LILR, which form a monophyletic clade in the phylogenetic tree. However, eutherian MI type LRC domains, such as the first Ig domain of LILR, LAIR, GPVI, FCAR and NCR1, are interspersed with marsupial Ig domains (Supplementary Figure 10). Furthermore, a putative GPVI ortholog previously identified in koala (KIG11), devil (DIG15) and opossum (LRC112) was also identified in numbat (MyfaIG29 & IG30) and woylie (BepeIG52). These Ig LRC domains form a clade with the first Ig domain of eutherian GPVI with high bootstrap support (99%) (Supplementary Figure 11).

Supplementary Table 5. LRC NK receptor genes identified in koala, woylie, wombat, antechinus and numbat genomes. The numbers in brackets indicate partial sequences.

|  | **Koala** | **Woylie** | **Wombat** | **Antechinus** | **Numbat** |
| --- | --- | --- | --- | --- | --- |
| **No. unique Ig domains** | 51 | 111 | 55 | 91 | 70 |
| **No. LRC genes** | 18 | 40 | 20 | 35 | 38 |
| **No. Ig domains encoded per LRC gene** | 1-4 | 1-6 | 1-4 | 1-5 | 1-5 |
| **No. scaffolds encoding LRC** | 1 | 5 | 16 | 34 | 33 |
| **No. extended LRC genes:**  **SIGLECs** | 2 (3) | 11 (8) | 3 (2) | 3 | 4 (1) |
| **CEACAMs** | 1 | 7 | 1 (3) | 3 (9) | 4 (2) |


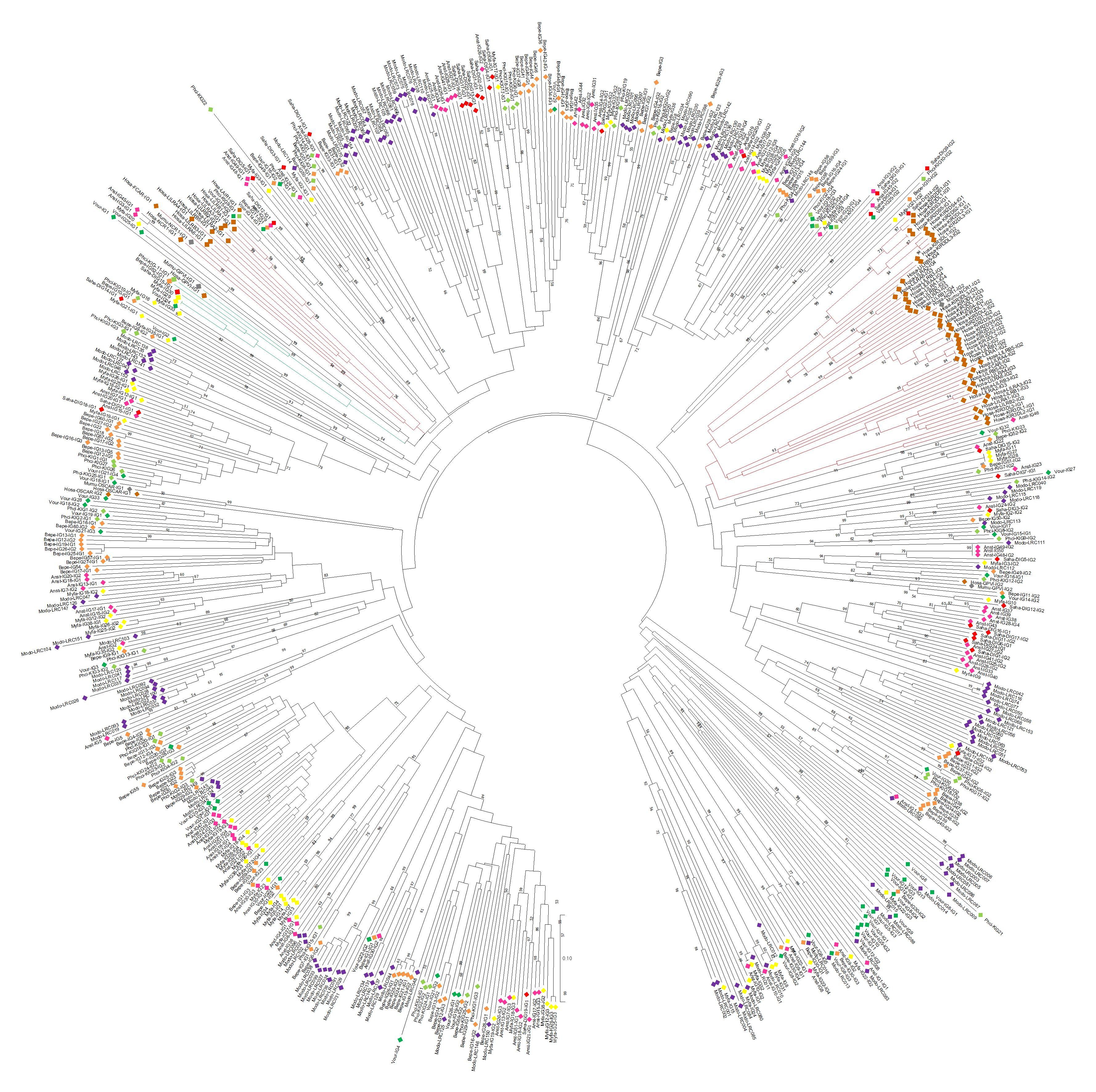
Supplementary Figure 10. Phylogenetic relationships amongst koala (light green), wombat (dark green), woylie (orange), numbat (yellow), antechinus (pink), devil (red), opossum (purple), human (brown) and mouse (grey) LRC domains. The majority of marsupial LRC domains are not orthologous to eutherian MI or MII type LRC domains (red branches). Clustering of putative marsupial LRC domains orthologous to the first IGSF domain of eutherian GPVI are indicated by the green branches. Neighbour-joining phylogenetic tree was constructed in MEGAX using the p-distance method, pairwise deletion and 1000 bootstrap replicates. Only bootstrap values greater than 50% are shown.


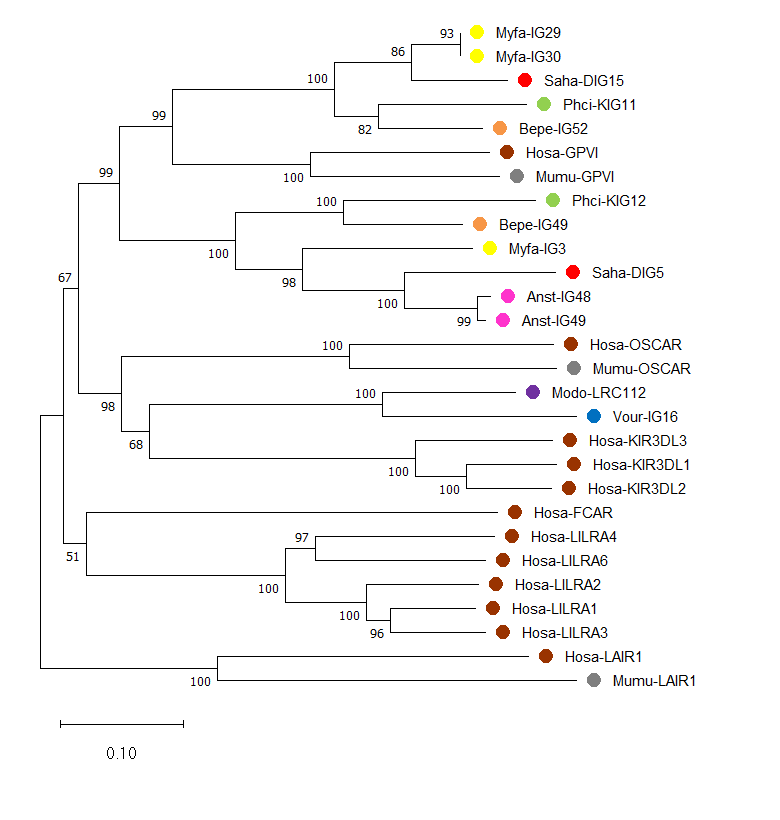
Supplementary Figure 11. Phylogenetic relationships amongst full-length marsupial and eutherian LRC genes, including putative marsupial GPVI orthologs in numbat (Myfa-IG29 & IG30, yellow) and woylie (Bepe-IG52, orange), which along with previously published orthologs in koala (Phci-KIG11, green) and Tasmanian devil (Saha-DIG15, red), cluster with eutherian GPVI with high bootstrap support. Additional marsupial IG domains that cluster with the second IG domain of GPVI in SX fig, form a separate clade, basal to that containing GPVI. Neighbour-joining phylogenetic tree was constructed in MEGAX using the p-distance method, pairwise deletion and 1000 bootstrap replicates. Only bootstrap values greater than 50% are shown.

# Cytokines

Cytokines are small signalling molecules secreted by numerous cell types which modulate the development, migration and function of immune cells. Cytokines are grouped into families including interferons (IFN), interleukins (IL), tumour necrosis factors (TNF) and transforming growth factors (TGF) amongst others. Each family will be discussed in further detail below.

## Interferons

Interferons (IFN) are one family of cytokines which are involved in anti-viral defence, anti-cancer immunity and immunomodulation. They are classified into three subclasses; type I, II and III.

Mammals have multiple type I IFN genes including α, β, δ, ε, ω and κ. IFNδ, ε and ω have not been identified in marsupials to date [10, 19, 31, 32], which was also the case for the five species in this study. IFNα is commonly duplicated in marsupials, with seven copies in the opossum [32] and four in the devil [10]. Similarly, IFNα was highly duplicated in the woylie (13), koala (13) and wombat (11), similar to the number in humans (13). Fewer copies of IFNα were identified in the antechinus (4) and numbat (2), although five potential IFNα pseudogenes were present in the numbat genome. Marsupial IFNα cluster within a separate clade to that containing eutherians, with clear marsupial orthologs evident (Supplementary Figure 12). A single copy of IFNβ has been identified in marsupials to date [10, 19, 32], which was also the case for woylie, wombat, antechinus and numbat (Supplementary Figure 12). Five copies of IFNβ were identified in the koala genome, all located within a 115KB region on scaffold 1 interspersed amongst multiple copies of IFNα. These copies have very short branch lengths in the phylogenetic tree (Supplementary Figure 12), hence are likely recent duplications. The final type I IFN identified in marsupials is IFNκ, with a single copy identified in opossum and thylacine [19, 32], but not in the devil likely due to genome assembly error [10]. A single IFNκ gene was also identified in the koala, woylie and wombat genomes, but was unable to be characterised in the antechinus or numbat likely due to genome fragmentation.

Type II IFN in mammals are represented by a single gene IFNγ, with single ortholog identified in marsupials [10, 19, 32] as was also the case for the five marsupials studied here. Type III IFN are similarly represented by a single gene in marsupials IFNλ [10], whereas humans have four genes (IFNλ-1 through 4) [62]. A single IFNλ was characterised in koala, woylie, wombat, antechinus and numbat.

Marsupial IFN cluster with those from eutherians with high bootstrap support, indicating and orthologous relationship. The species-specific expansions within IFNα and β genes in koala, woylie and wombat have short branch lengths, which suggests they likely arose through recent duplications (Supplementary Figure 12).

The genomic region encoding IFN in the koala was highly contiguous, with all genes located on a single scaffold flanked by interleukin and tumour necrosis factors. The region encoding IFN in the wombat genome was almost intact, with all but one gene located on a single scaffold similarly flanked by interleukins and tumour necrosis factors. IFNγ was located on a different scaffold, flanked by interleukins. This was not the case for the remaining three species, as IFN were encoded across multiple scaffolds (7 woylie, 4 numbat and 5 antechinus).


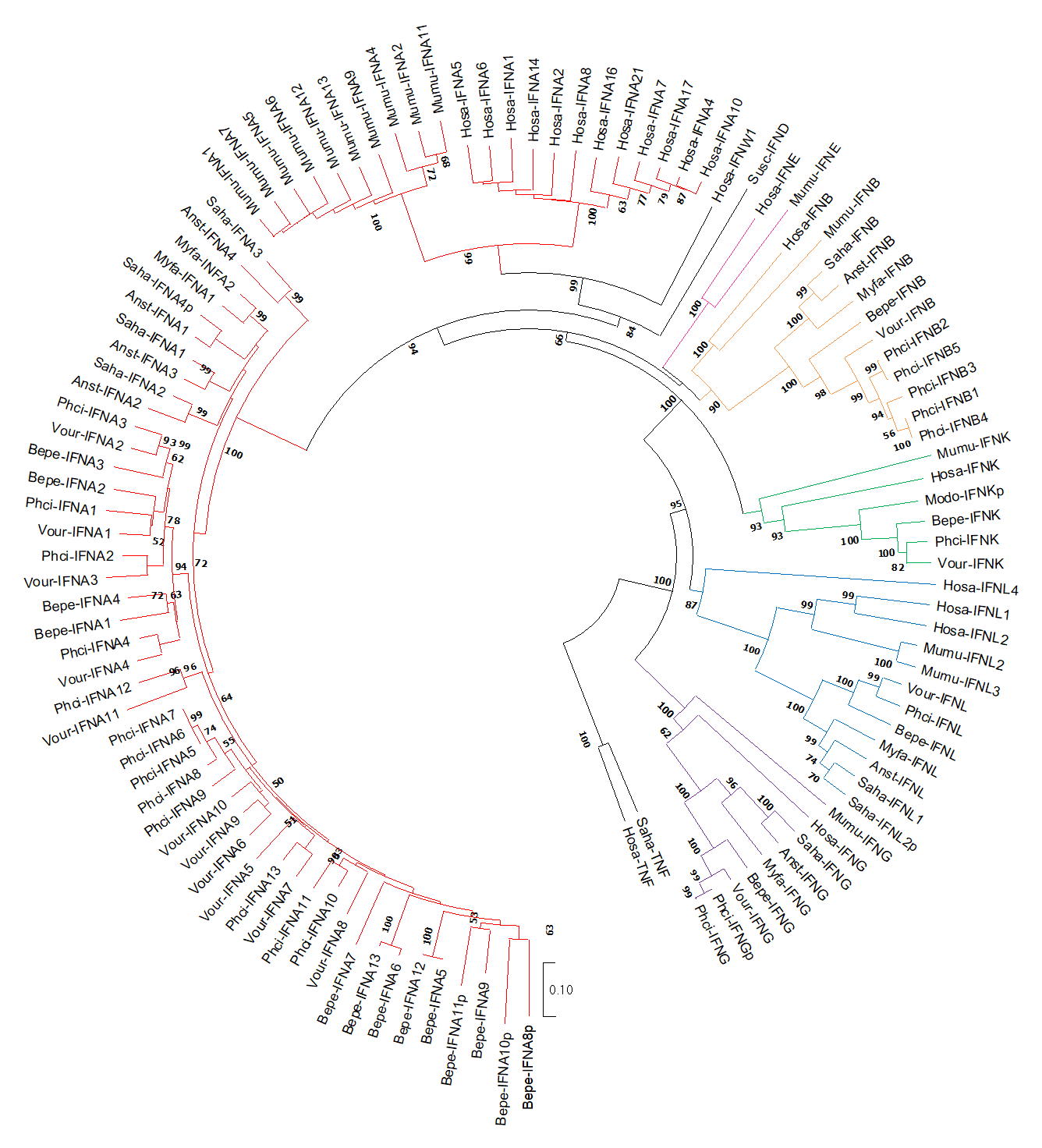
Supplementary Figure 12. Phylogenetic relationships amongst marsupial and eutherian interferons (IFN). Branches are coloured according to interferon gene family; IFNα (red), IFNε (pink), IFNβ (orange), IFNκ (green), IFNλ (blue), IFNγ (purple), and IFNω and IFNδ (black). Marsupial IFNα have undergone recent duplications in some lineages (koala, woylie and wombat) indicated by the short branch lengths, and cluster in a separate clade to eutherian IFNα with strong bootstrap support. The neighbour-joining phylogenetic tree was constructed in MEGAX using the p-distance method, pairwise deletion and 1000 bootstrap replicates. Bootstrap values less than 50% are not shown.

## Interleukins

Interleukins (IL) are cytokines secreted by immune cells which influence the development, chemotaxis and function of immune cell populations. Overall, 34 (koala and woylie) to 35 (wombat, antechinus and numbat) IL were characterised in each species, all of which were orthologous to eutherian IL evidenced by high bootstrap support within the phylogenetic tree (Supplementary Figure 13), as observed in other marsupials [10, 31, 33]. This included IL involved in Th1 innate (IL12A, IL12B, IL18 & IL27) and Th2 adaptive (IL9, IL4) immune responses, as well as all six members of the IL17 family (A, B, C, D, E and F), some of which have been characterised previously in the koala owing their involvement in chlamydia infection [33, 63, 64]. IL31 could not be identified in the koala or woylie genome, and IL3, IL32 and IL37 were not identified in any of the five species, as observed in other marsupials [10, 31, 32]

Marsupial-specific duplications have also evolved within the IL36, IL18 and IL22 families which have not been identified in eutherian mammals (Supplementary Figure 13). IL36L1 and IL36L2 in marsupials is related to eutherian IL36, and has been identified in the devil [10] and thylacine [19]. Both genes were characterised in all five species, except IL36L1 was a pseudogene in the current version of the koala genome. IL18 has also evolved through gene duplication in some marsupials, with three homologs (A, B and C) identified in the devil [10], two in the opossum [31] and one in the tammar wallaby as observed in humans. Three homologs were identified in all five marsupials studied here. Duplication of IL22 has only been documented in the devil, with 5 homologs identified [10]. Only a single IL22 gene was characterised in each of the five marsupials studied here.

IL genes were scattered throughout the genome in all five species, although was the most contiguous in the koala whereas they were encoded on 7 scaffolds interspersed with genes encoding other cytokines. IL genes were more fragmented in the remaining four species genomes, located on 26 scaffolds in the woylie, 12 in the wombat, 20 in the antechinus and 28 in the numbat.


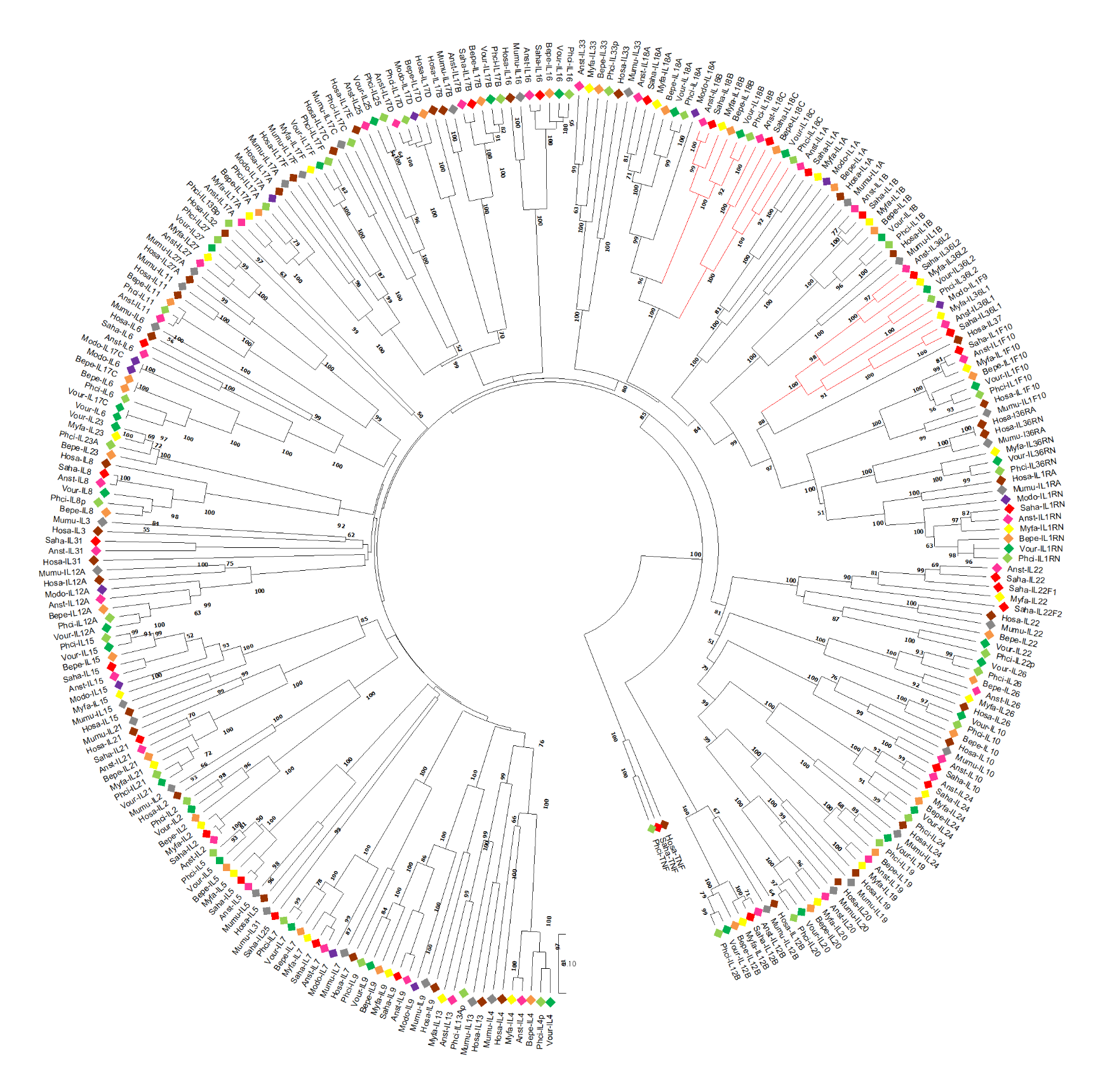
Supplementary Figure 13. Phylogenetic relationships amongst koala (light green), woylie (orange), wombat (dark green), antechinus (pink), numbat (yellow), Tasmanian devil (red), opossum (purple), human (brown) and mouse (grey) interleukins (IL). The majority of marsupial IL are orthologous to those in eutherians. Marsupial-specific interleukins which have not been identified in eutherians, such as multiple copies of IL18 (IL18B and IL18C), IL36L1 and IL36L2, are indicated by the red branches. The neighbour-joining phylogenetic tree was constructed in MEGAX using the p-distance method, pairwise deletion and 1000 bootstrap replicates. Bootstrap values less than 50% are not shown.

## Tumour necrosis factors and transforming growth factors

Tumour necrosis factors (TNF) are mainly expressed by immune cells and influence cell development and proliferation, chemotaxis and initiate immune activation. All but one marsupial TNF family member is orthologous to those in eutherians, as evidenced by the clustering and high bootstrap support within the phylogenetic tree (Supplementary Figure 14). 18 gene orthologs of the TNF family were identified in the koala, and 17 in the remaining four species as TNFSF12 could not be identified. Interestingly, TNFSF18 which is present in eutherians, platypus and some marsupials [10, 19], but absent from opossum [31], was present in all five species studied here. In addition, TNFSF10L was also identified in all five species, as observed in other marsupials [10, 19]. This gene is present in fish, birds and monotremes but as been lost in eutherian mammals throughout evolution [10]. The marsupial-specific gene TNFSF13L was first identified in opossum [31] and devil [10], and is not orthologous to eutherian TNF genes. TNFSF13L was identified in all five marsupials studied, and clusters within a sister clade to TNFSF13B in the phylogenetic tree (Supplementary Figure 14). Similar to other cytokine families, TNF genes were encoded on a number of scaffolds in the five marsupial genomes. In koala and wombat, TNF genes were located alongside other cytokine genes, while in the woylie, antechinus and numbat, they were encoded on multiple individual scaffolds.

TGFβ belongs to the transforming growth factor (TGF) family of cytokines and is expressed by numerous immune cell populations. TGFβ binds to receptors, initiating a signalling cascade which leads to immune activation, immune cell development and chemotaxis. There are three TGFβ subtypes in humans; TGFβ1, TGFβ2 and TGFβ3. All three subtypes were identified in the five marsupials studied and are orthologous to those in eutherians (Supplementary Figure 14). Each TGFβ gene was encoded on a different scaffold in the genomes of the five marsupials studied. In the case of koala and wombat, the TGFβ genes were located on scaffolds which also encoded other cytokines.


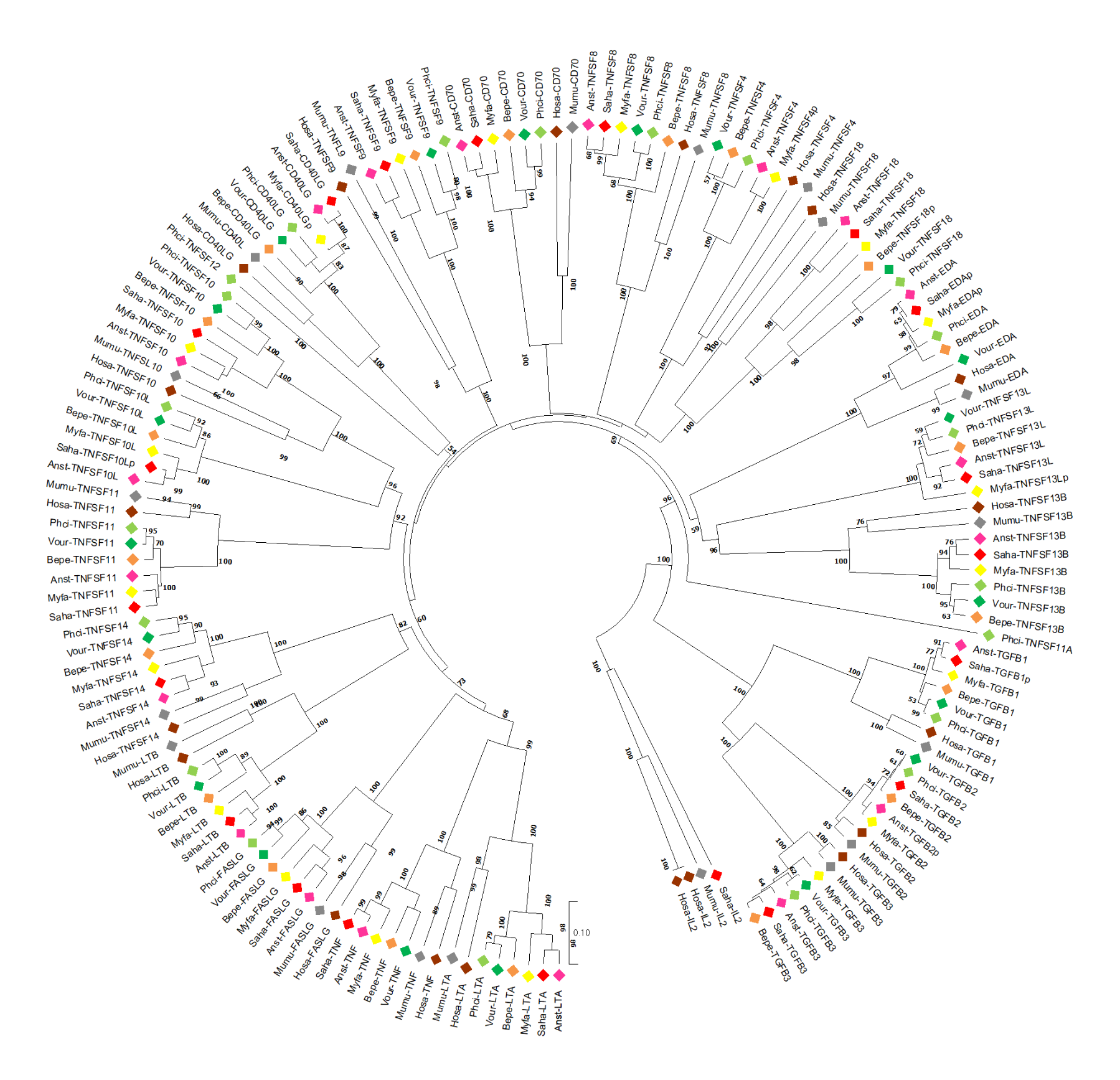
Supplementary Figure 14. Phylogenetic relationships amongst koala (light green), woylie (orange), wombat (dark green), antechinus (pink), numbat (yellow), Tasmanian devil (red), human (brown) and mouse (grey) tumour necrosis factors (TNF). Marsupial TNF are orthologous to those in eutherians. The neighbour-joining phylogenetic tree was constructed in MEGAX using the p-distance method, pairwise deletion and 1000 bootstrap replicates. Bootstrap values less than 50% are not shown.

# T cell receptors

T cell receptors (TCR) are expressed on the surface of T lymphocytes, where they recognise and bind MHC-bound antigens, resulting in activation of the adaptive immune response. There are four TCR chains conserved across jawed vertebrates (TCRα, β, γ and δ), all of which contain a constant region that defines the receptor type and anchors the peptide to the membrane, and a variable region that binds antigens. The gene encoding the constant region is relatively conserved across species, however some constant sequences have undergone duplications in some species. The variable region is constructed from variable (V), diversity (D) and joining (J) gene segments, which undergo V(D)J recombination to produce the significant TCR diversity required to recognise and bind a large number of antigens.

Marsupials differ to eutherian mammals in that they contain a fifth TCR chain, TCRμ. This is an ancient TCR chain which has been lost in eutherian mammals and contains several unique genetic features discussed below. The genomic organisation of TCR is highly conserved, with five TCR chains in marsupials (α, β, γ, δ and μ) encoded by four loci [3, 10, 14, 15, 17, 20, 65]. TRB, TRG and TRM loci encode the β, γ and μ chains respectively, while genes encoding α and δ chains are co-localised within the TRA/D locus. Each TCR locus is discussed in detail below.

## TRA/D

The genomic organisation of gene segments encoding the TCRα and TCRδ chains differs to other TCR loci in that they are co-localised within the genome of eutherians, birds, monotremes and marsupials [7, 17, 66-68]. This was also the case for the five marsupial genomes in this study, as the TRA/D locus ranged in size from 404kb (numbat) to 1.09Mb (koala) (Supplementary Table 6). The koala, wombat and antechinus TRA/D locus was similar in size to opossum (1.3Mb) [17], human (1Mb) [67] and mouse (1.65Mb) [69]. The small TRA/D locus characterised in the woylie and numbat genomes was likely due to fragmentation of the locus across multiple scaffolds (Supplementary Table 6). The genomic region encoding the TRA/D locus displays a high degree of synteny across mammals [17]. Genes flanking the 5’ and 3’ end of the locus were identified in woylie, wombat and antechinus, similar to opossum [17] and as previously characterised for koala [7]. The genes *methyl-transferase like 3* (*METTL3*) and *zinc finger protein* (*SALL2*) flanked the 5’ end, *while defender against cell death gene 1* (DAD1) and *abhydrolase domain-containing protein 4 gene* (ABHD4) flanked the 3’ end. Genes flanking the TRA/D locus in marsupials were not associated with the TRA/D locus in the current numbat genome, however this is likely due to fragmentation and assembly error. An additional conserved feature of the TRA/D locus is the presence of an inverted V gene segment downstream of Cδ [17]. This inverted V segment was also identified in koala, wombat and antechinus, however fragmentation of the locus prevented identified in numbat and woylie. These marsupial inverted V segments are orthologous to those in human and mouse, forming a separate clade (group B) within the phylogenetic tree with strong bootstrap support (100%) as observed previously in opossum [17] (Supplementary Figure 15).

The koala TRA/D locus has been characterised previously [7], and is similarly complete in the current version of the genome used in this study. The wombat and antechinus TRA/D loci were also complete, with Cα and Cδ sequences encoded upstream of multiple V gene segments, located on a single scaffold. The woylie TRA/D locus was located at the end of two large scaffolds (scaffold 50 6.4Mb and scaffold 649 2.9Mb); one encoded Cα and Cδ upstream of multiple V segments, while the other encoded only V segments. The numbat TRA/D locus was highly fragmented across six scaffolds; Cα, Cδ and multiple V gene segments were encoded on three large scaffolds (3.1 to 4.7Mb), with additional V segments located on individual short scaffolds (1 to 16KB).

A single Cα and Cδ gene was identified in woylie, wombat, antechinus and numbat (Supplementary Table 6), as characterised previously in the koala [1, 7], other marsupials [15, 17, 19] and eutherians [69] (Supplementary Figure 8). All Cα and Cδ sequences contained functional domains and motifs identified in other mammals [17], including a conserved lysine residue within the transmembrane domain that is involved in dimerization with other TCR chains [70].

Overall a high number of TRA/D V gene segments were identified in the five marsupial species studied here (51-76), similar to opossum (60), human (47-50) and mouse (78-89) [69] (Supplementary Table 6). The exact number of TRA/D V segments could not be confirmed in woylie and numbat due to fragmentation of the locus. TRA/D V gene segments from all five species in this study cluster in eight groups (A through H) within the phylogenetic tree (Supplementary Figure 16) as previously defined by [17]. All groups except B contain sequences from eutherians, marsupials and monotremes, indicating these V groups were present in the mammalian ancestor.

Supplementary Table 6. TCR constant (C) and variable (V) gene segments with complete coding sequences (excluding pseudogenes) identified in the koala, woylie, wombat, antechinus and numbat genomes. * indicates the locus was fragmented over multiple scaffolds, in which case the longest scaffold containing both constant and variable sequences is provided.

|  | **TRA/D** | | | **TRB** | | | **TRG** | | | **TRM** | | | |
| --- | --- | --- | --- | --- | --- | --- | --- | --- | --- | --- | --- | --- | --- |
|  | **C** | **V** | **Locus** | **C** | **V** | **Locus** | **C** | **V** | **Locus** | **C** | **V** | **Vj** | **Locus** |
| **Koala** | 1 Cα & 1 Cδ | 59 | Scaf 7 1.09Mb | 3 | 34 | Scaf 5  383kb | 1 | 5 | Scaf 1  65kb | 4 | 3 | 3 | Scaf1  397kb |
| **Woylie** | 1 Cα & 1 Cδ | 76 | Scaf 649 537kb* | 3 | 26 | Scaf 838  477kb | 1 | 12 | Scaf 131  220kb | 6 | 4 | 4 | Scaf 918  828kb* |
| **Wombat** | 1 Cα & 1 Cδ | 57 | Scaf 2 946kb | 3 | 17 | Scaf 5 380kb | 1 | 6 | Scaf 1 40kb* | 4 | 4 | 3 | Scaf 1  379kb* |
| **Antechinus** | 1 Cα & 1 Cδ | 57 | Scaf 143  1.05Mb | 3 | 46 | Scaf 333 490kb* | 1 | 13 | Scaf 366  72kb* | 5 | 5 | 5 | Scaf 358  211kb* |
| **Numbat** | 1 Cα & 1 Cδ | 51 | Scaf 1536  404kb* | 3 | 34 | Scaf 6578 258kb* | 1 | 12 | Scaf 3245 87kb* | 3 | 3 | 4 | Scaf 279091  14kb* |


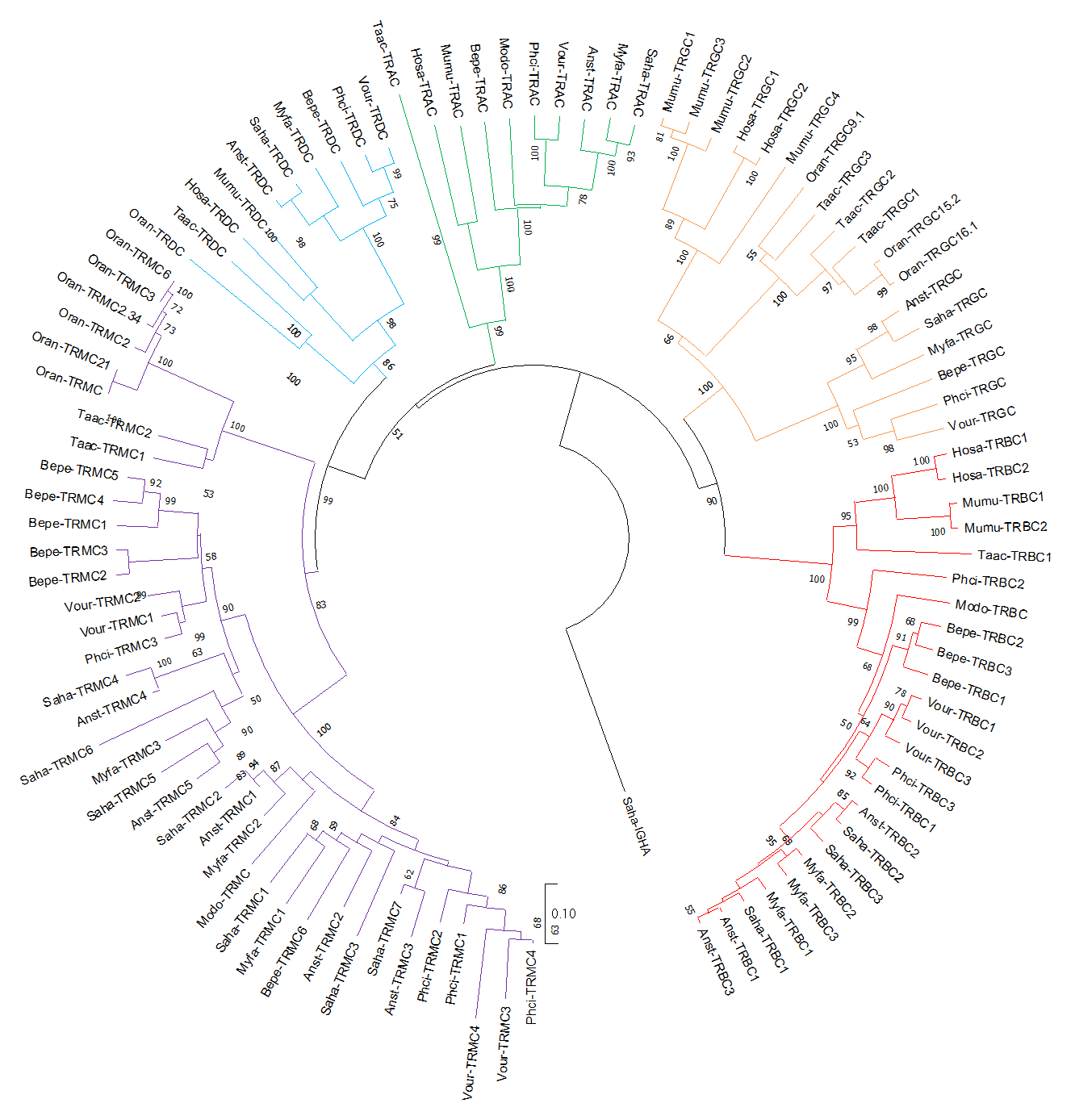


Supplementary Figure 15. Phylogenetic relationships amongst woylie, antechinus, wombat, numbat, koala, other marsupial, monotreme and eutherian T cell receptor (TCR) constant amino acid sequences. Colours indicate TCR chain; Cα (green), Cδ (blue), Cβ (red), Cγ (orange) and Cμ (purple). The neighbour-joining phylogenetic tree was constructed in MEGAX using the p-distance method, pairwise deletion and 1000 bootstrap replicates. Bootstrap values less than 50% are not shown.

Supplementary Figure 16. Phylogenetic relationships amongst brushtail bettong (orange), antechinus (pink), wombat (dark green), numbat (yellow), koala (light green), tammar wallaby (teal), platypus (dark blue), echidna (light blue), sheep (dark red), human (brown) and mouse (grey) TRA/D V segments. The eight V groups according to [17] are indicated by the branch colours; A (red), B (orange), C (dark blue), D (pink), E (light green), F (dark green), G (purple) and H (light blue). The neighbour-joining phylogenetic tree was constructed in MEGAX using the p-distance method, pairwise deletion and 1000 bootstrap replicates. Bootstrap values less than 50% are not shown.


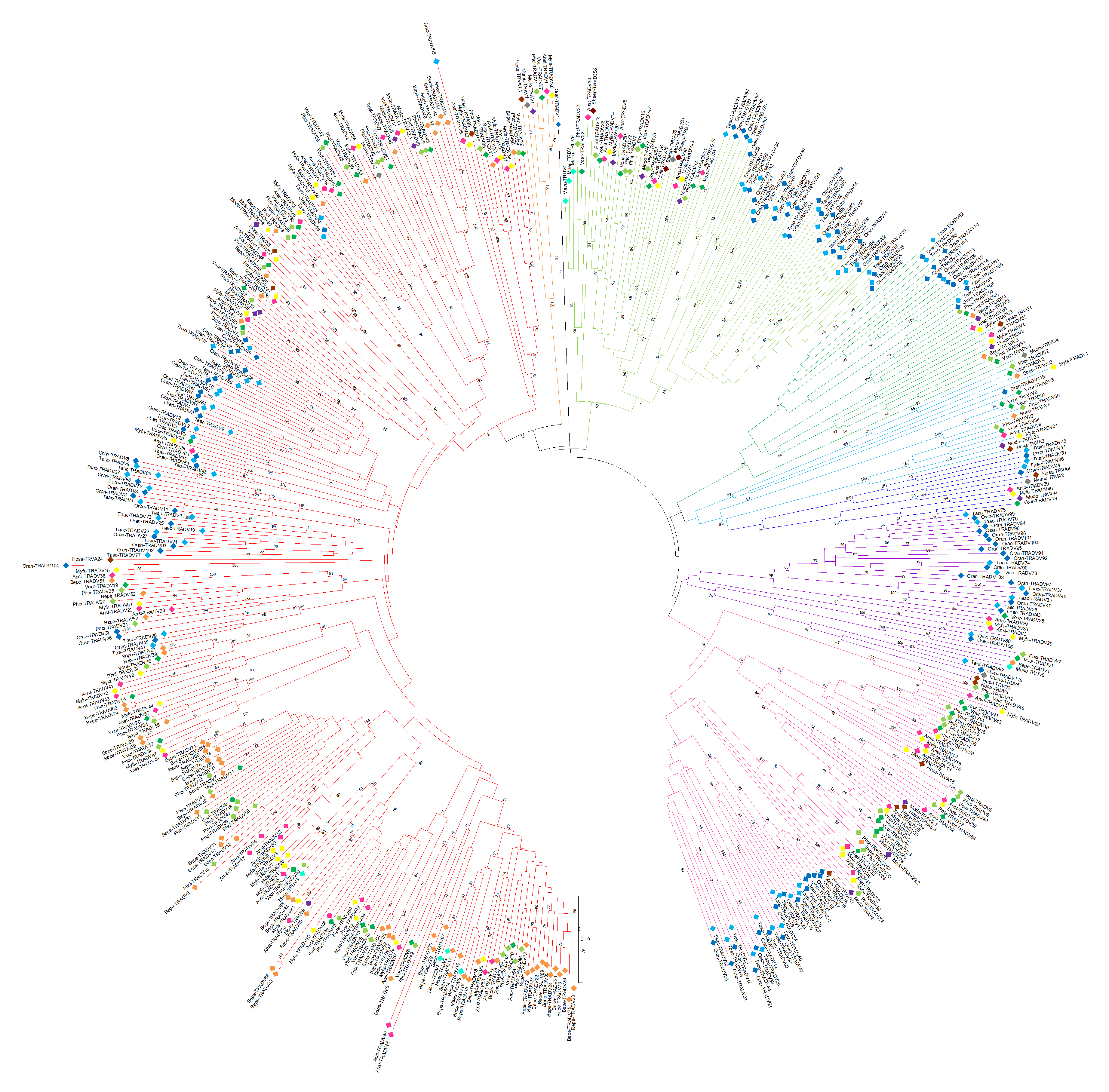


**B**

**E**

**F**

**H**

**C**

**G**

**D**

**A**

## TRB

The TRB locus that encodes the Cβ, Vβ, Dβ and Jβ gene segments which form the TCRβ chain ranged in size from 205KB (numbat) to 490KB (antechinus) amongst the five marsupials in this study (Supplementary Table 6). This is similar in size to the opossum TRB locus (400KB) [17] but smaller than humans and mice (both ~650KB) [69, 71]. The genomic organisation of the TRB locus is highly conserved, with Vβ gene segments located upstream of Cβ sequences [17]. The region surrounding the TRB locus also displays conserved synteny in mammals and chickens [17], and is flanked by *kell blood group glycoprotein* (KEL) and *ephrin type-b receptor 6 precursor* (EPHB6) genes at the 3’ end and a variable number of *mono-oxygenase DBH-like 2* (DBHL2) genes at the 5’ end. In addition, multiple copies of *trypsinogen* (TRY) are interspersed throughout the locus. This organisation is conserved across human, mouse, cow, chicken and opossum [17], and was also the case in the five marsupial genomes studied here.

The complete koala TRB locus has been characterised previously [7], and is similarly complete in the current version of the genome examined in this study. The woylie and wombat TRB loci were also complete, with Cβ, Vβ and flanking genes encoded on a single scaffold. This was not the case for the TRB loci in the antechinus and numbat genomes, which were both highly fragmented. In the case of the antechinus, Cβ, Vβ and flanking genes were encoded on a single scaffold, however additional Vβ gene segments were located on individual short scaffolds of up to 7KB in length. Similarly, numbat Vβ and flanking genes were located on a single long scaffold, with multiple Cβ located on a different scaffold, and additional Vβ and flanking genes located on individual scaffolds.

A defining feature of the TRB locus in mammals and chickens is the presence of a Vβ gene segment at the 3’ end of the locus that is in the opposite orientation to the other gene segments [17]. This has previously been identified in opossum [17] and koala [17], but was not characterised in the woylie despite high sequence contiguity within this region of the genome. An inverted Vβ was similarly not identified in the wombat and numbat genomes. While the entire wombat TRB locus is encoded on a single scaffold, half the locus is encoded in the reverse orientation, while the other in the forward. This unusual feature is likely an artifact of assembly or HiC scaffolding and makes identification of an inverted Vβ gene segment difficult. However, Vour-TRBV15 clusters with the inverted human TRBV30 and mouse TRVB31 in the phylogenetic tree (Supplementary Figure 17), hence may be present in wombats. In the case of the numbat, the TRB was too fragmented to determine the presence of an inverted Vβ segment.

The Cβ sequence is often duplicated in mammals, with two to five copies identified in marsupials studied to date [3, 10, 15, 17]. Three Cβ were identified in all five species, as characterised previously for the koala [7] (Supplementary Figure 15). All Cβ contained conserved domains and motifs identified in other marsupials. Three conserved cysteine residues were identified, two of which for an intradomain disulfide bond, and the third forms a dimer with the α TCR chain [72]. A conserved lysine was also present, essential for CD3 interaction [73], as well as the conserved antigen receptor transmembrane (CART) motif which is involved in assembling and signaling of the TCR/CD3 complex [74].

A high number of Vβ segments were identified in all five species (Supplementary Table 6), similar to opossum (27) [17] and mouse (21-22) [69]. Phylogenetic analysis suggests marsupial Vβ segments are very diverse, with sequences from all five species in this study clustering within six groups first defined by Parra et al (2008) for opossum (A through F) (Supplementary Figure 17). The majority of Vβ segments from all five species cluster in group A and E, however a high number of those from dasyurids (antechinus and numbat) cluster in the B group. All six groups within the phylogeny contain Vβ from eutherian, marsupial and monotreme species, indicating these sequences arose prior to divergence of these mammalian orders.

Supplementary Figure 17. Phylogenetic relationships amongst brushtail bettong (orange), antechinus (pink), wombat (dark green), numbat (yellow), koala (light green), tammar wallaby (teal), platypus (dark blue), echidna (light blue), cow (dark purple), sheep (teal), human (brown) and mouse (grey) TRB V segments. The seven groups identified by [75] are indicated by the branch colours; A (red), B (orange), D (pink), E (light green), F (dark green), mouse (teal) and chicken (brown). The neighbour-joining phylogenetic tree was constructed in MEGAX using the p-distance method, pairwise deletion and 1000 bootstrap replicates. Bootstrap values less than 50% are not shown.


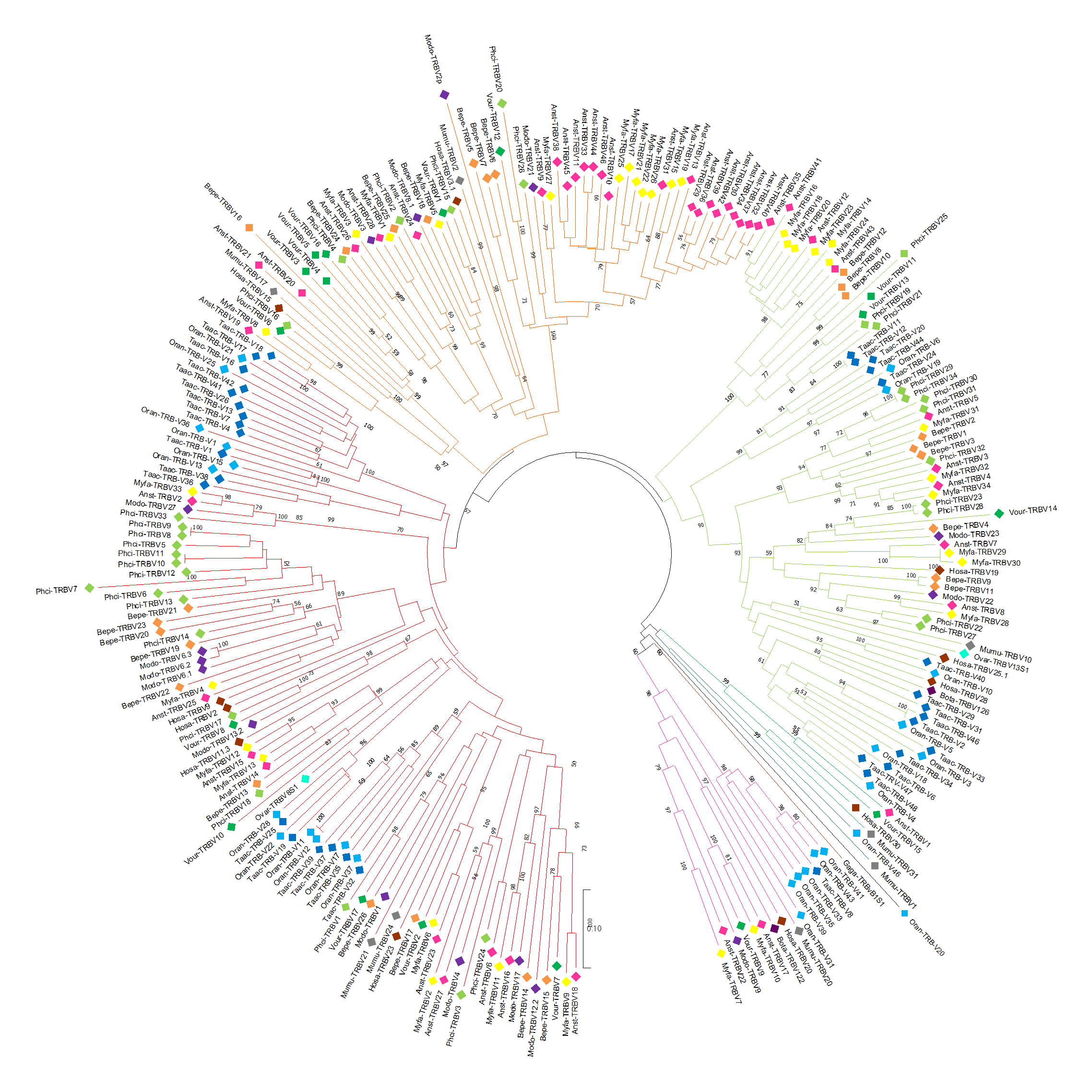


**Mouse**

**Chicken**

**D**

**A**

**B**

**E**

**F**

## TRG

The TRG locus that encodes the Cγ, Vγ, Dγ and Jγ gene segments which form the TCRγ chain ranged in size from 70KB (antechinus) to 220 KB (woylie) amongst the five marsupials in this study, within the range of opossum (90KB) [17] and mouse (205KB) [76]. Genes flanking the TRG locus are conserved amongst marsupials studied date, with *amphiphysin* (AMPH) flanking the 5’ end and *related to steroidogenic acute regulatory protein D3-N-terminal like* (STARD3nl) flanking the 3’ end of the locus, in the orientation of transcription [17]. Both AMPH & STARD3nl also flank the TRG locus in the current human (GRCh38.p13) and mouse (GRCm38.p6) genome assemblies on Ensembl. These flanking genes were used to determine if the TRG locus was intact (encoded on a single scaffold) in the koala, woylie, wombat, antechinus and numbat genomes. The complete koala TRG locus was initially characterised by Johnson et al (2018) and was similarly complete in the current updated version of the genome used in this study, encoded on a single scaffold. The woylie TRG locus was also complete, with flanking genes identified at the 5’ and ‘3 end of the locus. However, the TRG locus in the wombat, antechinus and numbat genome was fragmented, with C, V and flanking genes located on a single scaffold, and additional V gene segments located on different individual scaffolds (Supplementary Table 6).

A single Cγ gene was characterised in the koala, woylie, wombat, antechinus and numbat genomes, as observed in other marsupials [3, 17] and characterised previously in koala [7]. This differs to eutherians such as humans and mice, and monotremes such as the platypus [77], which have multiple copies of Cγ (Supplementary Figure 15). Cγ genes in the five marsupials in this study all contained functional domains and motifs, including a lysine residue within the transmembrane domain that is essential for interaction with CD3γ. Similar to monotremes [77] and other marsupials [17], Cγ also lacked the second cysteine residue involved in intradomain disulfide bond formation.

The five species studied here have a similar number of Vγ gene segments to other marsupials [17], and fewer than the expansion identified in monotremes [66] (Supplementary Table 6). The phylogenetic relationship of Vγ gene segments initially clustered into eight groups based on studies in eutherian mammals; A through F, I and H. Characterisation of opossum Vγ introduced an additional two groups [17]; J and “opossum”. Vγ gene segments from the five species in this study cluster within the two marsupial-specific groups J and K (previously “opossum”), both supported by high bootstrap values within the phylogenetic tree (Supplementary Figure 18). In addition, groups A and I contain Vγ gene segments from eutherians, marsupials and monotremes, including the species in this study, which indicates this may represent an ancient Vγ group. Vγ sequences from the five species in this study do not cluster within the monotreme-specific G group, that contains the large expansion of Vγ gene segments in platypus and echidna (Supplementary Figure 18).

Supplementary Figure 18. Phylogenetic relationships amongst brushtail bettong (orange), antechinus (pink), wombat (dark green), numbat (yellow), koala (light green), tammar wallaby (teal), platypus (dark blue), echidna (light blue), cow (dark purple), sheep (teal), human (brown) and mouse (grey) TRG V segments. The ten groups identified by [75]are indicated by the branch colours; A (red), C (dark blue), D (pink), E (light green), F (dark green), G (purple), J (teal), K (brown) and cow-sheep (black). The neighbour-joining phylogenetic tree was constructed in MEGAX using the p-distance method, pairwise deletion and 1000 bootstrap replicates. Bootstrap values less than 50% are not shown


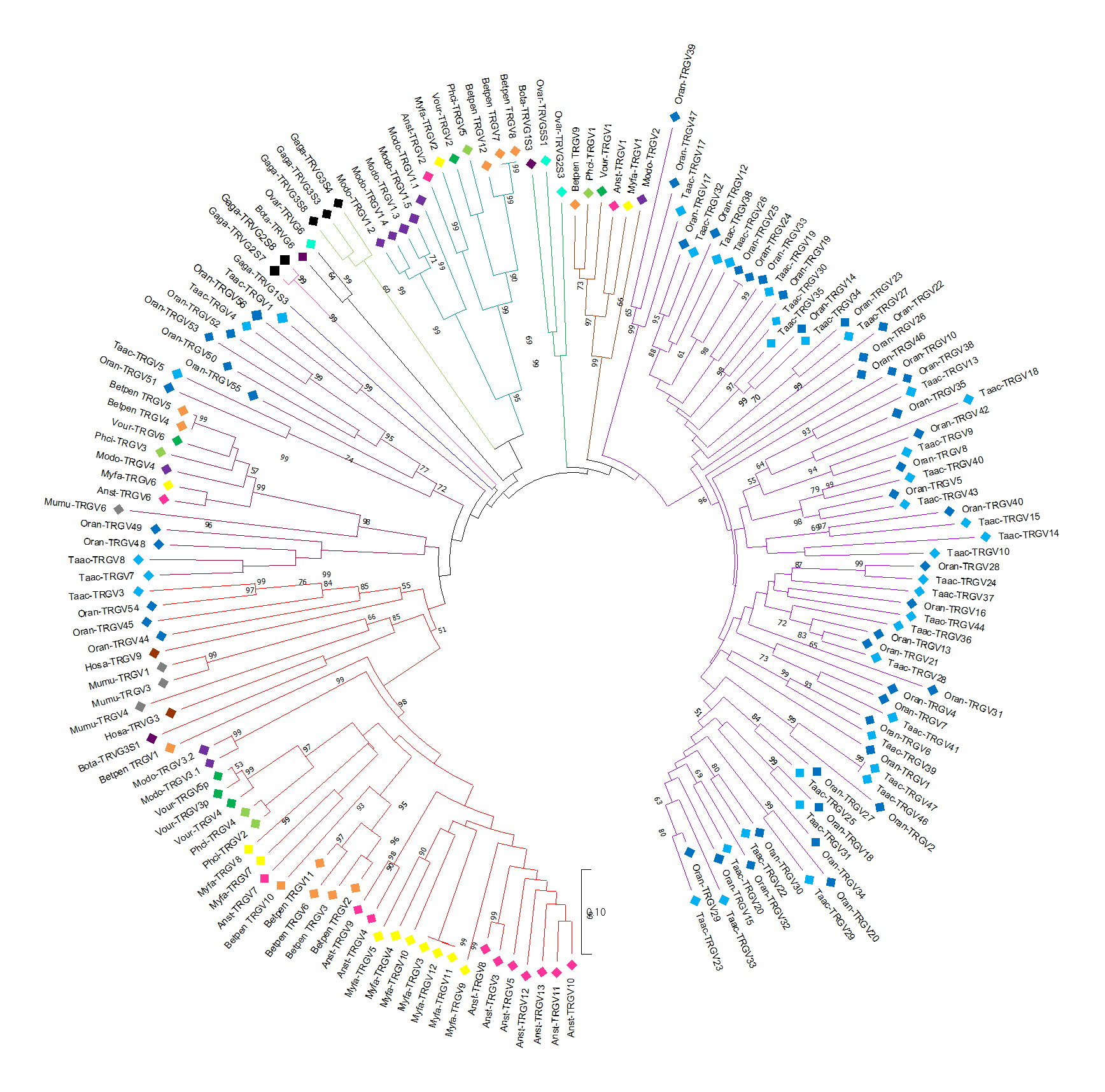


**A**

**I**

**C**

**D**

**Cow-sheep**

**E**

**J**

**K**

**G**

**F**

## TRM

Marsupials and monotremes are unique in that they contain a fifth TCR chain, TCRμ. This was first discovered in the northern-brown bandicoot [16, 20] and has since been identified in all other marsupials and monotremes studied to date [19, 78]. TCRμ is an ancient TCR chain that has been lost in eutherians and non-mammals throughout evolution [78]. TCRμ differs to other TCR chains in that it contains the only known germline-joined V, D and J segments, Vμj, which does not undergo V(D)J recombination and is encoded by a single exon upstream of Cμ [20]. TCRμ is also expressed in two isoforms; TCRμ1.0 and TCRμ2.0 [20]. TCRμ1.0 contains a single constant (Cμ) and variable domain encoded by Vμj, is primarily expressed in the thymus [79] and likely has an immune-related function other than antigen binding as the V domain is thought to be non-functional [20, 78]. TCRμ2.0 contains a single Cμ and double V domains, one encoded by Vμj and the other by Vμ which undergoes V(D)J recombination similar to V domains from other TCR chains [20].

The TRM locus is organised in tandem cassettes, each containing Vμ and/or Vμj, Dμ and Jμ segments upstream of Cμ. The number of cassettes differs amongst marsupials, with eight identified in the opossum genome [17]. From 4 (koala) to 10 (woylie) complete cassettes were identified amongst the five marsupials in this study, with a high number of partial cassettes identified in all species except koala and wombat. The TRM locus was encoded on a single scaffold in the wombat and koala genomes, however was fragmented across two scaffolds in the antechinus and three in the numbat and woylie. A high level of pseudogenization was observed within the TRM locus in all five species, as also identified in opossum [17].

Unlike other TCR loci, the genomic region encoding the TRM locus is not well conserved amongst mammals [17]. Annotation of TRM in the koala, woylie, wombat, antechinus and numbat genomes confirm that this region is also not well conserved amongst marsupials. The 5’ end of the TRM locus in opossum is flanked by *zinc finger protein* genes (ZFN3) which are similar to ZFN3 genes on several chromosomes in the human and mouse genome [17]. ZFN3 copies were associated with the TRM locus in koala, antechinus and woylie, with 7, 8 and 1 copy identified respectively. This was not the case in wombat and numbat, as one and two copies were identified on scaffolds other than that encoding TRM in both species. The genes *speckle type POZ-like protein* (PCIF1-like) and m*yelin oligodendrocyte glycoprotein* (MOG) flanked the 3’ end of the TRM locus in opossum, with paralogous copies located elsewhere within the genome [17]. PCIF1-like and MOG were not located adjacent to the TRM locus in any of the five species studied and were encoded on different scaffolds.

Cμ sequences are highly duplicated amongst marsupials, with seven in the devil [10] 8 in the opossum [17] and four in the thylacine [19]. Similarly, the number of functional Cμ sequences (those with complete open reading frames) identified ranged from three (numbat) to six (woylie) (Supplementary Table 6). Cμ sequences from all marsupials and monotremes cluster within a clade sister to that containing Cδ (Supplementary Figure 15), supporting the evolutionary hypothesis that TCRμ is derived from TCRδ.

A moderate number of Vμ gene segments were identified within the five marsupials studied, compared to the number encoded within the other four TCR loci (Supplementary Table 6). The number of functional Vμ (complete open reading frames) ranged from 3 (koala) to 5 (numbat), while Vμj also ranged from 3 (wombat) to 5 (antechinus) (Supplementary Table 5). Marsupial and monotreme Vμ and Vμj cluster segments form a sister clade to immunoglobulin heavy chain V segments with high bootstrap support, separate from other TCR chain V segments as identified previously (Supplementary Figure 19). This clustering provides insight into the evolutionary history of TCRμ, as Vμ and Vμj segments are likely derived from the ancestor of immunoglobulin and TCR V segments [17, 20, 78].


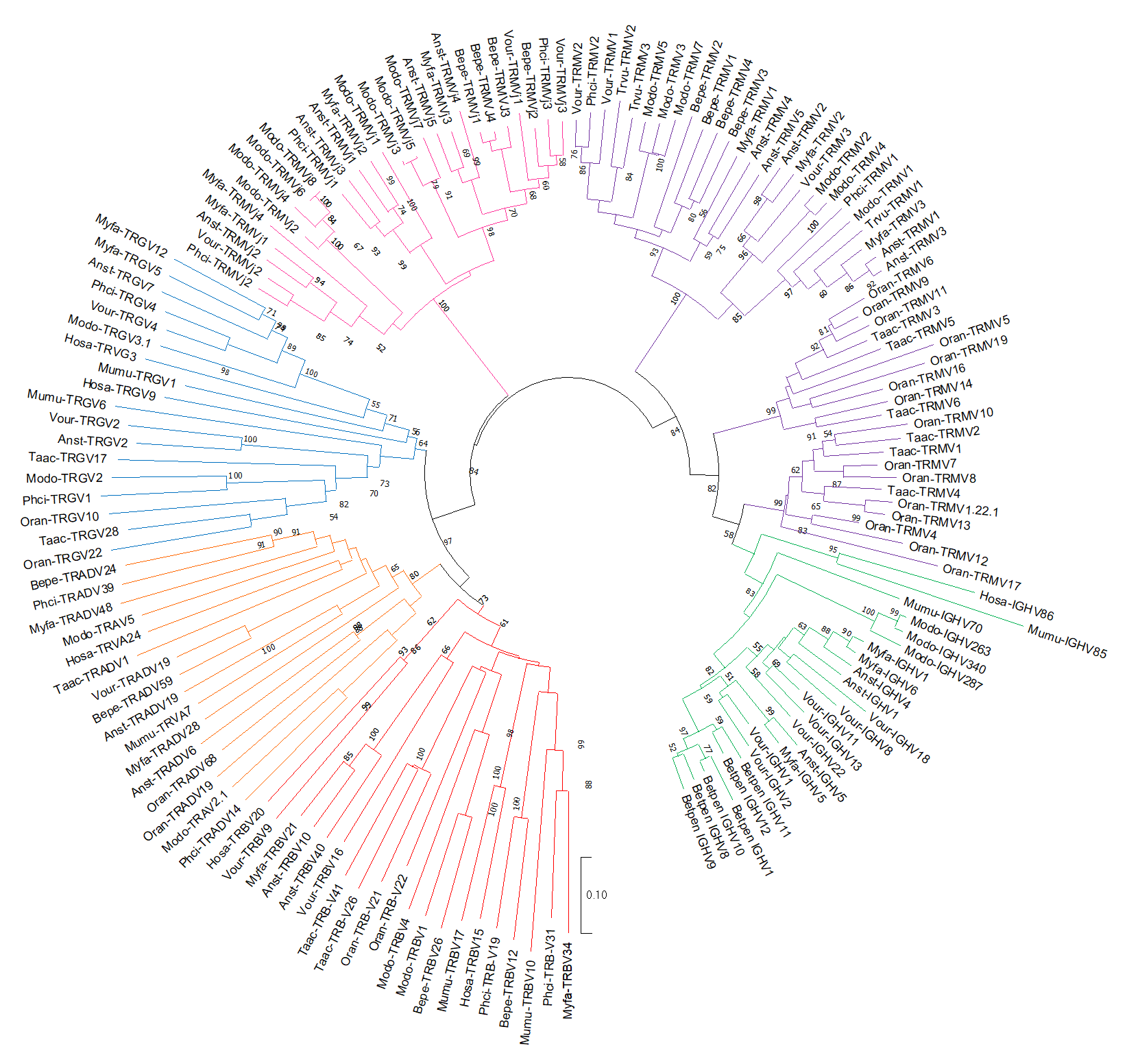
Supplementary Figure 19. Phylogenetic relationships amongst marsupial and monotreme Vμ (pink branches) and Vμj (purple branches), in relation to TCR Vα/δ (red), Vγ (blue), Vβ (orange), as well as immunoglobulin heavy chain V segments (green) from marsupials, monotremes and eutherian mammals. The neighbour joining phylogenetic tree was constructed in MEGAX using the p-distance method, pairwise deletion and 1000 bootstrap replicates. Bootstrap values less than 50% are not shown.

# Immunoglobulins

Immunoglobulins are major proteins involved in the humoral immune response and consist of two heavy chains and two light chains [6]. The constant region of the immunoglobulin heavy chains (Cμ, Cγ, Cα, Cε or Cδ) determine the class (IgM, IgG, IgA, IgE or IgD) [9]. The IgD class of immunoglobulins has not been identified within any marsupial studied to date [1, 3, 5, 19]. Cμ and Cα chains consists of 4 domains, whereas Cγ and Cε chains contain 3 domains and a hinge region. All four constant regions were identified in woylie, wombat, antechinus and numbat species, as well previously characterised koala constant sequences re-mapped to the current koala genome [1]. Apart from the numbat, all constant sequences were contained on a single scaffold. Interestingly, three copies of both Cμ and Cγ were identified in the woylie. Duplication of Cμ and Cγ has not been identified in other marsupials, although is common in eutherian mammals. Additional investigation is required to determine if these duplications in woylie are true or pseudogenes.

Similar to eutherian mammals, marsupials have two immunoglobulin light chain constant genes; Cκ and Cλ [3, 13, 19, 80]. Cλ sequences were identified in all five species, ranging from one copy (numbat) to 10 copies (woylie) (Supplementary Table 7). Similarly, the number of Cκ differed amongst marsupials in this study; with a single copy in the koala, antechinus and numbat genomes, two copies in the woylie genome and 4 partial copies in the wombat genome (Supplementary Table 7, Supplementary Figure 20).

When the immunoglobulin molecule is expressed on the surface of B lymphocytes, or secreted as antibodies, attached to each heavy chain constant region is a V-D-J unit encoded by variable (V), diversity (D) and joining (J) gene segments [81]. There are numerous copies of each V, D and J segment, which following recombination, give rise to the immense diversity observed within immunoglobulin molecules. The genomic organisation of the immunoglobulin locus is highly complex, with multiple V, D and J gene segments located upstream of constant sequences within the genome. Given these repetitive sequences, genome assemblers often struggle to resolve the region encoding immunoglobulins without manual curation and annotation. This was certainly the case for the five marsupial genomes examined in this study, with all but the koala and woylie immunoglobulin locus highly fragmented across multiple scaffolds. Only five V segments were identified in the antechinus genome, and six in the numbat genome, located across four and five scaffolds respectively. A higher number of V segments were identified in the wombat genome, with 22 V segments located on 19 scaffolds, three of these located on the same scaffold as the IGH constant genes. 31 V segments were identified in the woylie genome, 30 of which were located on the same scaffold as IGH constant. The highest number of V gene segments were identified in the koala genome, with 147 characterised across 27 scaffolds, 91 of which were located on the same scaffold as IGH constant. The high number of V gene segments identified in the koala is likely a factor of assembly quality rather than gene expansion. Additional work is required to improve contiguity within this region of the genome in the remaining four species in order to facilitate characterisation of additional V segments.

Light chains of the immunoglobulin molecule also contain a variable domain. However, unlike heavy chains, this domain is comprised of only V and J gene segments in both kappa and lambda light chains. V gene segments were overall more numerous for light chains compared to heavy chains. Only 15 lambda light chain V gene segments were identified in the koala and wombat, with the most identified in the woylie with 80 V segments located across three scaffolds. Numerous kappa light chain V gene segments were identified in all five species, ranging from 61 V genes on 34 scaffolds in the wombat to 127 genes on 6 scaffolds in the koala (Supplementary Table 7).

Supplementary Table 7. Number of immunoglobulin heavy and light chain constant (C) and variable (V) gene sequences identified in koala, woylie, wombat, antechinus and numbat. The number of scaffolds on which the genes were encoded is indicated in parantheses. Raised number indicated the number of V gene segments located on the same scaffold as constant gene. * indicates partial sequences were identified.

|  | **Koala** | **Woylie** | **Wombat** | **Antechinus** | **Numbat** |
| --- | --- | --- | --- | --- | --- |
| **IGH C genes** | 5 (1) | 8 (1) | 4 (1) | 4 (1) | 3 (4) |
| **IGH V gene segments** | 147^91^ (27) | 35^34^ (2) | 22^3^ (19) | 5^1^ (4) | 6^0^ (5) |
| **IGL C genes** | 9 | 10 | 4* | 1 | 1 |
| **IGL V gene segments** | 15 (1) | 80 (3) | 15 (15) | 21 (18) | 36 (27) |
| **IGK C genes** | 1 | 2 | 4* | 2 | 1 |
| **IGK V gene segments** | 127 (6) | 111 (6) | 61 (30) | 119 (82) | 79 (48) |


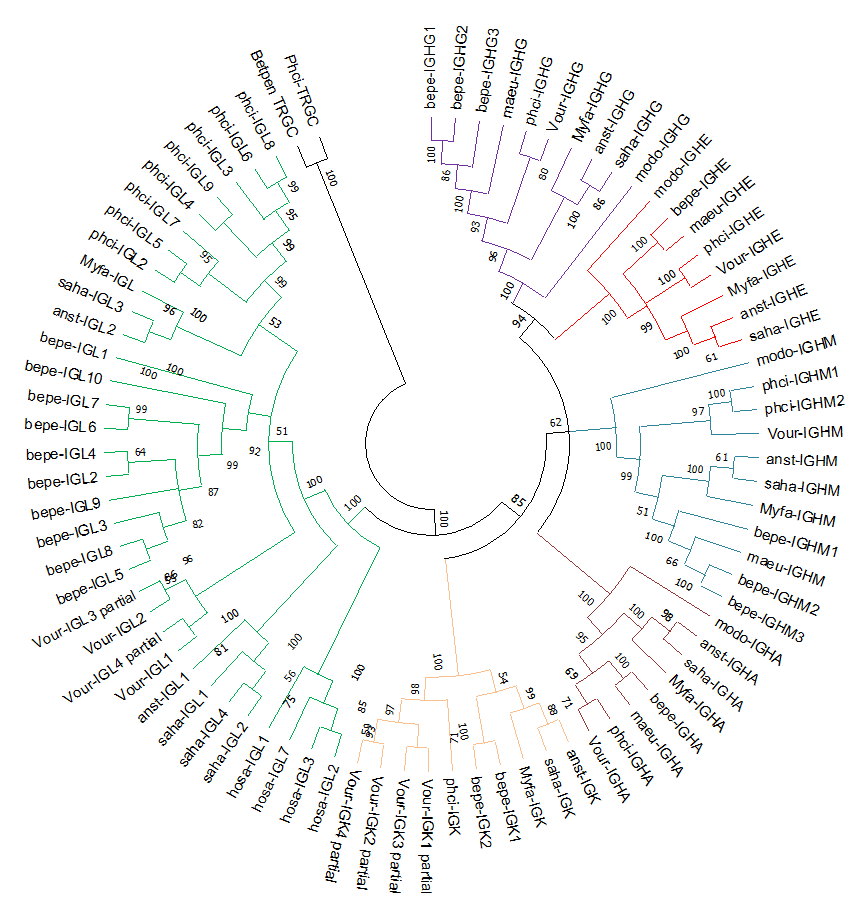


Supplementary Figure 20. Phylogenetic relationships amongst marsupial as well as immunoglobulin heavy and light chain constant regions. The neighbour joining phylogenetic tree was constructed in MEGAX using the p-distance method with pairwise deletion and 1000 bootstrap replicates. Bootstrapping values below 50% are not shown. Tree is rooted with TRGC sequences from koala and woylie.

# Major Histocompatibility Complex

The Major Histocompatibility Complex (MHC) consists of two main classes (class I and class II) of cell surface molecules. The two classes can be further characterised into classical and non-classical genes. Class I genes are responsible for presentation of antigens to T lymphocytes [82]. Classical class I genes are typically highly polymorphic and expressed throughout tissue types, whereas non classical class I genes are typically less polymorphic and more variably expressed across tissues [24]. Class I genes were identified in all five study species with the number of genes ranging from 3 (numbat) to 19 (koala). The genomic region encoding MHC class I genes was highly fragmented in all species, as genes were located across numerous scaffolds. As the classification of class I genes as classical or non-classical is dependent on expression profiles and polymorphism, it was not possible to assign genes further.

MHC class II genes encode cell surface molecules and are typically expressed on the surface of immune cells [83]. MHC II genes are responsible for binding peptides from pathogens (particularly bacteria and parasites) and presenting them to CD4+ helper T cells [84]. MHC class II molecules consist of an α and β chain, encoded by 4 genes in marsupials (DA, DB, DC and DM) which are not orthologous to eutherian MHC class II [29, 30]. Gene copy number and polymorphism within MHC class II genes differs between species. The α chain of DC and DA is typically represented by a single gene in most marsupials, while DB α, DA and DB β have duplicated in some species [24, 85, 86]. At least one α and β chain coding gene was identified in koala, woylie, wombat, antechinus and numbat (Supplementary Table 8, Supplementary Figure 21). However, only in the koala were α and β chains identified for all four class II genes. DA, DB and DM α and β chains were identified in the woylie genome, with 12 DA chains identified. In the wombat, all genes except DB β chain were identified. Only DA and DM α and β chains were characterised in the antechinus genome, with 5 complete DA β chains and 6 partial chains. The numbat MHC class II region was the most incomplete and fragmented of all five species studied, with only DA α and β chains and a single DB β chain identified (Supplementary Table 8, Supplementary Figure 21).

Supplementary Table 8. Number of MHC class II genes identified in koala, woylie, wombat, antechinus and numbat genomes.

|  | **DA** | | **DB** | | **DC** | | **DM** | | **Total** |
| --- | --- | --- | --- | --- | --- | --- | --- | --- | --- |
|  | **α** | **β** | **α** | **β** | **α** | **β** | **α** | **β** |  |
| **Koala** | 1 | 5 | 3 | 3 | 1 | 1 | 1 | 1 | 16 |
| **Woylie** | 1 | 12 | 2 | 5 | 0 | 0 | 2 | 1 | 23 |
| **Wombat** | 1 | 1 | 1 | 0 | 1 | 1 | 1 | 1 | 7 |
| **Antechinus** | 1 | 11 | 0 | 0 | 0 | 0 | 1 | 1 | 14 |
| **Numbat** | 1 | 4 | 0 | 1 | 0 | 0 | 0 | 0 | 6 |


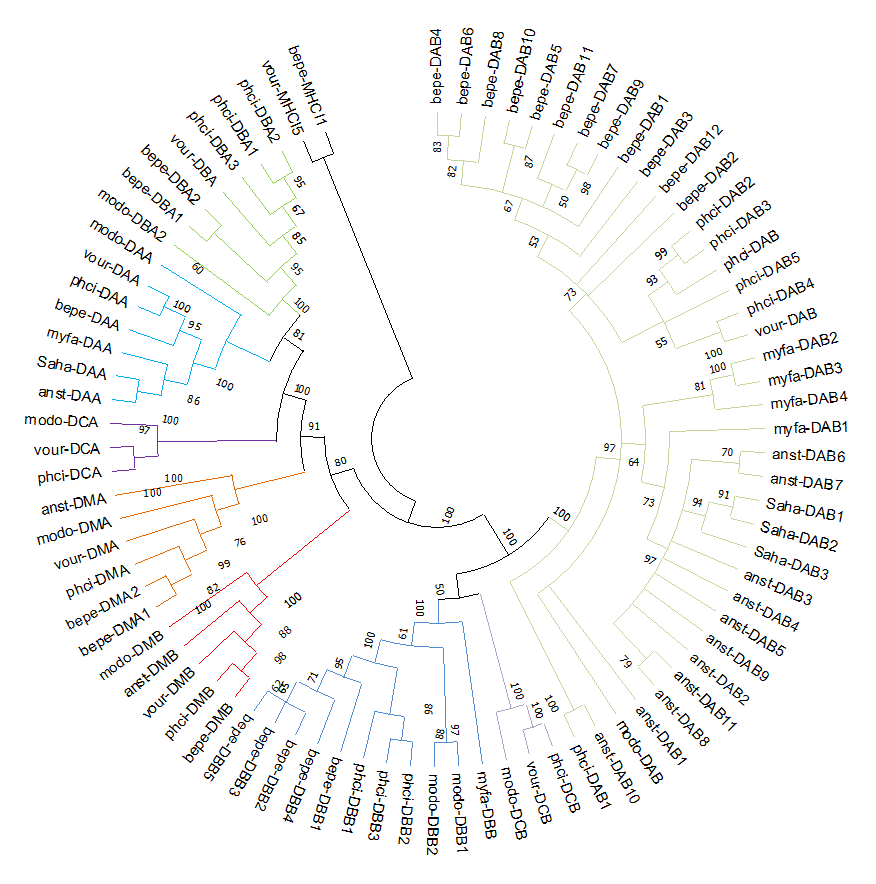


Supplementary Figure 21. Phylogenetic relationships amongst marsupial MHC class II genes. Branch colours indicate different MHC class II α and β genes. The neighbour joining phylogenetic tree was constructed in MEGAX using the p-distance method with pairwise deletion and 1000 bootstrap replicates. Bootstrapping values below 50% are not shown. Tree is rooted with MHC class I sequences from woylie and wombat.

# References

1. Morris K, Prentis PJ, O'Meally D, Pavasovic A, Brown AT, Timms P, Belov K, Polkinghorne A: **The koala immunological toolkit: sequence identification and comparison of key markers of the koala (*Phascolarctos cinereus*) immune response.** *Australian Journal of Zoology* 2014, **62:**195-199.

2. Daly KA, Digby M, Lefevre C, Mailer S, Thomson P, Nicholas KR, Williamson P: **Analysis of the expression of immunoglobulins throughout lactation suggests two periods of immune transfer in the tammar wallaby (*Macropus eugenii*).** *Veterinary Immunology and Immunopathology* 2007, **120:**187-200.

3. Morris KM, Weaver HJ, O'Meally D, Desclozeaux M, Gillett A, Polkinghorne A: **Transcriptome sequencing of the long-nosed bandicoot (*Perameles nasuta*) reveals conservation and innovation of immune genes in the marsupial order Peramelemorphia.** *Immunogenetics* 2017, **69:**1-10.

4. Aveskogh M, Hellman L: **Evidence for an early appearance of modern post-switch isotypes in mammalian evolution; cloning of IgE, IgG and IgA from the marsupial *Monodelphis domestica*.** *European Journal of Immunology* 1998, **28:**2738-2750.

5. Wang X, Olp JJ, Miller RD: **On the genomics of immunoglobulins in the gray, short-tailed opossum *Monodelphis domestica*.** *Immunogenetics* 2009, **61:**581-596.

6. Belov K, Harrison GA, Miller RD, Cooper DW: **Isolation and sequence of a cDNA coding for the heavy chain constant region of IgG from the Australian brushtail possum, *Trichosurus vulpecula*.** *Molecular Immunology* 1999, **36:**535-541.

7. Johnson RN, O’Meally D, Chen Z, Etherington GJ, Ho SYW, Nash WJ, Grueber CE, Cheng Y, Whittington CM, Dennison S, et al: **Adaptation and conservation insights from the koala genome.** *Nature Genetics* 2018, **50:**1102-1111.

8. Miller RD, Grabe H, Rosenberg GH: **VH repertoire of a marsupial (*Monodelphis domestica*).** *The Journal of Immunology* 1998, **160:**259-265.

9. Belov K, Harrison GA, Rosenberg GH, Miller RD, Cooper DW: **Isolation and comparison of the IgM heavy chain constant regions from Australian (*Trichosurus vulpecula*) and American *(Monodelphis domestica*) marsupials.** *Developmenal and Comparative Immunology* 1999, **23:**649-656.

10. Morris B, Cheng Y, Warren W, Papenfuss AT, Belov K: **Identification and analysis of divergent immune gene families within the Tasmanian devil genome.** *BMC Genomics* 2015, **16**.

11. Belov K, Harrison GA, Miller RD, Cooper DW: **Molecular cloning of four lambda light chain cDNAs from the Australian brushtail possum *Trichosurus vulpecula*.** *European Journal of Immunogenetics* 2002, **29:**95-99.

12. Miller RD, Bergemann ER, Rosenberg GH: **Marsupial light chains: *IGK* with four *V* families in the opossum *Monodelphis domestica*.** *Immunogenetics* 1999, **50:**329-335.

13. Belov K, Harrison GM, RD, Cooper D: **Characterization of the kappa light chain of the brushtail possum (*Trichosurus vulpeca*).** *Veterinary Immunology and Immunopathology* 2001, **78:**317-324.

14. Baker ML, Rosenberg GH, Zuccolotto P, Harrison GA, Deane EM, Miller RD: **Further characterization of T cell receptor chains or marsupials.** *Developmenal and Comparative Immunology* 2001, **25:**495-507.

15. Zuccolotto P, Harrison GA, Deane EM: **Cloning of marsupial T cell receptor alpha and beta constant region cDNAs.** *Immunology and Cell Biology* 2000, **78:**103-109.

16. Baker ML, Indiviglio SAM, Rosenberg GH, Lindblad-Toh K, Miller RD, Papenfuss AT: **Analysis of a set of Australian northern brown bandicoot expressed sequence tags with comparison to the genome sequence of the South American gray short tailed opossum.** *BMC Genomics* 2007, **8:**1-10.

17. Parra ZE, Baker ML, Hathaway J, Lopez AM, Trujillo J, Sharp A, Miller RD: **Comparative genomic analysis and evolution of the T cell receptor loci in the opossum *Monodelphis domestica*.** *BMC Genomics* 2008, **9:**1-19.

18. Harrison GA, Taylor CL, Miller RD, Deane EM: **Primary structure and variation of the T-cell receptor delta-chain from a marsupial, *Macropus eugenii*.** *Immunology Letters* 2003, **88:**117-125.

19. Peel E, Frankenberg SR, Pask A, Belov K: **Annotation of immune genes in the extinct thylacine (*Thylacinus cynocephalus*).** *Immunogenetics* 2021.

20. Parra ZE, Baker ML, Schwarz RS, Deakin JE, Lindblad-Toh K, Miller RD: **A unique T cell receptor discovered in marsupials.** *PNAS* 2007, **104:**9776-9781.

21. Cui J, Cheng Y, Belov K: **Diversity in the Toll-like receptor genes of the Tasmanian devil (Sarcophilus harrisii).** *Immunogenetics* 2015, **67:**195-201.

22. Belov K, Sanderson CE, Deakin JE, Wong ESW, Assange D, McColl KA, Gout A, Bono B, Barrow AD, Speed TP, et al: **Characterization of the opossum immune genome provides insight into the evolution of the mammalian immune system.** *Genome Research* 2007, **17:**982-991.

23. Cui J, Frankham GJ, Johnson RN, Polkinghorne A, Timms P, O'Meally D, Cheng Y, Belov K: **SNP marker discovery in koala TLR genes.** *PLOS ONE* 2015, **10:**1-15.

24. Cheng Y, Stuart A, Morris K, Taylor R, Siddle HV, Deakin JE, Jones M, Amemiya CT, Belov K: **Antigen-presenting genes and genomic copy number variations in the Tasmanian devil MHC.** *BMC Genomics* 2012, **13:**87.

25. Cheng Y, Belov K: **Characterisation of non-classical MHC class I genes in the Tasmanian devil (Sarcophilus harrisii).** *Immunogenetics* 2014, **66:**727-735.

26. Papenfuss AT, Feng Z, Krasnec K, Deakin JE, Baker ML, Miller RD: **Marsupials and monotremes possess a novel family of MHC class I genes that is lost from the eutherian lineage.** *BMC Genomics* 2015, **16:**535.

27. Belov K, Deakin JE, Papenfuss AT, Baker ML, Melman SD, Siddle HV, Gouin N, Goode DL, Sargent TJ, Robinson MD, et al: **Reconstructing an ancestral mammalian immune supercomplex from a marsupial major histocompatability complex.** *PLOS Biology* 2006, **4:**317-328.

28. Siddle HV, Deakin JE, Coggill P, Hart E, Chen Y, Wong ESW, Harrow J, Beck S, Belov K: **MHC-linked and un-linked class I genes in the wallaby.** *BMC Genomics* 2009, **10:**310.

29. Belov K, Lam MKP, Colgan DJ: **Marsupial MHC class II DAB and DBB genes are not orthologous to the eutherian beta gene families.** *The Journal of Heredity* 2004, **95:**338-345.

30. Siddle HV, Deakin JE, Coggill P, Whilming LG, Harrow J, Kaufman J, Beck S, Belov K: **The tammar wallaby major histocompatability complex shows evidence of past genomic instability.** *BMC Genomics* 2011, **12:**421.

31. Wong ESW, Papenfuss AT, Belov K: **Genomic identification of chemokines and cytokines in opossum.** *Journal of Interferon and Cytokine Research* 2011, **31:**317-330.

32. Wong ESW, Young LJ, Papenfuss AT, Belov K: ***In silico* identification of opossum cytokine genes suggest the complexity of the marsupial immune system rivals that of eutherian mammals.** *Immunome Research* 2006, **2**.

33. Mathew M, Waugh C, Beagley KW, Timms P, Polkinghorne A: **Interleukin 17A is an immune marker for chlamydial disease severity and pathogenesis in the koala (Phascolarctos cinereus).** *Developmental & Comparative Immunology* 2014, **46:**423-429.

34. Wedlock DN, Goh LP, Parlane NA, Buddle BM: **Molecular cloning and physiological effects of brushtail possum interleukin-1beta.** *Veterinary Immunology and Immunopathology* 1999, **67:**359-372.

35. Young LJ, Harrison GA: **Molecular characterization of Interleukin-1Beta in the Tammar wallaby (*Macropus eugenii*).** *Journal of Veterinary Medical Science* 2010, **72:**1521-1526.

36. Young LJ, Gurr J, Morris K, Flenady S, Belov K: **Molecular characterisation of interleukin-2 in two Australian marsupials (thre tammar wallaby, *Notamacropus eugenii*, and the Tasmanian devil, *Sarcophilus harrisii*) facilitates the development of marsupial-specific immunological reagents.** *Australian Mammalogy* 2019, **41**.

37. Young LJ, Cross ML, Duckworth JA, Flenady S, Belov K: **Molecular identification of interleukin-2 in the lymphoid tissues of the common brushtail possum, *Trichosurus vulpeca*.** *Developmenal and Comparative Immunology* 2012, **36:**236-240.

38. Young L: **Expressed sequence identification and characterisation of the cDNA for Interleukin-4 from the mitogen-stimulated lymphoid tissue of a marsupial, *Macropus eugenii*.** *Veterinary Immunology and Immunopathology* 2011, **140:**335-340.

39. Hawken RJ, Maccarone P, Toder R, Marshall Graves JA, Maddox JF: **Isolation and charactierzation of marsupial *IL5* genes.** *Immunogenetics* 1999, **49:**942-948.

40. Maher IE, Griffith JE, Lau Q, Reeves T, Higgins DP: **Expression profiles of the immune genes CD4, CD8 beta, IFN gamma, IL-4, IL-6 and IL-10 in mitogen-stimulated koala lymphocytes (*Phascolarctos cinereus*) by qRT-PCR.** *PeerJ* 2014, **2**.

41. Borthwick CR, Young LJ, McAllan BM, Old JM: **Identification of the mRNA encoding interleukin-6 and its receptor, interleukin-6 receptor α, in five marsupial species.** *Developmental & Comparative Immunology* 2016, **65:**211-217.

42. Alsemgeest J, Old JM, Young LJ: **Molecular characterization and expression of Interleukin-6 and Interleukin-6 delta 2 in the Tammar wallaby (*Macropus eugenii*).** *Veterinary Immunology and Immunopathology* 2013, **155:**139-145.

43. Wedlock DN, Aldwell FE, Buddle BM: **Nucleotide sequence of a marsupial interleukin-10 cDNA from the Australian brushtail possum (*Trichosurus vulpecula*).** *The Journal of Sequencing and Mapping* 1998, **9:**239-244.

44. Alsemgeest J, Old JM, Young LJ: **Further characterisation of cytokines in macropod marsupials: IL-10 and IL-10Δ3.** *Cytokine* 2016, **88:**37-44.

45. Suthers AN, Old JM, Young LJ: **The common gamma chain cytokine interleukin-21 is expressed by activated lymphocytes from two macropod marsupials, *Macropus eugenii* and *Onychogalea fraenata*.** *International Journal of Immunogenetics* 2016, **43:**209-217.

46. Alsemgeest J, Old JM, Young LJ: **The macropod type 2 interferon gene shares important regulatory and functionally relevant regions with eutherian IFN-γ.** *Molecular Immunology* 2015, **63:**297-304.

47. Harrison GA, Deane EM: **cDNA cloning of lymphotoxin alpha from a marsupial, *Macropus eugenii*.** *DNA sequence* 2000, **10:**399-403.

48. Harrison GA, Deane EM: **cDNA sequence of the lymphotoxin beta chain from a marsupial, *Macropus eugenii* (Tammar wallaby).** *Journal of Interferon and Cytokine Research* 1999, **19:**1099-1102.

49. Wedlock DN, Aldwell FE, Buddle BM: **Molecular cloning and characterization of tumour necrosis factor alpha (TNF-alpha) from the Australian common brushtail possum, *Trichosurus vulpecula*.** *Immunology and Cell Biology* 1996, **74:**151-158.

50. Morris KM, Matthew M, Waugh C, Ujvari B, Timms P, Polkinghorne A, Belov K: **Identification, characterisation and expression analysis of natural killer receptor genes in *Chlamydia pecorum* infected koalas (*Phascolarctos cinereus*).** *BMC Genomics* 2015, **16**.

51. van der Kraan LE, Wong ESW, Lo N, Ujvari B, Belov K: **Identification of natural killer cell receptor genes in the genome of the marsupial Tasmanian devil (*Sarcophilus harrisii*).** *Immunogenetics* 2013, **65:**25-35.

52. Roach JC, Glusman G, Rowen L, Kaur A, Purcell MK, Smith KD, Hood LE, Aderem A: **The evolution of vertebrate Toll-like receptors.** *PNAS* 2005, **102:**9577-9582.

53. Jin MS, Kim SE, Heo JY, Lee ME, Kim HM, Paik S-G, Lee H, Lee J-O: **Crystal Structure of the TLR1-TLR2 Heterodimer Induced by Binding of a Tri-Acylated Lipopeptide.** *Cell* 2007, **130:**1071-1082.

54. Hajjar AM, Ernst RK, Tsai JH, Wilson CB, Miller SI: **Human Toll-like receptor 4 recognises host-specific LPS modifications.** *Nature Immunology* 2002, **3:**354-359.

55. Meng J, Drolet JR, Monks BG, Golenbock DT: **MD-2 residues tyrosine 42, arginine 69, aspartic acid 122, and leucine 125 provide species specificity for lipid IVA.** *The Journal of Biological Chemistry* 2010, **285:**27935-27943.

56. Mizel SB, West AP, Hantgan RR: **Identification of a sequence in human Toll-like receptor 5 required for the binding of gram-negative flagellin.** *The Journal of Biological Chemistry* 2003, **278:**23624-23629.

57. Wei T, Gong J, Jamitzky F, Heckl WM, Stark RW, Rossle SC: **Homology modeling of human Toll-like receptors TLR7, 8 and 9 ligand-binding domains.** *Protein Science* 2009, **18:**1664-1691.

58. Govindaraj RG, Manavalan B, Lee G, Choi S: **Molecular modeling-based evaluation of hTLR10 and identification of potential ligans in Toll-like receptor signalling.** *PLoS one* 2010, **5:**e12713.

59. Kelley J, Walfer L, Trowsdale J: **Comparative genomics of natural killer cell receptor gene clusters.** *PLOS Genetics* 2005, **1:**129-139.

60. Wong ESW, Sanderson CE, Deakin JE, Whittington CM, Papenfuss AT, Belov K: **Identification of natural killer cell receptor clusters in the platypus genome reveals an expansion of C-type lectin genes.** *Immunogenetics* 2009, **61:**565-579.

61. Hobbs M, Pavasovic A, King AG, Prentis PJ, Eldridge MDB, Chen Z, Colgan DJ, Polkinghorne A, Wilkins MR, Flanagan C, et al: **A transcriptome resource for the koala (*Phascolarctos cinereus*): insights into koala retrovirus transcription and sequence diversity.** *BMC Genomics* 2014, **15:**786.

62. Wack A, Terczynska-Dyla E, Hartmann R: **Guarding the frontiers: the biology of type III interferons.** *Nature Immunology* 2015, **16:**802-809.

63. Maher IE, Higgins DP: **Altered immune cytokine expression associated with KoRV B infection and season in captive koalas.** *PLoS One* 2016, **11**.

64. Mathew M, Beagley KW, Timms P, Polkinghorne A: **Preliminary characterisation of tumor necrosis factor alpha and interleukin-10 responses to *Chlamydia pecorum* infection in the koala (*Phascolarctos cinereus*).** *PLOS one* 2013, **8:**1-9.

65. Baker ML, Osferman AK, Brumburgh S: **Divergent T-cell reeptor delta chains from marsupials.** *Immunogenetics* 2005, **57:**665-673.

66. Zhou Y, Shearwin-Whyatt L, Li J, Song Z, Hayakawa T, Stevens D, Fenelon JC, Peel E, Cheng Y, Pajpach F, et al: **Platypus and echidna genomes reveal mammalian biology and evolution.** *Nature* 2021.

67. Satyanarayana K, Hata S, Devlin P, Roncarolo MG, De Vries JE, Spits H, Strominger JL, Krangel MS: **Genomic organization of the human T-cell-antigen-receptor alpha/delta locus.** *Proceedings of the National Academy of Sciences* 1988, **85:**8166-8170.

68. Kubota T, Wang J-y, Göbel TWF, Hockett RD, Cooper MD, Chen C-lH: **Characterization of an Avian (&lt;em&gt;Gallus gallus domesticus&lt;/em&gt;) TCR αδ Gene Locus.** *The Journal of Immunology* 1999, **163:**3858.

69. Glusman G, Rowen L, Lee I, Boysen C, Roach JC, Smit AFA, Wang K, Koop BF, Hood L: **Comparative Genomics of the Human and Mouse T Cell Receptor Loci.** *Immunity* 2001, **15:**337-349.

70. Call ME, Wucherpfennig KW: **The T cell receptor: Critical Role of the Membrane Environment in Receptor Assembly and Function.** *Annual Review of Immunology* 2005, **23:**101-125.

71. Rowen L, Koop BF, Hood LE: **The complete 685-kilobase sequence of the human beta T cell receptor locus.** *Science* 1996, **272:**1755-1762.

72. Saito H, Kranz DM, Takagaki Y, Hayday AC, Eisen HN, Tonegawa S: **Complete primary structure of a heterodimeric T-cell receptor deduced from cDNA sequences.** *Nature* 1984, **309:**757-762.

73. Alcover A, Mariuzza RA, Ermonval M, Acuto O: **Lysine 271 in the transmembrane domain of the T-cell antigen receptor beta chain is necessary for its assembly with the CD3 complex but not for alpha/beta dimerization.** *Journal of Biological Chemistry* 1990, **265:**4131-4135.

74. Campbell KS, Backstrom T, Tiefenthaler G, Palmer E: **CART: a conserved antigen receptor transmembrane motif.** *Seminars in Immunology* 1994, **6:**393-410.

75. Su c, Jakobsen I, Gu X, Nei M: **Diversity and evolution of T-cell receptor variable region genes in mammals and birds.** *Immunogenetics* 1999, **50:**301-308.

76. Vernooij BTM, Lenstra JA, Wang K, Hood L: **Organization of the Murine T-Cell Receptor γ Locus.** *Genomics* 1993, **17:**566-574.

77. Parra ZE, Arnold T, Nowak MA, Hellman L, Miller RD: **TCR gamma chain diversity in the splen of the duckbill platypus (*Ornithorhynchus anatinus*).** *Developmenal and Comparative Immunology* 2006, **30:**699-671.

78. Miller RD: **Those other mammals: The immunoglobulins and T cell receptors of marsupials and monotremes.** *Seminars in Immunology* 2010, **22:**3-9.

79. Parra ZE, Baker ML, Lopez AM, Trujillo J, Volpe JM, Miller RD: **TCRmu recombination and transcription relative to the conventional TCR during postnatal development in opossums.** *The Journal of Immunology* 2009, **182:**154-163.

80. Miller RD, Belov K: **Immunoglobulin genetics of marsupials.** *Developmenal and Comparative Immunology* 2000, **24:**485-490.

81. Hesslein DGT, Schatz DG: **Factors and forces controlling V (D) J recombination.** *Advances in Immunology* 2001, **78:**169-232.

82. Cresswell P, Ackerman AL, Giodini A, Peaper DR, Wearsch PA: **Mechanisms of MHC class-I restricted antigen processing and cross-presentation.** *Immunological Reviews* 2005, **207:**145-157.

83. Lam MKP, Belov K, Harrison GA, Cooper DW: **Cloning of the MHC class II DRB cDNA from the brushtail possum (*Trichosurus vulpecula*).** *Immunology Letters* 2001, **76:**31-36.

84. Frank SA: *Immunology and evolution of infectious disease.* Princeton: Princeton University Press; 2002.

85. Cheng Y, Siddle HV, Beck S, Eldridge MDB, Belov K: **High levels of genetic variation at MHC class II DBB loci in the tammar wallaby (*Macropus eugenii*).** *Immunogenetics* 2009, **61:**111-118.

86. Lau Q, Jobbins SE, Belov K, Higgins DP: **Characterisation of four major histocompatibility complex class II genes of the koala (*Phascolarctos cinereus*).** *Immunogenetics* 2013, **65:**37-46.
