# Supplementary material for: Best genome sequencing strategies for annotation of complex immune gene families in wildlife
Source: Gigascience. 2022 Oct 30;11:giac100. doi: 10.1093/gigascience/giac100 (PMC9618407; doi:10.1093/gigascience/giac100)
Supplement: giac100_Response_to_Reviewer_Comments_Original_Submission [file giac100_response_to_reviewer_comments_original_submission.pdf]

Author's Response To Reviewer Comments

Close

Also attached as a file to maintain formatting for ease of reading.

Response to reviewers
Reviewer #1:
REVIEWER COMMENT: Knowledge about immune genes is critical for species conservation programs. However, immune genes occur in large gene clusters that are difficult to assemble and annotate. This important and timely study uses a number of marsupial genomes and the platypus to assess which sequencing technologies enable complete reconstructions of immune gene clusters and which methods enable annotations of these immune genes.

I have the following comments.

Since Fgenesh++ and Maker produce automatic annotations, I wonder why not all 6 genomes were annotated with these two methods? This would allow a comparison between Fgenesh++ against Maker. Maybe it is possible to annotate at least a few genomes with both methods.

RESPONSE: All six genomes were not annotated using Fgenesh++ and Maker as the authors wanted to utilise existing annotations available for 5 of the 6 genomes in our study (all except koala). The authors agree that re-annotating all genomes with both Fgenesh++ and Maker would enable a direct comparison between the two methods. However, determining the best automated annotation software for immune gene annotation was not the focus of this study, but rather the impact of assembly quality on immune gene annotation. A secondary aim of the paper was to investigate whether automated annotation software was able to accurately identify immune genes, compared to our manual annotations. While it is widely known within the field of wildlife immunogenetics that automated genome annotations fail to correctly characterise immune genes, to date there are no publications which quantitatively assess this observation.
The computation required to annotate all six genomes using both Fgenesh++ and MAKER was not feasible within the given three-month timeframe provided for changes to the manuscript. As such, the koala, wombat and 2021 platypus genomes have been annotated with Fgenesh++ which will enable investigation of how this popular annotation software performs for immune gene annotation within all genome assemblies of varying quality included in this study (woylie, antechinus and numbat were already annotated with Fgenesh++). The methods, results and figure 1 have been modified to reflect this. See lines 213-218, 261-273 of the results and below. Additional supplementary figures have been generated in response to the reviewer’s comment. See supplementary figure 3, 4 and 5 in Additional file 2.
Table 1 has been modified to also include all genome annotations used in this study. This includes existing published annotations by NCBI, MAKER and Fgenesh++, as well as Fgenesh++ annotations conducted as part of this study.

Lines 213-218
“We assessed how well our manual immune gene annotation aligned with automated annotations by Fgenesh++ (2018 platypus, woylie, koala, antechinus, numbat and wombat), MAKER (wombat) and the NCBI pipeline (2021 platypus). Inclusion of the 2021 platypus NCBI and wombat MAKER annotations ensures that any differences in automated and manual immune gene annotation were not due to deficiencies within the Fgenesh++ annotation pipeline, as the woylie, antechinus and numbat genomes were all annotated with Fgenesh++ using the same parameters.”
Lines 263-275
“This pattern of poor immune gene annotation was not an artefact of inherent differences between automated annotation pipelines amongst the six genomes (NCBI, MAKER and Fgenesh++) nor genome quality, as similar patterns were observed for Fgenesh++ annotations of the 2021 platypus and wombat genome generated as part of this study (Supplementary Figure 3, Supplementary Figure 4). Generally, the Fgenesh++ annotation resulted in fewer correctly annotated immune genes (≥90% overlap) compared to NCBI (2021 platypus) or MAKER (wombat) (Supplementary Figure 3). Although, the

proportion of missing immune genes (0% overlap) was higher in the NCBI (2021 platypus) and MAKER (wombat) annotation than the Fgenesh++ annotation of both species genomes. As with NCBI and MAKER, Fgenesh++ poorly annotated TCR and IG families at the gene-level (Supplementary Figure 4) in the high-quality platypus and low-quality wombat. Correct annotations were somewhat recovered at the exon-level in both genomes (Supplementary Figure 5), although, the number of missing TCR and IG exons in the Fgenesh++ annotation was almost half that of NCBI and MAKER in platypus and wombat respectively."

REVIEWER COMMENT: Direct assessments of assembly quality should ideally be done on different assemblies of the same species to rule out real differences between species. Would it be possible to include previous koala or platypus genome that was much more fragmented?

RESPONSE: The authors agree that multiple versions of the same genome assembly would enable direct assessment of assembly quality on immune gene annotation. As such, the authors have annotated the latest 2021 version of the platypus genome assembly published by Zhou et al 2021 (NCBI ID GCA_004115215.4) and the previous 2018 version (GCA_002966995.1) with Fgenesh++. Platypus was selected as the species for this comparison over koala (the only other species in our study with multiple genome assemblies available) as the improvement in assembly metrics between the 2021 and 2018 platypus genome assemblies is more significant than the 2018 and 2020 koala genome assemblies. This is due to the addition of numerous data types to the 2021 platypus assembly since the 2018 version. Genome assembly metrics for the 2018 platypus genome have been added to Table 1. The results section "Relationship between genome quality and manual immune gene annotation" has been modified to include a comparison between the 2018 and 2021 platypus assemblies. Specifically, see lines 283-290 and below. Figure 2 has also been updated to include the 2018 platypus genome assembly.
Fgenesh++ was selected for automated annotation of the two platypus assemblies over other methods such as MAKER as this would enable direct comparison of Fgenesh++ performance across all genomes in this study.
Lines 285-292
"To rule out species-specific differences in our direct assessment of assembly quality on immune gene annotation, we annotated a previous version of the platypus genome from 2018 (GCA_002966995.1) with Fgenesh++ to enable comparison with our Fgenesh++ annotation of the 2021 platypus genome (GCA_004115215.4) also generated as part of this study. Compared to the 2021 assembly, the 2018 platypus assembly was more fragmented given the 6-fold increase in the number of contigs, 14-fold increase in the number of scaffolds, and associated 2-fold decrease in contig N50 and 4-fold decrease in scaffold N50 between the two assemblies. Despite these metrics, the 2018 platypus assembly is still highly contiguous as it was generated using long-read data."

REVIEWER COMMENT: Figure 1 shows a useful of all immune genes. However, some genes like TLRs are actually easy to annotate as they are have a standard gene structure. Therefore, it would be informative to provide in this figure a breakdown of how well the different immune gene families are annotates, as the authors nicely did in table 2. This would inform on which immune genes are particularly difficult to annotate.

RESPONSE: A breakdown of Figure 1 by immune family is now presented in Supplementary Figure 1 of Additional File 2. A breakdown of annotation at the exon-level by immune family has been added as Supplementary Figure 2. See lines 235-255 and below. Similar breakdown of this analysis by immune family have also been added for Fgenesh++ versus MAKER (wombat) or NCBI (2021 platypus) annotations of the platypus and wombat genome assemblies at the gene level for all seven families (Supplementary Figure 4), in addition to exon-level for TCR and IG families (Supplementary figure 5).
Lines 235-255
"A breakdown of this analysis by immune family revealed that marsupial- and monotreme-specific immune genes which are not orthologous to those in eutherians were generally poorly annotated, regardless of automated pipeline or genome quality (Supplementary Figure 1). This was particularly the case for TCR and IG gene families, with up to 88% of genes in these families incorrectly annotated by automated pipelines (≤10% overlap) amongst the six species (Table 2). This is likely due to highly duplicated variable gene segments that do not encode conventional exon-intron splice sites which may hinder annotation with automated pipelines. Poor gene annotations of TCR and IG families was somewhat recovered at the exon level, as some TCR and IG variable gene segments were annotated as exons by automated pipelines. Correct annotation (≥90% overlap) of the TCR family increased from 0-2% at the gene level to 2-15% at the exon level amongst the six genomes (Supplementary Figure 2). This improvement was even greater for the IG family, with an increase from 0-2% correct annotation at the gene level to 15-43% at the exon level amongst the six genomes (Supplementary Figure 2). Despite

this, up to 67% of TCR and IG variable segments were still not annotated at the exon level (0% overlap) amongst the six genomes, highlighting the difficulty in automated annotation of these regions. Similarly, marsupial-specific gene expansions within the leukocyte receptor complex (LRC) and monotreme-specific gene expansions within the natural killer complex (NKC) family of NK receptors were also poorly annotated by automated pipelines (Supplementary Figure 1). As with TCR and IG families, correct annotation increased from the gene- (0-28% marsupial LRC, 31% platypus NKC) to exon-level (6-65% marsupial LRC, 79% platypus NKC) (Table 2, Supplementary Figure 2), likely due to the presence of variable numbers of duplicated immunoglobulin superfamily (IGSF) domains and C-type lectin (CLEC) domains within each LRC and NKC gene respectively."

REVIEWER COMMENT: Figure 3B is not colorblind friendly.

RESPONSE: Colours in Figure 3B have been amended according to the colourblind friendly palette outlined in Wong, B. Points of view: Color blindness. Nat Methods 8, 441 (2011). https://doi.org/10.1038/nmeth.1618

REVIEWER COMMENT: Line 275: The discussion makes it clear that this is a scaffolding error and not a real inversion. This should be clarified here as well.

RESPONSE: This has been clarified in the text, see lines 345-347 and below.
"This organisation is unusual amongst mammalian TCR and is likely a result of the HiC scaffolding error and not a true inversion."

REVIEWER COMMENT: I fully agree with the value of the manual annotations. Therefore, it would be helpful to provide the manual annotations also as a gff3 or gtf file that provide the full exon structure. Additional file 2 only lists the start and end coordinates of genes with multiple exons. The assembly accession should also be listed.

RESPONSE: Additional file 1 (previously Additional file 2) has been amended to include both the gene and exon coordinates for all immune genes across the 7 genome assemblies.

REVIEWER COMMENT: As a suggestion: A haplotype-resolved assembly of a marsupial is likely not yet available, but such an assembly would provide an opportunity to further investigate the influence of assembly quality and haplotype variation in immune genes.

RESPONSE: The authors agree with the reviewer's comment. However, a haplotype-resolved assembly for marsupials will be challenging to generate given current recommendations include the use of trios to completely resolve paternal and maternal haplotypes. Samples from trios are incredibly difficult to obtain for wildlife such as marsupials given the opportunistic nature of most sample collection. This would be especially difficult for marsupials which are threatened or endangered, or are not currently housed in captivity.

Reviewer #2:
REVIEWER COMMENT: In this work, Peel and collaborators asses the accuracy of immune gene annotation in marsupial species by comparing the outcome of manually and automated annotation approaches. This allowed them to conclude that sequence data type and assembly quality determine the accuracy of gene annotation. I find the study interesting, although I have some general comments. I find that both the introduction and discussion sections would benefit from some re-structuring. Both sections are a bit long, with some repetitions. Also, the discussion section contains material from results. I would also appreciate more detailed figure legends.

RESPONSE: The authors thank reviewer 2 for their comments. In light of no specific changes provided by reviewer 2, and changes already made to both the discussion and introduction for reviewer 1 and 3, we took no further action.

Reviewer #3:
REVIEWER COMMENT: In this manuscript, Peel et al examine the impact of assembly quality and sequencing/assembly method on the ability to annotate complex genes of the immune system, using a case study the five marsupial genomes and one monotreme genome of varying quality. While the conclusions the authors present are not particularly surprising given what we know about genome assembly, this manuscript does a nice job outlining the reasons why higher quality (in particular, long-read) assemblies are important to facilitate annotation of these critical genes, and exploring in depth the

impact of various aspects of assembly quality. The authors present their results in a convincing and clear way, and this work provides a useful summary for the genomics community.

I do have some minor comments that I hope will help improve this work, listed below.

1. The conditional "in wildlife" is perhaps a little confusing in the title, as I believe the issues the authors raise should be widely relevant to vertebrate, or at least mammalian, genomes, and "wildlife" is a term with varying colloquial definitions among the readership of Gigascience. Relatedly the discussion in the background section of the abstract, as well as the intro of the manuscript and some parts of the discussion, could probably focus on mammals generally, or even vertebrates, not wildlife specifically. It would also make sense to make the implicit vertebrate focus explicit.

RESPONSE: The authors agree that the issues raised in our manuscript would be applicable to many mammalian or vertebrate genomes. However, genomics projects for non-model species such as wildlife generally work within constraints that are not always applicable to mammalian or vertebrate genomes more broadly. These include budget considerations, access to samples (remote locations, permits, CITES listing, threat status) and sample quantity (volume and tissue types available, sample quality (opportunistic sampling, non-invasive samples, sub-optimal preservation method, no access to liquid nitrogen or -80 freezer), amongst many others. All these factors influence the type of genome sequencing available to wildlife genomics projects, and hence resulting assembly quality. Mammals and many vertebrates more broadly, do not generally face these multitude of challenges when generating reference genomes. While the link between input sample, assembly quality and curation to generate a high-quality assembly has been established in wildlife (Rhie et al 2021), what has not been assessed is the impact of assembly quality on functionally important regions of the genome, such as immune genes. Our aim was to provide guidance for the wildlife genomics community, particularly those working on species impacted by disease, on how different genome sequencing strategies impact quality of immune gene annotations.

REVIEWER COMMENT: 2. The introduction goes into extensive detail about the case study systems presented here - perhaps more detail than is really needed (e.g., lines 130 - 136 on DFTD and chlamydial vaccines). However, there is little background information about the specific immune gene families that are the focus of this work. The authors present a compelling argument for why studying these gene families is important, but some additional information to help guide readers who may not be expert in the specific immune families under discussion would be valuable. In particular, reminding readers why these genes in particular are such a challenge to annotate, with perhaps a brief overview of the six immune gene families that are the focus of the work.

RESPONSE: Additional detail regarding the six immune gene families that are the focus of the manuscript, and why immune genes are challenging to annotate has been provided in the introduction at lines 66-101 and below for easy reference.
“The COVID-19 pandemic is one of many examples which highlight the ever-increasing importance of understanding wildlife immunity and disease to better understand and manage disease spill over [17]. In the case of wildlife threatened by disease, conservation questions are more challenging to answer and typically involve immunogenetic diversity which relies on accurate immune gene annotations. Immune genes in mammals can be classified into six major families based on their evolutionary history and function: T cell receptors (TCR), immunoglobulins (IG), major histocompatibility complex (MHC), natural killer (NK) receptors, toll-like receptors (TLR) and cytokines. Mammals utilise two antigen recognition systems: TCR and IG expressed by T lymphocytes and B lymphocytes respectively. TCR and IG are encoded in large clusters within the genome, each of which contain few constant sequences that define the receptor sub-type, and multiple highly duplicated variable segments that recognise and bind antigens. The number and sequence polymorphism of IG and TCR V segments varies significantly between mammalian species [18-20]. Another major family of immune genes is the major histocompatibility complex which contains three classes of genes (class I, II and III). MHC class I and II genes encode cell-surface receptors which bind and present self- and pathogen-derived antigens to T lymphocytes, activating the adaptive immune response. Class I and II genes evolve via duplication and can be highly polymorphic, hence gene number differs between species [21, 22]. Natural killer (NK) cells directly kill virus-infected and cancerous cells and are an important component of innate immunity. Their activity is mediated via cell-surface receptors encoded by genes classified into two functionally similar but structurally dissimilar families; the leukocyte receptor complex (LRC) and natural killer complex (NKC). These families are encoded in separate clusters within the genome, and as they evolve via gene duplication, gene number varies significantly between species [23]. TLRs are membrane-spanning receptors expressed by immune and non-immune cells which bind pathogen-associated molecular

patterns (PAMP), activating the innate and adaptive immune response. Compared to other immune genes, TLRs gene number and sequence is relatively conserved across mammals [24]. Lastly, cytokines are small proteins secreted by numerous cell types which direct the immune response. Cytokines can be classified into multiple families including interferons (IFN), tumour necrosis factors (TNF) and interleukins (IL), and gene content within each family varies between mammals [25].
Immune genes are some of the most polymorphic regions of the genome, owing to the need to generate diversity in response to ever-changing pathogenic pressures [26, 27]. Diversity within these gene families is generated through gene duplication, gene copy number variation, SNPs and rapid evolution, resulting in a complex genomic organisation and high level of pseudogenization [26]. Generally, immune genes are encoded within repetitive clusters in the genome, especially highly duplicated families such as the MHC and NK receptors [28]. Given these factors, accurate assembly and annotation of genomic regions encoding immune genes can be challenging [29-31], especially in wildlife."

REVIEWER COMMENT: 3. I would recommend ordering the species in Table 1, Table 2, Figure 1, Figure 2, and Figure 3 in a consistent order, perhaps from highest to lowest contig N50. This will help readers keep track of the key patterns.

RESPONSE: Ordering of species and immune families in figures and tables (except for table 1) in the main manuscript and Additional file 2 is now consistent with the reviewer's suggestion. Species are presented in the order of platypus, koala, woylie, wombat, antechinus then numbat, and immune families are presented in the order of cytokines, TLR, MHC, NKC, LRC, IG and TCR.

REVIEWER COMMENT: 4. The authors present a qualitative assessment of the kinds of genes where automated annotation fails in lines 202-212 and Fig 3. However a quantitative breakdown here would also I think be useful to the community, and should be easy to generate. One could simply list the fraction of manually annotated genes correctly recovered (and completely missed with <10% overlap) for each class in Table 2 for each species. This would also allow the authors to put some numbers alongside statements in this paragraph like "Most of these genes comprised... [line 210]"

RESPONSE: ≥90% and ≤10% overlap in genomic coordinates between manual and automated annotation of immune genes has been added for each species and immune family in table 2. A quantitative breakdown has been added to this section of the results. See lines 235-255 and below. The authors have also added additional detail regarding automated versus manual immune annotation at the exon-level for the TCR, IG and LRC families which were poorly annotated by automated pipelines at the gene-level.
Lines 235-255
"A breakdown of this analysis by immune family revealed that marsupial- and monotreme-specific immune genes which are not orthologous to those in eutherians were generally poorly annotated, regardless of automated pipeline or genome quality (Supplementary Figure 1). This was particularly the case for TCR and IG gene families, with up to 88% of genes in these families incorrectly annotated by automated pipelines (≤10% overlap) amongst the six species (Table 2). This is likely due to highly duplicated variable gene segments that don't encode conventional exon-intron splice sites which may hinder annotation with automated pipelines. Poor gene annotations of TCR and IG families was somewhat recovered at the exon level, as some TCR and IG variable gene segments were annotated as exons by automated pipelines. Correct annotation (≥90% overlap) of the TCR family increased from 0-2% at the gene level to 2-15% at the exon level amongst the six genomes (Supplementary Figure 2). This improvement was even greater for the IG family, with an increase from 0-2% correct annotation at the gene level to 15-43% at the exon level amongst the six genomes (Supplementary Figure 2). Despite this, up to 67% of TCR and IG variable segments were still not annotated at the exon level (0% overlap) amongst the six genomes, highlighting the difficulty in automated annotation of these regions. Similarly, marsupial-specific gene expansions within the leukocyte receptor complex (LRC) and monotreme-specific gene expansions within the natural killer complex (NKC) family of NK receptors were also poorly annotated by automated pipelines (Supplementary Figure 1). As with TCR and IG families, correct annotation increased from the gene- (0-28% marsupial LRC, 31% platypus NKC) to exon-level (6-65% marsupial LRC, 79% platypus NKC) (Table 2, Supplementary Figure 2), likely due to the presence of variable numbers of duplicated immunoglobulin superfamily (IGSF) domains and C-type lectin (CLEC) domains within each LRC and NKC gene respectively."

REVIEWER COMMENT: 5. I am not sure the statement (298-299): "that a kitchen sink approach, that uses long-read data combined with HiC technology, to generate a high-quality genome assembly is required to investigate immunity and disease in wildlife" is fully supported by the results the authors present. The annotation of the woylie genome, which as I understand it does not include any HiC

scaffolding, seems to be as good or nearly as good as the two kitchen sink genomes. I would propose that the key conclusion is that long-read data specifically (with or without HiC) and high contig N50 (probably at least 1 Mb) is what is required for a successful manual annotation of these complex immune genes. This issue resurfaces in the discussion section, where again the point that HiC + Illumina is not sufficient is quite clear, but the converse does not seem well supported: long-read data in the absence of HiC does just fine.

RESPONSE: The authors agree that this statement could be improved. Our results do support the reviewer's suggestion that assemblies based on long-read data, with or without scaffolding technology, are required for successful immune gene annotation. However, as outlined in the results section lines 298-320, immune gene families in the kitchen sink genomes represented by the 2021 platypus and koala assemblies were more intact than the woylie or 2018 platypus assembly (results presented in lines 368-380), both of which are based on long-read data. This was especially true for highly duplicated families such as the MHC, LRC NK receptors and TCR. The opening statement of the discussion has been modified to reflect the reviewer's suggestion, see lines 382-390 and below.
"By manually annotating immune genes in five marsupial genomes and two versions of the platypus genome, all varying qualities, we have confirmed that genome quality is directly linked to our ability to annotate complex immune gene families. Without long reads and scaffolding technologies, immune genes are scattered across many individual scaffolds and gene family organisation and evolution cannot be elucidated. We conclude that long-read data, with or without HiC technology, to generate a high-quality genome assembly with a contig N50 of at least 1MB is required to investigate immunity and disease in wildlife. However, a kitchen sink approach to genome sequencing and assembly will enable complete reconstruction of complex and duplicated families such as MHC, TCR and LRC NK receptors as in the platypus 2021 and koala genomes."

REVIEWER COMMENT: 6. The discussion of the limits of automated annotation is very important, but I found this section (starting on line 311) a little muddled. One key clarification is that it would probably be useful to separately discuss TCR and IG variable segments from all other immune genes. As the authors mention, automated analysis is not expected to successfully recover these variable regions, and it would probably be more useful to readers to get a sense of how automated analysis and RNA-seq alignment performs excluding these elements, in addition to the discussion on lines 329-337 of the specific challenges of variable regions.

RESPONSE: This section of the discussion has been revised in response to the reviewer's comment and additional detail added. Automated annotation and RNAseq support for immune genes other than TCR and IG is now discussed in lines 408-437, while TCR and IG are solely discussed in lines 424-434. See amended text below.
"Aside from TCR and IG, the majority of immune genes incorrectly annotated or missing from the automated annotations were divergent genes not orthologous to those in eutherian mammals, such as MHC, marsupial-specific gene expansions within the LRC and monotreme-specific gene expansions within the NKC. Given their divergence, these genes often have low or no BLAST homology to nucleotide or protein databases. Gene models generated by automated annotation software are hypotheses based on supporting evidence such as RNAseq data and homology to nucleotide and protein databases. While immune transcripts were identified in the transcriptomes from these species, RNAseq data only supported gene models for a low proportion of MHC, LRC and NKC genes. RNAseq data only supported 8-16% of LRC gene predictions and 16-37% of MHC gene predictions amongst the four marsupial genome annotations which used RNAseq data as gene model evidence (koala, woylie, antechinus and numbat). Similarly, around 60% of NKC genes within the platypus genomes were supported by RNAseq data. Overall, RNAseq data did not provide enough evidence to support gene models for ~20% of immune genes within the genome. Some immune genes may not have been expressed in the tissue sequenced, were expressed at low levels, or were fragmented. For human and mouse, comprehensive and curated gene sets such as GENCODE and RefSeq are available to guide gene model predictions, comprising data from more than 10,000 RNA experiments and decades of dedicated work in this field [95, 96]. Given time, budget and sample constraints for wildlife, these curated gene sets are not available, hence RNAseq evidence is incomplete resulting in deficient gene models by automated annotation software.
It is not surprising that TCR and IG V segments were poorly or not annotated by all automated pipelines used to annotate the genomes in this study. These genes are notoriously difficult to characterise and are manually annotated in the human and mouse genome on Ensembl using the International Immunogenetics Information System (IMGT) database [38, 97]. Alignment of mature IG and TCR sequences from RNAseq data to the genome results in poor automated annotation, as V segments utilize different sequence signal splice sites to introns, which are not recognized by the open reading frame

prediction algorithms. Indeed, RNAseq evidence only supported 7% to 18% of TCR V segment and 0% to 6.9%% of IG V segment gene predictions by automated pipelines amongst the four marsupial and platypus genomes. V sequences from three marsupials and two monotremes are available in IMGT, however as non-model species, they are not included in the scope for manual annotation by Ensembl or NCBI, so these important functional features are not annotated."

REVIEWER COMMENT: 7. Regarding "it is not a requirement for manual changes to annotations to be tracked between genome versions" on line 353, I am not sure this is so simple. Even lifting over the old manual curation to new assembly coordinates probably needs itself to be manually verified before one can be confident that the new model is correct. But I do not think this would mean the information is lost, as I believe NCBI and Ensembl both maintain old annotations and assembly versions.

RESPONSE: The authors agree that this statement was vague and so has been removed from the manuscript. While NCBI and Ensembl maintain old annotations and assembly versions, our argument still stands as there is currently limited scope to include manual gene annotations of the scale presented in our manuscript alongside existing automated annotations from these databases.

REVIEWER COMMENT: 8. Given that 10x linked reads are no longer available for genome assembly, the extensive discussion of their uses and limitations on lines 431-457 could probably be condensed considerably.

RESPONSE: This section of the discussion has been condensed, see lines 531—552 and text below. However, the authors feel discussing the limitations of 10x genomes for immune gene annotation is still warranted to make use of existing 10x assemblies, particularly for species where additional genome sequencing is unlikely due to sample or budget constraints.
"10x Chromium linked-read sequencing was insufficient to accurately re-assemble immune gene clusters in our study (Figure 4C). While this technology is no longer available for genome sequencing, acknowledging the limitations of this technology for immune gene annotation remains valid in order to make use of existing 10x genomes. Complete marsupial immune gene clusters can span hundreds of kilobases to megabases, as shown by annotation of the complete MHC, NK receptor and TC
