# Supplementary material for: Best genome sequencing strategies for annotation of complex immune gene families in wildlife
Source: Gigascience. 2022 Oct 30;11:giac100. doi: 10.1093/gigascience/giac100 (PMC9618407; doi:10.1093/gigascience/giac100)
Supplement: giac100_Response_to_Reviewer_Comments_Revision_1 [file giac100_response_to_reviewer_comments_revision_1.pdf]

Author's Response To Reviewer Comments

Close

Amendments made as requested to the data availability section

Close
