# Supplementary material for: Best genome sequencing strategies for annotation of complex immune gene families in wildlife
Source: Gigascience. 2022 Oct 30;11:giac100. doi: 10.1093/gigascience/giac100 (PMC9618407; doi:10.1093/gigascience/giac100)
Supplement: giac100_Reviewer_1_Report_Original_Submission — Michael Hiller -- 4/30/2022 Reviewed [file giac100_reviewer_1_report_original_submission.pdf]

Reviewer Report

Title: Best genome sequencing strategies for annotation of complex immune gene families in wildlife

Version: Original Submission **Date:** 4/30/2022

Reviewer name: Michael Hiller

Reviewer Comments to Author:

Knowledge about immune genes is critical for species conservation programs. However, immune genes occur in large gene clusters that are difficult to assemble and annotate. This important and timely study uses a number of marsupial genomes and the platypus to assess which sequencing technologies enable complete reconstructions of immune gene clusters and which methods enable annotations of these immune genes.
I have the following comments.
Since Fgenesh++ and Maker produce automatic annotations, I wonder why not all 6 genomes were annotated with these two methods? This would allow a comparison between Fgenesh++ against Maker. Maybe it is possible to annotate at least a few genomes with both methods.
Direct assessments of assembly quality should ideally be done on different assemblies of the same species to rule out real differences between species.
Would it be possible to include previous koala or platypus genome that was much more fragmented?
Figure 1 shows a useful of all immune genes. However, some genes like TLRs are actually easy to annotate as they are have a standard gene structure.
Therefore, it would be informative to provide in this figure a breakdown of how well the different immune gene families are annotates, as the authors nicely did in table 2.
This would inform on which immune genes are particularly difficult to annotate.
Figure 3B is not colorblind friendly.
Line 275: The discussion makes it clear that this is a scaffolding error and not a real inversion. This should be clarified here as well.
I fully agree with the value of the manual annotations. Therefore, it would be helpful to provide the manual annotations also as a gff3 or gtf file that provide the full exon structure. Additional file 2 only lists the start and end coordinates of genes with multiple exons. The assembly accession should also be listed.
As a suggestion: A haplotype-resolved assembly of a marsupial is likely not yet available, but such an assembly would provide an opportunity to further investigate the influence of assembly quality and haplotype variation in immune genes.

Methods

Are the methods appropriate to the aims of the study, are they well described, and are necessary controls included? Choose an item.

Conclusions

Are the conclusions adequately supported by the data shown? Choose an item.

Reporting Standards

Does the manuscript adhere to the journal's guidelines on minimum standards of reporting? Choose an item.

Choose an item.

Statistics

Are you able to assess all statistics in the manuscript, including the appropriateness of statistical tests used? Choose an item.

Quality of Written English

Please indicate the quality of language in the manuscript: Choose an item.

Declaration of Competing Interests

Please complete a declaration of competing interests, considering the following questions:

- Have you in the past five years received reimbursements, fees, funding, or salary from an organisation that may in any way gain or lose financially from the publication of this manuscript, either now or in the future?
- Do you hold any stocks or shares in an organisation that may in any way gain or lose financially from the publication of this manuscript, either now or in the future?
- Do you hold or are you currently applying for any patents relating to the content of the manuscript?
- Have you received reimbursements, fees, funding, or salary from an organization that holds or has applied for patents relating to the content of the manuscript?
- Do you have any other financial competing interests?
- Do you have any non-financial competing interests in relation to this paper?

If you can answer no to all of the above, write 'I declare that I have no competing interests' below. If your reply is yes to any, please give details below.

none

I agree to the open peer review policy of the journal. I understand that my name will be included on my report to the authors and, if the manuscript is accepted for publication, my named report including any attachments I upload will be posted on the website along with the authors' responses. I agree for my

report to be made available under an Open Access Creative Commons CC-BY license (http://creativecommons.org/licenses/by/4.0/). I understand that any comments which I do not wish to be included in my named report can be included as confidential comments to the editors, which will not be published.

Choose an item.

To further support our reviewers, we have joined with Publons, where you can gain additional credit to further highlight your hard work (see: https://publons.com/journal/530/gigascience). On publication of this paper, your review will be automatically added to Publons, you can then choose whether or not to claim your Publons credit. I understand this statement.

Yes Choose an item.
