# Supplementary material for: Best genome sequencing strategies for annotation of complex immune gene families in wildlife
Source: Gigascience. 2022 Oct 30;11:giac100. doi: 10.1093/gigascience/giac100 (PMC9618407; doi:10.1093/gigascience/giac100)
Supplement: giac100_Reviewer_3_Report_Original_Submission — Tim Sackton -- 5/11/2022 Reviewed [file giac100_reviewer_3_report_original_submission.pdf]

Reviewer Report

Title: Best genome sequencing strategies for annotation of complex immune gene families in wildlife

Version: Original Submission **Date:** 5/11/2022

Reviewer name: Tim Sackton

Reviewer Comments to Author:

In this manuscript, Peel et al examine the impact of assembly quality and sequencing/assembly method on the ability to annotate complex genes of the immune system, using a case study the five marsupial genomes and one monotreme genome of varying quality. While the conclusions the authors present are not particularly surprising given what we know about genome assembly, this manuscript does a nice job outlining the reasons why higher quality (in particular, long-read) assemblies are important to facilitate annotation of these critical genes, and exploring in depth the impact of various aspects of assembly quality. The authors present their results in a convincing and clear way, and this work provides a useful summary for the genomics community.

I do have some minor comments that I hope will help improve this work, listed below.

1. The conditional "in wildlife" is perhaps a little confusing in the title, as I believe the issues the authors raise should be widely relevant to vertebrate, or at least mammalian, genomes, and "wildlife" is a term with varying colloquial definitions among the readership of Gigascience. Relatedly the discussion in the background section of the abstract, as well as the intro of the manuscript and some parts of the discussion, could probably focus on mammals generally, or even vertebrates, not wildlife specifically. It would also make sense to make the implicit vertebrate focus explicit.

2. The introduction goes into extensive detail about the case study systems presented here - perhaps more detail than is really needed (e.g., lines 130 - 136 on DFTD and chlamydial vaccines). However, there is little background information about the specific immune gene families that are the focus of this work. The authors present a compelling argument for why studying these gene families is important, but some additional information to help guide readers who may not be expert in the specific immune families under discussion would be valuable. In particular, reminding readers why these genes in particular are such a challenge to annotate, with perhaps a brief overview of the six immune gene families that are the focus of the work.

3. I would recommend ordering the species in Table 1, Table 2, Figure 1, Figure 2, and Figure 3 in a consistent order, perhaps from highest to lowest contig N50. This will help readers keep track of the key patterns.

4. The authors present a qualitative assessment of the kinds of genes where automated annotation fails in lines 202-212 and Fig 3. However a quantitative breakdown here would also I think be useful to the community, and should be easy to generate. One could simply list the fraction of manually annotated genes correctly recovered (and completely missed with <10% overlap) for each class in Table 2 for each species. This would also allow the authors to put some numbers alongside statements in this paragraph like "Most of these genes comprised... [line 210]"

5. I am not sure the statement (298-299): "that a kitchen sink approach, that uses long-read data

combined with HiC technology, to generate a high-quality genome assembly is required to investigate immunity and disease in wildlife" is fully supported by the results the authors present. The annotation of the woylie genome, which as I understand it does not include any HiC scaffolding, seems to be as good or nearly as good as the two kitchen sink genomes. I would propose that the key conclusion is that long-read data specifically (with or without HiC) and high contig N50 (probably at least 1 Mb) is what is required for a successful manual annotation of these complex immune genes. This issue resurfaces in the discussion section, where again the point that HiC + Illumina is not sufficient is quite clear, but the converse does not seem well supported: long-read data in the absence of HiC does just fine.
6. The discussion of the limits of automated annotation is very important, but I found this section (starting on line 311) a little muddled. One key clarification is that it would probably be useful to separately discuss TCR and IG variable segments from all other immune genes. As the authors mention, automated analysis is not expected to successfully recover these variable regions, and it would probably be more useful to readers to get a sense of how automated analysis and RNA-seq alignment performs excluding these elements, in addition to the discussion on lines 329-337 of the specific challenges of variable regions.
7. Regarding "it is not a requirement for manual changes to annotations to be tracked between genome versions" on line 353, I am not sure this is so simple. Even lifting over the old manual curation to new assembly coordinates probably needs itself to be manually verified before one can be confident that the new model is correct. But I do not think this would mean the information is lost, as I believe NCBI and Ensembl both maintain old annotations and assembly versions.
8. Given that 10x linked reads are no longer available for genome assembly, the extensive discussion of their uses and limitations on lines 431-457 could probably be condensed considerably.

Methods

Are the methods appropriate to the aims of the study, are they well described, and are necessary controls included? Choose an item.

Conclusions

Are the conclusions adequately supported by the data shown? Choose an item.

Reporting Standards

Does the manuscript adhere to the journal's guidelines on minimum standards of reporting? Choose an item.

Choose an item.

Statistics

Are you able to assess all statistics in the manuscript, including the appropriateness of statistical tests used? Choose an item.

Quality of Written English

Please indicate the quality of language in the manuscript: Choose an item.

Declaration of Competing Interests

Please complete a declaration of competing interests, considering the following questions:

- Have you in the past five years received reimbursements, fees, funding, or salary from an organisation that may in any way gain or lose financially from the publication of this manuscript, either now or in the future?
- Do you hold any stocks or shares in an organisation that may in any way gain or lose financially from the publication of this manuscript, either now or in the future?
- Do you hold or are you currently applying for any patents relating to the content of the manuscript?
- Have you received reimbursements, fees, funding, or salary from an organization that holds or has applied for patents relating to the content of the manuscript?
- Do you have any other financial competing interests?
- Do you have any non-financial competing interests in relation to this paper?

If you can answer no to all of the above, write 'I declare that I have no competing interests' below. If your reply is yes to any, please give details below.

I declare that I have no competing interests

I agree to the open peer review policy of the journal. I understand that my name will be included on my report to the authors and, if the manuscript is accepted for publication, my named report including any attachments I upload will be posted on the website along with the authors' responses. I agree for my report to be made available under an Open Access Creative Commons CC-BY license (http://creativecommons.org/licenses/by/4.0/). I understand that any comments which I do not wish to be included in my named report can be included as confidential comments to the editors, which will not be published.

Choose an item.

To further support our reviewers, we have joined with Publons, where you can gain additional credit to further highlight your hard work (see: https://publons.com/journal/530/gigascience). On publication of this paper, your review will be automatically added to Publons, you can then choose whether or not to claim your Publons credit. I understand this statement.

Yes Choose an item.
